# 2024 ACVIM Forum Research Abstract Program

**DOI:** 10.1111/jvim.17182

**Published:** 2024-08-28

The American College of Veterinary Internal Medicine (ACVIM) Forum and the Journal of Veterinary Internal Medicine (JVIM) are not responsible for the content or dosage recommendations in the abstracts. The abstracts are not peer reviewed before publication. The opinions expressed in the abstracts are those of the author(s) and may not represent the views or position of the ACVIM. The authors are solely responsible for the content of the abstracts.

**2024 ACVIM Forum**

**June 5–October 31, 2024**

**Research Abstract Oral Program**

**Index of Abstracts**

**Table:** 

**CARDIOLOGY**			
**Presenting Author**	**Abstract Title**	**Sequence Number**	**Award Eligible**
Elise Lenske	Hemodynamic, Echocardiographic, Electrocardiographic, and Behavioral Effects of Oral Gabapentin in Healthy Dogs	C01	x
Kara Maneval	Correlation of Echocardiographic and Angiographic Measurements of Pulmonary Annular Diameter in Dogs with Pulmonary Stenosis	C02	x
Joe Herbert	Multicentre Per‐catheter Ductal Occlusion in Small Dogs; Device Selection, Patient Characteristics, Outcomes, and Complication Rates	C03	x
Cortney Pelzek	Effect of Orientation and Additional Planes in the Echocardiographic Determination of Three‐Dimensional Left Ventricular Volume	C04	x
Sumana Prabhakar	Feasibility, Efficiency and Reproducibility of Left Ventricular Volume Estimation by Two‐Dimensional and Three‐Dimensional Echocardiography	C05	x
Andrea Plá	Effects of Oral Single‐Dose Empagliflozin on Urine Glucose and Serum β‐Hydroxybutyrate in Healthy Adult Dogs	C06	x
Brad Lytle	Comparison of Low‐ and High‐pressure Balloon Valvuloplasty in Dogs with Severe Pulmonary Valve Stenosis	C07	x
Luke Dutton	Association of the TTN and PDK4 Gene Variants with Dilated Cardiomyopathy in British Doberman Pinschers	C08	x
Marisa Ciccozzi	Multicenter Transvenous Pacemaker Implantation in 595 Dogs: Complications and Survival Rates	C09	x
Christianna Ziccardi	Aldosterone Breakthrough During Enalapril or Telmisartan Therapy in Dogs with Advanced Myxomatous Mitral Valve Disease	C10	x
James Wood	Characterization of the Tissue Renin‐angiotensin‐aldosterone System in Normal Cats and Cats with Hypertrophic Cardiomyopathy	C11	x
I‐Jung Chi	Pimobendan Alters Mitral Annular Dynamics and Reduces Mitral Regurgitation in Dogs: Cardiac Computed Tomographic Assessment	C12	x
Evan Ross	Effects of General Anesthesia on Echocardiographic Indices of Pulmonary Stenosis Severity in Dogs	C13	x
Brian Scansen	Perfusion Computed Tomography of Canine Pelvic Limb Vasculature After Femoral Arterial Catheterization: Ligation vs Repair	C14	
Victor Rivas	CK‐586 Reduces Contractility and Ameliorates Obstruction in Feline Hypertrophic Cardiomyopathy	C15	
Poppy Bristow	Short Term Survival Following Mitral Valve Repair Surgery in 43 Dogs	C16	
Lalida Tantisuwat	Proximity of Coronary Arteries to Right Ventricular Outflow Tract in Dogs with Pulmonary Valve Stenosis	C17	
Lalida Tantisuwat	Invasive Pressures During Right Heart Catheterization in Canine Pulmonary Stenosis	C18	
Ashley Saunders	Patent Ductus Arteriosus Characterized by Transesophageal Echocardiography in Dogs	C19	
George Kramer	Trans‐Jugular Transseptal Delivery of the CoApt Valve in a Canine Model	C20	
Annie Showers	Comparison of Three‐dimensional Mitral Valve Morphologic Measurements Obtained Using Two Different Ultrasound Machines	C21	x
Lalida Tantisuwat	Cardiac Computed Tomographic Assessment of the Pulmonary Outflow Tract in Dogs with Pulmonary Valve Stenosis	C22	
Maria Luz Wang	Echocardiographic Features and Survival of Dogs with Constrictive Pericarditis Secondary to Coccidioidomycosis	CR01	x
**EQUINE**			
**Presenting Author**	**Abstract Title**	**Sequence Number**	**Award Eligible**
Patricia Egli	Characterization of Equine Platelet Lysate for Nebulization in the Horse	E01	x
Lauren Holley	Multi‐Dose Preserved Lidocaine Nebulization Does Not Alter Respiratory Microbiota or Inflammatory Markers in Healthy Horse	E02	x
Ananya Mahalingam‐Dhingra	An In‐Hospital Clinical Trial Assessing Nebulized Lidocaine Compared to Saline for Treatment of Equine Asthma.	E03	x
Clara Raisky	Effect of Steamed Hay on Horses with Severe Equine Asthma in Remission	E04	x
Florence Dupuis‐Dowd	Antimicrobial Susceptibility Results of Streptococcus Equi Subsp. Equi Isolates from Horses	E05	x
Camille RUAULT	Androgen Administration in Severe Equine Asthma: A Blinded, Cross‐over, Randomized Study	E06	x
Brittnee Sayler	Pharmacokinetics of Chloramphenicol in Horses Following Rectal and Nasogastric Administration	E07	x
Mallory Lehman	Pharmacokinetics and Safety of Oral Fluralaner in Healthy Horses	E08	x
Megan Palmisano	Vitamin E Concentration in Horses Following Hospitalization	E09	x
Kali Slavik	Characterization of Renal Lipidosis in Equids: A Postmortem Case–Control Study (2008‐2022)	E10	x
Xueli Wang	Decline of Maternal Antibody and Natural Flavivirus Infection in Foals	E11	x
Flavie Payette	Diagnosis of Bacteremia in Neonatal Foals Using High‐Throughput Sequencing: A Pilot Study	E12	x
Diego Gomez	Risk Factors for Mortality of Sick Foals Admitted to a Tertiary Hospital in Ontario, Canada	E13	
Caitrin Lowndes	Effects of Two Different Pioglitazone Dosage Regimens on Insulin Dynamics in Severely Insulin Dysregulated Equids	E14	
Akos Kenez	Metabolomic Alterations Associated with Allergic Response in Equine Insect Bite Hypersensitivity	E15	
Javier Perez Quesada	Biomarkers of Brain Injury in Foals with Neonatal Maladjustment Syndrome	E16	x
Allison Palmer	A High‐protein Meal is Associated with Increased Glucose‐dependent Insulinotropic Polypeptide Secretion in Insulin‐dysregulated Horses	E17	x
Megan Palmisano	Assessment of Cardiovascular Structural Changes and Non‐invasive Blood Pressure in Warmblood Horses with Insulin Dysregulation	E18	x
Emma Stapley	Novel Fibrosis Scoring and Automated Histopathology Offer a Onehealth Approach to Equine Adipose Tissue Abnormalities	E19	x
Wenqing Wang	Pituitary Histomorphometry Correlation with Adrenocorticotrophin Response to Thyrotropin‐releasing Hormone in Pituitary Pars Intermedia Dysfunction Diagnosis	E20	
Jonathan Turck	Fecal Concentrations of Fatty Acids and Sterols in Horses with Colitis	E21	
Bettina Wagner	Intramuscular EHV Vaccination Results in Systemic and Mucosal Antibodies	E22	
Amanda Samuels	Exploring the Diagnostic Utility of Neutrophil Activation Markers in Hospitalized Foals	E23	x
Carolyn Arnold	Comparison of 16S rRNA, Shallow Shotgun, and qPCR for Taxonomic Characterization of Equine Fecal Microbiota	E24	
Sharanne Raidal	Comparison of Mask and Binasal Prongs for Delivery of Non‐invasive Ventilation to Foals	E25	
Georgia Skelton	Evaluation of Digital Radiographic Measurements for the Diagnosis of Acute Laminitis	E26	
Kate Kemp	Phenylbutazone Improves Insulin Sensitivity and Reduces Insulin Secretion in Horses with Insulin Dysregulation	E27	
Rachel Lemcke	Prevalence of Pituitary Pars Intermedia Dysfunction and Insulin Dysregulation and Endocrine‐Associated Clinical Signs in Ponies	E28	
**FOOD ANIMAL**			
**Presenting Author**	**Abstract Title**	**Sequence Number**	**Award Eligible**
Trey Neyland	The Effect of Flunixin Meglumine on Viral Shedding in Calves	F01	x
Zuzanna Sikorska	Abomasal Displacement in Pre‐weaned Dairy Calves–27 Cases (2018‐2023)	F02	x
Alyssa Sullivan	Twenty‐four‐hour Electrocardiographic Monitoring for Assessment of Cardiac Arrhythmias in Healthy and Hospitalized Goats	F03	x
Alyssa Sullivan	Clinical Findings and Outcome of Goats Diagnosed with Discospondylitis and Vertebral Osteomyelitis on Computed Tomography	F04	x
**NEUROLOGY**			
**Presenting Author**	**Abstract Title**	**Sequence Number**	**Award Eligible**
Zoe Bailey	Effect of a Probiotic on Seizure Frequency in Dogs with Idiopathic Epilepsy Receiving Anti‐epileptic Drugs	N01	x
Megan Wolfe	Integrated Endoscopic Mini‐Hemilaminectomy and Thoracolumbar Lateral Corpectomy in Cadaveric Dogs	N02	x
Jessica Wagner	Single‐dose Pharmacokinetics of Intranasal Levetiracetam in Healthy Dogs	N03	x
Mathieu Boutin	Accuracy of a Canine and Feline CT‐guided 3D‐printed Stereotactic Brain Biopsy Guide Using Dental Anchors	N04	x
Adrien Dupanloup	Perioperative Assessment of Electroencephalography in Dogs with Congenital Portosystemic Shunts	N05	x
Go Togawa	Prognostic Utility of F‐waves in Paraplegic Dogs with Absent Pain Perception from Intervertebral Disc Extrusion	N06	x
Adrien Dupanloup	Diffusion‐Weighted Imaging of Intracranial Ring‐enhancing Lesions	N07	x
Rachel Geiger	Canine Meningiomas: Surgical Resection versus Radiation Therapy	N08	x
Lizabeth Lueck	Evaluation of Plasma Biomarkers in Cats With and Without Evidence of Feline Cognitive Dysfunction	N09	x
Maria Johnson	Vaccination and Seasonality as Risk Factors for Development of Auto‐immune Meningoencephalitis in Dogs	N10	
Katherine Simon	A Randomized Controlled Clinical Trial of senolytic and NAD+ Precursor in Aged Companion Dogs	N11	
Kathryn Dalzell	Creation of a Breed‐Specific Neurologic Disease Online Database for Veterinary Practitioners	N12	
Quentin Reuet	Metabolic Acidosis Associated with Zonisamide Administration in Dogs with Idiopathic Epilepsy: A Prospective Study	N20	
**NUTRITION**			
**Presenting Author**	**Abstract Title**	**Sequence Number**	**Award Eligible**
Allison McGrath	Assessment of Lean, Fat, and Total Body Mass Changes with Age in Dogs and Cats	NM03	
Jenessa Winston	Utility of FitBark to Monitor Activity in Obese Dogs Undergoing a Structured Weight Loss Program	NM04	
Elizabeth Morris	Therapeutic Renal Diets Differentially Influence Ionized and Total Calcium in Cats with Early‐stage Renal Disease	NM05	
**ONCOLOGY**			
**Presenting Author**	**Abstract Title**	**Sequence Number**	**Award Eligible**
Nicole Gibbs	Hypoxia‐inducible Factor 1α Expression in Canine Urothelial Carcinoma	O01	x
Kathleen Bardales	Transcriptional Profiling of the Immune Tumor Microenvironment in Canine Cutaneous Mast Cell Tumors	O02	x
Guannan Wang	Unveiling Shared Oncogenic Mutations and Signaling Pathways in Canine and Human Hepatocellular Carcinoma	O03	
Andrew Poon	Comparative Analysis of Metabolic Flexibility in Canine and Murine Osteosarcoma Cells	O04	
Leah Dunston	Alkaline Phosphatase and Mast Cell Tryptase Labeling in Canine Skin‐Associated Mast Cell Tumors	O05	
Yiyu Li	Retrospective Analysis of Canine Testicular Tumors: 101 cases	O06	
Dennis Ronzani	Unraveling Correlations Between Exercise and Neoplastic Disease in Golden Retrievers Using Big Data Analysis	O07	
Doyun Kim	A Preliminary Prospective Study: Sorafenib in Aggressive Canine Carcinomas–Tolerability and Clinical Efficacy	O08	
Laura Machado Ribas	MicroRNA Differential Expression Analysis for Identification of Diagnostic Biomarkers for Canine Visceral Hemangiosarcoma	ON01	
**SMALL ANIMAL INTERNAL MEDICINE**–**ENDOCRINOLOGY**			
**Presenting Author**	**Abstract Title**	**Sequence Number**	**Award Eligible**
Oliver Waite	Epidemiology of Diabetes Mellitus in Cats Under British Primary Veterinary Care in 2019	EN01	x
Alice Watson	Steroid Profiles in Cats with Primary Hyperaldosteronism	EN02	x
Marilou Castonguay‐Poirier	Long‐term Safety and Efficacy of Oral Bezafibrate Use in Dogs with Hypertriglyceridemia	EN03	x
Camille Brassard	Evaluation of a Feline‐Optimized TSH Assay in Cats with Hyperthyroidism and with Non‐Thyroidal Illness	EN04	x
Mathieu Paulin	Plasma Arginine Vasopressin and Serum Copeptin Concentrations Under Hypo‐, Iso‐ and Hyper‐osmolar Conditions	EN05	
Jake Salzman	Duration of Sedation Effects on the Adrenocorticotrophic Hormone Stimulation Test in Healthy Dogs	EN06	x
Emily Cohen	Transmucosal Glucagon Rapidly Increases Blood Glucose Concentration in Healthy Cats	EN07	
Anna Kraemer	Relationship Between Hemoglobin A1c and Fructosamine Concentrations and Survival in Diabetic Dogs	EN08	
**SMALL ANIMAL INTERNAL MEDICINE**–**GASTROENTEROLOGY**			
**Presenting Author**	**Abstract Title**	**Sequence Number**	**Award Eligible**
Yi Kwan Lee	Autologous Oral Fecal Microbiota Transplantation and Microbiome Recovery After Antibiotic Treatment, a Randomized Controlled Trial	GI01	x
Aditi Handa	Impact of Omeprazole on Esophageal Microbiota in Dogs Using a Minimally Invasive Sampling Method	GI02	x
Caylie Voudren	Gastrointestinal Microbiome Relationship to Plasma Glucagon‐Like Peptide‐2 in Dogs with Idiopathic Chronic Enteropathies	GI03	x
Sydney Oberholtzer	Internists Preferred Methods of Colonoscopy Preparation and Perceived Success of Protocols in Dogs and Cats	GI04	x
Alexis Hoelmer	Management of Acute Diarrhea in Dogs: A Questionnaire of United States Veterinarians	GI05	x
Katerina Moraiti	Serum Cobalamin and Folate Concentrations, Dysbiosis Index, and Histopathological Findings in Cats with Chronic Enteropathy	GI06	
Meg Nakazawa	Inflammatory Cytokines Suppress Expression of Tight Junction and Stemness‐Related Properties in Canine Intestinal Organoids	GI07	
Nina Kristen Randolph	The Long‐term in Vitro Bacterial Viability of Lyophilized and Frozen Feline Fecal Microbial Transplantation Products	GI08	
Romy Heilmann	Ileal and Colonic Renin‐angiotensin‐aldosterone System (RAAS) Receptor Dysregulation and Fibrosis Markers in Canine Chronic Enteropathy	GI09	
Yoko Ambrosini	3D Morphogenesis in Canine Gut‐on‐a‐Chip with Healthy and IBD Biopsy‐Derived Organoids	GI10	
Connie Rojas	Defining the Core Microbiome in Healthy Dogs	GI11	
Chih‐Chun Chen	Concentrations of Fecal Carbohydrates in Dogs with Chronic Enteropathy	GI12	
Patrick Barko	Microbial Indole Catabolites of Tryptophan Regulate Mucosal Barrier Function and Inflammatory Responses in Canine Colonoids	GI13	
Nina Kristen Randolph	The Long‐term in Vitro Bacterial Viability of Lyophilized and Frozen Canine Fecal Microbial Transplantation Products	GI14	
Nina Kristen Randolph	The In Vitro Bacterial Viability of Commercially Available Veterinary Fecal Microbial Transplantation Products	GI15	
Yu‐An Wu	Twice Daily Modified‐Cyclosporine for the Treatment of Chronic Pancreatitis in Cats	GI16	
Holly Ganz	Reasons for Donor Attrition in a Companion Animal Stool Bank	GI17	
Aarti Kathrani	Electrolyte Imbalances in Cats with Chronic Inflammatory Enteropathy	GI18	
Chi‐Hsuan Sung	Serum Concentrations of 7α‐hydroxy‐4‐cholesten‐3‐one (C4) in Dogs with Chronic Enteropathy	GI19	
Jyoti Kalita	Initial Studies on Fecal Microbiota Transplantation in Dogs: An Adjunct Therapy in Canine Parvoviral Diarrhea	GI20	
Nicole Akers	Effects of Prokinetic Drugs in Dogs with Clonidine‐delayed Gastric Emptying	GI21	
Jenessa Winston	Bile Acid Dysmetabolism in Feline Chronic Kidney Disease is Associated with 26 Peptacetobacter Hiranonis Variants	GI31	
**SMALL ANIMAL INTERNAL MEDICINE**–**HEMATOLOGY**			
**Presenting Author**	**Abstract Title**	**Sequence Number**	**Award Eligible**
Jade Peralta	Metabolic and Genomic Analyses in a Dog with Green Urine Identify a Biliverdin Reductase Defect	HM01	x
Victoria Neale	Temporal Associations Between Vaccination and Onset of Immune‐mediated Hemolytic Anemia or Thrombocytopenia in Dogs.	HM02	
**SMALL ANIMAL INTERNAL MEDICINE**–**HEPATOLOGY**			
**Presenting Author**	**Abstract Title**	**Sequence Number**	**Award Eligible**
Nozomi Shiohara	Feasibility of Shear Wave Elastography for Inflammation and Fibrosis in Dogs with Hepatic Disease	HP01	
Min Chun Chen	Detection and Phylogenetic Analysis of Domestic Cat Hepadnavirus in Blood from Cats in Texas	HP02	
Itsuma Nagao	Comprehensive Gene Expression Analysis in Gallbladder Mucosal Epithelial Cells of Dogs with Gallbladder Mucocele	HP03	
Floris Dröes	Point‐of‐Care Viscoelastometric Evaluation of Dogs with Congenital Intrahepatic Portosystemic Shunts	HP04	
Deborah Linder	Hepatic Copper Accumulation in Dogs: An Exploratory Study on Assessment of Dietary Factors	HP05	
Adrian Tinoco‐Najera	Untargeted Profiling of Serum Metabolites in Dogs with Chronic Hepatitis	HP06	
**SMALL ANIMAL INTERNAL MEDICINE**–**IMMUNOLOGY**			
**Presenting Author**	**Abstract Title**	**Sequence Number**	**Award Eligible**
Bianca Lara	Large Scale, Retrospective Study of Vaccine Associated Adverse Events in Cats	IM01	x
Lauren Chittick	In Vivo Effects of Methadone Administration on Immune Function in Healthy Dogs	IM02	
Yi‐Jen Chang	Effect of Cyclosporine on Activated T Cell interleukin‐2 Expression in Canine Hepatic Tissue	IM03	
**SMALL ANIMAL INTERNAL MEDICINE**–**INFECTIOUS DISEASE**			
**Presenting Author**	**Abstract Title**	**Sequence Number**	**Award Eligible**
Shino Yoshida	Treatment Response, Course of Improvement, and Follow‐up Information with Molnupiravir Treatment for Feline Infectious Peritonitis	ID03	
Constanca Pomba	Klebsiella Pneumoniae in Pets: Carbapenem Resistance Surveillance and Invasive Disease in Veterinary Care	ID04	
Constanca Pomba	A Preliminary Insight on the Molecular Epidemiology of Feline Immunodeficiency Vírus in Portugal, Europe	ID05	
**SMALL ANIMAL INTERNAL MEDICINE**–**NEPHROLOGY/UROLOGY**			
**Presenting Author**	**Abstract Title**	**Sequence Number**	**Award Eligible**
Adam Hunt	A Pilot Study of Burst Wave Lithotripsy for Treatment of Obstructive Ureteroliths in Cats	NU01	x
Danielle LaVine	Estimates of Urinary Calcium Excretion in Dogs with and Without Calcium Oxalate Urolithiasis	NU02	x
Alisa Berg	Kidney Injury Biomarkers are More Correlated with Proteinuria than Serum Creatinine in Telmisartan Treatment Study	NU03	x
Jasmine Zaibek	Retrospective Evaluation of Complications Associated with Ultra‐sound Guided Percutaneous Renal Biopsy in Dogs.	NU04	x
Sara Wilkes	Urinary Protein Banding Patterns as Potential Biomarkers of Non‐Neoplastic Prostatic Diseases in Intact Male Dogs	NU05	x
Riley Claude	Determining Within‐individual and Between‐subject Biological Variation in Urine Ammonia‐to‐creatinine Ratio in Healthy Adult Dogs	NU06	x
John Shamoun	Utilizing the Ellik Evacuator During Cystoscopic Retrieval of Uroliths in 12 Dogs: A Descriptive Study	NU07	x
Jason Bestwick	Feline Small Intestine Regional Distribution of mRNA Expression of Phosphate Cotransporters and Intestinal Alkaline Phosphatase	NU08	
Jane Huang	Evaluation of the Circulating Renin‐Angiotensin‐Aldosterone System in Cats with Surgically Induced Chronic Kidney Disease	NU09	
Rankyung Jung	Urine Protein Electrophoresis in Dogs with Systemic Inflammatory Response Syndrome	NU10	
Nuttha Hengtrakul	Osteogenic Phenotype in Feline Renal Mineralization: Implications for Kidney Stone Prevention	NU11	
Susan Carr	Investigation of a Novel Low‐Dose Sedation Protocol for Canine Urodynamic Studies	NU12	
Carrie Palm	Phosphate Kinetics During Intermittent Hemodialysis in Dogs with Acute Kidney Injury	NU13	
Emily Coffey	Serum Lipidomic and Metabolomic Profiling in Miniature Schnauzer Dogs With and Without Calcium Oxalate Urolithiasis	NU14	
Joanna White	Immune Complex‐Mediated Glomerulonephropathy in Australian and New Zealand Dogs	NU15	
**SMALL ANIMAL INTERNAL MEDICINE**–**OTHER**			
**Presenting Author**	**Abstract Title**	**Sequence Number**	**Award Eligible**
Conner Hayes	Hypoalbuminemia Associated with Decreased Survival in Cats Presenting to a Tertiary Hospital: Retrospective Case–Control Study	OT01	x
Eva Kao	Oxidative Stress Induces Phosphatidylserine Externalization in Canine Erythrocytes In Vitro	OT02	x
Kimberly Kuhlman	Impact of Generative AI on Veterinary Record‐Keeping: A Case Study	OT03	
Gerard O'Leary	Evaluating the Efficacy of NxVET, a Multi‐Functional Wearable Medical Device for Animal Health Monitoring	OT04	
Nora Jean Nealon	Age‐Associated Changes in the Global, Untargeted Serum Metabolome of Healthy Client‐Owned Domestic Cats	OT05	
Matina Pitropaki	Primary Hyperlipidemia in Miniature Schnauzers in Europe	OT06	
Amelia Frye	Gastrointestinal Antimicrobial Resistance in Cats Treated with Antibiotics, a Prospective Cohort Study	OT07	
**SMALL ANIMAL INTERNAL MEDICINE**–**PHARMACOLOGY**			
**Presenting Author**	**Abstract Title**	**Sequence Number**	**Award Eligible**
Yishan Kuo	Single‐Dose, Intravenous and Oral Pharmacokinetics of Isavuconazole In Dogs	PH01	x
Nicole Alleva	Comparison of Dissolution Profiles of Human Oral Generic Cyclosporine to Atopica®	PH02	x
Jean‐Baptiste Jentzer	Palatants Increase the Voluntary Intake of Placebo Tablets by Cats and Dogs	PH03	
**SMALL ANIMAL INTERNAL MEDICINE**–**RESPIRATORY**			
**Presenting Author**	**Abstract Title**	**Sequence Number**	**Award Eligible**
Iliana Navarro	Comparison of Sedated Respiratory‐Gated Computed Tomography (CT) to Anesthetized Inspiratory:Expiratory Breath Hold CT in Dogs	RS01	x

**Research Abstract ePoster Program**

**Index of Abstracts**

**Table:** 

**CARDIOLOGY**			
**Presenting Author**	**Abstract Title**	**Sequence Number**	**Award Eligible**
Jeongmin Lee	Clinical Outcomes Through 3 Months in Dogs with MMVD Treated with the TEER	C23	
Emily Suess‐Radford	The Angiotensin‐converting Enzyme Polymorphism in the North American Irish Wolfhound: Variant‐positive Prevalence and Clinical Implications	C25	x
Soh‐Yeon Lee	Association Between the Mitral Insufficiency Echocardiographic Score and Progression of Myxomatous Mitral Valve Disease	C28	
Aimi Yokoi	The Impact of Vericiguat, a Soluble Guanylate Cyclase Stimulator, on Cardiovascular Properties in Baroreflex‐absent Dogs	C29	
SINDHU RAJAN	Insight into the Association of Angiotensin Converting Enzyme Gene Polymorphism with Dilated Cardiomyopathy in Dogs	C30	
Matthew Denton	Assessment of Electrocardiography and Thoracic Radiography to Identify Echocardiographic Structural Heart Disease in Cats.	C31	
Kentaro Kurogochi	Outcome of Canine Mitral Valve Repair for Myxomatous Mitral Valve Disease with Severe Pulmonary Hypertension	C32	
Zack English	Ratio of Vena Contracta Width to Mitral Commissural Diameter in Canine Degenerative Mitral Valve Disease	C33	
Bobbie Ditzler	The Role of Vector‐borne Pathogens and Striatin Genotype in Boxers with Arrhythmogenic Right Ventricular Cardiomyopathy	C34	
Jeongmin Lee	Transcatheter Edge‐to‐Edge Mitral Valve Repair for Myxomatous MItral Valve Disease in Dogs Under 2.5 kg	C35	
Cosette Ayoub	Investigations of Asymmetric Dimethyl Arginine (ADMA) as a Biomarker in Preclinical Myxomatous Mitral Valve Disease	C36	
Kailah Buchanan	Changes to Pacemaker Programming Parameters over Time in Dogs	C37	
Mio Ishizaka	A Prospective Comparative Study on Novel and Conventional Inotropic Drugs in Clinically Healthy Cats	C38	
Jennifer Applebaum	Assessment of the Eko Core Stethoscope‐Analysis Software System to Detect Canine Heart Murmurs	C39	
Sukjung Lim	Clinical and Imaging Characteristics of Patent Ductus Arteriosus in Standard Poodles and Their Cross‐breeds	C40	
Kendra Zelachowski	Cardiac Abnormalities Using a Simplified Diagnostic Evaluation in Dogs at Risk for Trypanosoma Cruzi Infection	C41	x
Bruce Keene	Evaluation of Trazodone on Heart Rate, Heart Rate Variability and QT‐intervals in Dogs	C42	
Samantha Scott	Small Coronary Arterial Size Is Associated with Dilated Cardiomyopathy in Doberman Pinschers	C43	
Catherine Georges	Circulating Surfactant Protein‐B in Healthy, Stage B2 and Acute Congestive Heart Failure (Stage C) Dogs	C44	x
Alba Stavri	Echocardiographic Evaluation of Pulmonary Vascular Resistance in Dogs with Pulmonary Hypertension	C45	x
Jeongmin Lee	The Prevalence of Mitral Valve Cleft in Dogs with Myxomatous Mitral Valve Disease	C46	
Jacqueline Sankisov	Two‐dimensional Echocardiographic Ratios for Assessment of Right Heart Size in Dogs	C47	
Sydney St. Clair	Effects of Medetomidine‐Vatinoxan on Echocardiographic Examination in Ventricular Tachy‐paced Dogs with Mild Heart Failure	C48	
Grace Flynn	Prevalence of Cardiac Disease and Population Characterization of Feline Telemedicine Cardiology Case Submissions	C49	
**EQUINE**			
**Presenting Author**	**Abstract Title**	**Sequence Number**	**Award Eligible**
Francesca Freccero	Tissue Doppler Imaging Assessment of the Left Ventricle in Healthy Standardbred Newborn Foals	E29	
Erica Jacquay	Impact of Short Distance Transportation on Horses with and Without Pars Pituitary Intermedia Dysfunction (PPID).	E30	
Ahmed Al Ansar	Fecal Microbiota and Serum Metabolome Association with Equine Metabolic Syndrome in Connemara Ponies	E31	
Claire Dixon	Developing clinical reasoning in veterinary students assessing equine colic – barriers and positive teaching strategies	E32	
Ashley Boyle	The Effects of Streptococcus Equi Equi Status on the Upper Respiratory Bacterial Microbiota of Horses	E33	
Andrew Waller	Unsaddling Streptococcus Equi: European Experiences with a New Fusion Protein Vaccine Against Strangles	E34	
Andrew Waller	Safety of Strangvac, a Vaccine Against Streptococcus Equi, in European Horses	E35	
Andrew Waller	Evolution of S. Equi in the UK: A Torrid Tale of Population Replacement by Strangulation	E36	
Morgan Askins	Effect of Changes in Pasture Water Soluble Carbohydrates in Horses with and Without Insulin Dysregulation	E37	
Bethanie Cooper	Investigating the impact of an algae‐derived DHA respiratory supplement on equine lower airway parameters	E38	
Nicolas Galinelli	Search for Biomarkers of Muscle Atrophy in Horses and Ponies with Pituitary Pars Intermedia Dysfunction	E39	
Rhonda Hoffman	Feeding Grain Before Thyrotropin Releasing Hormone Stimulation Did Not Affect ACTH Concentration in Horses	E40	
Marine Rullier	Lymphocytes Characterization in the Duodenal and Rectal Mucosal of Healthy Horses Exposed to Three Diets	E41	
Kathleen Mullen	Characterization of Concomitant Common Variable Immunodeficiency and Cutaneous or Pharyngeal Lymphoma in Horses	E42	
Csenge Tolnai	Long‐term Humoral Immune Protection Following West Nile Virus Infection in Horses	E44	
Camilo Jaramillo‐Morales	Serum parathyroid hormone, serum calcium and urinary fractional excretions in horses with nutritional secondary hyperparathyroidism	E45	
Kallie Hobbs	Evaluation of Endothelial Glycocalyx Shedding in Horses with Small Intestinal Disease	E46	
Kate Hepworth‐Warren	Changes in Peritoneal Fluid Associated with Colitis in Adult Horses	E48	
Clemence Loublier	Fecal Microbiota Dynamics Throughout Hospitalization in Horses with Different Types of Colic.	E49	
Dagmar Trachsel	Preliminary Assessment of the Leukocyte Coping Capacity as Stress‐Marker in Horses with Various Diseases	E51	
Kate Hepworth‐Warren	Delayed, Severe Rhabdomyolysis Following Uncomplicated Anesthesia in Six Warmblood Horses	E52	
Claire O'Brien	Physical Restraint for Veterinary Procedures – Efficacy and Welfare Effects on Horses	E54	
Sharanne Raidal	Tolerance of Binasal Prongs for Delivery of Non‐invasive Ventilation to Foals	E55	
David Byrne	Three‐dimensional Thoracic Electrical Impedance Tomography of Horses During Normal and Increased Tidal Volumes	E56	
**FOOD ANIMAL**			
**Presenting Author**	**Abstract Title**	**Sequence Number**	**Award Eligible**
Véronique Bernier Gosselin	Antimicrobial Resistance of Fecal Escherichia Coli from Calves Fed Milk Containing Antimicrobial Residues	F05	
Ryan Flynn	Evaluating the preliminary bias of a human point of care lactate meter in farm animals	F06	
Jessie Ziegler	Pharmacokinetics of Single and Multidose Oral Gabapentin in Goats	F07	
Christian Gerspach	The Intestinal Microbiome of Cows with Hemorrhagic Bowel Syndrome Compared to Healthy Cows.	F08	
Diego Gomez	The Fecal Microbiota of Calves Naturally Infected with Cryptosporidium spp	F09	
Luis Felipe Barbosa Braga Feitoza	Evaluation of Strategic Chute‐side Point‐of‐Care Ultrasonography Viability for Identification of Interstitial Pneumonia	F10	
**NEUROLOGY**			
**Presenting Author**	**Abstract Title**	**Sequence Number**	**Award Eligible**
Joonghyun Song	S100B as a Potential Biomarker of Non‐infectious Inflammatory Central Nervous System Diseases in Dogs	N13	
Croix Griffin	Preliminary Investigation of Paraspinal Musculature Magnetic Resonance Imaging (MRI) Characteristics in Canine Degenerative Myelopathy	N14	x
John Macri	Cerebrospinal Fluid Concentrations of Calcitonin Gene Related Peptide in Dogs with Chiari‐like Malformation	N15	x
Stephanie Marzullo	Recovery of Ambulation in Dogs with T3‐L3 and L4‐S3 Myelopathies Following Hemilaminectomy for Acute IVDE	N16	x
Savannah Giannasi	Clinical Outcome and Side Effects of Procarbazine in 67 Dogs with Presumptive Meningoencephalitis	N17	x
Bruno Torres	Assessment of Recommended Cerebrospinal Fluid Volume Collection in Dogs and Cats	N18	
Aryanne Ottoboni	Accuracy of Urinary Dipsticks for Glucose and Protein Determination in Cerebrospinal Fluid of Dogs	N19	
Bruno Torres	Epidemiological Characteristics and Risk Factors Associated with Neurological Manifestation of Canine Distemper Virus	NE01	
Woo‐Jin Song	Diagnosis and Management of Movement Disorder (paroxysmal Dyskinesia) in Small‐breed Dogs	NE02	
**NUTRITION**			
**Presenting Author**	**Abstract Title**	**Sequence Number**	**Award Eligible**
Maria Peralta	Evaluation of a Commercially Available Water Supplement to Promote Hydration in Clinically Ill Cats	NM01	
Kyle German	Evaluating Copper and Zinc Solubility in Canine Diets Using an in Vitro Model	NM02	
Ana Rita Pereira	Prevalence and Risk Factors of Feline Obesity in Client‐Owned Cats in Goiânia, Brazil	NM06	
**ONCOLOGY**			
**Presenting Author**	**Abstract Title**	**Sequence Number**	**Award Eligible**
Ga‐Hyun Lim	Assessment of the Impact of TSG6 on Chemoresistance Using Canine Mammary Gland Tumor Spheroids	O09	
gukil Joung	Evaluating the Effectiveness of Electrochemotherapy as an Adjuvant Therapy for Oral Malignant Tumors Following Surgery	O10	
Hanah Go	Holmium Laser‐Assisted Biopsy and Palliative Therapy for Bladder Masses in Dogs and a Cat	O11	
Hyeona Bae	Identifying Functional Roles and Pathways of Shared Mutations in Canine Solid Tumors	O12	
SoYoung Park	Assessing Prognostic Significance of Neutrophil to Lymphocyte Ratios in Canine Multicentric Lymphoma in Small‐Breed Dogs	O13	
Chiao‐Hsu Ke	Increased Disease‐free Survival in Dogs with Oral Malignant Melanoma Receiving CEA‐CAR‐NK‐92 MI Cell Therapies	O14	
**SMALL ANIMAL INTERNAL MEDICINE**–**ENDOCRINOLOGY**			
**Presenting Author**	**Abstract Title**	**Sequence Number**	**Award Eligible**
Jeong‐Ho Ha	Utilization of glycated Hemoglobin as a Biomarker to Identify and Monitor High‐risk Groups for Diabetes	EN09	
Jayeon Park	Evaluation of Serum microRNA‐375 Concentration in Dogs with Diabetes Mellitus	EN10	
Yeon Chae	Evaluating Efficacy Through Individualized Dosing Adjustment of Modified Radioiodine Therapy in Feline Hyperthyroidism	EN11	
Carly Patterson	Assessing a Third‐generation Flash Glucose Monitoring System in Nondiabetic Dogs with Rapidly Induced Hypoglycemia	EN12	
Taesik Yun	Efficiency and Safety Evaluation of Thyroid Scintigraphy Using Small‐Field‐of‐View Gamma Camera in Normal Cats	EN13	
**SMALL ANIMAL INTERNAL MEDICINE**–**GASTROENTEROLOGY**			
**Presenting Author**	**Abstract Title**	**Sequence Number**	**Award Eligible**
Laure Poincelot	Efficacy of a Flavored Oral Suspension of Metronidazole Against Giardia Duodenalis in Naturally Infected Dogs	GI22	
Laure Poincelot	Safety Evaluation of a Flavored Oral Suspension of Metronidazole in Dogs	GI23	
Hasegawa Nene	Prospective Evaluation of the Prevalence of Thromboembolism in Dogs with Inflammatory Protein‐losing Enteropathy	GI24	
Albert Jergens	Investigation of Different Leukocyte Ratios as Diagnostic Markers in Cats with Chronic Enteropathy	GI25	
Dong‐In Jung	A Study of Real‐Time Video Capsule Endoscopy for Diagnosing Acute Vomiting in Dogs	GI26	
Chand Khanna	Pilot Study Evaluating Fecal Microbiota and Clinical Response to Novel Probiotic in Dogs with Diarrhea	GI27	
Ana Rita Pereira	Comparison of Clinicalpathological Features Between Healthy Yorkshire Terriers and Those with Portosystemic Shunt Throughout Treatment	GI28	
Ana Rita Pereira	Diagnosis and Therapeutic Response of Chronic Colitis in French Bulldogs	GI29	
**SMALL ANIMAL INTERNAL MEDICINE**–**HEMATOLOGY**			
**Presenting Author**	**Abstract Title**	**Sequence Number**	**Award Eligible**
Eric Morissette	Diagnostic Assessment of Point‐of‐care Scanning System Integrated with Deep‐learning Algorithms for Canine/feline Blood Film Evaluation.	HM03	
DoHyeon Yu	The in Vitro Effects of Acidemia and Acidemia Reversal on Coagulation in Dogs	HM05	
Alejandra Príncipe Martínez	Evaluation of Circumferential Securing Tape Around Blood Pressure Cuffs on Doppler Ultrasound Blood Pressure Measurements	HM06	x
**SMALL ANIMAL INTERNAL MEDICINE**–**HEPATOLOGY**			
**Presenting Author**	**Abstract Title**	**Sequence Number**	**Award Eligible**
Jiseong Woo	Evaluation of Gallbladder Motility and Cholestasis by Hepatobiliary Scintigraphy in Dogs with Sludge	HP07	
Tarini Ullal	Evaluation of Serum Immunoglobulin G in Dogs Diagnosed and Treated for Immune‐mediated Chronic Hepatitis	HP08	
Tarini Ullal	Evaluation of Coagulation Parameters in Dogs with Immune‐mediated Chronic Hepatitis and Copper‐associated Chronic Hepatitis	HP09	
Tarini Ullal	Urinary copper: zinc Ratios in Dogs with Immune‐mediated Chronic Hepatitis and Copper‐associated Hepatitis	HP10	
**SMALL ANIMAL INTERNAL MEDICINE**–**IMMUNOLOGY**			
**Presenting Author**	**Abstract Title**	**Sequence Number**	**Award Eligible**
Rhonda LaFleur	Canine Parainfluenza One Year Duration of Immunity of a Combination BbPi Oral Vaccine Following Challenge	IM04	
Selena Tavener	Up‐regulation of CCR5 and CCL5 May Contribute to Chronic Low‐grade Inflammation in Aging Dogs	IM05	
Selena Tavener	Inhibition of Multiple Signaling Pathways May Attenuate mTOR and Promote Healthy Aging in Older Dogs	IM06	
Jeongmin Lee	Use of Eltrombopag‐Based Combination Treatment for Immune‐Mediated Thrombocytopenia in Dogs	IM07	
**SMALL ANIMAL INTERNAL MEDICINE**–**INFECTIOUS DISEASE**			
**Presenting Author**	**Abstract Title**	**Sequence Number**	**Award Eligible**
Anne Hoenl	Coexpression of Complement‐Regulating Factors and FCoV‐Antigen in Feline Infectious Peritonitis	ID06	
Amanda Gimenez	Fecal Microbial Transplant for Parvovirus in the Outpatient Setting Interim Analysis: A Randomized Controlled Trial	ID07	
Jimin Park	Molecular Investigation of Tick‐borne Pathogens from 6141 Dogs and 682 Cats in South Korea	ID08	
SoYeon Park	Molecular Prevalence of Upper Respiratory Tract Infection Pathogens in Dogs and Cats in South Korea	ID09	
Tomoki Motegi	Assessing Antimicrobial Resistance in Companion Animals at Referral Hospital: The Impact of Antimicrobial Stewardship Strategies	ID10	
Lisa Kim	Period‐prevalence and Distribution of Babesia species Exposure in Thrombocytopenic Dogs in the Upper Midwest	ID11	
Erin Lashnits	Successful In‐hospital Treatment of FIP with FDA‐approved Remdesivir (Veklury®) in 5 Cats	ID12	
**SMALL ANIMAL INTERNAL MEDICINE**–**NEPHROLOGY/UROLOGY**			
**Presenting Author**	**Abstract Title**	**Sequence Number**	**Award Eligible**
Lauren Reynolds	Vitamin D Metabolite Profiles in Cats with Chronic Kidney Disease Compared to Healthy Cats	NU16	x
Rene Paschall	The Effect of Porus® One on Uremic Toxin Concentrations In Cats with Chronic Kidney Disease	NU17	x
Amanda Alli	Evaluation of the Effect of Bilirubinuria on Urine Dipstick Results	NU18	
Amanda Blake	Analytical Validation of an Assay for Measurement of Uremic Toxins in Dog and Cat Serum	NU19	
Claire Cassou	Characterization of Cardiac Disease in Cats with Ureteral Obstruction Undergoing subcutaneous Ureteral Bypass Device Placement	NU20	
Zachary George	Comparing Defecation Frequency Between Cats with and Without Chronic Kidney Disease	NU21	
Anna Panyutin	The Effect of Feeding on Urine Ammonia Levels in Cats with and Without CKD	NU22	x
Anonda Haskin	Comparison of Urine Sample Preparation Methods in Recovering Urine Sediment Elements from Canine/Feline Samples	NU23	
Katelyn McFadden	Pathophysiology of Hyperammonemia from Acute Kidney Injury or Acute on Chronic Kidney Disease in Cats	NU24	x
Cory Penn	Deep Learning Artificial Intelligence (AI) for Rapid and Reliable Evaluation of Canine/Feline Urine Sediment Samples	NU25	
Jessica Quimby	Quantification of Serum Leptin Concentration in Cats with and without Chronic Kidney Disease	NU26	
Kayla Dunn	Effects of Urine Biochemical Parameters in Use of Preservative Collection Tubes and Preservative Free Tubes	NU27	
Lina Lim	Feline Chronic Kidney Disease Is Associated with Significant Caregiver Burden	NU28	
Amber Carson	Evaluation of a Salivary Urea Nitrogen Test in Cats with Lower Urinary Tract Outflow Obstructions.	NU29	
Elayna Anderson	Potential for Extending the Chloramphenicol Dosing Interval for Canine Urinary Tract Infections	NU30	
Minju Baek	Retrospective Evaluation of Risk Factors for Kidney Injury After Angiotensin‐converting Enzyme Inhibitor Treatment in Dogs	NU31	x
Leah Ramsaran	Episioplasty Reduces the Incidence of Urinary Tract Infections and Perivulvar Pyoderma in Dogs	NU32	
Jamie Hart	Retrospective Evaluation of Hypoalbuminemia in Proteinuric Dogs	NU33	
Rene Paschall	The Effect of Porus® One on Amino Acid Concentrations In Cats with Chronic Kidney Disease	NU34	x
**SMALL ANIMAL INTERNAL MEDICINE**–**PHARMACOLOGY**			
**Presenting Author**	**Abstract Title**	**Sequence Number**	**Award Eligible**
David Griffin	Investigating the Pharmacokinetics and Pharmacodynamics of Glucoraphanin: A Crossover Study in Healthy Cats	OH09	
Amanda Garrick	Bioavailability of Oral Ondansetron in Dogs, a Cross‐over Study	PH04	x
Chaeyoon Im	Pirfenidone Inhibits TGF‐β1‐Induced Fibrosis via Downregulation of Smad and ERK Pathway in MDCK Cells	PH05	
Zhe (Alice) Wang	The Pharmacokinetics of Ampicillin Sulbactam in Azotemic and Non‐Azotemic Dogs	PH06	x

## ABSTRACT C01: Hemodynamic, echocardiographic, electrocardiographic, and behavioral effects of oral gabapentin in healthy dogs

### **Elise Lenske** ^1^; Allison Gagnon^2^, DVM, MS, DACVIM (Cardiology); Lauren Nakonechny^3^, DVM; Machelle Wilson^4^, PhD; Melissa Bain^5^, DVM, DACVB, MS, DACAW

#### ^1^University of California‐Davis, Davis, CA, USA; ^2^Assistant Professor of Cardiology, Medicine & Epidemiology, University of California‐Davis, Davis, CA, USA; ^3^Cardiology Resident, University of California‐Davis, Davis, CA, USA; ^4^Principal Statistician, Department of Public Health Sciences, University of California‐Davis, Davis, CA, USA; ^5^Professor of Clinical Animal Behavior, University of California‐Davis, Davis, CA, USA

**Background:** Gabapentin has been used prior to veterinary visits in dogs to reduce signs of anxiety; however, its effects on cardiac diagnostics have not been evaluated in dogs.

**Hypothesis/objectives:** To investigate the effects of gabapentin on blood pressure, echocardiography, electrocardiography, and behavior scores in healthy dogs.

**Animals:** 15 healthy, client‐owned dogs aged 1‐8 years.

**Methods:** Dogs were enrolled in a prospective, double‐blinded, placebo‐controlled, crossover study. They were randomized to receive placebo or gabapentin (30‐31.5 mg/kg) orally 90 minutes prior to visit. Physical examination, Doppler blood pressure, echocardiogram, electrocardiogram, and 24‐hour Holter placement were performed. After a minimum 7‐day washout period, diagnostics were repeated with the alternate treatment. Anxiety was assessed by blinded review of video obtained during visits. Results were described with mean ± SD, and linear mixed effects models were performed.

**Results:** Left atrium to aorta ratio measured in long axis was smaller following gabapentin (2.11 ± 0.14 vs. 2.19 ± 0.17, *P* = .017), but the difference was considered clinically unimportant. Normalized left ventricular internal dimension in systole and diastole, ejection fraction, blood pressure, corrected QT interval, 24‐hour average heart rate, and number of ventricular premature complexes were not statistically significantly different. Video scores assessing stress following gabapentin were not significantly different from placebo (2.16 ± 0.50 vs. 2.35 ± 0.67, *P* = .16). Adverse effects reported with gabapentin were sleepiness (3/15), incoordination (1/15), and urinary incontinence (1/15).

**Conclusions and Clinical Importance:** A single, oral, pre‐visit dose of gabapentin was well‐tolerated and caused a clinically unimportant reduction in left atrial to aortic ratio without significant effects on other parameters.

## ABSTRACT C02: Correlation of echocardiographic and angiographic measurements of pulmonary annular diameter in dogs with pulmonary stenosis

### **Kara L. Maneval** ^1^; Randolph Winter^1^, DVM, PhD, DACVIM (Cardiology); SeungWoo Jung^2^, DVM, MS, PhD, DACVIM (Cardiology), DAiCVIM (Cardiology)

#### ^1^Auburn University, Auburn, AL, USA; ^2^Echo Vet Cardio, Tustin, CA, USA

**Background:** Pulmonary valve annular (PVA) diameter is assessed with angiography (PVA‐Ang) and transthoracic echocardiography (PVA‐TTE), and both may impact procedural planning for balloon valvuloplasty in dogs with pulmonary stenosis (PS).

**Hypothesis/Objectives:** The objective of this study was to describe the relationship between PVA‐Ang and PVA‐TTE in dogs with PS. We hypothesized that echocardiographic image quality would impact level of agreement and that PVA‐Ang would be greater than PVA‐TTE in most dogs.

**Animals:** Observational, retrospective study of 93 client‐owned dogs.

**Methods:** Medical records of dogs with PS were reviewed. PVA diameter was measured on both angiographic (PVA‐Ang) and transthoracic echocardiographic (PVA‐TTE) images. Image quality scores were assigned to echocardiographic images of the PVA based on visualization of PVA margins and valve leaflet hinge points. Agreement between image modalities was assessed by Bland–Altman analysis, and an ANOVA or Kruskal‐Wallis test was used for between‐group comparisons. A *P* value <.05 was considered significant.

**Results:** In 70% of dogs, the PVA‐Ang was larger than the PVA‐TTE. The median difference between measurements was 1.6 mm (range 0.0‐8.4 mm). Dogs with poor echocardiographic image quality had greater differences (*P* < .001) between PVA‐Ang and PVA‐TTE (median 2.7 mm, range 1.35‐8.4 mm) compared to those with excellent image quality (median 1.2 mm, range 0.1‐3.8 mm).

**Conclusions and Clinical Importance:** Diameters of PVA‐Ang were greater than PVA‐TTE in most dogs, which was most apparent with worse echocardiographic image quality. These differences may be clinically relevant to interventional procedure planning.

## ABSTRACT C03: Multicentre per‐catheter ductal occlusion in small dogs; Device selection, patient characteristics, outcomes, and complication rates

### **Joe Herbert** ^1^; Christopher Stuathammer^2^, DVM DACVIM (Cardiology); Allison Masters^3^, DVM, MPH, DACVIM; Omri Belachsen^4^, DVM PgCertVPS CertAVP(VC) DECVIM‐CA (Cardiology) MRCVS; Matthias Schneider^5^; Alexis Santana Gonzalez, LV. MCs. GPCert (Cardio); Fabio Sarcinella^6^; Chris Linney^7^, BVSc MSc CertAVP(VC) DECVIM CA (Cardiology) MRCVS; Mathias Schneider^8^; Matthew Aherne, MVB (Hons 1), GradDipVetStud, MS, MANZCVS (Small Animal Surgery), DACVIM (Cardiology); Marissa Cicozzi^1^, DVM; Emily Gavic^1^, DVM; Daniel Whu; Tobias Wagner^9^, DECVIM‐CA (Cardiology) Dr.med.vet. MRCVS

#### ^1^University of Minnesota, Minneapolis, MN, USA; ^2^Professor, Cardiology, University of Minnesota, Minneapolis, MN, USA; ^3^Assistant Professor, Cardiology, University of Minnesota, Minneapolis, MN, USA; ^4^Southern Counties Veterinary Specialists, Hangersley, Ringwood, UK; ^5^Professor, Justus‐Liebig‐University Gießen, Gießen, Germany; ^6^Head of Cardiology, Cardiology, Willows Veterinary Centre and Referral Service, Solihull, UK; ^7^Head of Cardiology, Cardiology, Paragon Veterinary Referrals, Wakefield, UK; ^8^Universitat Giessen, Giessen, Germany; ^9^Clinical Director

**Background:** Per‐catheter occlusion of patent ductus arteriosus (PDA) in dogs ≤3.5 kg is challenging, often requiring ligation via thoracotomy with higher complication rates. A number of occlusive devices have been individually described in small dogs for ductal occlusion.

**OBJECTIVE:** Present the results and complications of ductal occlusion in small dogs with AVPII, AVPIV, Flipper Coils, Vet‐PDA occluder, and ACDO devices.

**Animals:** 213 dogs (0.7‐3.5 kg) underwent interventional occlusion of a PDA. Methods: Multi‐institutional retrospective analysis of outcomes and complications in <3.5 kg dogs receiving ACDO, AVPII, AVPIV, Vet‐PDA occluder, and Flipper coils. Ductal morphology and dimensions, procedural outcomes, complication rates, and residual flow were evaluated at 24 hr and between 2 and 4 weeks post‐implantation. Procedural success was defined as successful device placement within the ductus.

**Results:** Successful occlusion was achieved in 206 (96.71%). Four (2.52%) peri‐operative deaths occurred and one postoperative mortality occurred (1.26%). Three (1.41%) additional cases were unsuccessful in device placement. At 2‐4 week recheck, 81.69% showed no residual flow, 2.35% trivial, 4.69% mild, 1.41% moderate, 0.94% severe 6.10% lost to follow up and 2.82% other. Major complications occurred in 23 (10.8%) patients, including death, severe pulmonary hypertension, hemorrhage, right atrial perforation, asystole when wire passed into the right ventricle, caval perforation, hemorrhagic pleural effusion of unknown etiology, and device embolization.

**Conclusions and Clinical Importance:** Per‐catheter ductal occlusion in small dogs <3.5 kg may be safely achieved. Our study reveals comparable outcomes regardless of device or patient weight, refining intervention recommendations for PDA occlusion in small dogs.

## ABSTRACT C04: Effect of orientation and additional planes in the echocardiographic determination of three‐dimensional left ventricular volume

### **Cortney E. Pelzek** ^1^; Samantha Siess^2^, PhD; Benjamin Terhaar^3^; Shana Mintz^4^, DVM, DACVIM (Cardiology); Weihow Hsue^4^, DVM, DACVIM (Cardiology)

#### ^1^Cornell University, Ithaca, NY, USA; ^2^Veterinary Student, Cornell University, Ithaca, NY, USA; ^3^Undergraduate Student, Cornell University, Ithaca, NY, USA; ^4^Assistant Professor, Clinical Sciences, Cornell University, Ithaca, NY, USA

**Background:** Deriving mitral regurgitant fraction relies on accurate determination of left ventricular (LV) volumes. Many linear and planar echocardiographic methods, in different orientations and number of planes, are clinically practical, but have not been comprehensively evaluated against real‐time three‐dimensional echocardiographic volume (3DV).

**Hypothesis/Objectives:** Identify the LV volumetric approaches that generate the best agreement and interoperative reproducibility compared to 3DV across varying disease severities.

**Animals:** Sixty client‐owned dogs with myxomatous mitral valve disease (38 Stage B1, 13 Stage B2, 9 Stages C/D) received echocardiograms, with a subset of 29 dogs imaged by two operators.

**Methods:** Prospective method comparison study. End‐diastolic and end‐systolic LV volumes calculated via linear methods in long‐ and short‐axis (Teichholz, cubed, modified cubed), monoplane methods in right parasternal and left apical views (area‐length and Simpson's method of disc), biplane Simpson's method of disc, and real‐time triplane were compared against 3DV using Bland‐Altman analysis and concordance correlation coefficients. Interoperator reproducibility was assessed via intraclass correlation coefficients and reproducibility coefficients.

**Results:** The linear methods overall had substantial bias and poor agreement compared to 3DV (Table 1). Among the monoplane methods, the right parasternal view provided better agreement and interoperator reproducibility, particularly in systole (Table 2), than the left apical view. Among the multiplane methods, only triplane offered improved agreement above the monoplane methods, although its interoperator reproducibility in systole was worse. Interoperator reproducibility was poor with 3DV.

**Conclusions and Clinical Importance:** No single method appeared consistently superior. The results of this study help delineate the limitations of each method.

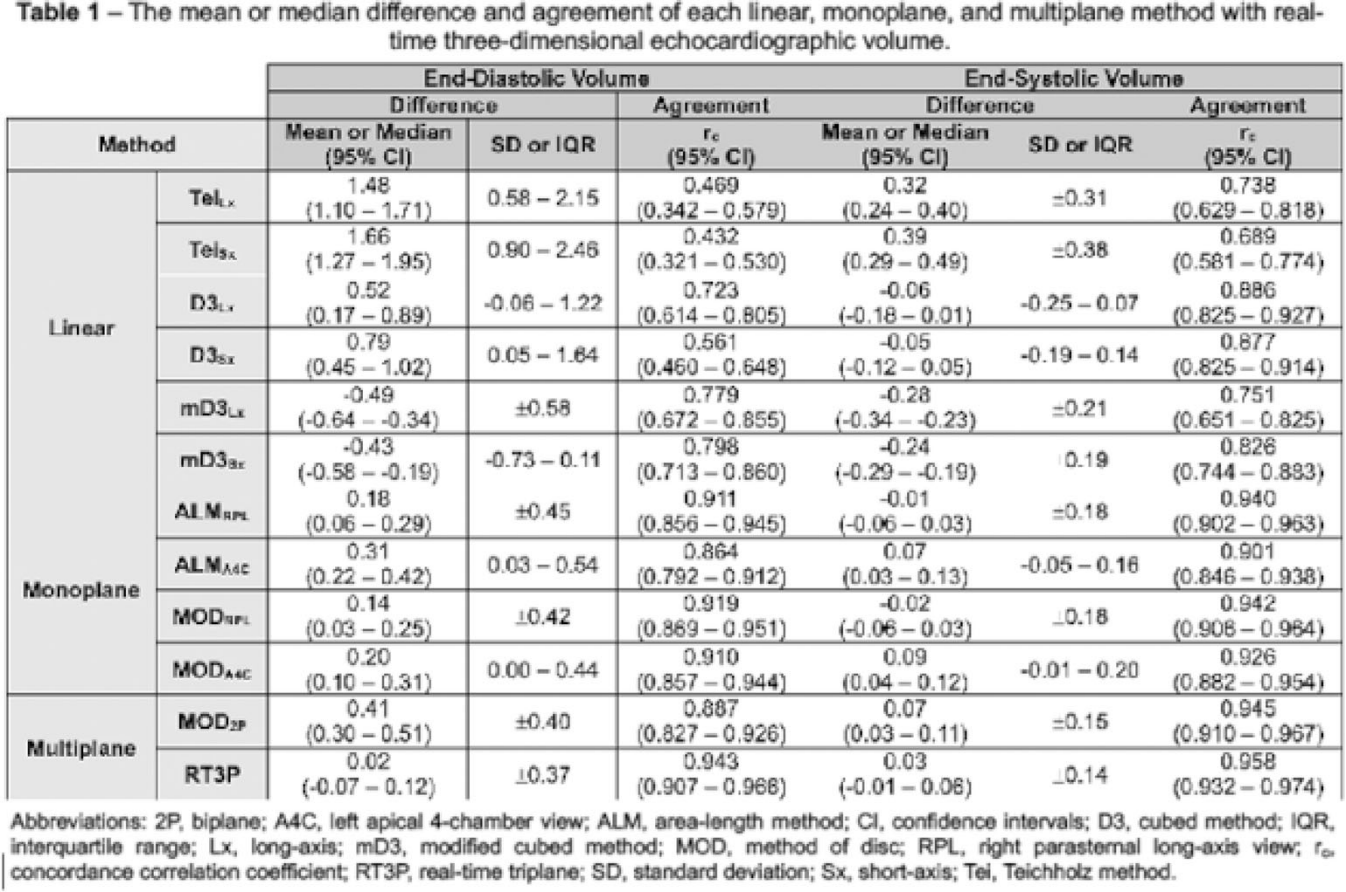

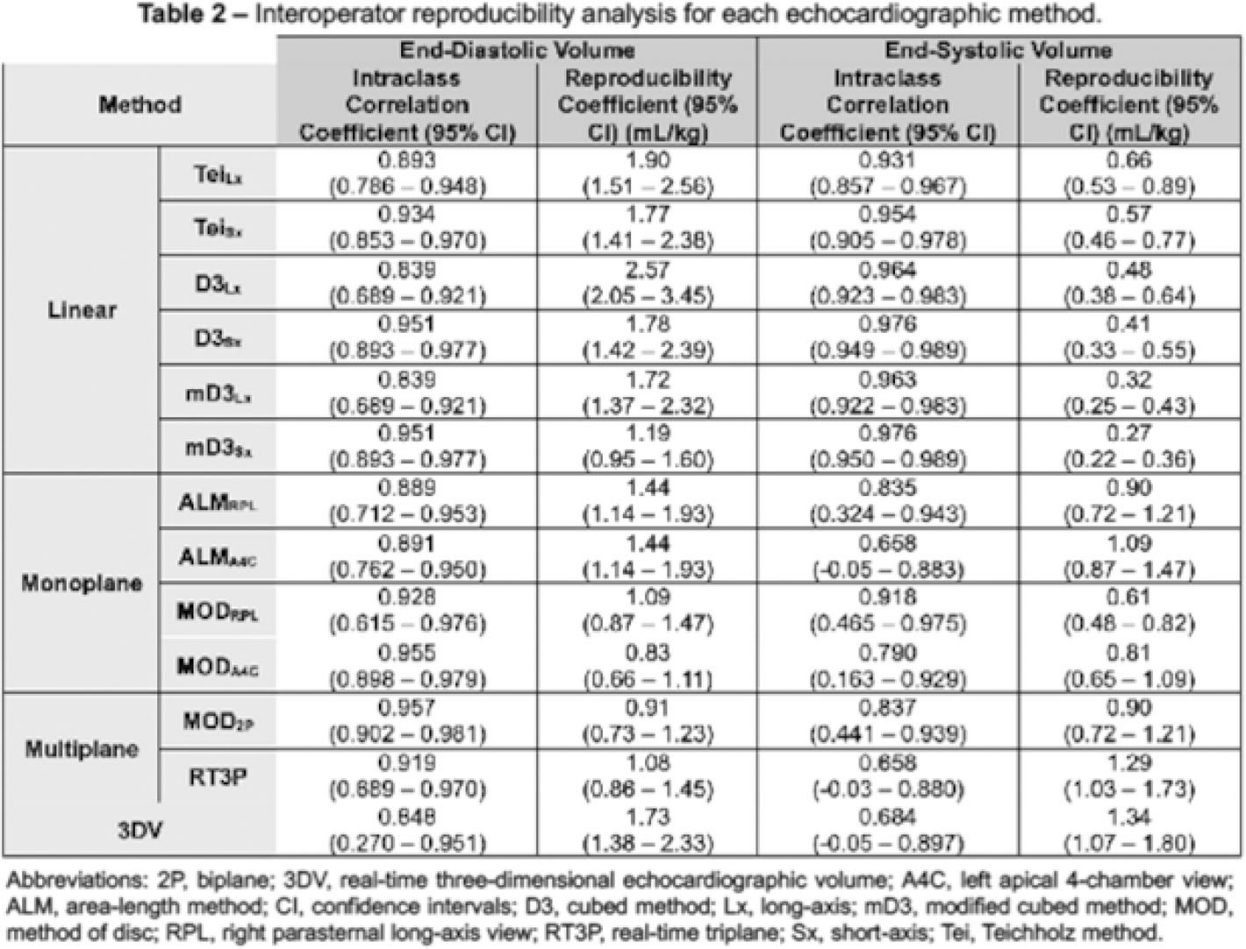

## ABSTRACT C05: Feasibility, efficiency, and reproducibility of left ventricular volume estimation by two‐dimensional and three‐dimensional echocardiography

### Sumana Prabhakar

#### University of Illinois, Champaign, IL, USA

**Background:** In healthy dogs, transthoracic three‐dimensional echocardiography (3DE) compares more favorably with reference standard imaging and has higher reproducibility than transthoracic two‐dimensional echocardiography (2DE). In dogs with cardiac disease, comparison of feasibility, efficiency, and reproducibility of left ventricular (LV) volume assessment by 3DE and 2DE has not been evaluated.

**Hypothesis:** Both methods will be feasible and 3DE will be more efficient and reproducible than 2DE.

**Animals:** Fifty‐three dogs with cardiac disease from a university hospital.

**Methods:** Prospectively, all dogs underwent 2DE and 3DE examinations by three observers and 12 dogs had a second study performed on the same day. Feasibility, acquisition, and analysis time were compared using Bland‐Altman and Wilcoxon signed rank test. Inter‐ and intraobserver variability was quantified using coefficients of variation (CV).

**Results:** Feasibility of 2DE and 3DE were 95% and 98% respectively. Total efficiency was 45.7% faster for 3DE (*P* < .001). Compared with 3DE, 2DE overestimates stroke volume (SV) (+3.3 mL, *P* < .001) and ejection fraction (EF) (+6.1%, *P* < .001) and underestimates LV end‐systolic volume (−3.5 mL, *P* < .001). 3DE had lower or equivalent reproducibility than 2DE for interobserver CV (end‐diastolic volume 6.4% vs. 8.7%; end‐systolic volume 12.2% vs. 14.1%; SV 15.3% vs. 15.7%, EF 10.4% vs. 10.4%) and intraobserver CV (end‐diastolic volume 13.6% vs. 21%; end‐systolic volume 14.8% vs. 29.8%; SV 16.2% vs. 25.2%; EF 13.4% vs. 14.6%).

**Conclusions and Clinical Importance:** In dogs with cardiac disease, 3DE assessment of LV volume is feasible, and more efficient and reproducible than 2DE.

## ABSTRACT C06: Effects of oral single‐dose empagliflozin on urine glucose and serum β‐hydroxybutyrate in healthy adult dogs

### **Andrea Plá**; Etienne Côté, DVM, DACVIM (Cardiology, SAIM); Sandra McConkey, DVM, PhD, DACVP; M. Lynne O'Sullivan, DVM, PhD, DACVIM (Cardiology)

#### Atlantic Veterinary College, Charlottetown, PE, Canada

**Background:** Empagliflozin is a sodium‐glucose cotransporter‐2 (SGLT‐2) inhibitor used in the treatment of human heart failure patients. This drug could have applications in veterinary cardiology, but pharmacokinetic and pharmacodynamic information on empagliflozin in healthy dogs has not been published.

**Hypothesis/Objectives:** A single oral dose of empagliflozin at a target dosage of 0.3 mg/kg given to healthy dogs will result in significant changes in quantitative urine glucose and serum β‐hydroxybutyrate (BHBA) concentrations.

**Animals:** Six healthy, privately‐owned dogs.

**Methods:** Prospective study evaluating urinary glucose and serum BHBA concentrations at 0, 6, 12, 24, 48, and 72 hours after administration of 10 mg empagliflozin PO. Normally distributed data (Shapiro‐Wilk test) for urine glucose concentrations were analyzed using repeated measures one‐way ANOVA.

**Results:** Dosage administered was 0.28‐0.45 mg/kg. Serum BHBA concentrations were below the normal canine reference range (<200 umol/L) at all time points (Figure 1). Urine glucose concentrations showed significant (*P* < .0001) change throughout the study period (Figure 1) with peak excretion at 24 h with a complete return to baseline by 72 h.

**Conclusions and Clinical Importance:** Empagliflozin 0.28‐0.45 mg/kg PO produced measurable effects in urine glucose concentrations in healthy dogs and not in serum BHBA concentrations.

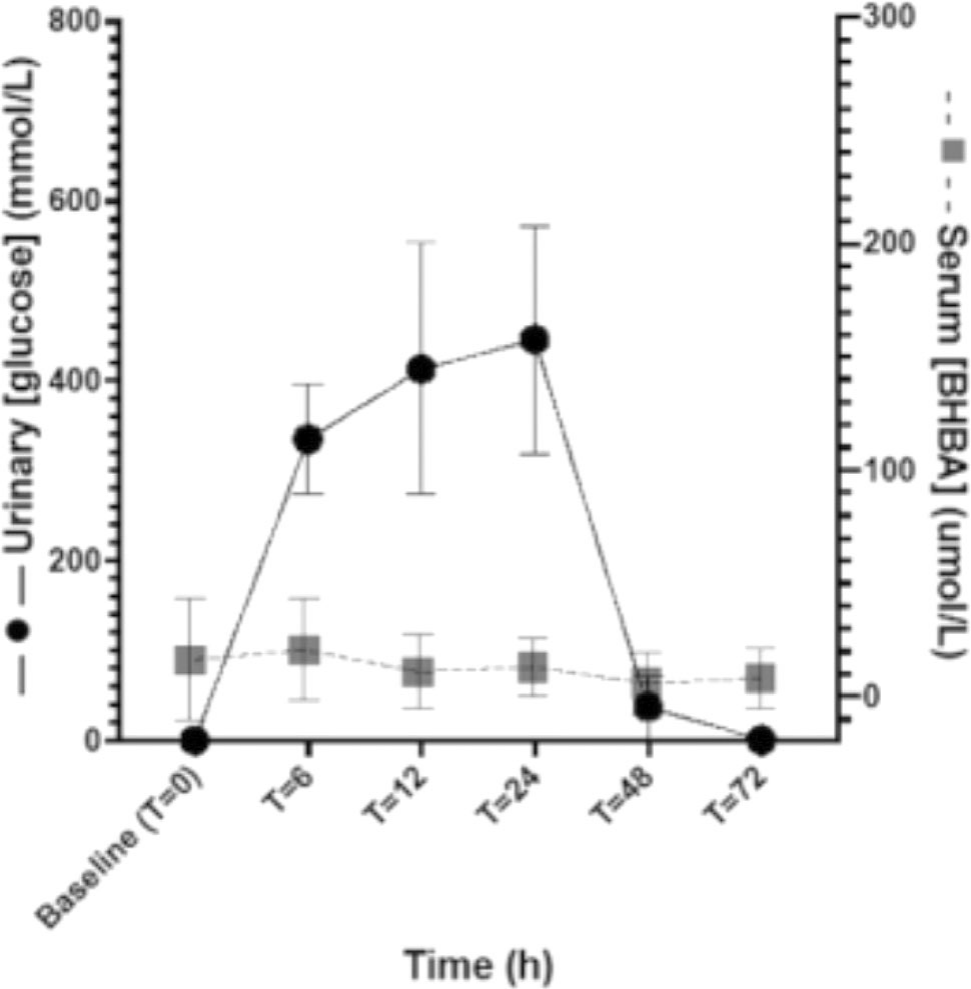

## ABSTRACT C07: Comparison of low‐ and high‐pressure balloon valvuloplasty in dogs with severe pulmonary valve stenosis

### **Brad Lytle** ^1^; Daniel Hogan^1^; Anna McManamey^1^ ; Matheus Mantovani^2^; Luis Dos Santos^1^

#### ^1^Purdue University, West Lafayette, IN, USA; ^2^Federal University of Uberlandia, Uberlândia, Brazil

**Background:** Balloon valvuloplasty (BVP) is standard treatment for severe valvular pulmonic stenosis (PS) in dogs. Efficacy and safety of low‐pressure balloon catheters (LP) and high‐pressure balloon catheters (HP) for BVP has not been compared.

**Hypothesis/Objectives:** There will be no difference in pressure gradient reduction between HP and LP. Cardiac troponin I (cTnI) will be higher with HP compared to LP immediately following BVP.

**Animals:** Twenty‐five (25) client‐owned dogs with severe PS.

**Methods:** Prospective, randomized study. Patients matched based on pulmonic annulus size. Echocardiographic measures of PS severity included transvalvular pressure gradient (PGmax) and aorta‐to‐pulmonary artery velocity time integral ratio (Ao/PAVTI) at baseline, 18‐24 hours following BVP and at 3‐month recheck. Serum cTnI was measured at each time point.

**Results:** There were 13 dogs in the LP group and 12 in the HP group with no difference in sex, weight, or age between groups. There was no significant difference in PS severity between groups at baseline [(Ao/PAVTI; *P* = .376), (PGmax; *P* = .406)], 18‐24 hours post‐BVP [(Ao/PAVTI; *P* = .270), (PGmax; *P* = 0.263)], or at 3‐month recheck [(Ao/PAVTI; *P* = .418), (PGmax; *P* = .184)]. There was no significant difference in serum cTnI between groups at baseline (*P* = .069), 18‐24 hours post‐BVP (*P* = .378), or at 3‐month recheck (*P* = .705). Major complications were rare with no difference between HP and LP.

**Conclusions and Clinical Importance:** There was no difference in reduction of PS severity between HP and LP. The use of HP is not expected to provide superior reduction in PS severity compared to LP or be associated with increased myocardial injury.

**Table:** Table 1

Variables	Group	Pre balloon	Post balloon	3 m after balloon	*P* value
PV_max_ (m/s)	HP	5.83 ± 1.26^a^	3.51 ± 0.78^b^	3.85 ± 0.82^b^	<.0001
	LP	5.31 ± 0.96^a^	3.15 ± 0.68^b^	3.52 ± 0.93^b^	.001
	*P* value	0.458	0.275	0.147	
PS PG (mm Hg)	HP	143.45 ± 61^a^	51.69 ± 22^b^	61.97 ± 27^b^	<.0001
	LP	116.50 ± 41^a^	41.55 ± 17^b^	52.68 ± 27^b^	<.0001
	*P* value	0.406	0.263	0.184	
PV mean PG (mm Hg)	HP	81.58 ± 33^a^	29.70 ± 15^b^	29.54 ± 18^b^	<.0001
	LP	59.54 ± 32^a^	26.25 ± 14^b^	24.74 ± 20^b^	<.0001
	*P* value	0.280	0.280	0.280	
VTIPV (cm)	HP	90.80 ± 31^a^	52.45 ± 22^b^	53.87 ± 20^b^	<.0001
	LP	86.07 ± 19^a^	44.81 ± 12^b^	55.47 ± 18^b^	<.0001
	*P* value	0.725	0.355	0.745	
Ao:PV diameter	HP	1.18 ± 0.15	1.25 ± 0.18	1.22 ± 0.16	.450
	LP	1.12 ± 0.23	1.15 ± 0.16	0.99 ± 0.39	.450
	*P* value	0.417	0.251	0.181	
VTIpv:VTIAo	HP	6.49 (4.98‐7.59)^a^	3.94 (2.90‐4.61)^b^	3.28 (2.73‐4.63)^b^	.001
	LP	6.54 (5.28‐8.78)^a^	2.68 (2.4‐4.11)^b^	3.67 (2.74‐5.58)^b^	<.0001
	*P* value	0.376	0.270	0.602	
PV_max_:Ao_max_	HP	4.92 ± 1.24^a^	3.4 ± 0.58^b^	3.31 ± 0.57^b^	.002
	LP	4.82 ± 1.72^a^	2.71 ± 0.55^b^	2.96 ± 0.83^b^	.008
	*P* value	0.855	0.055	0.364	
cTnI (ng/mL)	HP	0.14 (0.07‐0.35)^a^	2.3 (0.62‐5.6)^b^	0.13 (0.07‐0.19)^a^	.0004
	LP	0.04 (0.01‐0.13)^a^	0.85 (0.43‐4.5)^b^	0.10 (0.03‐0.23)^a^	<.0001
	*P* value	0.069	0.378	0.705	

Note: Different superscript letters on the same line indicate statistical differences by the Bonferroni test (*P* < 0.05).

## ABSTRACT C08: Association of the TTN and PDK4 gene variants with dilated cardiomyopathy in british doberman pinschers

### **Luke C. Dutton** ^1^; David Connolly^2^, BSc, BVetMed, PhD, CertVC, CertSAM, DECVIM (cardiology), MRCVS; Joanna Dukes‐McEwan^3^ , BVMS(Hons), MVM, PhD, DVC, DECVIM‐CA (Cardiology), FRCVS; Andrew Crosland^4^, DVM, BSc (Med Sci), Grad dip. Vet Stud

#### ^1^Royal Veterinary College, London, UK; ^2^Professor of Veterinary Cardiology, Clinical Science and Services, Royal Veterinary College, London, UK; ^3^Professor of Veterinary Cardiology, Small Animal Clinical Science, University of Liverpool, Liverpool, UK; ^4^Resident in Cardiology, Small Animal Teaching Hospital, University of Liverpool, Liverpool, UK

**Background:** A missense mutation in the titin gene (TTN) and a splice‐site mutation in the pyruvate dehydrogenase kinase 4 gene (PDK4) have been associated with dilated cardiomyopathy (DCM) in Doberman Pinchers from the USA.

**Hypothesis:** The previously reported TTN and PDK4 variants would be associated with DCM in a population of Doberman Pinchers from the United Kingdom.

**Animals:** 84 client‐owned dogs (40 control dogs and 44 dogs with DCM).

**Methods:** Allele frequencies for each variant were calculated. Chi‐square test used to assess for differences in genotype proportions between groups.

**Results:** Overall allele frequency in this cohort was 35% for the TTN variant and 18% for the PDK4 variant. Of the dogs with DCM, 19/44 (43%) were wild type (WT), 19/44 (43%) were heterozygous, and 6/44 (14%) were homozygous for the TTN variant. In the control dogs, 19/40 (47.5%) were wild type (WT), 15/40 (37.5%) were heterozygous, and 6/40 (15%) were homozygous for the TTN variant. When the PDK4 variant was sequenced, of the DCM dogs 33/44 (75%) were wild‐type (WT), 5/44 (11%) were heterozygous, and 6/44 (14%) were homozygous, and for the control dogs 31/40 (77.5%) were wild‐type (WT), 4/40 (10%) were heterozygous, and 5/40 (12.5%) were homozygous. There was no difference in the genotype frequencies for either the TTN variant (*P* = .87) or PDK4 variant (*P* = .96) between DCM dogs and control dogs.

**Conclusions and Clinical Importance:** Neither the previously reported TTN variant or PDK4 appear associated with DCM in a British population of Doberman Pinchers.

## ABSTRACT C09: Multicenter transvenous pacemaker implantation in 595 dogs: complications and survival rates

### **Marisa Ciccozzi** ^1^; Christopher Stauthammer^2^; Christopher Whipp^1^; Lance Visser^3^; Kelly Flynn^4^; Heidi Kellihan^4^; William Byun^5^; Robert Sanders^5^; Jake Ryave^5^; Marisa Ames^3^; Aaron Rendahl^1^

#### ^1^University of Minnesota, Minneapolis, MN, USA; ^2^Professor of Cardiology, University of Minnesota, Minneapolis, MN, USA; ^3^University of California‐Davis, Davis, CA, USA; ^4^University of Wisconsin, Madison, WI, USA; ^5^Michigan State University, East Lansing, MI, USA

**Background:** Transvenous pacemakers are routinely used for pathologic bradycardias including 3rd degree AV block (3AVB), Sick Sinus Syndrome (SSS), high grade 2nd degree AV block (2AVB), and atrial standstill (AS).

**OBJECTIVES:** This study reports the multi‐institutional complications and survival rates in a large cohort of dogs.

**Animals:** Dogs presenting for transvenous pacemaker implantation between January 2000 and December 2016 at four academic institutions.

**Methods:** A retrospective analysis of outcomes and complications in dogs who underwent transvenous pacemaker implantation.

**Results:** 595 dogs were identified (221 male; 374 female) with a median age of 9.6 years (range, 0.27‐17 years). Presenting arrhythmias included 3AVB (54.5%), SSS (24%), high grade 2AVB (16.1%), and AS (3.9%). Major complications occurred in 17% (105/595) of cases including lead dislodgement (n = 39), infection (n = 25), thrombus formation (n = 19), ventricular fibrillation (n = 8), cardiac arrest (n = 6), cardiac perforation (n = 5), and iatrogenic tricuspid stenosis (n = 3). Procedural mortality rate was 0.8% (5/595). The rate of ventricular lead dislodgement was 24% in AS, 8% in 3AVB, 2% in 2AVB, and 1% in SSS. The rate of dislodgement in active ventricular leads was 10% and 4% in passive leads. Overall median survival time was 38.7 months (2AVB, 43.9 months; 3AVB, 43.3 months; AS, 40.4 months; SSS, 34.8 months). Cardiac mortality occurred in 75% of AS compared to 2AVB (30%), 3AVB (27%), and SSS (19%).

**Conclusions:** There is a high procedural success rate with low procedural mortality rate. Overall, complications were minimal. Type of lead and underlying disease process may impact the risk of lead dislodgement.

## ABSTRACT C10: Aldosterone breakthrough during enalapril or telmisartan therapy in dogs with advanced myxomatous mitral valve disease

### **Christianna Ziccardi** ^1^; Amanda Coleman^2^; Amelia Sinkin^2^; Bianca Lourenço^2^

#### ^1^UGA Veterinary Teaching Hospital, Athens, GA, USA; ^2^University of Georgia, Athens, GA, USA

**Background:** Whether and to what extent aldosterone “breakthrough” (ABT)–the undesirable phenomenon in which aldosterone exceeds pre‐treatment levels despite blockade of the renin‐angiotensin‐aldosterone system ‐ occurs in dogs with naturally occurring myxomatous mitral valve disease (MMVD) treated with an angiotensin receptor blocker (ARB) has not been systematically evaluated.

**OBJECTIVES:** To compare the incidence of ABT in dogs with advanced MMVD treated with an angiotensin‐converting enzyme inhibitor (ACEi; enalapril) or an ARB (telmisartan).

**Animals:** Sixteen client‐owned dogs with advanced MMVD.

**Methods:** Prospective, randomized, masked clinical study. Serum equilibrium concentrations of aldosterone (measured using liquid chromatography‐mass spectrometry) and urinary aldosterone‐to‐creatinine ratio (UACR; aldosterone and creatinine measured using radioimmunoassay and modified Jaffe's method, respectively) were determined on samples collected before and after 30 days of treatment with enalapril (0.5 mg/kg PO BID) or telmisartan (1 mg/kg PO q24h). ABT was defined as any increase in serum aldosterone concentration or UACR compared to pre‐treatment baseline. The proportion of dogs experiencing ABT was compared between groups using the Fisher's exact test.

**Results:** ABT occurred in 4 (66.7%) of 6 enalapril and 3 (33.3%) of 9 telmisartan‐treated dogs based on serum aldosterone concentrations (*P* = .31), and 2 (33.3%) of 6 enalapril and 2 (22%) of 9 telmisartan‐treated dogs based on UACR (*P* = .99).

**Conclusions and Clinical Importance:** ABT occurs in dogs receiving an ACEi or an ARB. We did not demonstrate a difference in incidence between treatment groups, possibly due to type II error.

## ABSTRACT C11: Characterization of the tissue renin‐angiotensin‐aldosterone system in normal cats and cats with hypertrophic cardiomyopathy

### **James Wood** ^1^; Marisa Ames^2^, DVM, DACVIM (Cardiology); Victor Rivas^3^, MS; Joanna Kaplan^4^, DVM, DACVIM (Cardiology); Joshua Stern^5^

#### ^1^University of California‐Davis, Davis, CA, USA; ^2^Associate Professor of Cardiology, Medicine and Epidemiology, University of California‐Davis, Davis, CA, USA, ^3^Graduate Student Researcher, Department of Clinical Sciences, North Carolina State University; ^4^Acting Assistant Professor of Cardiology, Department of Medicine and Epidemiology, University of California‐Davis, Davis, CA, USA; ^5^Associate Dean, Veterinary Medicine, North Carolina State University, Raleigh, NC, USA

**Background:** The local (tissue) renin‐angiotensin system (RAS) has not been characterized in cats.

**Hypothesis/Objectives:** Investigate myocardial and renal tissue RAS regulation in cardiovascularly healthy and hypertrophic cardiomyopathy (HCM) affected cats.

**Animals:** A convenience sample of 17 adult purpose‐bred cats euthanized for other study protocols and confirmed to be healthy (n = 8); ACVIM stage B1 HCM (n = 6); and ACVIM Stage C HCM (n = 3). Cats were not receiving any medications.

**Methods:** Left ventricular myocardium and renal cortex and medulla samples were harvested and immediately flash frozen within 10 minutes of euthanasia, with a second sample held at room temperature for 3 hours prior to freezing. Mass spectrometry was used to quantify serum and tissue angiotensin peptide (AP) concentrations and to estimate select RAS enzyme activities. Tissue AP concentrations were compared between groups using Kruskal‐Wallis analysis. Angiotensin (Ang) II and 1,7 formation rates in fresh and 3‐hour samples were compared using Bland‐Altman analysis.

**Results:** Angiotensin I and II were the only measurable APs in the myocardial samples. Angiotensin I, II, and 1,7 were measurable in all kidney samples. Myocardial and renal AP concentrations did not differ among the groups (AngII: *P* = .32 and *P* = .68 for myocardium and kidney, respectively). Endogenous angiotensin‐converting enzyme, not chymase, was the greatest contributor to AngII formation in both tissues, regardless of disease status. The AP formation rates assay was stable in the 3‐hour samples.

**Conclusions and Clinical Importance:** Tissue RAS was not activated by advanced HCM in this cohort. RAS enzyme activity is stable for at least 3 hours postmortem.

## ABSTRACT C12: Pimobendan alters mitral annular dynamics and reduces mitral regurgitation in dogs: Cardiac computed tomographic assessment

### **I‐Jung B. Chi**; Brian Scansen, DVM, MS, DACVIM (Cardiology); E. Christopher Orton, DVM, PhD, DACVS

#### Colorado State University, Fort Collins, CO, USA

**Background:** Pimobendan is reported to reduce left heart size and extend the preclinical period in dogs with ACVIM stage B2 degenerative mitral valve disease (DMVD). The precise mechanism underlying this benefit is unclear.

**Hypothesis:** Pimobendan enhances systolic contraction of the mitral annulus thereby reducing regurgitant orifice and severity of mitral regurgitation.

**Animals:** Twenty client‐owned dogs with newly diagnosed ACVIM stage B2 DMVD.

**Methods:** Prospective clinical trial. All dogs underwent cardiac computed tomography (CCT) before and 14 days after starting oral pimobendan. Geometric indices of the mitral annulus and cardiac volumes were measured at multiple cardiac phases by CCT. Regurgitant volume and fraction were estimated by calculating left ventricular total stroke volume compared to right ventricular stroke volume. Paired t test or Wilcoxon signed‐rank test was applied for parametric or non‐parametric comparisons between time points.

**Results:** Minimal adverse events were observed (n = 1, mild diarrhea). End‐systolic mitral annular area (*P* < .001), septo‐parietal distance (*P* < .001), inter‐commissural distance (*P* < .001), and trigone‐to‐trigone distance (*P* = .041) were decreased after 14 days of pimobendan, whereas end‐diastolic geometric indices were not different (all *P* values >.05). Left ventricular end‐diastolic volume (*P* < .001), end‐systolic volume (*P* < .01), and stroke volume (*P* = .023) decreased after pimobendan. Left ventricular ejection fraction was unchanged (*P* = 0.35). Regurgitant volume (*P* < .01), regurgitant fraction (*P* < .001), and left atrial volumes (*P* < .001) reduced after pimobendan.

**CONCLUSION:** Short‐term administration of pimobendan augmented systolic contraction of the mitral annulus thereby decreasing the severity of mitral regurgitation. These dynamic changes may underlie observed clinical benefits in dogs with subclinical DMVD.

## ABSTRACT C13: Effects of general anesthesia on echocardiographic indices of pulmonary stenosis severity in dogs

### **Evan S. Ross**; Lance Visser; Lalida Tantisuwat; Khursheed Mama; Brianna Potter; Brian Scansen

#### Colorado State University, Fort Collins, CO, USA

**Background:** General anesthesia (GA) alters blood flow and hemodynamics, which might impact echocardiographic estimates of maximum transpulmonary pressure gradient (maxPG).

**Hypothesis/Objectives:** We sought to describe intra‐operator between‐day reproducibility and determine the effects of GA on maxPG and less flow‐dependent indices of pulmonary stenosis (PS) severity such as, velocity (Vmax) ratio, velocity time integral (VTI) ratio, and indexed pulmonary valve area (iPVA).

**Animals:** Thirty‐nine dogs with PS (maxPG >50 mmHg).

**Methods:** Prospective observational study. Dogs underwent two echocardiographic examinations by the same operator on different days before GA (reproducibility assessment) and after GA. Within group comparisons were made using Wilcoxon's test. Reproducibility was quantitated using coefficients of variation (CV) and reproducibility coefficients (RC).

**Results:** Median (IQR) cardiac index, maxPG, and tricuspid annular plane systolic excursion were significantly (*P* ≤ .001) decreased after GA (percent change: −14.8 [−35.8, 4.8]%, −35.3 [−49.0, −16.5]%, and −38.9 [−49.7, −18.0]%, respectively). Whereas Vmax ratio, VTI ratio, and iPVA were not significantly different (*P* ≥ .35) after GA (0.0 [−10.9, 13.8]%, 0.0 [−10.3, 13.9]%, and −4.3 [−21.6, 12.5]%, respectively). The CVs and RCs were as follows for maxPG: 13.7%, 23.0 mmHg; Vmax ratio: 9.4%, 0.06; VTI ratio: 12.2%, 0.05; and iPVA: 14.3%, 0.12 cm^2^/m^2^.

**Conclusions and Clinical Importance:** Less flow‐dependent indices of PS severity (Vmax ratio, VTI ratio, and iPVA) might be more accurate under GA and in states of altered blood flow. Results support the use of an integrative assessment of PS severity. Clinicians should be mindful of the between‐day variability of indices of PS severity during serial examinations.

## ABSTRACT C14: Perfusion computed tomography of canine pelvic limb vasculature after femoral arterial catheterization: ligation vs. repair

### **Brian A. Scansen** ^1^; Katie Abbott‐Johnson^2^, DVM, MS

#### ^1^Colorado State University, Fort Collins, CO, USA; ^2^Research Associate, Clinical Sciences, Colorado State University, Fort Collins, CO, USA

**Background:** Femoral arterial catheterization (FAC) in dogs often involves ligation of the femoral artery; long‐term impacts of ligation on limb perfusion are unknown.

**Hypothesis:** Dogs with femoral artery ligation have altered limb perfusion compared to repaired dogs.

**Animals:** 18 dogs with previous history of right FAC (ligated = 9; repaired = 9).

**Methods:** Prospective clinical trial. Noninvasive blood pressure (BP) was measured by Doppler, followed by vascular ultrasound of both legs. Dynamic perfusion computed tomography involved 700 mg I/kg contrast bolus followed by 1 ml/kg saline bolus; volume datasets were acquired every 1.5 seconds for 40 acquisitions.

**Results:** All dogs had FAC for occlusion of patent arterial duct. Median (IQR) age at time of FAC was 5.3 (3.7‐7.3) months; median time since FAC was 38.3 (20.5‐59.2) months. Right vs. left pelvic limb BP was not different in ligated dogs (right = 127 ± 17; left = 138 ± 16 mmHg; *P* = .19), or repaired dogs (right = 127 ± 28; left = 139 ± 25 mmHg; *P* = .32). Peak arterial flow velocity in ligated dogs was reduced (*P* < .0001) in the right (37.8 ± 24.3 cm/sec) compared to the left (126.9 ± 29.1 cm/sec) but was not different between limbs in repaired dogs (right = 132.0 ± 37.4; left = 124.0 ± 45.6; *P* = .689). Ligated dogs had extensive collateral perfusion, via caudal gluteal and deep femoral arteries.

**Conclusions:** Perfusion of the pelvic limb is altered after femoral arterial ligation; femoral arterial flow is reduced, and extensive collateralization develops. The functional relevance of these findings requires further study.

## ABSTRACT C15: CK‐586 reduces contractility and ameliorates obstruction in feline hypertrophic cardiomyopathy

### **Victor N. Rivas** ^1^; Jalena Wouters^2^; Betty Yang^2^; Luke Wittenburg^3^, DVM, PhD; Joanna Kaplan^2^, DVM, DACVIM (Cardiology); Darren Hwee^4^, PhD; Anne Murphy^4^, PhD; Bradley Morgan^4^, PhD; Fady Malik^4^, MD, PhD; Samantha Harris^5^, PhD; Joshua Stern^6^, DVM, PhD, DACVIM (Cardiology)

#### ^1^College of Veterinary Medicine, North Carolina State University, Raleigh, NC, USA; ^2^Department of Medicine and Epidemiology, University of California‐Davis, Davis, CA, USA; ^3^Department of Veterinary Surgical and Radiological Sciences, University of California‐Davis, Davis, CA, USA; ^4^Cytokinetics Inc., San Francisco, CA, USA; ^5^Department of Physiology, University of Arizona, Tucson, AZ, USA; ^6^Associate Dean for Research and Graduate Studies, College of Veterinary Medicine, North Carolina State University, Raleigh, NC, USA

**Background:** Hypertrophic cardiomyopathy remains the most common heritable cardiomyopathy in humans and cats with few preclinical pharmacologic interventional studies. Small‐molecule inhibitors that modulate the sarcomere are promising novel therapeutics for the management of obstructive hypertrophic cardiomyopathy (oHCM) patients and have shown efficacy in left ventricular outflow tract obstruction (LVOTO) relief.

**Hypothesis/Objectives:** To explore the 6‐, 24‐, and 48‐hour post‐dose, pharmacodynamic effects of the cardiac myosin inhibitor, CK‐4021586 (CK‐586), in cats with naturally occurring oHCM.

**Animals:** Six purpose‐bred cats bearing the A31P MYBPC3 mutation.

**Methods:** A blinded, randomized, five‐treatment group, crossover preclinical trial was conducted to assess the pharmacodynamic effects of cardiac myosin inhibitor CK‐586 in a feline model of oHCM. Serial plasma concentrations were obtained for dose assessments (2‐15 mg/kg, PO) and select echocardiographic variables were assessed five times over a 48‐hr period.

**Results:** Treatment with oral CK‐586 relieved LVOTO in oHCM cats. In this study, we report the beneficial dose‐dependent effects of CK‐586 treatment by eliminating obstruction through cardiac myosin inhibition and subsequent decreases in LV FS% and EF% in the absence of impact on HR.

**Conclusions and Clinical Importance:** At all tested doses, a single oral dose of CK‐586 was well tolerated and resulted in modest, dose‐dependent, reductions in LV systolic function that was associated with improved or resolved LVOTO. Further studies assessing the long‐term effects of CK‐586 in oHCM‐affected cats are warranted. The results from this study pave the way for the potential use of this compound in both the veterinary and human clinical setting.

## ABSTRACT C16: Short‐term survival following mitral valve repair surgery in 43 dogs

### **Poppy Bristow** ^**1**^; Anne Kurosawa^2^, DVM, MVetMed, PGCert (VetEd), FHEA, DACVIM (Cardiology), MRCVS

#### ^1^N/A; Head of Cardiology, DWR

**Background:** Myxomatous mitral valve disease remains the most common cause of heart failure in dogs. There has been an increase in demand for mitral valve repair (MVR) surgery in the last few years, however, there is still a sparsity of published peer reviewed data in this field.

**Hypothesis/Objectives:** To describe success rates of a new heart surgery programme.

**Animals:** 43 client owned dogs undergoing MVR surgery.

**Methods:** Data from client owned dogs who underwent MVR surgery (artificial chordae tendinae placement and an annuloplasty), under cardiopulmonary bypass, were prospectively collected between September 2021 and August 2023. Dogs were considered suitable for surgery if they were in ACVIM stage C or D of disease, informed owner consent was obtained and minimal comorbidities were present.

**Results:** Cavalier King Charles Spaniels were the most common breed (n = 10, 24%). Median age was 10 years, (range 6‐13 years). 72% of dogs were in ACVIM stage C of disease, with 28% in stage D. Surgical survival was 98%, with one dying due to a protamine reaction. Survival to discharge was 81% overall (n = 35/43); 75% in 2022 and 91% in 2023. Following discharge, one dog was euthanised 6 weeks postoperatively due to unresponsive IMTP.

**Conclusions and Clinical Importance:** Survival following MVR surgery was high in this cohort of dogs, though owners need to be carefully counseled on potential risks.

## ABSTRACT C17: Proximity of coronary arteries to right ventricular outflow tract in dogs with pulmonary valve stenosis

### **Lalida Tantisuwat** ^1^; Brian Scansen^2^, DVM, MS, DACVIM (Cardiology)

#### ^1^Colorado State University, Fort Collins, CO, USA; ^2^Professor, Clinical Sciences, Colorado State University, Fort Collins, CO, USA

**Background:** The coronary arteries (CAs) arise near the pulmonary annulus (PVA). In humans, <3 mm distance from CA to right ventricular outflow tract (RVOT) predicts risk for CA compression during intervention for pulmonary valve stenosis (PS).

**Objective:** Characterize proximity and course of the major CAs relative to the RVOT in dogs with PS by cardiac computed tomography (CCT).

**Animals:** Retrospectively‐gated CCT datasets from 64 PS dogs.

**Methods:** Retrospective study. Studied vessels included right (RCA), left main (LCA), paraconal (LPc), circumflex (LCx), and septal (LSep) CAs. Distance from proximal CAs to RVOT was assessed at end‐systole and end‐diastole at five levels: right ventricular infundibulum, 5 mm below the PVA, PVA, mid‐sinus, and sinotubular junction. Site of closest proximity (both CA branch and RVOT level) was noted for each dog.

**Results:** Seven dogs (10.9%) had a CA anomaly. Median nearest distance between any CA and RVOT was 0.8 mm (range = 0.5‐1.3 mm) at end‐diastole. The CA closest to the RVOT was the RCA in 31.3% of dogs, followed by the LPc (28.1%), LCA (28.1%), LSep (6.3%), and LCx (4.7%). The RCA was most seen adjacent to right ventricular infundibulum/subvalve, LCA was most visualized near sinotubular junction, and LPc was most visualized at the subvalve and PVA.

**Conclusions:** Dogs with PS have CAs proximate to the RVOT and would be considered at high risk for CA compression based on human guidelines. Further work to identify risk for, and prevalence of, CA compression in dogs undergoing PS intervention is warranted.

## ABSTRACT C18: Invasive pressures during right heart catheterization in canine pulmonary stenosis

### **Lalida Tantisuwat** ^1^; Brian Scansen^2^, DVM, MS, DACVIM (Cardiology)

#### ^1^Colorado State University, Fort Collins, CO, USA; ^2^ Professor, Clinical Sciences, Colorado State University, Fort Collins, CO, USA

**Background:** Invasive pressures by right heart catheterization (RHC) in a population of dogs with pulmonary valve stenosis (PS) have not been reported.

**OBJECTIVE:** This study compared RHC parameters between dogs with (Nf16) and without (120) congestive heart failure (CHF). The relationship between awake echocardiographic parameters and RHC measurements was also investigated.

**Animals:** One hundred and thirty‐six dogs with PS.

**Methods:** Retrospective study. Stored RHC recordings before and after intervention were reviewed and measured by a single operator. Invasive RHC parameters were compared between groups. Correlation and agreement between invasive RHC measurements under anesthesia and awake echocardiographic estimates of intracardiac pressures were investigated using Pearson correlation tests and Bland‐Altman plots.

**Results:** Dogs in CHF had higher mean right atrial pressure than non‐CHF dogs (9 vs. 6 mmHg; *P* < .001) whereas peak right ventricular (RV) pressure was not different (*P* = 0.170). Interventions reduced peak RV pressure and increased systolic pulmonary artery pressure, resulting in reduced peak‐to‐peak pressure gradient (PG) and increased pulmonary valve area (PVA; both *P* < .0001). A moderate positive correlation was observed between RHC measurements and echocardiographic estimates for PVA (*r* = 0.541, *P* < .001), maximal PG (*r* = 0.527, *P* < .001) and mean PG (*r* = 0.521, *P* < .001), though limits of agreement were wide.

**Conclusions:** Elevated right atrial pressure during RHC may suggest risk for CHF. Invasive RHC parameters differ from awake echocardiographic estimates of PS severity, though the two methods are correlated.

## ABSTRACT C19: Patent ductus arteriosus characterized by transesophageal echocardiography in dogs

### **Ashley Saunders**; Kendra Zelachowski; Sonya Wesselowski; Sukjung Lim; Sonya Gordon

#### Texas A&M University, College Station, TX, USA

**Background:** Patent ductus arteriosus (PDA) morphology in dogs has relied primarily on angiographic description. Transesophageal echocardiography (TEE) provides unique imaging capabilities.

**Objective:** Characterize PDA based on TEE description.

**Animals:** 299 client‐owned dogs.

**Methods:** Retrospective review of TEE imaging studies to obtain measurements of pulmonary ostium diameter, ampulla length, ampulla width 4 mm above the ostium and at aorta level, and to describe ductal shape, unique characteristics, and unusual morphologies.

**Results:** The most common breeds were mixed (Nf36), German shepherd (Nf33), Labrador retriever (Nf16), and Australian shepherd (Nf15) with a female to male ratio of 204 to 95. Mean ostium diameter was 3.3 mm (SD 1.5; range, 0.6‐9.1 mm). In the majority of dogs, the PDA tapered to an ostium with varying ostium to ampulla ratios (267 < 50%; 32 > 50%) and with combinations of short and long ampullas based on a length to width 4 mm above the ostium ratio (52 < 1.0; 239 > 1.0; Nf291). Additional observed characteristics included the presence of constrictions within the ampulla in 22 dogs, some of which were in the location of device landing zones, and ampulla motion that could be characterized as an extension or expansion throughout the cardiac cycle.

**Conclusions and Clinical Importance:** Information from TEE imaging can aid in refining the current angiographic derived PDA morphology scheme. Imaging with TEE provides an opportunity to make specific measurements and observations about PDA characteristics that can provide valuable information when selecting the best closure method for an individual dog.

## ABSTRACT C20: Trans‐jugular transseptal delivery of the CoApt valve in a canine model

### **George A. Kramer** ^1^; Brienne Williams^1^, DVM, DACVIM (Cardiology); Yanping Cheng^2^, MD; Kacper Nowak^2^, DVM, PhD; Patricia Mount^2^, BS; Genghua Yi^2^, MD; Robert Moon^2^, BS; Gerard Conditt^2^, RCIS

#### ^1^Atlantic Coast Veterinary Specialists, Bohemia, NY, USA; ^2^CRF Skirball Center for Innovation, New York, NY, USA

**Background:** Degenerative mitral valve disease is the most common heart disease in dogs. Successful development of a percutaneous transcatheter mitral valve would have a dramatic effect on the treatment of this disease.

**Hypothesis/Objective:** We hypothesized that our valve could be deployed through a 9.5 Fr catheter via the trans‐jugular transseptal route without untoward effects on the heart.

**Animals:** Two purpose‐bred research dogs were used in this study.

**Methods:** The study was a case‐series design. Valve delivery was performed through a 9.5 Fr steerable sheath under fluoroscopic and transesophageal echocardiographic guidance. Deployment of three anchors in the LV apex preceded the valve assembly implantation in the mitral annulus. Echocardiographic evaluations of heart function and valve performance were performed post‐implantation, and days 1, 14, 30. Animals were euthanized at day 30, and the hearts underwent histologic assessment.

**Results:** Successful deployment and anchoring of the valve in the mitral annulus was achieved in one dog. In the other, the anchoring system deployed successfully but there was malalignment of the valve assembly. Trace MR was observed in both dogs after implantation without LVOT obstruction or pericardial effusion. Follow‐up echocardiograms indicated that the devices remained in the initial position, with mean transvalvular pressure gradient of 1 mmHg and trace to mild MR. Normal LV function was maintained without LVOT obstruction (mean PG <3 mmHg) or pericardial effusion observed from day 1 to day 30.

**Conclusions and Clinical Importance:** Transcatheter placement of the valves had no negative effects on the subjects, which will allow us to proceed to clinical trials.

## ABSTRACT C21: Comparison of three‐dimensional mitral valve morphologic measurements obtained using two different ultrasound machines

### **Annie Showers** ^1^; Giulio Menciotti^2^, DVM, MS, PhD, DACVIM (Cardiology), DECVIM‐CA (Cardiology)

#### ^1^Virginia Tech, Blacksburg, VA, USA; ^2^VA‐MD College of Veterinary Medicine

**Background:** Real‐time three‐dimensional transthoracic echocardiography (RT‐3DTTE) has been utilized to assess the geometry of the mitral valve (MV) in dogs. Although cross‐platform software for MV 3D modeling exists, it is unknown whether the machine used to acquire images affects MV morphologic variables.

**Objective:** To compare MV morphologic variables between RT‐3DTTE datasets acquired with two different ultrasound machines.

**Animals:** Eleven dogs.

**Methods:** Prospective diagnostic comparison study. Each dog underwent RT‐3DTTE acquisition using two different ultrasound machines: Philips Epiq CVx and Canon Aplio i900. RT‐3DTTE datasets were analyzed with dedicated software for MV morphologic analysis by a single observer. Bland‐Altman analysis was performed for each variable. The mean of the absolute differences was compared to previously reported inter‐observer repeatability coefficients (RC).

**Results:** A significant bias between Philips and Canon measurements was identified for the following MV morphologic variables: normalized anterolateral‐posteromedial annulus diameter (−(−0.11 [−0.21‐0.02]; *P* = .02), sphericity index (0.06 [0.005‐0.11]; *P* = .03), non‐planar angle (12.43 [3.20‐21.65]; *P* = .01), tenting area (−0.15 [−0.27‐0.03]; *P* = .02), tenting height (−0.98 [−1.79‐0.17]; *P* = .02), and normalized commissural diameter (−0.12 [−0.22‐0.02]; *P* = .02). The measurement difference was greater than previously reported inter‐observer RCs for the non‐planar angle, annulus height, tenting height, normalized posterior leaflet area, and annulus height to commissural width ratio, suggesting that the difference is beyond what expected just by inter‐observer variability.

**Conclusions and Clinical Importance:** MV morphologic variable measurements can differ between ultrasound machines. These differences may be relevant when utilizing RT‐3DTTE for patient assessment influencing surgical planning.

## ABSTRACT C22: Cardiac computed tomographic assessment of the pulmonary outflow tract in dogs with pulmonary valve stenosis

### **Lalida Tantisuwat** ^1^; Brian Scansen^2^, DVM, MS, DACVIM (Cardiology)

#### ^1^Colorado State University, Fort Collins, CO, USA; ^2^Professor, Clinical Sciences, Colorado State University, Fort Collins, CO, USA

**Background:** Computed tomography (CT) aids planning of structural heart interventions in human medicine. Device sizing for pulmonary valve stenosis (PS) intervention in dogs relies on 2‐dimensional (2D) imaging.

**OBJECTIVE:** Obtain detailed measurements of the pulmonary outflow tract in dogs with PS.

**Animals:** Retrospectively‐gated cardiac CT datasets from 64 PS dogs.

**Methods:** Retrospective study. End‐diastolic (ED) and end‐systolic (ES) right ventricular (RV) volumes were calculated. Area, perimeter, and minimum/maximum diameter of the pulmonary outflow tract were obtained at eight levels, both at ED and ES. The pulmonary valve annulus (PVA) area‐derived diameter and annular eccentricity were calculated. Percent leaflet fibrosis was evaluated at PVA, mid‐sinus, and sino‐tubular junction (STJ).

**Results:** Median (range) RV ED and ES volumes were 7.30 (4.23, 12.15) and 16.45 (11.75, 28.23) mL, with ejection fraction of 58% (43, 70). Median PVA maximal diameter was 13.85 (12.20, 16.75) mm, minimal diameter was 11.15 (9.20, 13.15) mm, and PVA area‐derived diameter was 12.40 (10.73, 14.75) mm. Change of PVA area‐derived diameter from ED to ES was 2.95% (−3.15, 13.28). PVA eccentricity was 0.2 (0.14, 0.28), suggesting ovoid geometry. Pulmonary valve opening area was 17 (12, 24) mm2, averaging 14.1% of PVA area. The greatest fibrosis area (57.5%) was present at the STJ, ranging from 34.3% to 69.5%.

**Conclusions:** Eccentricity of the PVA suggests that measurements obtained from 2D imaging may underestimate or overestimate size of device. Fibrosis of the leaflets, particularly the STJ, was a dominant lesion in this population of PS dogs.

## ABSTRACT C23: Clinical outcomes through 3 months in dogs with MMVD treated with the TEER

### **Jeongmin Lee** ^1^; Jinhwa Chang^1^, DVM, PhD; Young‐Wook Cho^1^, DVM; Jiwoong Her^2^, DVM, MS, DACVECC; Youn‐Seo Jung^1^, DVM; Ah‐ra Lee^1^, DVM, PhD; Kyoung‐A Youp^1^, DVM, MS; Sun‐tae Lee^1^, DVM, MS

#### ^1^Korea Animal Medical Center; ^2^The Ohio State University Veterinary Medical Center

**Background:** Transcatheter edge‐to‐edge repair (TEER) is an emerging treatment option for dogs with myxomatous valve disease (MMVD) but reports on the effectiveness and safety of TEER are lacking.

**Hypothesis/Objectives:** To report the clinical outcomes through 3 months in four dogs with MMVD with TEER.

**Animals:** Four client‐owned dogs undergoing TEER.

**Methods:** This retrospective study included four dogs treated with TEER using the V‐clamp device. The following data were obtained from the medical records for analyses: history, clinical signs, body weight, complete blood count, serum chemistry, venous blood gas analysis, cardiac troponin I, NT‐proBNP, thoracic radiography, and trans‐thoracic echocardiography results.

**Results:** Two dogs were in advanced MMVD stage B2, one in MMVD stage C and one in MMVD stage D. Before the surgical procedure, pimobendan and furosemide were prescribed for all dogs. The median body weight was 3.68 kg (range, 2.44‐6.28 kg). After the surgical procedure, all dogs exhibited a significant reduction in mitral regurgitation. Three dogs successfully discontinued diuretics whereas one dog required diuretics starting from the third month. No postoperative complications were observed during the three‐month observation period.

**Conclusions and Clinical Importance:** TEER with the V‐clamp device showed promising short‐term efficacy in managing myxomatous valve disease in dogs.

## ABSTRACT C25: The angiotensin‐converting enzyme polymorphism in the north american irish wolfhound: Variant‐positive prevalence and clinical implications

### **Emily Suess‐Radford** ^1^, CVCA; Robin Shoemaker^2^, PhD; Josh Stern^3^, DVM, PhD, DACVIM (Cardiology); Bill Tyrrell^4^, DVM, DACVIM (Cardiology)

#### ^1^Cardiac Care for Pets; ^2^Assistant Professor, UK RAAS Analytical Lab, Department of Dietetics and Human Nutrition, University of Kentucky College of Agriculture, Food, and Environment; ^3^Associate Dean for Research & Graduate Studies, Cardiology, NCSU College of Veterinary Medicine; ^4^Medical Director, CVCA—Cardiac Care for Pets

**Background:** An angiotensin‐converting enzyme (ACE) gene polymorphism has been identified in dogs. Its functional importance appears to be variable and possibly breed‐dependent.

1. **Hypothesis:** The majority of Irish Wolfhounds (IWs) would be homozygous or heterozygous for the ACE‐polymorphism.
2. IWs with this polymorphism would demonstrate decreased classical renin‐angiotensin‐aldosterone system (RAAS) pathway activation and increased alternative RAAS pathway utilization both at baseline and after angiotensin converting enzyme inhibitor (ACEi) administration.
3. IWs without the mutation would exhibit more typical RAAS pathway parameters at baseline and after ACEi administration.

**Animals:** 144 client‐owned, purebred IWs were genotyped and phenotypically described via cardiovascular evaluation (auscultation, echocardiogram, and ECG) over a 3 year period. Eighteen IWs that were deemed phenotypically normal were then selected for RAAS fingerprinting before and after an ACEi challenge: two wild types (WT), four heterozygotes (Aa), and 12 homozygotes (aa).

**Methods:** All IWs were phenotypically described under the direct supervision of a boarded cardiologist. For the purposes of this study, phenotypically “normal” was categorized by a normal echocardiogram (based on values previously established for IWs), normal auscultation, and a normal sinus rhythm/sinus arrhythmia on ECG. Blood draws were performed on the pre‐selected phenotypically normal IWs at baseline and post two‐week ACEi challenge. Equilibrium analysis was performed to evaluate serum RAAS metabolites and enzyme activities.

**Results:** In our population, 46 IWs (31.9%) had atrial fibrillation and varying degrees of heart muscle dysfunction/enlargement consistent with Irish wolfhound cardiomyopathy. 96 IWs (66.6%) were phenotypically normal. With genotyping, 120 (83.3%) of IWs were homozygous, 19 (13.2%) were heterozygous, and 5 (3.5%) were homozygous WT for the polymorphism.

In the selected phenotypically normal IWs, whole group analysis showed all IWs exhibited normal baseline RAAS activity. After a two‐week ACEi challenge (benazepril 0.5 mg/kg every 12 hours), all IWs also displayed an appropriate inhibition of the classical RAAS pathway and a significant up‐regulation of the alternative pathway.

**Conclusion:** The ACE‐polymorphism appears to be non‐functional in the IW. When discovering new mutations, further investigation is warranted to determine the penetrance and expressivity (as it may be breed‐dependent).

## ABSTRACT C28: Association between the mitral insufficiency echocardiographic score and progression of myxomatous mitral valve disease

### **Soh‐Yeon Lee**; SeHoon Kim; Min‐Ok Ryu; Hwa‐Young Youn; KyoungWon Seo

#### Laboratory of Veterinary Internal Medicine, College of Veterinary Medicine, Seoul National University

**Background:** The Mitral Insufficiency Echocardiographic (MINE) score, validated for Myxomatous Mitral Valve Disease (MMVD) severity assessment, prognosticates survival outcomes.

**Objectives:** This study aimed to employ the MINE score at MMVD stage B2 diagnosis to correlate with disease progression to stage C.

**Animals:** The study analyzed the medical records of 83 dogs diagnosed with stage B2 MMVD at Seoul National University Veterinary Medicine Teaching Hospital (May 2018‐August 2023).

**Methods:** These dogs underwent regular echocardiographic evaluations (minimum four assessments annually post‐diagnosis). The cohort was divided into two groups: 53 dogs progressing to stage C and a control group (remaining in stage B2 beyond the mean progression time).

**Results:** Categorizing based on MINE scores (mild, moderate, severe), mean progression times to stage C were 811 days (mild), 544 days (moderate), and 336 days (severe). Statistical analysis revealed a significant correlation (*P* < .01).

**Conclusions and Clinical Importance:** The study suggests the MINE score may predict MMVD stage B2 to C transition, allowing early intervention for high‐risk patients. Acknowledging limitations like the small sample size and lacking MINE scores at later stages, further large‐scale studies with comprehensive MINE score evaluations are crucial for increased clinical applicability in MMVD management. This study highlights the importance of early risk assessment and intervention in potentially improving patient outcomes.

## ABSTRACT C29: The impact of vericiguat, a soluble guanylate cyclase stimulator, on cardiovascular properties in baroreflex‐absent dogs

### **Aimi Yokoi** ^1^; Midori Kakuuchi^2^; Akitsugu Nishiura^2^; Sho Fukuzumi^2^; Shohei Yokota^2^; Hiroki Matsushita^2^; Kazunori Uemura^2^; Toru Kawada^2^; Ryou Tanaka^3^; Keita Saku^2^

#### ^1^Department of Veterinary Surgery, Tokyo University of Agriculture and Technology; ^2^Department of Cardiovascular Dynamics, National Cerebral and Cardiovascular Center; ^3^Department of Veterinary Surgery, Tokyo University of Agriculture and Technology

**Background:** Vericiguat, a direct soluble guanylate cyclase (sGC) stimulator, improves heart failure outcomes in human, including reduced rehospitalization rates. However, its specific effects on individual cardiovascular properties, such as heart rate, cardiac function, systemic vascular resistance (SVR), and stressed blood volume (SBV), remain unclear.

**Objectives:** Given vasodilators trigger baroreflex‐mediated sympathetic activation, we aimed to evaluate the isolated impact of vericiguat on cardiovascular properties in baroreflex‐absent dogs.

**Animals:** Five healthy beagle dogs under general anesthesia were studied.

**Methods:** Arterial pressure (AP), right atrial pressure (RAP), left atrial pressure (LAP), and cardiac output (CO) were measured simultaneously. SBV was calculated as (CO + 19.61*RAP+3.49*LAP)*0.129. We infused vericiguat (30 γ) for 10 minutes and assessed its effects on cardiovascular properties in the absence of baroreflex AP buffering (sino‐aortic denervation).

**Results:** In baroreflex‐absent dogs, vericiguat reduced both mean AP and CO without significantly altering atrial pressures. It significantly decreased SVR (71.8 vs. 49.9 mmHg*min/L, *P* < .05) but not left ventricular pump function (SL, the slope of CO curve: 55.6 vs. 57.4 mL/min*kg, NS). SBV also decreased significantly (28.9 vs. 27.6 mL/kg, *P* < .05), while the change was modest.

**Conclusions and Clinical Importance:** The acute hemodynamic effect of vericiguat primarily resulted from SVR reduction. These findings can inform optimized drug therapy strategies for heart failure patients.

## ABSTRACT C30: Insight into the association of angiotensin converting enzyme gene polymorphism with dilated cardiomyopathy in dogs

### **Sindhu K. Rajan** ^1^; G. Radhika^2^, PhD; M. Shynu^3^, PhD; Usha Pillai^4^, PhD; S.V. Ajithkumar^4^, PhD; Madhavan Unny^4^, PhD

#### ^1^Kerala Veterinary and Animal Sciences University, India; ^2^Professor, Department of Animal Breeding and Genetics, Kerala Veterinary and Animal Sciences University, India; ^3^Professor, Department of Veterinary Biochemistry, Kerala Veterinary and Animal Sciences University, India; ^4^Professor, Department of Veterinary Clinical Medicine Ethics and Jurisprudence, Kerala Veterinary and Animal Sciences University, India

**Background:** Angiotensin converting enzyme (ACE) gene polymorphism has been previously demonstrated in the dogs with cardiac disease. But the clinical significance of this finding was not studied in detail.

**Hypothesis:** Dogs with cardiac diseases would be predominantly polymorphism positive irrespective of the type of cardiac diseases.

**Animals:** Dogs brought to the University Veterinary Hospitals under Kerala Veterinary and Animal Sciences University for the past 2 years were screened for the presence of cardiac diseases. Fifty‐one apparently healthy dogs and 71 dogs with cardiac disease such as myxomatous mitral valve disease and dilated cardiomyopathy (MMVD and DCM) were randomly selected for the study.

**Methods:** Healthy dogs and dogs with cardiac diseases were genotyped for ACE gene polymorphism (rs850683722 SNP). Pearson's Chi square statistic was performed to assess the association of genotype among healthy and diseased population and also to assess the association of genotype with DCM and MMVD.

**Results:** The genotype varied significantly between healthy and diseased population (*P*‐value = .008) as per Pearson's Chi square statistic (9.622). Furthermore, genotype also varied significantly (*P* = .008) with type of cardiac diseases on Pearson's Chi square statistic (25.422). The homozygous variant genotype (AA) was frequently occurred in dilated cardiomyopathy cases as compared to mitral valve disease.

**Conclusions and CLINICAL Importance:** Positive association of homozygous variant genotype with DCM, indicated the possibility of involvement of variant genotype in the development and the progression of disease. Hence, dogs with homozygous variant genotype may be excluded from breeding to reduce the occurrence of DCM.

## ABSTRACT C31: Assessment of electrocardiography and thoracic radiography to identify echocardiographic structural heart disease in cats

### **Matthew Denton** ^1^; Sukjung Lim^2^, DVM; Jenny Applebaum^3^, DVM; Grace Flynn^3^; Sonya Gordon^4^, BSc, DVM, DVSc, DACVIM (Cardiology)

#### ^1^Texas A&M University; ^2^Cardiology Intern, Cardiology, Texas A&M University; ^3^Rotating Intern, Texas A&M University; ^4^Professor of Cardiology, Cardiology, Texas A&M University

**Background:** Echocardiography is the preferred test to diagnose and stage feline cardiac disease, however it may not be available resulting in the desire to use more readily available tests to screen for clinically relevant structural heart disease [HD]. The potential utility of electrocardiography [ECG] and thoracic radiography have not been assessed for this indication.

**Objective:** Assess the clinical utility of an ECG and radiography to identify cats at high risk for having HD.

**Animals:** Feline telemedicine cardiology case submissions to IDEXX laboratories that included contemporaneous ECG, echocardiogram, and thoracic radiographs (Nf400).

**Methods:** Echocardiography reports were reviewed, and cats were categorized as structurally normal [SN], equivocal [ED], and HD. Vertebral heart size [VHS] and subjective classification of cardiomegaly [SC] were recorded. Selected ECG findings were recorded from reports and categorized as dysrhythmia [DR], conduction abnormality [CA] and tall R wave [TR]. Discriminatory ability of individual tests/test categories to identify HD versus SN + ED was evaluated.

**Results:** Prevalence of SN, ED and HD was 40.0%, 33.5%, 26.5% respectively. Cardiomyopathy represented 97.2% (103/106) of HD diagnoses. The area under the curve for VHS to discriminate between HD and SN + ED was 0.763. A VHS cutoff of > = 8.5 had 67.5% sensitivity and 78.6% specificity. For radiographic SC, the classification accuracy was 65.8% with 86.4% sensitivity and 58.4% specificity. For the ECG categories DR, CA, and TR the correct classification of HD versus SN + ED was 65.2%, 57.1%, and 69.7% respectively.

**Conclusions:** Radiography and electrocardiography have acceptable discriminatory ability to detect feline HD and may be useful as part of a risk assessment screening protocol.

## ABSTRACT C32: Outcome of canine mitral valve repair for myxomatous mitral valve disease with severe pulmonary hypertension

### **Kentaro Kurogochi**; Masami Uechi, DVM, PhD, DACVIM (Cardiology)

#### JASMINE Veterinary Cardiovascular Medical Center

**Background:** Mitral valve repair (MVR) is a potential curative treatment for myxomatous mitral valve disease (MMVD) in dogs. Pulmonary hypertension (PH) commonly accompanies MMVD, yet its prognostic impact on MVR outcomes is unclear.

**Hypothesis/Objectives:** To assess MVR outcomes in dogs with severe PH and provide insights for MVR candidacy.

**Animals:** We retrospectively analyzed MMVD dogs with severe PH that underwent MVR at our institute from February 2017 to December 2020. Severe PH was diagnosed based on the ACVIM consensus and defined as tricuspid valve regurgitant pressure gradient (TRPG) above 75 mmHg.

**Methods:** Over a 3‐6‐year postoperative follow‐up, patients were categorized into two groups: those who developed PH post‐surgery (PH group), and those who did not (non‐PH group). Echocardiographic values and clinical course were evaluated based on medical records.

**Results:** Seventeen dogs were included (non‐PH: n = 9, PH: n = 8). There is no difference between non‐PH and PH in preoperative TRPG (83 [79‐95] vs. 85 [83‐89] mm Hg, *P* = .888). One month after surgery, TRPG was significantly higher in the PH group (27 [23‐47] vs. 59 [44‐71] mm Hg, *P* = .02). Both groups showed significant TRPG reduction after surgery (*P* < .01, *P* < .01). The median time to develop right congestive heart failure (n = 6) was 441 days [101‐1084] post‐surgery.

**Conclusions and Clinical Importance:** Postoperative TRPG reductions are achievable by MVR in dogs with severe PH. The data suggest that MVR can be a viable option to manage MMVD in dogs with concomitant severe PH.

## ABSTRACT C33: Ratio of vena contracta width to mitral commissural diameter in canine degenerative mitral valve disease

### **Zack T. English** ^1^; Christopher Orton^2^, DACVS, DACVIM (Cardiology) (Honorary); Brianna Potter^3^, DACVIM (Cardiology); Lance Visser^4^, DACVIM (Cardiology)

#### ^1^Colorado State University; ^2^Helen D. Van Dyke Chair, Interventional Cardiology and Cardiac Surgery, Professor, Clinical Sciences, Colorado State University; ^3^Assistant Professor, Cardiology, Clinical Sciences, Colorado State University; ^4^Associate Professor, Cardiology, Clinical Sciences, Colorado State University

**Background:** Mitral annular dilation contributes to worsening mitral regurgitation (MR) by causing separation of the mitral leaflets (Carpentier type I defect). Widening of the vena contracta along the zone of leaflet apposition could reflect this mechanism of secondary functional mitral regurgitation (FMR) and may have implications for prognosis and mitral intervention in dogs with degenerative mitral valve disease (DMVD).

**Hypothesis:** The percent‐ratio of vena contracta width (VCW) to mitral valve commissural diameter (MVCD) increases with disease stage in dogs with DMVD.

**Animals:** Fifty‐four client‐owned dogs with ACVIM stage B1 (n = 17), stage B2 (n = 18), or stage C (n = 19) DMVD. Inclusion criteria were ≥ 5 years of age, body weight ≤ 25 kg, and systolic blood pressure.

**Methods:** Echocardiograms were performed by a cardiologist (BMP) and measurements performed by a single investigator (ZTE). Measurements of mid‐systolic MVCD were obtained from left apical commissural view and normalized to aortic valve diameter. VCW was measured from the same mid‐systolic left apical commissural view. Differences between disease stage were determined by one‐way ANOVA.

**Results:** The normalized MVCD increased (*P* = .0002) with disease stage (B1 = 1.50, B2 = 1.77, C = 2.17). The percent‐ratio of VCW to MVCD increased (*P* < .0001) with disease stage (B1 = 19.7%, B2 = 37.0%, C = 51.8%).

**Conclusions:** Widening of the vena contracta relative to the MVCD supports a conclusion that mitral annular dilation and resultant secondary FMR (Carpentier type I defect) is associated with progression of DMVD. This mechanism may have implications for prognosis as well as predict MR reduction in dogs undergoing mitral intervention.

## ABSTRACT C34: The role of vector‐borne pathogens and striatin genotype in boxers with arrhythmogenic right ventricular cardiomyopathy

### **Bobbie Ditzler** ^1^; Ed Breitschwerdt^2^; Erin Lashnits^3^; Ricardo Maggi^2^; Kathryn Meurs^2^; Pradeep Neupane^2^; Mariko Yata^4^

#### ^1^Texas A&M University; ^2^North Carolina State University; ^3^University of Wisconsin‐Madison; ^4^The Pet Specialists

**Background:** Risk factors for severe disease in Boxer dogs with arrhythmogenic right ventricular cardiomyopathy (ARVC) are not well understood. This study was performed to better understand triggers for increasing disease severity of Boxer ARVC and to better inform clinical outcome.

**OBJECTIVE:** This study's objective was to determine whether Striatin genotype or canine vector borne pathogen (CVBP) exposure/infection in Boxer dogs with ARVC was associated with disease severity or survival.

**Animals:** Sixty‐four client‐owned, adult Boxer dogs with ARVC.

**Methods:** Prospective descriptive study. Disease severity was determined by echocardiography and Holter monitoring. Potential risk factors included CVBP exposure/infection (*Anaplasma* spp., *Babesia* spp., *Bartonella* spp., *Borrelia burgdorferi*, *Dirofilaria immitis*, *Ehrlichia* spp., and *Rickettsia* spp.) and Striatin genotype.

**Results:** Median survival time after enrollment was 270 days (95% confidence interval [CI] 226‐798 days), and median age at time of death or censoring was 11 years (95% CI 10.3‐11.7 years). Striatin mutation genotype results included 31 homozygous negative, 26 heterozygous and seven homozygous positive. Ten Boxer dogs had exposure to *Bartonella* spp., four to *Rickettsia*, two to *Ehrlichia* spp., one to *Anaplasma* spp. Striatin homozygous positive Boxer dogs had a statistically significantly shorter median survival time (93 days vs. 373 days for heterozygous [*P* = .010] and 214 days for homozygous negative [*P* = .036]). Exposure/infection to CVBP was not statistically significantly associated with median survival time or age at time of death.

**Conclusions:** Striatin genotype screening can be considered for prognostic information. Exposure/infection to CVBP appears unlikely to influence survival time for Boxer dogs with ARVC.

## ABSTRACT C35: Transcatheter edge‐to‐edge mitral valve repair for myxomatous mitral valve disease in dogs under 2.5 kg

### **Jeongmin Lee** ^1^; Kyoung‐A Youp^1^, DVM, MS; Dongmin Sihn^3^, DVM, MS

#### ^1^Korea Animal Medical Center; ^3^Ilsan Animal Medical Center

**Background:** Transcatheter Edge‐to‐Edge mitral valve repair (TEER) using v‐clamp is a hybrid surgery that installs a clamp on mitral valve as an intervention after thoracotomy to Myxomatous Mitral Valve Disease (MMVD) patient. The existing standard for a patient's weight for TEER is 4 kg, and it is known that risk is very high or impossible for those under 2.8 kg. However, it was figured out that TEER was possible in patients under 2.5 kg.

**OBJECTIVES:** To report TEER surgery in small patients under 2.5 kg.

**ANIMAL:** 3 dogs with MMVD stage B2 ~ D, had TEER surgery.

**Methods:** Case series, Ventral 1/3 thoracotomy at 7‐8th intercostal space, insert sheath catheter from heart apex to left atrium, emplace of v‐clamp in mitral valve under Trans‐Esophageal Echocardiography (TEE).

**Result:** TEER was successful in all three dogs. Size II clamp was used in all patients. Average surgery time was 120 minutes. Recovery time varied from 2 to 7 days and was affected by MMVD stage. Size of heart was more important than weight. If annulus diameter was more than 12 mm, TEER was possible. 5‐Fr sheath was installed to femoral artery to measure arterial pressure. Because of the small heart, cardiac axis was seen as distorted on TEE, making surgery difficult. The size of the left atrium is small, details of adjusting the gap between two clamps are necessary. TEER in small hearts has a risk of postoperative mitral stenosis, so careful evaluation during surgery must be performed.

**Conclusions:** TEER can be done successfully with careful details under 2.5 kg dogs.

## ABSTRACT C36: Investigations of asymmetric dimethyl arginine (ADMA) as a biomarker in preclinical myxomatous mitral valve disease

### **Cosette Ayoub** ^1^; Melanie Hezzell^2^; Marco Mazzarella^3^, DVM, MRCVS; Jade Ward^4^, BSc (Hons), RVN

#### ^1^Ontario Veterinary College; ^2^University of Bristol; ^3^PhD Student, University of Bristol; ^4^Veterinary Nurse Research Technician, University of Bristol

**Background:** Asymmetric dimethyl arginine (ADMA) is a biomarker of endothelial dysfunction. Increased circulating ADMA has been demonstrated in dogs with congestive heart failure secondary to myxomatous mitral valve disease (MMVD). We hypothesize that endothelial dysfunction, and therefore plasma ADMA, is greater in dogs with stage B2 (preclinical MMVD with cardiomegaly) vs stage B1 (preclinical MMVD without cardiomegaly).

**Objectives:** To compare plasma ADMA measurements between dogs with stage B1 vs B2 MMVD.

**Animals:** 42 dogs of a variety of breeds examined between March 2020 and August 2023 (16/42 B, 26/42 B2).

**Methods:** Prospective, cross‐sectional study. All dogs underwent history, physical examination, blood pressure measurement, electrocardiography, echocardiography and blood sampling for diagnostic testing. Surplus plasma was stored at −80°C for batched measurement of ADMA using a commercially available competitive ELISA. Between groups comparisons were made using Mann Whitney‐*U* tests. Receiver operator characteristic (ROC) curve analysis was used to test the performance of plasma ADMA in differentiating stage B1 from stage B2.

**Results:** Plasma ADMA measurements were significantly higher in dogs with stage B2 (median = 24.26 ng/mL [minimum = 19.44, maximum = 57.88]) versus stage B1 MMVD (19.31 ng/mL [13.94‐22.56]) MMVD (*P* < .0001).

**Conclusions and Clinical Importance:** Dogs with preclinical MMVD and plasma ADMA>21.41 ng/mL are more likely to be in stage B2; this might be useful in primary practice to help prioritize dogs for echocardiographic assessment.

## ABSTRACT C37: Changes to pacemaker programming parameters over time in dogs

### **Kailah M. Buchanan** ^1^; Shana Mintz^2^, DVM, DACVIM (Cardiology); Weihow Hsue^2^, DVM, DACVIM (Cardiology)

#### ^1^Cornell University; ^2^Assistant Professor, Department of Clinical Sciences, Cornell University

**Background:** Although permanent pacemakers are routinely used to treat symptomatic bradyarrhythmias, long‐term changes in interrogated and programmed parameters are not well characterized.

**Objectives:** The primary goal is to identify pacemaker parameters that significantly change over time. Secondary aims include comparing patient or pacemaker characteristics, such as body weight, generator brand, lead fixation type, etc., in relation to these changes and complications.

**Animals:** Seventy‐three client‐owned dogs who received a pacemaker and had at least two subsequent interrogations were included.

**Methods:** Retrospective observational study. Patient characteristic and pacemaker parameters were documented. Timing of recheck interrogations were grouped for analysis in a linear mixed effects model (Table 1). Complications noted at each interrogation were recorded.

**Results:** Pulse width, battery life, and battery impedance significantly changed over time (Figure 1). Patients under 7 years old had less battery life overall (*P* = .0014) as well as at multiple time points. Patients with endocardial leads with active fixation had a higher amplitude threshold (*P* = .0013), programmed amplitude (*P* = .0073), and sensitivity (*P* < .001), but there was no difference in the proportion of complications between types of fixation. Medtronic generators had more programming‐related complications (*P* = .0102) and required a numerically higher sensitivity (*P* = .0018).

**Conclusions and Clinical Importance:** Some pacemaker parameters changed over time, highlighting the necessity for evaluation of the individual patient. Certain patient or pacemaker characteristics may influence pacemaker function, with lead fixation type showing particular importance, but may not lead to more complications.

**Table:** Table 1. Bins used for statistical analysis of changes to pacemaker parameters over time

Bin	Time since pacemaker implantation
0	24 hours
1	0.25‐ < 2 months
2	2‐ < 4 months
3	4‐ < 8 months
4	8‐12 months
5	13‐24 months
6	25‐36 months
7	≥37 months

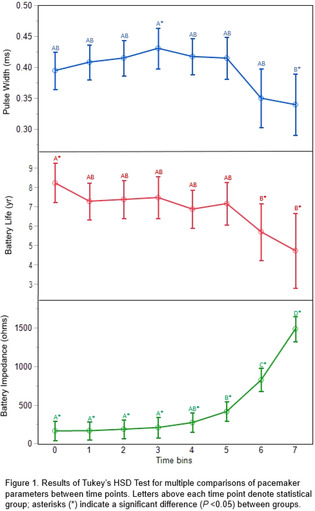

## ABSTRACT C38: A prospective comparative study on novel and conventional inotropic drugs in clinically healthy cats

### **Mio Ishizaka**; HuaiHsun Hsu, DVM; Akiko Miyagawa, DVM; Yuichi Miyagawa, PhD; Naoyuki Takemura, PhD

#### Nippon Veterinary and Life Science University

**Background:** Heart failure (HF) treatment involves various strategies, including inotropic agents and diuretics. Conventional inotropic agents raise concerns about side effects such as arrhythmias and myocardial ischemia. Selective cardiac myosin activators in human medicine enhance contractility without adverse events. If novel agents prove effective or better than traditional ones, it may impact treatment in veterinary medicine.

**Objectives:** This study aimed to compare the effects of novel inotropic drugs with conventional HF medications.

**Animals:** This study involved five clinically healthy cats.

**Methods:** Employing a prospective cohort study design, cats were administered omecamtiv mecarbil (OM), dobutamine, pimobendan, and milrinone, and underwent echocardiographic examination.

**Results:** OM significantly reduced heart rate compared to other inotropic agents (*P* = 0.044). No significant differences were observed in blood pressure among the drugs. Tricuspid valve annulus and mitral annular septal systolic velocity significantly increased with dobutamine compared to other inotropic agents (*P* = .044). Mitral annulus post‐systolic excursion significantly increased with OM and dobutamine compared to milrinone (*P* = .044). Left ventricular ejection time was significantly prolonged with OM compared to milrinone (*P* = .044). Left ventricular global longitudinal strain (GLS) significantly increased with dobutamine compared to milrinone (*P* = .044). GLS exhibited comparable increases with OM and dobutamine.

**Conclusions and Clinical Importance:** OM demonstrates inotropic effects similar to dobutamine and pimobendan, with a stronger contractile enhancement than milrinone. With its inotropic effects and rate control, OM holds promise as a veterinary therapy.

## ABSTRACT C39: Assessment of the eko core stethoscope‐analysis software system to detect canine heart murmurs

### **Jennifer P. Applebaum** ^1^; Sonya Gordon^2^, BSc, DVM, DVSc, DACVIM (Cardiology); Sukjung Lim^3^, DVM

#### ^1^Texas A&M University; ^2^Professor, Cardiology, Texas A&M University; ^3^Research Intern, Cardiology, Texas A&M University

**Background:** The EKO Core Stethoscope‐Analysis Software system (Eko‐system) is an FDA‐cleared software intended to provide support to physicians in the evaluation of human patients' heart sounds. There is scarce data regarding the possible utility of this technology in dogs.

**Objective:** Assessment of the Eko‐system's ability to detect canine heart murmurs of variable intensity.

**Animals:** Sixty‐one Cavalier King Charles Spaniels were recruited from an auscultation screening clinic for myxomatous mitral valve disease (MMVD).

**Methods:** Dogs underwent auscultation by three observers. A board‐certified cardiologist and an experienced veterinarian listened with traditional stethoscopes. The third observer, an early‐career veterinarian, used the Eko‐system to auscultate, record, and analyze 30‐second heart sound recordings. Observers were blinded to each other's results. Murmurs were graded I‐VI, and findings were compared to the cardiologist's assessment.

**Results:** Auscultation by a cardiologist revealed systolic murmurs in 32 dogs whilst 29 had no murmur (1‐I, 10‐II, 7‐III, 7‐IV, 5‐V, 1‐VI). The Eko‐system achieved 78.7% accuracy in identifying murmurs, with 75.9% sensitivity and 81.3% specificity. For louder murmurs (grades III‐VI), accuracy rose to 84.0% with 88.9% sensitivity and 81.3% specificity. For softer murmurs (grades I & II), sensitivity and specificity were 54.5% and 81.3%. Overall murmur classification accuracy for the experienced veterinarian was 81.7% with 93.1% sensitivity and 71.0% specificity.

**Conclusions and Clinical Importance:** The Eko‐system's classification accuracy for murmur detection was similar to an experienced veterinarian. Accuracy for the Eko‐system was best for murmurs ≥III/VI. These findings suggest its potential utility as part of auscultation screening for canine MMVD.

## ABSTRACT C40: Clinical and imaging characteristics of patent ductus arteriosus in standard poodles and their cross‐breeds

### **Sukjung Lim** ^1^; Sonya Wesselowski^2^; Saki Kadotani^3^; Randolph Winter^4^; Ashley Saunders^5^

#### ^1^College of Veterinary Medicine, Texas A&M University; ^2^Assistant Professor, College of Veterinary Medicine, Texas A&M University; ^3^Clinical Assistant Professor, College of Veterinary Medicine, University of Illinois Urbana‐Champaign; ^4^Associate Professor, College of Veterinary Medicine, Auburn University; ^5^Professor of Cardiology I Assistant Department Head for Teaching Presidential Professor of Teaching Excellence, College of Veterinary Medicine, Texas A&M University

**Background:** Standard poodle (SP) cross‐bred (SP‐C) dogs have gained popularity with limited literature representation. Awareness of patent ductus arteriosus (PDA) characteristics in these breeds is needed.

**Objectives:** Report clinical, transesophageal echocardiography (TEE), and procedural data in SP/SP‐C dogs with PDA.

**Animals:** 29 client‐owned SP/SP‐C dogs.

**Methods:** Multi‐institutional medical record review (2010‐2024). Data are reported as median and range.

**Results:** Breeds included SP (13/29), goldendoodle (8/29), labradoodle (6/29), and bernedoodle (2/29). Age and weight at presentation were 0.6 years (0.2‐6.0) and 13.8 kg (3.1‐25.6), respectively. 9/29 had concurrent congenital heart disease; 13/29 required furosemide at presentation. Closure methods included Amplatz canine duct occluder (ACDO) device (26/29) and surgical ligation (3/29). Device size was 7 mm (3‐12). Based on TEE (18/29), PDA morphology was type IIA (9/18), IIB (7/18), and other (2/18). Pulmonary ostium diameter, ampulla diameter 4 mm above ostium, ampulla diameter at aorta level, and ampulla length were 4.2 mm (1.4‐8.1), 9.8 mm (3.4‐13.0), 12.4 mm (2.6‐21.0), and 15.8 mm (8.8‐24.5), respectively. Pulmonary ostium diameter (ostium to ampulla diameter 4 mm ratio x 100) was 41% (35‐66) of the ampulla. Immediately post‐ACDO occlusion, ampulla diameter at device and aorta level were 11.8 mm (3.7‐14.9) and 15.0 mm (3.1‐22.1), a median increase of 20% (3‐60) and 16% (6‐37), respectively. Procedural complications included intra‐operative atrial fibrillation (2/29), device embolization following ampulla dilation with subsequent ligation and mild persistent residual flow (1/29), and post‐operative death following PDA rupture with partial ligation (1/29).

**Conclusions and Clinical Importance:** Large PDAs that enlarge further post‐ACDO occlusion are appreciated in SP/SP‐C dogs, with TEE providing useful anatomic information.

## ABSTRACT C41: Cardiac abnormalities using a simplified diagnostic evaluation in dogs at risk for *Trypanosoma cruzi* infection

### **Kendra Zelachowski** ^1^; Lisa Auckland^1^; Stephanie Collins^2^; Sarah Hamer^1^; Marty Henderson^3^; Nicholas Jeffery^1^; Ashley Saunders^1^

#### ^1^Texas A&M University; ^2^Field Veterinarian; ^3^SonoVet

**Background:** *Trypanosoma cruzi* causes cardiac abnormalities in a subset of infected dogs. Diagnosis is challenging with limited access to advanced diagnostics.

**Objective:** To describe association of abnormalities and serostatus using a simplified diagnostic evaluation.

**Animals:** Forty‐six asymptomatic dogs at risk for *T. cruzi* infection.

**Methods:** Prospective, cross‐sectional study consisting of two antibody tests for *T. cruzi* (indirect fluorescent antibody, TVMDL; Chagas STAT‐PAK validated for humans, Chembio Diagnostics), blood PCR, cardiac troponin I, 30‐second IDEXX CardioPet ECG (with review of automated measurements), and focused echocardiogram (right sided study with 7 variables). Dogs were categorized as seropositive (SP), seronegative (SN), or discordant (SD) based on antibody results. Associations of ordinal data were explored with chi‐square tests.

**Results:** Dogs were SP (19), SN (17), and SD (10) with 9 PCR positive across groups (7 SP, 1 SN, and 1 SD). Serum troponin was above reference range in 6/46 (4 SP, 1 SN, and 1 SD). ECG abnormalities were documented in 10/46 (8 SP, 0 SN, and 2 SD), including conduction abnormalities (prolonged P, PR, QRS durations, second degree atrioventricular block, splintered QRS complex) and ventricular ectopy. Echocardiographic abnormalities were documented in 23/46 (9 SP, 10 SN, and 4 SD) predominately related to left atrial and ventricular enlargement and complicated by degenerative valve disease. Conduction abnormalities and ventricular ectopy were associated with positive serostatus.

**Conclusions and Clinical Importance:** Echocardiographic abnormalities were complicated by concurrent heart disease and did not distinguish between serostatus. ECG is a simple test to perform with abnormalities more common in SP dogs.

## ABSTRACT C42: Evaluation of trazodone on heart rate, heart rate variability and QT intervals in dogs

### **Sandra Tou** ^1^; Vanessa Silvia^2^, DVM; Teresa DeFrancesco^3^ , DVM, DACVECC, DACVIM (Cardiology); Bruce Keene^3^, DVM, MSc, DACVIM (Cardiology)

#### ^1^North Carolina State University, Raleigh, NC, USA; ^2^College of Veterinary Medicine, North Carolina State University, Raleigh, NC, USA; ^3^Professor, Department of Clinical Sciences, College of Veterinary Medicine, North Carolina State University, Raleigh, NC, USA

**Background:** Trazodone is a serotonin receptor antagonist and reuptake inhibitor increasingly used in dogs for anxiolysis.

**Hypothesis/Objectives:** To determine the effect of trazodone on heart rate (HR), heart rate variability (HRV), and QT intervals (QTi) in dogs after oral administration of trazodone or placebo.

**Animals:** Twenty healthy adult client‐owned dogs.

**Methods:** Dogs were randomized in a double‐blinded, placebo‐controlled crossover trial. Dogs received trazodone (6 mg/kg q 8 h) or placebo for 24 hrs during which a 24‐hours ambulatory ECG (Holter) was recorded. Diagnostic ECGs and behavior scores were obtained before and after the 24‐hours study period. Following a minimum 1‐week washout period, dogs received the alternate study drug, and all procedures were repeated. Owners scored the dog's behavior change at home during each of the 24‐hours study periods. Linear and mixed models were used for statistical analyses.

**Results:** Dogs receiving trazodone had higher average HRs (*P* = 0.035), higher minimum HRs (*P* < .001).

**Conclusions and Clinical Importance:** Trazodone increased HR and decreased HRV. Explanations include a previously described anticholinergic effect or a possible decrease in blood pressure causing a HR response. Blood pressure measurement was not performed as part of this study. The lack of demonstrable effect on QTi is reassuring in this cohort of healthy dogs using standard trazodone dosing.

## ABSTRACT C43: Small coronary arterial size is associated with dilated cardiomyopathy in doberman pinschers

### **Samantha Scott** ^1^; Meg Sleeper^2^, VMD, DACVIM (Cardiology); Renee Girens^3^

#### ^1^College of Veterinary Medicine, University of Florida, Gainesville, FL, USA; ^2^University of Florida, Gainesville, FL, USA; ^3^Summit Veterinary Referral Center, Tacoma, WA, USA

**Background:** There is little information on the relationship between coronary artery size and the pathogenesis of dilated cardiomyopathy (DCM) in Doberman Pinschers.

**Hypothesis/Objectives:** To determine if Doberman Pinschers with dilated cardiomyopathy will have smaller coronary artery dimensions relative to their body weight compared to other dogs.

**Animals:** Sixty‐two dogs undergoing interventional procedures with angiography between 2018 and 2023, consisting of Doberman Pinschers with DCM (N = 9), pulmonic stenosis (PS, N = 43), patent ductus arteriosus (PDA, N = 6), Duchenne Muscular Dystrophy (DMD, N = 3), and apparently healthy dogs (N = 1).

**Methods:** A single‐institution retrospective study evaluating the association between coronary artery size and body weight. Measurements of the right coronary artery ostium were obtained from existing angiography and compared with the patient's body weight. The right coronary was chosen due to consistent visibility in angiographic studies.

**Results:** Doberman Pinschers with DCM overall had smaller right coronary artery measurements relative to their body weights compared to other evaluated dogs. Statistics and interobserver variability are currently in progress.

**Conclusions and Clinical Importance:** These results may support that decreased coronary artery size contributes to the pathogenesis of DCM in Doberman Pinschers. Further studies into the clinical significance of this finding are required and may include evaluation of the left coronary arteries and analysis with alternate imaging modalities.

## ABSTRACT C44: Circulating surfactant protein‐B in healthy, stage B2 and acute congestive heart failure (stage C) dogs

### **Catherine J. Georges** ^1^; Ann Chan^2^; Darcy Adin^3^, DACVIM (Cardiology)

#### ^1^Small Animal Hospital, University of Florida, Gainesville, FL, USA; ^2^Manager, Scientific Clinical Laboratory, Department of Research and Graduate Studies, College of Veterinary Medicine, University of Florida, Gainesville, FL, USA; ^3^Clinical Professor of Cardiology, Chief Medical Officer, Small Animal Hospital, University of Florida, Gainesville, FL, USA

**Background:** Cardiogenic pulmonary edema is incompletely explained by increased hydrostatic pressure. Blood‐lung barrier dysfunction, as indicated by circulating surfactant, might contribute.

**Hypothesis:** Dogs with acute heart failure (stage C) have higher circulating surfactant protein‐B (SP‐B) concentrations compared to healthy and stage B2 dogs.

**Animals:** Twenty healthy, 41 stage B2 myxomatous mitral valve disease (MMVD), and 41 acute stage C MMVD dogs.

**Methods:** Prospective observational study. Serum SP‐B was measured using an ELISA kit (LSBio; lower detection 1.56 ng/mL). Lyophilized canine lung was a positive control with Western blot verification. Dogs were fasted for serum collection. Samples from C dogs were collected within 12 hours of admission. Results are expressed as median (range). SP‐B was compared between groups (Kruskal‐Wallis test). Relationships between SP‐B and clinical variables were explored with Spearman's correlation coefficients (rs). Proportion of dogs with detectable SP‐B having daily cough and <1‐month survival was tested with Fisher's exac*t* test.

**Results:** Circulating SP‐B was detected in 9/102 dogs (9%) with no group differences (healthy: 0 (0‐36.8 ng/mL), B2: 0 (0‐32.5 ng/mL), C: 0 (0‐161.9 ng/mL; *P* = .93). The proportion of dogs with detectable SP‐B was not different between coughing and non‐coughing dogs (*P* = .68) or < 1‐month survival (*P* = .12). SP‐B was weakly correlated with total furosemide administered (*P* = .04, rs = 0.3173).

**Conclusions and Clinical Importance:** Circulating SP‐B was detected in a small number of dogs but not more so in acute C dogs. If total furosemide dose relates to disease severity, its weak relationship to SP‐B might indicate barrier disruption in some dogs.

## ABSTRACT C45: Echocardiographic evaluation of pulmonary vascular resistance in dogs with pulmonary hypertension

### **Alba Stavri**; Maya Krasnow, DVM, DACVIM (Neurology); Karsten Schober, DVM, PhD, DECVIM‐CA (Cardiology)

#### The Ohio State University, Columbus, OH, USA

**Background:** Pulmonary vascular resistance (PVR) is an important hemodynamic variable in the development and classification of pulmonary hypertension (PH) and may aid in therapeutic decision‐making. There is a lack of data on PVR in dogs with PH.

**Hypothesis/Objectives:** Echocardiographic estimation of PVR in dogs is feasible and can be used to differentiate between pre‐capillary PH (pre‐PH), isolated post‐capillary PH (Ipc‐PH), and combined post‐ and pre‐capillary PH (Cpc‐PH) and provides valuable hemodynamic information beyond that derived from tricuspid regurgitation (TR) velocity alone.

**Animals:** 459 dogs with PH and 38 control dogs with TR.

**Methods:** Retrospective observational study. Eighteen clinical and 60 echocardiographic variables predicting PH and PVR were evaluated. PVR (pulmonary artery pressure divided by pulmonary flow) was calculated using 3 equations validated in people. Dogs with pre‐PH, Ipc‐PH, and Cpc‐PH were compared using common statistical tests for large group comparisons. A *P* value <.05 was considered significant.

**Results:** Normal values were derived from the control dogs. There were 213 dogs with pre‐PH and 246 dogs with post‐PH. Average PVR was 2.4 ± 0.8 WU, 10.2 ± 6.2 WU, 4.3 ± 1.1 WU, and 9.9 ± 3.4 WU in control dogs and dogs with pre‐PH, Ipc‐PH, and Cpc‐PH, respectively. PVR was disproportionally increased (6.6‐15.4 WU) in some dogs with mild Cpc‐PH (TR velocity 2.89‐3.37 m/s) and normal (<4.7 WU) in some dogs with severe Ipc‐PH (TR velocity 3.95‐4.72 m/s), identifying subpopulations where estimation of PVR could be particularly beneficial.

**Conclusions and Clinical Importance:** Echocardiographic PVR estimation is feasible and clinically useful.

## ABSTRACT C46: The prevalence of mitral valve cleft in dogs with myxomatous mitral valve disease

### **Jeongmin Lee** ^1^; Jiwoong Her^2^, DVM, MS, DACVECC; Young‐Wook Cho^1^, DVM; Youn‐Seo Jung^1^, DVM; Ahra Lee^1^, DVM, PhD; Sun‐tae Lee^1^, DVM, MS; Kyoung‐A Youp^1^, DVM, MS

#### ^1^Korea Animal Medical Center, Chungcheongbuk‐do, Republic of Korea; ^2^Veterinary Medical Center, The Ohio State University, Columbus, OH, USA

**Background:** Mitral valve cleft is identified as the cause of mitral regurgitation in humans, but its prevalence is limited in veterinary medicine.

**Hypothesis/Objectives:** The study aims to determine the prevalence of mitral valve cleft in dogs diagnosed with myxomatous mitral valve disease (MMVD).

**Animals:** Twenty‐one client‐owned dogs with MMVD.

**Methods:** Retrospective observational study. All dogs were evaluated by using three‐dimensional trans‐esophageal echocardiography.

**Results:** Six dogs were in MMVD stage B2, seven in stage C, and eight in stage D. Notably, 13 dogs (prevalence 62%) were diagnosed with mitral valve clefts, primarily located in the mid‐A1 segment (n = 5). Specifically, 6/8 (75%) of dogs in MMVD stage D, 5/7 (71%) in stage C, and 2/6 (33%) in stage B2 exhibited mitral valve clefts. Additionally, indentation was observed in only one dog.

**Conclusions and Clinical Importance:** Mitral valve clefts are frequently seen in dogs with MMVD.

## ABSTRACT C47: Two‐dimensional echocardiographic ratios for assessment of right heart size in dogs

### **Jackie N. Sankisov**; Lance Visser; Kate Davis; June Boon; Evan Ross; Abigail Laws

#### Colorado State University, Fort Collins, CO, USA

**Background:** Reference intervals (RIs) for simple bodyweight‐independent measurements of right atrial (RA), right ventricular (RV) minor chamber dimension, and RV wall thickness (RVWT) are limited.

**Hypothesis/Objectives:** We sought to generate reference intervals for measurements of right heart size indexed to the right parasternal long‐axis aortic diameter (AoD) and the corresponding left heart structure. Additionally, we sought to describe the reproducibility of these measurements.

**Animals:** Ninety healthy adult dogs of varying body size.

**Methods:** Prospective study. All dogs underwent an echocardiogram performed by the same operator using a right parasternal long‐axis 4‐chamber (RPLx) view and a left apical 4‐chamber (Ap4Ch) view. Eight dogs underwent repeated echocardiograms by 3 different operators (intra‐day) and the same operator performed echocardiograms on these 8 dogs on 3 different days. 95% RIs were generated (Clinical Laboratory Standards Institute methodology). Measurement variability was quantitated using coefficients of variation.

**Results:** Reference intervals for RA_RPLx/AoD, RV_RPLx/AoD, and RVWT_RPLx/AoD were 1.08‐1.95, 0.67‐1.57, and 0.25‐0.49, respectively, and for RA_RPLx/left atrium (LA)_RPLx, RV_RPLx/left ventricle (LV)_RPLx, and RVWT_RPLx/left ventricular wall thickness (LVWT)_RPLx were 0.53‐0.95, 0.33‐0.68, and 0.45‐0.78, respectively. Reference intervals for RA_Ap4Ch/AoD and RV_Ap4Ch/AoD were 0.89‐1.90 and 0.83‐0.1.76, respectively, and for RA_Ap4Ch/LA_Ap4Ch, RV_Ap4Ch/LV_Ap4Ch were 0.51‐1.07 and 0.37‐0.76, respectively. The RVWT could not be reliably measured from the Ap4Ch view. All intra‐operator (between‐day) and inter‐operator (intra‐day) coefficients of variation were <16.4%.

**Conclusions and Clinical Importance:** Simple bodyweight‐independent RIs for assessment of right heart size are available for clinical use. Reproducibility appears sufficient for serial evaluations.

acvim24_501.

## ABSTRACT C48: Effects of medetomidine‐vatinoxan on echocardiographic examination in ventricular tachy‐paced dogs with mild heart failure

### **Sydney St. Clair** ^1^; Heta Turunen^2^, DVM, PhD; Nancy Zimmerman^3^, DVM; Steve Roof^1^; Robert Hamlin^1^; William Muir^3^, DVM, MS, PhD, DACVAA, DACVECC

#### ^1^QTest Labs, Dublin, OH, USA; ^2^Expert Scientist, Clinical R&D, Vetcare Oy, Finland; ^3^Global Business Development Director, Dechra Pharmaceuticals PLC; ^4^Professor, Associate Dean, Basic Sciences and Research, Lincoln Memorial University, Harrogate, TN, USA

**Background:** Medetomidine‐vatinoxan combination (Zenalpha) is a novel sedative‐analgesic for dogs causing fewer adverse cardiovascular changes compared to traditional alpha_2_‐agonists. Fractious, fearful, or excited cardiac patients requiring considerable manual restraint to perform echocardiography could benefit from procedural sedation if there was a safe and efficacious sedative option that would not interfere with the interpretation of the results.

**Hypothesis/Objectives:** The objective was to compare the echocardiographic effects of medetomidine‐vatinoxan to dexmedetomidine in dogs with mild heart failure.

**Animals:** Seven purpose‐bred, tachy‐paced beagles with ejection fraction of 49 ± 4% including mild concomitant left‐ventricular enlargement.

**Methods:** In this blinded crossover study each dog received 0.25 mg/m^2^ medetomidine and 5 mg/m^2^ vatinoxan or 0.25 mg/m^2^ dexmedetomidine intramuscularly. A transthoracic echocardiographic examination was performed at baseline and 15, 30, and 45 minutes post‐treatment.

**Results:** End diastolic and systolic volumes were significantly lower at 45 minutes and left ventricular diameter during diastole and systole significantly smaller at each observation point with medetomidine‐vatinoxan in comparison to dexmedetomidine. Ejection fraction and fractional shortening remained significantly higher at 30 and 45 minutes with medetomidine‐vatinoxan than with dexmedetomidine. Left atrial diameter to aortic diameter ratio was significantly lower with medetomidine‐vatinoxan than with dexmedetomidine at 15 minutes. The only significant change compared to baseline with medetomidine‐vatinoxan was decreased left ventricular diameter during diastole at 45 minutes. With dexmedetomidine most variables changed significantly from baseline.

**Conclusions and Clinical Importance:** Medetomidine‐vatinoxan caused less echocardiographic changes in dogs with mild heart failure in comparison to dexmedetomidine, and most variables remained close to baseline values.

## ABSTRACT C49: Prevalence of cardiac disease and population characterization of feline telemedicine cardiology case submissions

### **Grace E. Flynn** ^1^; Sukjung Lim^2^; Matthew Denton^3^; Jenny Applebaum^3^; Sonya Gordon^4^

#### ^1^College of Veterinary Medicine, Texas A&M University, College Station, TX, USA; ^2^Cardiology Research Intern, College of Veterinary Medicine, Texas A&M University, College Station, TX, USA; ^3^Rotating Intern, College of Veterinary Medicine, Texas A&M University, College Station, TX, USA; ^4^Professor of Cardiology, Eugene Ch'en Chair in Cardiology, College of Veterinary Medicine, Texas A&M University, College Station, TX, USA

**Introduction:** Telemedicine facilitates convenient access to board‐certified cardiologists and is widely utilized by veterinarians. Characterization of case submissions can help understand the value of this service.

**Objectives:** Describe a population of feline telemedicine cases with respect to submission indication, demographics, physical examination (PE) and type/stage of heart disease.

**Animals:** Feline telemedicine cardiology cases submitted to Idexx Laboratories that included contemporaneous, bloodwork, ECG, echocardiogram, and thoracic radiographs (Nf400).

**Materials and Methods:** Telemedicine reports were reviewed, and results recorded.

**Results:** Breed was domestic short, medium or long hair in 64.3%, pure‐breed in 32.8% and unidentified in 2.9%. Sex was male in 57.0% and female in 37.8% and unidentified in 5.2%. Median (interquartile range) for weight (kg) and age (year) were 4.7 (3.6‐5.9) and 9.0 (5.9‐13.0), respectively. Primary indication for case submission was pre‐anesthesia work‐up (37.5%), clinical signs (27.5%), PE abnormality (17.3%), re‐evaluation (16.5%), and cardiac screen (1.2%). The prevalence of murmurs was 76.5% overall and 81.0% in cats presented for PE or pre‐anesthesia indications. Prevalence of dysrhythmias (PE or ECG) was 17.8% overall. Gallops were identified in 3.5% of cats. The echocardiographic diagnosis was normal (18.5%), equivocal (7.8%) and abnormal (73.8%). The most common abnormal diagnosis was a cardiomyopathy (94.9%). Represented ACVIM stages of cardiomyopathy included B1 (52.9%), B2 (16.8%), C (30.3%).

**Conclusions:** Cardiomyopathy is very common (73.8%) in this feline telemedicine population. Pure‐breed cats were relatively common, representing a third of the population. Pre‐anesthesia work‐up and clinical signs were the most common indications for consultation. Remote access to expert opinion via telemedicine results in a high prevalence of diagnosis of clinically relevant disease which results in recommendations that can help optimize care.

## ABSTRACT CR01: Echocardiographic features and survival of dogs with constrictive pericarditis secondary to coccidioidomycosis

### **Maria Luz Wang** ^1^; Jared Jaffey^2^, DVM, MS, DACVIM (SAIM); Amanda Liggett^3^, DVM; Whit Church^4^, DVM, DACVIM (Cardiology); Iris Schaitkin^5^; Annalise Cavender^5^

#### ^1^Desert Veterinary Medical Specialists, Gilbert, AZ, USA; ^2^Assistant Professor, Specialty Medicine, College of Veterinary Medicine, Midwestern University, Glendale, AZ, USA; ^3^Cardiology Resident, Cardiology, Desert Veterinary Medical Specialists, Gilbert, AZ, USA; ^4^Director, Cardiology, Desert Veterinary Medical Specialists, Gilbert, AZ, USA; ^5^Veterinary Student, Specialty Medicine, College of Veterinary Medicine, Midwestern University, Glendale, AZ, USA

**Background:** Constrictive pericarditis (CP) secondary to coccidioidomycosis is potentially life‐threatening in dogs with limited information.

**Objectives:** Describe clinically relevant echocardiographic features and survival in dogs with coccidioidomycosis associated CP treated with or without subtotal pericardectomy.

**Animals:** Twenty‐three client‐owned dogs.

**Methods:** Dogs were eligible for inclusion in this retrospective study if they were diagnosed with coccidioidomycosis associated CP with or without pericardial effusion from 2020 to 2023. Diagnosis required echocardiographic features of CP in conjunction with ≥1 of positive anti‐*Coccidioides* spp. antibody serology or identification of organisms on cytological/histopathological examination. Echocardiograms were performed by a board‐certified cardiologist or a resident under supervision. Dogs were treated either with subtotal pericardectomy or medical therapy alone.

**Results:** All dogs had positive IgG titers (median, range; 1:16, 1:1‐1:32) and 10 (43%) had positive IgM results. The mean pericardial thickness measured by echocardiography was 4.3 mm (SD, 2.3). Other important features of CP included the presence of septal bounce (47%, 11/23), accentuation of mitral e’ (86%, 18/21), mitral annulus reversus (28%, 5/18), and hepatic venous distension with flow reversal (70%, 16/23). Fourteen (61%) dogs were treated with medical therapy alone and the remaining 9 dogs (39%) with subtotal pericardectomy. Median follow‐up time was 501.5 days (range, 23‐800 days; n = 22). There was no difference in survival between dogs treated with surgery (median, range; 800 days, 126‐800 days) and without (undefined, 23‐738 days; *P* = .06; log‐rank test).

**Conclusions and Clinical Importance:** Dogs with CP secondary to coccidioidomycosis can have prolonged survival times without surgery.

## ABSTRACT E01: Characterization of equine platelet lysate for nebulization in the horse

### **Patricia Egli** ^1^; Lindsay Boone^2^; Julie Gordon^3^; Laura Huber^4^; Kara Lascola^2^; Maria Naskou^4^; John Peroni^3^

#### ^1^Auburn University, Auburn, AL, USA; ^2^Department of Clinical Sciences, Auburn University, Auburn, AL, USA; ^3^Department of Large Animal Medicine, University of Georgia, Athens, GA, USA; ^4^Department of Pathobiology, Auburn University, Auburn, AL, USA

**Background:** Therapeutic potential of equine platelet lysate (ePL) for treatment of respiratory disease in horses remains unknown.

**Hypotheses/Objectives:** To characterize and compare pre‐ and post‐nebulized ePL according to protein composition and in vitro antimicrobial activity against clinically relevant respiratory pathogens. Differences between pre‐ and post‐nebulized ePL were not expected.

**Animals:** Pooled ePL from 3 healthy horses.

**Methods:** Sterile ePL nebulized condensate was collected via mesh nebulizer. Flow rate and aerosolized particle size distribution were quantified. Pre‐ and post‐nebulized ePL aliquots were compared according to: growth factor, antimicrobial peptide, and cytokine concentrations; proteomic analysis; and bacterial growth inhibition parameters [maximum growth (**μ**); carrying capacity (**K**)] for *Streptococcus equi* subsp. *zooepidemicus* and *Rhodococcus equi* (susceptible and MDR) clinical isolates using pre‐ and post‐nebulized ePL concentrations of 50% (PreN50, PostN50).

**Results:** Flow rate and median particle size were 0.8 ml/min and 4.991 μm with 52% of particles ≤5 μm. Differences in cytokine, growth factor, or proteomic analysis were not identified between pre‐ and post‐nebulization (*P* > .1). Negative effects on **K** were noted for *S. zooepidemicus* (PreN50, *P* = .009; PostN50, *P* = .009)) and *E. coli* (PreN50, *P* = .05) compared to BHI. No effect on condition was observed on **μ** for *S. zooepidemicus* and *E. coli*. For *R. equi*‐MDR and *R. equi* (WT), **K** and **μ** were **positively** affected by all PL treatments (PreN50 and PostN50, *P* ≤ .05).

**Conclusions and Clinical Importance:** Bacterial growth inhibition of ePL is organism dependent, but nebulization does not appear to affect protein composition. Further research is recommended to better characterize ePL antimicrobial activity against clinically relevant pathogens and suitability for nebulization in horses.

## ABSTRACT E02: Multi‐dose preserved lidocaine nebulization does not alter respiratory microbiota or inflammatory markers in healthy horse

### **Lauren C. Holley** ^1^; Daniela Bedenice^1^; Giovanni Widmer^1^; Tyler‐Jane Robins^1^; Victoria Trautwein^1^; Sarah Reed^2^; Melissa Mazan^1^

#### ^1^Cummings School of Veterinary Medicine, Tufts University, North Grafton, MA, USA; ^2^University of Connecticut, Storrs, CT, USA

**Background:** Nebulized 4% preservative‐free lidocaine has been previously shown to reduce airway inflammation in equine asthma (Mahalingam‐Dhingra 2022). However, 2% multi‐use vial, preservative‐containing lidocaine is more accessible, less expensive, and would increase client compliance.

**Hypothesis/Objectives:** Methyl paraben, the preservative in multi‐use vials, may increase morbidity in children with asthma. Therefore, this study aimed to establish multi‐dose safety of 2% preserved lidocaine in horses.

**Animals:** Fourteen clinically healthy, non‐asthmatic horses.

**Methods:** A prospective, randomized, controlled, blinded 2‐way crossover study was performed to compare nebulization of 1 mg/kg 2% preservative‐containing lidocaine via Flexineb twice daily over 4 days, to saline control and no treatment. Clinical parameters, chemokine, cytokine, and bronchoalveolar lavage (BAL) cytology, as well as 16S‐amplicon sequencing of nasal and tracheal microbiota, before and after each intervention were compared between treatment groups, using paired and related samples analyses, based on data normality (*P* < .05).

**Results:** Neither saline nor 2% preservative‐containing lidocaine nebulization induced evidence of airway inflammation based on BAL cytology, chemokine and cytokine concentrations in epithelial lining fluid, or changes in clinical scoring. A mean 5.9% decrease in BAL fluid neutrophil percentage was noted over time without intervention (control). When comparing intervention effects on BAL cytology, only saline nebulization (compared to control) was associated with a statistically (but not clinically) significant difference in percent neutrophil change (7.7%). Treatment did not impact the operational taxonomic units (OTU) profile of respiratory microbiota.

**Conclusions:** No differences in outcomes were found in healthy horses nebulized multi‐use vial, 2% methyl paraben‐containing lidocaine versus saline.

## ABSTRACT E03: An in‐hospital clinical trial assessing nebulized lidocaine compared to saline for treatment of equine asthma

### **Ananya Mahalingam‐Dhingra** ^1^; Daniela Bedenice^1^, DrMedVet, DACVIM (LAIM), DACVECC (Eq); Melissa Larkin^1^, DVM; Melissa Mazan, DVM, DACVIM (LAIM)^1^; Jillian Minuto^2^, DVM DACVIM (LAIM); Tyler‐Jane Robins^1^; Victoria Trautwein^1^

#### ^1^Cummings School of Veterinary Medicine, Tufts University, North Grafton, MA, USA; ^2^Chapparal Veterinary Medical Center, Cave Creek, AZ, USA

**Background:** Corticosteroids are the mainstay of pharmacologic treatment for equine asthma (EA), but may have adverse effects in metabolically unstable horses. Recent pilot studies support the use of nebulized lidocaine as an alternative treatment option.

**Hypothesis:** Nebulized lidocaine will improve clinical parameters and decrease airway inflammation associated with EA.

**Animals:** 20 client‐owned horses diagnosed with EA.

**Methods:** A randomized, blinded, in‐hospital study compared the effects of 1 mg/kg 4% lidocaine (n = 10) nebulized twice daily via Flexineb™ over 4 days to saline control (n = 10). Clinical examination, endoscopy, lung function, bronchoalveolar lavage cytology, and inflammatory markers in blood and epithelial lining fluid (ELF) were compared between treatment groups using univariate analyses, based on data normality.

**Results:** Neither lidocaine nor saline nebulization resulted in significant improvement of any of the assessment parameters between day 0 and 4. Comparing effects between treatment groups, horses undergoing saline nebulization showed a significantly greater decrease in ELF interferon‐γ (*P* = .015) and tissue necrosis factor‐α (*P* = .034) compared to lidocaine treated horses.

**Conclusions and Clinical Importance:** Nebulized lidocaine did not show efficacy as an alternative treatment for EA in this study, but a longer duration of treatment may be necessary. The significant changes in cytokine levels may help elucidate the immunology of EA.

## ABSTRACT E04: Effect of steamed hay on horses with severe equine asthma in remission

### **Clara Raisky** ^1^; Berta Mozo Vives^2^, DVM; Laurence Leduc^1^, DVM, MS, DACVIM (LAIM); Antoine Symoens^1^, DVM; Tristan Juette^1^, PhD; Mathilde Leclère^1^, DVM, PhD, DACVIM (LAIM)

#### ^1^Université de Montréal, Saint‐Hyacinthe, QC, Canada; ^2^Resident, Université de Montréal, Saint‐Hyacinthe, QC, Canada

**Background:** Steaming hay reduces respirable particles and is commonly used to feed horses with asthma. However, it showed inconsistent benefits in clinical studies.

**Hypothesis/Objectives:** We hypothesized that horses with severe equine asthma (SEA) would develop airway obstruction when fed dry hay but not steamed hay.

**Animals:** Nine horses with SEA from a research herd, in remission on a pelleted diet.

**Methods:** Horses were fed steamed and dry hay for 4 weeks in a prospective, cross‐over study. Lung function (impulse oscillometry) and blinded weighted clinical scores (WCS) were recorded before and after 4 weeks of hay feeding. A linear mixed model with post‐hoc tests was used.

**Results:** Resistance at 5 hertz (R5) increased over the 4‐week period (time effect and post‐hoc End versus Baseline: *P* < .001), with no difference between treatments (mean (SD), kPa/L/s) (Baseline dry: 0.065 (0.014); End dry: 0.079 (0.019); Baseline steamed: 0.063 (0.009); End steamed: 0.078 (0.014)). There was a treatment‐sequence interaction (*P* < .001) with higher R5 during the second treatment. WCS did not change significantly (median (range)) (Baseline dry: 2 (1‐5); End dry: 2 (1‐4); Baseline steamed: 3 (1‐6); End steamed: 1 (1‐4), time and treatment effects *P* > .1).

**CONCLUSION AND CLINICAL IMPORTANCE:** Steamed hay induced a mild but significant deterioration of lung function in horses with SEA. The lack of differences with dry hay could be due to the unexpectedly mild exacerbation during this study. The treatment‐sequence interaction suggests a cumulative effect of stabling and hay feeding on airway resistance.

## ABSTRACT E05: Antimicrobial susceptibility results of *Streptococcus equi* subsp. *equi* isolates from horses

### **Florence Dupuis‐Dowd** ^1^; Camilo Jaramillo‐Morales^2^; Lucas Thacker^3^; K. Gary Magdesian^4^

#### ^1^Large Animal Clinic, University of California‐Davis, Davis, CA, USA; ^2^Staff Veterinarian, Large Animal Clinic, University of California‐Davis, Davis, CA, USA; ^3^Student, Veterinary Medical Teaching Hospital, University of California–Davis, Davis, CA, USA; ^4^Associate Professor, University of California–Davis, Davis, CA, USA

**Background:** Strangles is a highly infectious disease, endemic throughout the world. There is a paucity of reported data on antimicrobial susceptibility in clinical isolates.

**Objectives:** To describe the antimicrobial susceptibility results of *Streptococcus equi* subsp. *equi* (*S. equi*) isolated from horses at a referral center. Animals: 45 horses that were diagnosed with *S. equi* infection in a referral hospital between 2015 and 2023 that had positive cultures and antimicrobial susceptibility testing.

**Methods:** Retrospective, observational study. Data were collected from the medical records: signalment, clinical signs, sampling site, and MIC to a panel of antimicrobials were recorded.

**Results:** 45 horses met the inclusion criteria, with 46 different isolates of *S. equi*. The median age was 9.78 (0.1‐25) years. There were 64% males and 36% females. The most common breeds were Quarter Horse (47%) and Thoroughbred (13%). 65% of samples were obtained from the guttural pouches; 17.4% were swabs from submandibular abscesses, and 17.4% were from other sites, including other abscesses and sinus aspirate. 47% of the horses had received antimicrobial treatment before sampling. 100% of tested isolates were susceptible to ceftiofur (n = 46), doxycycline (n = 45), minocycline (n = 33), and trimethoprim sulfamethoxazole (n = 46). 97.8% (n = 46) of tested isolates were susceptible to penicillin, ampicillin, and rifampin. 97.6% and 97.0% of tested isolates were susceptible to azithromycin and clarithromycin, respectively.

**Conclusions and Clinical Importance:** This study provides information on the antimicrobial susceptibility of *S. equi* isolates from clinical equine cases. *S. equi* isolates remain highly susceptible to β‐lactams, minocycline, doxycycline, rifampin, potentiated sulfonamides, azithromycin, and clarithromycin.

## ABSTRACT E06: Androgen administration in severe equine asthma: A blinded, cross‐over, randomized study

### **Camille Ruault**; Sophie Mainguy Seers, DMV, DACVIM, PhD; Laurence Leduc, DMV,DACVIM; Jean‐Pierre Lavoie, DMV, DACVIM; Tristan Juette

#### Université de Montréal, Saint‐Hyacinthe, QC, Canada

**Background:** Low testosterone levels are associated with poor lung function in human asthmatics, and conversely, androgen administration improves airway obstruction in asthmatic women.

**Hypothesis:** Androgens improve lung function in horses with severe asthma (SEA).

**Animals:** Ten horses with SEA from a research herd.

**Methods:** The effects of oral dexamethasone (0.06 mg/kg, q 24 h, 10 days) and intramuscular testosterone cypionate (single dose, 0.35 mg/kg) were compared in a randomized, blinded, cross‐over study (2 weeks washout). Lung function was assessed by standard lung mechanics at baseline (day [D] 0), D5, and D10. The cytology of bronchoalveolar lavage was evaluated at baseline and D10. Data was analyzed using mixed linear models.

**Results:** The lung function improved over time (*P* < .001) with no difference between groups. There was a significant interaction between the treatment group and time as dexamethasone produced a larger reduction of resistance (*P* < .001; median (IQR) of 2.8 cm H_2_O/L/s (2.1‐3.5) at D0 and 0.6 (0.5‐0.8) at D10 for dexamethasone, and of 2.5 (2.1‐3.1) at D0 and 2.3 (1.4‐3.1) at D10 for testosterone) and elastance (*P* < .04; 5.1 cm H_2_O/L (3.1‐5.6) at D0 and 0.5 (0.4‐0.8) at D10 for dexamethasone, and 4.5 (1.7‐6.8) at D0 and 3.0 (1.2‐3.7) at D10 for testosterone). Neither dexamethasone nor testosterone modified airway neutrophilia. The treatment order had no impact on measured parameters.

**Conclusions and Clinical Importance:** Testosterone was not as effective as dexamethasone in relieving airway obstruction in SEA, but the mechanisms leading to lung function improvement with androgens require further investigation.

## ABSTRACT E07: Pharmacokinetics of chloramphenicol in horses following rectal and nasogastric administration

### **Brittnee A. Sayler** ^1^; Jennifer Davis^2^, DVM, PhD, DAVIM (LAIM), DACVCP; Lyndi Gilliam^3^, DVM, PhD, DACVIM (LAIM); A.J. Manship^4^, DVM, DAVIM (LAIM); Jared Taylor^5^, DVM, MPB, PhD, DACVPM

#### ^1^Oklahoma State University, Stillwater, OK, USA; ^2^Associate Professor, Biomedical Sciences and Pathobiology, Virginia‐Maryland College of Veterinary Medicine, Virginia Tech, Blacksburg, VA, USA; ^3^Professor Equine Internal Medicine, Clinical Sciences, Oklahoma State University, Stillwater, OK, USA; ^4^Assistant Clinical Professor Equine Internal Medicine, Clinical Science, Oklahoma State University, Stillwater, OK, USA; ^5^Associate Professor, Veterinary Pathobiology, Oklahoma State University, Stillwater, OK, USA

**Background:** Chloramphenicol is a broad‐spectrum antibiotic used in equine practice, but hyporexia/anorexia may occur with oral administration. Administration per rectum (PR) could decrease appetite suppression seen with oral use and allow its use in horses unable to receive oral medications while altering hepatic metabolism ratios. The pharmacokinetics of chloramphenicol per rectum have not been studied in horses.

**Hypothesis/Objectives:** The objectives of this study were to evaluate the relative bioavailability of chloramphenicol administered via nasogastric tube (NGT) versus PR and determine relevant pharmacokinetic parameters and metabolic ratios.

**Animals:** Ten healthy, adult horses from the university teaching herd.

**Methods:** All horses received chloramphenicol tablets (50 mg/kg) dissolved in water either via NGT, or PR via red rubber catheter, using a 2‐way, randomized crossover design with 14‐day washout period. Blood samples were collected at predetermined times over 24 hours and plasma concentrations of chloramphenicol and metabolite were analyzed by UPLC‐MS/MS and noncompartmental pharmacokinetics.

**Results:** Chloramphenicol was rapidly metabolized to inactive chloramphenicol glucuronide following both routes of administration. Administration PR resulted in a relative bioavailability of 0.56 ± 0.86% with a maximum concentration (Cmax; μg/mL) of 0.119 ± 0.135 versus 11.7 ± 5.8 with NGT administration. The metabolic ratio of chloramphenicol glucuronide: chloramphenicol was 20.2 ± 6.19 for PR and 5 ± 1.88 for NGT.

**Conclusions and Clinical Importance:** Results of this study show PR administration of chloramphenicol at 50 mg/kg does not reach therapeutic concentrations in horses and does not slow hepatic metabolism. Administration via NGT produced total chloramphenicol concentrations >2 μg/mL for 3.93 ± 0.44 hrs.

## ABSTRACT E08: Pharmacokinetics and safety of oral fluralaner in healthy horses

### **Mallory Lehman**; Samantha Gentille; Lauren Maas; Jennifer Cassano; Heather Knych; Stephen White; Jessica Morgan

#### University of California‐Davis, Davis, CA, USA

**Background:** Ectoparasites including ticks, mites, and lice represent a significant source of irritation and dermatologic disease in the horse. Fluralaner, a long‐acting ectoparasiticide in the isoxazoline class, is currently used in small animals, but the absorption and safety are not well established in horses.

**Hypothesis/Objectives:** The objective of this study was to describe the pharmacokinetics of fluralaner in horses when administered orally.

**Animals:** Six healthy adult horses from a university teaching herd (3 mares, 3 geldings).

**Methods:** Fasted horses received a single dose of 10 mg/kg oral fluralaner and were followed for 84 days. Plasma was sampled at 20 predetermined time points. Skin biopsies from the dorsal metacarpal region were collected 1 day after oral administration. Fluralaner concentrations were measured by LC‐MS/MS. Neurologic examinations, complete blood counts, and serum biochemistries were performed at predetermined time points. Non‐compartmental analysis was used to determine pharmacokinetic parameters.

**Results:** The maximum concentration of fluralaner was 162.1 ± 21.6 ng/ml at 0.42 ± 0.14 days. The terminal half‐life was 6.33 ± 4.13 days. Area under the curve was 485.1 ± 151.6 day*ng/ml. Plasma fluralaner was detectable for 21‐48 days, and in one horse up to 70 days. Skin concentrations were 0.85 ± 0.77 ng/mL at 1 day post administration. No adverse neurologic events were noted throughout the 3 month monitoring period and there were no clinically relevant alterations in blood parameters.

**Conclusions and Clinical Importance:** Oral fluralaner was well tolerated at 10 mg/kg in healthy adult horses. Plasma half‐life in horses is shorter than that reported in dogs at similar doses.

cvim24_511

## ABSTRACT E09: Vitamin E concentration in horses following hospitalization

### **Megan Palmisano** ^1^; Sarah Colmer^1^, VMD, DACVIM; Yih Ling Saw^2^; Xin Xu^2^; Darko Stefanovski^1^, MS, PhD; Lisa Murphy^1^, VMD, DABT; Amy Johnson^1^, DVM, DACVIM (LAIM & Neuro)

#### ^1^New Bolton Center, University of Pennsylvania, Kennet Square, PA, USA; ^2^University of Pennsylvania, Philadelphia, PA, USA

**Background:** Vitamin E is an essential micronutrient, protective against oxidative damage. Vitamin E deficiency is linked to development of neuromuscular disorders in horses. Deficiency is observed in humans hospitalized for critical illness, potentially due to decreased intake or increased oxidative damage.

**Hypothesis/Objectives:** Serum vitamin E concentration [vitE] will decrease from time of admission to discharge, with [vitE] associated with duration of hospitalization.

**Animals:** Client‐owned horses admitted as patients or companions through the emergency service at a tertiary referral center were enrolled. 40 adult horses and 20 foals met inclusion criteria (hospitalized for ≥5 days, no vitamin E supplementation, age ≤ 3 months or ≥ 1 year).

**Methods:** A cohort study was performed. Whole blood was drawn at the time of admission and discharge. Serum [vitE] was obtained using high‐performance liquid chromatography. The data were non‐normally distributed and subsequently analyzed with Spearman rank test with p of <.5 for significance.

**Results:** Duration of hospitalization had no significant effect on [vitE] (*P* = .85) when accounting for all age patients. Mean [vitE] did vary between adults (3.6 ppm), and foals (4.8 ppm) (*P* = .1). When comparing timepoints this difference in vitamin E concentration in adults and foals was maintained for both [vitE]_admit_ and [vitE]_discharge_. In foals, IgG concentration was inversely correlated with [vitE]_admit_ (*P* = .003). Seven adult horses and 2 foals (15% of study population) were considered deficient at admission.

**Conclusions and Clinical Importance:** The average horse admitted to the hospital does not require vitamin E supplementation to maintain adequate [vitE].

**Vitamin E concentration (ppm)**

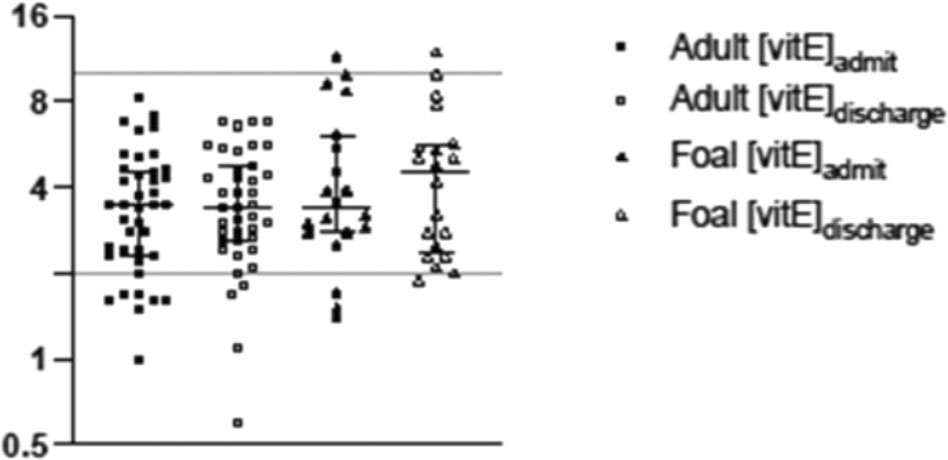

## ABSTRACT E10: Characterization of renal lipidosis in equids: A postmortem case‐control study (2008‐2022)

### **Kali K. Slavik** ^1^; Rose Nolen‐Walston^2^, DVM, DACVIM (LAIM); Susan Bender^3^, VMD, PhD, MS, DACVP; Leslie Sharkey^4^, DVM, PhD, DACVP (Clinical Pathology)

#### ^1^New Bolton Center, University of Pennsylvania, Kennet Square, PA, USA; ^2^Associate Professor, Large Animal Internal Medicine, New Bolton Center, University of Pennsylvania, Kennet Square, PA, USA; ^3^Assistant Professor of Clinical Pathobiology, New Bolton Center, University of Pennsylvania, Kennet Square, PA, USA; ^4^Professor, Department of Clinical Sciences, Tufts University, North Grafton, MA, USA

**Background:** Although reported in other species, findings associated with renal lipidosis have not been previously described in horses and donkeys.

**Objective:** To describe the signalment, clinicopathologic indices, and postmortem findings of equids with histologic diagnosis of both hepatic lipidosis and renal lipidosis (HL + RL) and compare to matched cases with hepatic lipidosis only (HL).

**Animals:** Twenty‐five equids with findings of renal and/or hepatic lipidosis at necropsy from a state diagnostic laboratory between 2008 and 2022.

**Methods:** Retrospective case‐control study. Signalment, history, affected system, and selected biochemical data were extracted from medical records. Each case of HL + RL was assigned a matched control from group HL for comparison of selected clinical data.

**Results:** Renal lipidosis was diagnosed in 0.55% of equid necropsies. Donkeys with hepatic lipidosis were more likely to also have renal lipidosis (7/13, 54%) compared to horses and ponies (18/197, 9%; *P* < .005). No cases of renal lipidosis were identified without concurrent hepatic lipidosis. Renal lipidosis cases most commonly presented with gastrointestinal (61%, 16/25) or neurologic (46%, 12/25) disease. Group HL + RL had a higher median intake plasma lactate (+6.2 mmol/L, IQR 0.4‐10.5; *P* = .04) and higher GGT activity (+246 U/L, IQR 72‐360; *P* = 0.02) compared to group HL controls. No significant differences between groups were noted in creatinine or triglyceride concentration.

**Conclusions:** Renal lipidosis is an occasional postmortem finding in equids with hepatic lipidosis, but is markedly more common in donkeys. Cases with renal lipidosis were not more likely to be azotemic than those with only hepatic lipidosis. The clinical significance of renal lipidosis is unknown.

## ABSTRACT E11: Decline of maternal antibody and natural flavivirus infection in foals

### **Xueli Wang**; Joanne Meers; Allison Stewart; Jessica Wise

#### University of Queensland, Laidley Heights, QLD, Australia

**Background:** Pathogenic Flaviviridae affecting Australian horses include Murray Valley encephalitis, Japanese encephalitis, and Kunjin strain of West Nile viruses.

Hypothesis/**OBJECTIVE:** Maternally derived Flavivirus antibodies in foals will be present at 24 hours of age but will have declined by 4 months of age. Most foals will be naturally exposed to Flaviviruses within their first year of life.

**Animals:** 23 mare/foal pairs from a teaching herd.

**Methods:** A pan‐Flavivirus blocking ELISA was performed on serum obtained from: (a) mares 2 months prior to foaling and when their foals were 24 hours and 16 weeks of age; (b) foals at birth (pre‐suckle), and then at 24 hours, 1, 2, and 3 weeks, then 1, 2, 3, 4, 5, 6, 7, 8, 9, 10, 11, 12, and 13 months of age. The lab defined >40% inhibition as positive. The percentage of mares and foals with positive Flavivirus results at each time point was calculated.

**Results:** 95.6% of mares had positive pan‐Flavivirus ELISAs. All foals were negative on pre‐suckle samples, while 18/23 (78%) were positive for Flavivirus maternal antibodies at 24 hours of age. Maternal antibody persisted in 9/23 (40%) foals at 4 months of age. Natural infection occurred in 7/23 (30.4%) foals with positive results occurring as a second peak after decline of maternal antibody.

**Conclusions and Clinical Importance:** In endemic areas, persistence of maternal antibody may confuse the diagnosis of Flavivirus infection in foals. This study occurred during drought conditions resulting in reduced infection rates in foals.

## ABSTRACT E12: Diagnosis of bacteremia in neonatal foals using high‐throughput sequencing: A pilot study

### **Flavie Payette** ^1^; Alicia Long^2^, DVM, DACVIM, DACVECC; Weiming Hu^3^, PhD; Kyle Bittinger^3^, PhD; Michelle Abraham^2^, BSc, BVMS, DACVIM; Maia Aitken^2^, BA, DVM, DACVS, DACVECC

#### ^1^New Bolton Center, University of Pennsylvania, Kennet Square, PA, USA; ^2^Department of Clinical Studies, New Bolton Center, University of Pennsylvania, Kennet Square, PA, USA; ^3^Division of Gastroenterology, Hepatology, and Nutrition, Children's Hospital of Philadelphia, Philadelphia, PA, USA

**Background:** Neonatal septicemia is an important cause of morbidity and mortality in foals, requiring rapid identification and initiation of treatment for a favorable outcome. Diagnosis is, however, complicated by non‐specific clinical signs and delayed confirmation of infection via blood culture. Molecular assays represent an attractive diagnostic complement by being rapid and more sensitive.

**Objectives:** To evaluate and compare bacterial load and composition in blood and blood culture media of sick and healthy foals, and to compare results with bacterial culture.

**Animals:** 13 septic hospitalized foals, 10 sick non‐septic, and 8 healthy foals.

**Methods:** Prospective pilot study. Bacterial load and composition from whole blood and from blood culture media pre‐enriched for 5 hours and 24 hours were analyzed by quantitative PCR and sequencing of the universal bacterial 16S rRNA marker gene.

**Results:** Whole blood yielded more positive samples (25/31) than blood culture media (6/62; *P* < .0001), but 16S qPCR had low yield overall. Positive blood culture and whole blood sequencing were comparable in only 3/12 foals. The three most abundant genera on whole blood sequencing included *Paucibacter* (mean relative abundance 43.77%), *Staphylococcus* (10.21%), and *Actinobacillus* (7.12%). There were no statistically significant differences between the three groups of foals for alpha diversity, absolute DNA concentration, or sequencing read counts.

**Conclusion and Clinical Relevance:** High‐throughput sequencing represents an interesting avenue for the diagnosis of bacteremia in foals but cannot replace blood culture at this time. Further research to optimize detection yield and sensitivity on whole blood samples is warranted.

## ABSTRACT E14: Effects of two different pioglitazone dosage regimens on insulin dynamics in severely insulin dysregulated equids

### **Caitrin R. Lowndes** ^1^; Ashley Boyle^2^, BA, DVM, DACVIM; Jeaneen Kulp^2^; Andrew Van Eps^2^, BVSc, PhD, DACVIM

#### ^1^New Bolton Center, University of Pennsylvania, Kennet Square, PA, USA; ^2^Department of Clinical Studies, New Bolton Center, University of Pennsylvania, Kennet Square, PA, USA

**Background:** Pioglitazone shows potential for improving insulin dynamics in horses, but longer‐term clinical evaluation is necessary.

**Hypothesis/Objectives:** To characterize the effects of 2 doses of pioglitazone on insulin dynamics in equids with severe insulin dysregulation (ID) over an extended study period.

**Animals:** 17 client‐owned equids with severe ID (resting insulin >100 μIU/mL).

**Methods:** Prospective cohort study. Equids received 2 mg/kg pioglitazone orally either sid (PIO‐SID) or bid (PIO‐BID). Eight resting blood samples over a 70 day study period were analyzed for insulin, high molecular weight adiponectin, and pioglitazone concentration. Oral sugar tests (OST) were performed on days 0 and 28. Data were analyzed using linear regression and t‐tests.

**Results:** Basal serum insulin did not change significantly over time for PIO‐SID (*P* = .49) or PIO‐BID (*P* = .36). Mean [95% CI] post OST insulin (μIU/mL) did not change significantly (day 0 vs. 28) for PIO‐SID (249 [74‐424] vs. 517 [38‐997]; *P* = .4) or PIO‐BID (265 [157‐372] vs. 165 [34‐297] *P* = .2). Adiponectin (day 0 vs. 70, μg/mL) increased in PIO‐SID (0.7 [0.2‐1.3] vs. 4 [−3‐11.1]; *P* = .01) and PIO‐BID (3.5 [−0.3‐7.2] vs. 15 [6.9‐23]).

**Conclusions and Clinical Importance:** Pioglitazone did not significantly improve insulin dynamics; however, there was a marked increase in adiponectin particularly with twice daily administration: a potentially beneficial effect independent of insulin control. Plasma pioglitazone concentrations only reached the low end of the human therapeutic range with twice daily administration.

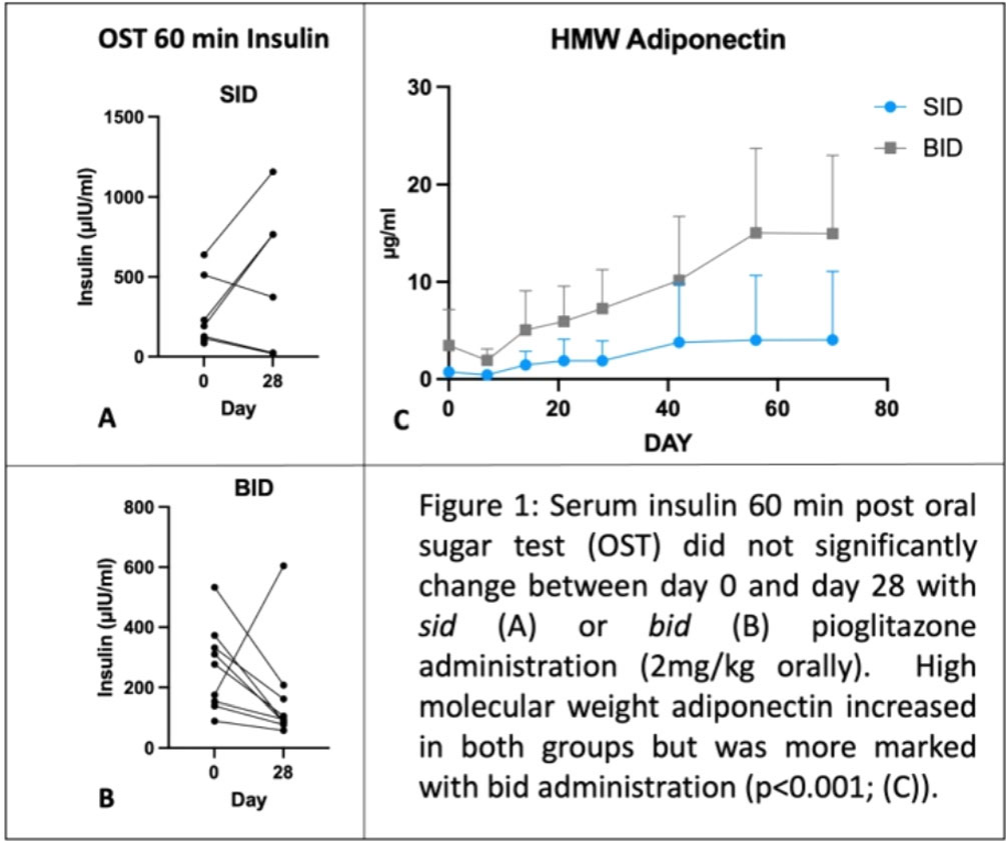

## ABSTRACT E15: Metabolomic alterations associated with allergic response in equine insect bite hypersensitivity

### **Akos Kenez** ^1^; Ezequiel Jorge‐Smeding^1^; Yun Young Go^2^; Johanna Sonntag^3^; Tobias Warnken^3^

#### ^1^Jockey Club College of Veterinary Medicine and Life Sciences, City University of Hong Kong, Kowloon, Hong Kong; ^2^College of Veterinary Medicine, Konkuk University, Seoul, South Korea; ^3^Boehringer Ingelheim Vetmedica GmbH, Germany

**Background:** Insect bite hypersensitivity (IBH) in horses involves an inappropriate immune response against mosquito‐derived antigens; however, the exact cellular pathophysiological mechanisms are unclear.

**Hypothesis/Objectives:** The cells affected by the immune response might leave specific biochemical fingerprints behind in the circulation, as they engage with this immune response. This study aimed to explore the serum metabolomic profiles of horses acutely suffering from an IBH reaction.

**Animals:** Thirty Icelandic horses classified as either healthy non‐IBH controls or IBH cases.

**Methods:** Blood serum was collected in the summer from the healthy controls (n = 15) and once in the summer (acute reaction, n = 15) and once in the winter (latent phase, n = 15) from the IBH cases. Samples were analyzed by untargeted chemical isotope labelling liquid chromatography‐mass spectrometry and the obtained metabolomic profiles were subjected to partial least squares‐discriminant analysis, t‐tests, and pathway enrichment analysis.

**Results:** The metabolomic profiles clustered according to the clinical classification, that is, healthy, acute, and latent. A total of 47 and 104 metabolites had a different abundance between the healthy and the acutely affected, and the acutely affected and latent IBH horses, respectively (FDR‐adjusted *P* < .05). The former included 10 AMino acids and derivatives, 8 peptides, and 10 fatty acids and derivatives, which were linked to 9 significantly altered metabolic pathways (P < .05).

**Conclusions and Clinical Importance:** IBH was associated with changes in the serum metabolomic profile. The metabolic pathway alterations can help explain the pathogenesis of IBH. The affected serum metabolites should undergo further validation for biomarker identification.

## ABSTRACT E16: Biomarkers of brain injury in foals with neonatal maladjustment syndrome

### **Javier Perez Quesada** ^1^; Nimet Browne^2^; Kinnidy Coley^1^; Katarzyna Dembek^1^, DVM, DACVIM(LAIM), PhD; David Wong^3^

#### ^1^North Carolina State University, Raleigh, NC, USA; ^2^Hagyard Equine Hospital, Lexington, KY, USA; ^3^Iowa State University, Ames, IA, USA

**Background:** Neonatal maladjustment syndrome (NMS) is a common disease of foals resulting in neurological dysfunction and increased mortality. Plasma biomarkers of brain injury, such as brain‐derived neurotrophic factor (BDNF), glial‐fibrillary‐acidic protein(GFAP), and astrocytic‐protein‐S100B may be used for diagnosis and monitoring of foals with NMS.

**Objectives:** To measure plasma concentration of biomarkers of neurological damage (BDNF, GFAP, and S100B) in foals with NMS, foals presented for other diseases (sick‐foals), and healthy foals, and determine their association with outcome.

**Animals:** 8 healthy foals, 10 NMS foals, 16 sick‐foals hospitalized for other diseases (eg, diarrhea) <7 days of age. Of the NMS and sick‐foals, 20 survived and 6 did not.

**Methods:** Biomarker concentrations were determined in all foals on admission and at 24, 48, and 72 hours of hospitalization in this prospective, longitudinal study. Plasma concentration of biomarkers was measured with ELISA and single‐molecule‐array technology. Data were analyzed with parametric methods.

**Results:** GFAP concentration was decreased in NMS and sick‐foals compared to healthy foals at time 0 (0.9 ± 0.52, 0.6 ± 0.21, 3.7 ± 1.2 ng/mL), 24 hours (0.84 ± 0.22, 0.73 ± 0.25, 3.6 ± 1.03), and 48 hours (0.5 ± 0.1, 0.56 ± 0.12, 3.1 ± 0.8) (*P* < 0.05), respectively. Non‐survivors had a decreased concentration of GFAP (0.4 ± 0.12 ng/mL) compared to healthy foals (3.7 ± 1.2) and survivors(1.2 ± 0.21) over the first 24 hours of hospitalization (*P* < .05). BDNF and S100B concentrations were not different between groups of foals or time points (*P* > .05).

**Conclusions:** Reduced GFAP concentration in NMS and sick‐foals suggests astroglial dysfunction or delayed postnatal astrogliogenesis. GFAP may be used as a prognosticating factor in critically ill foals.

## ABSTRACT E17: A high‐protein meal is associated with increased glucose‐dependent insulinotropic polypeptide secretion in insulin‐dysregulated horses

### **Allison T. Palmer** ^1^; Mauria Watts^1^; Kathryn Timko^1^, DVM, MS, DACVIM; Erin Pinnell^2^, DVM, MS, DACVIM‐LA; Katelyn Keefer^1^; Laura Hostnik^1^, DVM, MS, DACVIM; Teresa Burns^1^, DVM, PhD, DACVIM (LAIM)

#### ^1^College of Veterinary Medicine, The Ohio State University, Columbus, OH, USA; ^2^College of Veterinary Medicine, Washington State University, Pullman, WA, USA

**Background:** The incretin hormones glucose‐dependent insulinotropic polypeptide (GIP) and glucagon‐like peptide 1 (GLP‐1) augment post‐prandial insulin secretion. Managing equine insulin dysregulation (ID) often involves feeding high‐protein ration balancers. Recent studies suggest that dietary amino acids can promote GIP secretion, enhancing post‐prandial [insulin]; this may increase the risk of hyperinsulinemia following consumption of high‐protein meals in horses with ID.

**Hypothesis:** Consumption of high‐protein meals will increase post‐prandial [GIP] and [GLP‐1] in horses with experimentally‐induced ID.

**Animals:** Adult light‐breed horses with normal [ACTH] (n = 7).

**Methods:** Each horse underwent a frequently‐sampled insulin‐modified IV glucose tolerance test to characterize systemic insulin/glucose dynamics and a feed challenge test (FCT; 1 kg ration balancer [min 32% CP, max 13% NSC] consumed within 15 minutes, [GIP] and [GLP‐1] measured 0‐240 minutes afterward). Both tests were repeated after induction of ID (dexamethasone, 0.08 mg/kg PO SID, 7 days). Outcomes, including [GIP] and [GLP‐1] during the FCT, were compared between baseline and ID.

**Results:** [GIP] and [insulin] increased after a high‐protein meal; ID AUC‐GIP (1166 ± 363 pg/mL) was significantly higher than baseline AUC‐GIP (767 ± 199 pg/mL; *P* = .014). There was no difference in AUC‐GLP‐1 between baseline (50.7 ± 16.6 pg/mL) and ID (39.1 ± 25.3 pg/mL; *P* = .47).

**Conclusions and Clinical Importance:** Horses with experimentally‐induced ID displayed significantly greater GIP responses to a high‐protein meal than at baseline, suggesting that GIP plays a role exacerbating post‐prandial hyperinsulinemia in this context.

## ABSTRACT E18: Assessment of cardiovascular structural changes and non‐invasive blood pressure in warmblood horses with insulin dysregulation

### **Megan Palmisano** ^1^; Andrew Van Eps^1^, BVSc, DACVIM, PhD; Jeaneen Kulp^2^; Darko Stefanovski^2^, MS, PhD; JoAnn Slack^1^, DVM, MS, DACVIM

#### ^1^New Bolton Center, University of Pennsylvania, Kennet Square, PA, USA; ^2^University of Pennsylvania, Philadelphia, PA, USA

**Background:** Insulin dysregulation (ID) is correlated with cardiac disease in humans and myocardial hypertrophy in ponies.

**Hypothesis/Objectives:** The presence of ID will be associated with cardiac hypertrophy and hypertension in a population of Warmblood horses.

**Animals:** Nineteen client‐owned Warmblood horses 5‐15 years of age were included. Horses presented to the hospital for elective procedures were considered eligible. Patients with a history of thyroid supplementation, valvular or congenital cardiac disease were excluded.

**Methods:** The study utilized a prospective cohort design. Horses were classified as ID positive (+) or ID negative (−) based on results of an oral sugar test. Lateromedial front foot radiographs, non‐invasive blood pressure (NIBP) measurements, and focused left ventricular (LV) M‐mode echocardiographic assessment and measurements were performed, with calculation of mean wall thickness (MWT), relative wall thickness (RWT), LV mass (LVM), and intraventricular septal thickness in systole (IVSs) and diastole (IVSd). Data were analyzed using linear regression with statistical significance set at *P* < .05.

**Results:** Eight of nineteen horses were ID(+), 2 of which had radiographic evidence of laminitis. Mean, systolic, and diastolic NIBP were not associated with ID or echocardiographic variables. ID was associated with IVSd (*P* = .023), MWT (*P* = .036), and LVM (*P* = .033). IVSs >4.9 mm was a perfect predictor for ID.

**Conclusions and Clinical Importance:** ID was associated with cardiac hypertrophy in Warmblood horses, particularly increases in IVS thickness. Although ID was not associated with hypertension, a larger sample size is needed to examine this and any associations with laminitis.

## ABSTRACT E19: Novel fibrosis scoring and automated histopathology offer a one health approach to equine adipose tissue abnormalities

### **Emma Stapley** ^1^; Alexandra Burton^2^, BSc, BVSc, PhD, MRCVS, DACVIM (LAIM); Alycia Kowalski^3^

#### ^1^University of Wisconsin‐Madison, Madison, WI, USA; ^2^Clinical Assistant Professor, School of Veterinary Medicine, University of Wisconsin‐Madison, Madison, WI, USA; ^3^School of Veterinary Medicine, University of Wisconsin‐Madison, Madison, WI, USA

**Background:** Fibrosis of adipose tissue and adipocyte hypertrophy are key histopathologic features of obesity in human metabolic syndrome. Multiple tools have been validated in human medicine to assess these changes. Characterization of obesity‐associated structural changes in equine adipose tissue is limited, particularly in the metabolically active nuchal ligament deposit.

**Hypothesis/Objectives:** To assess the utility and repeatability of a validated human adipose fibrosis scoring system (FAT) and automated adipocyte measurement software (Adiposoft) for evaluation of equine nuchal ligament adipose tissue. Both degree of fibrosis and adipocyte size are expected to be greater in obese vs. lean horses.

**Animals:** Ten university‐owned mares.

**Methods:** Nuchal ligament adipose punch biopsies were obtained and assigned equine fibrosis of adipose tissue (eFAT) scores based on the established human scoring system. Adipocyte diameter was measured manually and using Adiposoft with and without manual corrections; these were compared using a Kruskal‐Wallis test. Spearman correlations were used to assess relationships between adipocyte diameter, eFAT scores, and multiple metrics per horse.

**Results:** Adipocyte diameter was significantly correlated with recent weight gain (*P* = .02) with no other significant correlations. Mean adipocyte area was significantly greater when measured manually than by Adiposoft (*P* = .003) due to numerous errors in the program's recognition of adipocytes in the presence of fibrosis. Fibrotic changes in equine adipose tissue mirrored those seen in humans, but interobserver agreement utilizing eFAT scoring was variable.

**Conclusions and Clinical Importance:** Equine adipocyte hypertrophy was associated with recent weight gain. Adiposoft and eFAT scores require refinement before use in fibrotic equine adipose tissue.

## ABSTRACT E20: Pituitary histomorphometry correlation with adrenocorticotrophin response to thyrotropin‐releasing hormone in pituitary pars intermedia dysfunction diagnosis

### **Wenqing Wang** ^1^; François‐René Bertin^2,3^, DVM, MS, PhD, FHEA, DACVIM (LAIM); Viviana Astudillo^2^, BVSc, MNR, PhD, DACVP

#### ^1^School of Veterinary Science, The University of Queensland, Brisbane, QLD, Australia; ^2^The University of Queensland, Brisbane, QLD, Australia; ^3^Purdue University, West Lafayette, IN, USA

**Background:** The thyrotrophin‐releasing hormone (TRH) stimulation test is widely recommended by equine endocrinologists for the clinical diagnosis of pituitary pars intermedia dysfunction (PPID); however, the correlation between the response of adrenocorticotropic hormone (ACTH) in TRH stimulation test and pituitary histomorphometry is still unknown.

**Hypothesis/Objective:** This study is to investigate the correlation between the pituitary histomorphometry and the ACTH concentrations after a TRH stimulation test.

**Animals:** Fourteen horses ≥13 years of age, including 9 cases presenting typical PPID signs such as hypertrichosis, delayed shedding, and muscle atrophy, were euthanized in spring and summer for reasons unrelated to this study.

**Methods:** In all horses, 1 mg TRH was administered intravenously and ACTH concentrations determined 30 min later. After euthanasia, each pituitary gland was collected and hematoxylin‐eosin stained slides were assessed by a board‐certified pathologist blinded to the clinical information using a previously published grading system. Horses were divided into five groups by pituitary histology and one‐way analysis of variance (ANOVA) used to determine the associations between histological grade and post‐TRH ACTH concentrations with *P* < .05 considered significant.

**Results:** There was a significant histology group effect (*P* = .04) on post‐TRH ACTH concentrations with higher histology grades having higher post‐TRH ACTH concentrations.

**Conclusions and Clinical Importance:** This study supports the use of the TRH stimulation test for the diagnosis of PPID as it correlates with pituitary histomorphometry. The recruitment of additional cases will allow the determination of more precise diagnostic cut‐offs for the TRH stimulation test.

## ABSTRACT E21: Fecal concentrations of fatty acids and sterols in horses with colitis

### **Jonathan L. Turck** ^1^; Claire Long^1^; Carolyn Arnold^2^, DVM, PhD, DACVS; Chih‐Chun Chen^1^, DVM; Jan Suchodolski^1^, MedVet, DrVetMed, PhD, AGAF, DACVM; Rachel Pilla^1^, DVM, PhD

#### ^1^Texas A&M University, Bryan, TX, USA; ^2^Texas Tech University, Lubbock, TX, USA

**Background:** Colitis is a leading cause of gastrointestinal disease in horses. Evidence from other animals suggests that measurement of fecal concentrations of selected fatty acids (FA) and zoo‐ and phytosterols may serve as biomarkers of epithelial barrier damage and intestinal inflammation. Little is known about the fecal FA and sterol profiles of horses with colitis and its etiology.

**Objective:** Determine the fecal FA and sterol concentrations of healthy horses and in those with clinically determined subtypes of colitis.

**Animals:** Healthy horses (HC, n = 31) and those with various subtypes of colitis: antibiotic‐associated diarrhea (AAD, n = 13), infectious (INFEC, n = 22, includes *Salmonella* and Clostridia), and inflammatory bowel disease (IBD, n = 7).

**Methods:** Targeted metabolomic analysis performed on lyophilized feces with gas chromatography/tandem accurate mass spectrometry. Metabolite concentrations were analyzed using one‐way ANOVA and adjusted for multiple comparisons.

**Results:** When compared with HC horses, there was a significant (*P* < .05) decrease in myristate (AAD), and significant increases in stearate (INFEC, IBD), nervonate, arachidonate, and total FA (INFEC). Among sterols, campesterol:cholesterol, lathosterol:cholesterol, and sitosterol:cholesterol ratios were decreased (AAD, INFEC, IBD) while concentrations of cholestanol, zoosterol, coprostanol, and total sterol (INFEC, IBD) were increased. Compared to colitis horses that survived, those that died had elevated concentrations of coprostanol.

**Conclusions and Clinical Importance:** Fecal FA and sterols are altered in horses with colitis, suggesting changes in metabolic processes of the GI tract epithelium. Fecal FA and sterol profiles may be developed as clinical predictors for colitis' presence and severity.

## ABSTRACT E22: Intramuscular EHV vaccination results in systemic and mucosal antibodies

### **Bettina Wagner**; Heather Freer; Alicia Rollins

#### Cornell University, Ithaca, NY, USA

**Background:** Equine herpesvirus type‐1 (EHV‐1) infects through the epithelium of the upper respiratory tract (URT). Mucosal antibodies (mucAbs) against EHV‐1 were shown previously to correlate with protection from disease. EHV vaccination is typically performed intramuscularly (i.m.). Transfer of systemic vaccine antibodies to the URT has not been shown.

**Hypothesis/Objectives:** The hypotheses were (i) intramuscular vaccination will result in both systemic and mucAbs, and (ii) frequent vaccination will not increase antibodies beyond certain concentrations. The objective was to provide information on systemic and mucAb responses after frequent vaccination of horses with prior EHV vaccination and/or infection history.

**Animals:** Fourteen Icelandic research horses, 5‐13 years old, ten mares, five geldings. All horses had existing EHV‐1 antibodies prior to this vaccination study and thus resemble adult client horses with EHV vaccination history.

**Methods:** Descriptive longitudinal vaccination study, Calvenza EHV(R) i.m. on days 0, 22 and 2, 3, 6, and 8 months. Serum and nasal swab samples were collected at different times post vaccination and used for antibody detection in a new sensitive EHV‐1 Risk Evaluation assay. EHV‐1 specific antibody responses were compared pre‐ and post‐vaccination by Friedman tests with Dunn's post‐tests.

**Results:** EHV‐1 specific serum antibodies plateaued after the second and subsequent vaccinations. MucAbs significantly increased beyond pre‐vaccination levels after the fourth vaccination and consisted mostly of IgG4/7.

**Conclusions/Clinical Importance:** Intramuscular vaccination resulted in increasing mucAbs at the URT which likely can neutralize EHV, thereby preventing disease. Frequent vaccination increased mucAbs while serum antibodies were less affected.

## ABSTRACT E23: Exploring the diagnostic utility of neutrophil activation markers in hospitalized foals

### **Amanda Samuels** ^1^; Celine Bartish^2^, MS; Ahmed Kamr^3^, PhD; Ramiro Toribio^4^, DVM, PhD, DACVIM (LAIM)

#### ^1^College of Veterinary Medicine, The Ohio State University, Columbus, OH, USA; ^2^Veterinary Student, The Ohio State University, Columbus, OH, USA; ^3^Visiting Scholar, The Ohio State University, Columbus, OH, USA; ^4^Professor, Veterinary Clinical Sciences, The Ohio State University, Columbus, OH, USA

**Background:** In human medicine, exuberant neutrophil activation is linked to disease severity and poor prognosis. Whether exuberant neutrophil activation contributes to disease severity and non‐survival in critically ill foals is unknown.

**Hypothesis/Objectives:** To determine the plasma concentration of neutrophil activation markers (myeloperoxidase (MPO), citrullinated histone 3 (H3cit), and cell‐free DNA (cfDNA)) in healthy, sick non‐septic (SNS), and septic foals. We hypothesize that septic foals will have increased concentrations of these markers that will be positively associated with non‐survival.

**Animals:** 50 foals <5 days of age classified on admission as healthy (n = 5), SNS (sepsis score < 11, n = 26), and septic (sepsis score > 12, n = 19).

**Methods:** Prospective, longitudinal study. Blood was collected at admission and 24 hours for analysis. Plasma concentrations of cfDNA, MPO, and H3cit were measured longitudinally using colorimetry (MPO), fluorometry (cfDNA), and immunoassay (H3cit).

**Results:** cfDNA concentrations were increased in septic foals at admission (3.4 ng/mL) and 24 hours (1.8 ng/mL) compared to SNS (1.6 ng/mL and 1.0 ng/mL, respectively) and healthy foals (1.2 ng/mL and 0.7 ng/mL, respectively). The difference was not statistically significant. MPO concentrations were higher in SNS foals at admission (33 U/L) and 24 hours (36 U/L) compared to septic (29 U/L and 25 U/L, respectively) and healthy foals (20 U/L and 11 U/L, respectively). The difference was not statistically significant. Non‐survival and H3cit analysis is ongoing.

**Conclusions:** The cfDNA and MPO concentrations in the plasma are not associated with sepsis score in foals <5 days of age. Using fluorescence microscopy, current investigations are ongoing to characterize the ability of foal neutrophils to release neutrophil extracellular traps which are the primary source of neutrophil cfDNA and MPO.

## ABSTRACT E24: Comparison of 16S rRNA, shallow shotgun, and qPCR for taxonomic characterization of equine fecal microbiota

### **Carolyn E. Arnold** ^1^; Rachel Pilla^2^, DVM, PhD; Jan Suchodolski^3^, MedVet, DrVetMed, PhD, AGAF, DACVM

#### ^1^Texas Tech University, Amarillo, TX, USA; ^2^Assistant Professor, Gastrointestinal Laboratory, Texas A&M University, College Station, TX, USA; ^3^Professor, Gastrointestinal Laboratory, Texas A&M University, College Station, TX, USA

**Background:** Taxonomic classification of the equine fecal microbiome using untargeted approaches results in differences in taxonomic resolution and characterization. The development of targeted assays may provide validated reference intervals to complement untargeted approaches allowing comparisons across methods.

**Hypothesis/Objectives:** Compare taxonomic data derived from 16S rRNA gene (16S) and shallow shotgun sequencing (SSS), and targeted qPCR assays performed on one sample set.

**Animals:** Feces from 28 horses (n = 17 non‐hospitalized, healthy horses; n = 11 hospitalized horses with colitis).

**Methods:** DNA was extracted from feces and submitted for 16S and SSS. qPCR for taxa previously found to be discriminatory between states of gastrointestinal health or disease was performed. Taxonomic abundance from 16S and SSS was compared using a Mann‐Whitney test and correlations between sequencing and qPCR were made with a Spearman's test.

**Results:** SSS identified a greater number of taxa at all taxonomic levels. There were significant differences in the abundance of 9 out of 10 phyla, including those important in gastrointestinal health or disease; Bacteroidetes was significantly less abundant by SSS compared to 16S and Verrucomicrobia was not detected by SSS. Spearman's correlation was significant (*P* < .05) between qPCR and SSS (*Blautia*, *Escherichia coli*, Firmicutes, *Lactobacillus*, *Ruminococcus*, *C. scindens*, and *Streptococcus*) and qPCR and 16S (*Akkermansia*, *Lactobacillus*, *Ruminococcus*, and *Streptococcus*).

**Conclusions and Clinical Importance:** While SSS has higher resolution at the genus and species level, 16S identified taxa critical to gastrointestinal health/disease. Targeted qPCR assays can be used to validate taxa identified with either untargeted method.

## ABSTRACT E25: Comparison of mask and binasal prongs for delivery of non‐invasive ventilation to foals

### **Sharanne L. Raidal** ^1^; Melanie Catanchin^2^, BVetBiol, BVetSc (Hons 1), GradDipEd (Tertiary); Heidi Lehmann^3^, BVMS, DACVAA; Chris Quinn^3^, BSc (Vet) (Hons), BVSc (Hons), MANZCVS, DACVAA, DECVAA; Michael van Diggelen^4^, BVSc (Hons)

#### ^1^School of Agricultural, Environmental, and Veterinary Sciences, Charles Sturt University, Wagga Wagga, NSW, Australia; ^2^Lecturer in Veterinary Anesthesia, Veterinary Clinical Centre, Charles Sturt University, Wagga Wagga, NSW, Australia; ^3^Senior Lecturer in Veterinary Anesthesia, Veterinary Clinical Centre, Charles Sturt University, Wagga Wagga, NSW, Australia; ^4^Veterinary Clinical Centre, Charles Sturt University, Wagga Wagga, NSW, Australia

**Background:** Non‐invasive ventilation (NIV) can be used to provide respiratory support to foals, but current methods have been associated with hypercapnia.

**Objectives:** Comparison of NIV delivered by binasal prongs or mask to healthy, sedated foals with pharmacologically induced respiratory insufficiency.

**Animals:** Six healthy foals, <5 weeks old.

**Methods:** Randomized cross‐over study. Six foals were blocked in pairs, with one foal in each pair randomly allocated to mask, the other to binasal prongs, for NIV. Foals were sedated and placed in lateral recumbency, with inspired/expired and blood gas analysis, spirometry, and ventilator variables collected at the end of each 10 minute intervention window of, sequentially, unassisted respiration through mask/prongs, supplementary oxygen delivered through mask/prongs, and NIV at two different pressures, with and without supplementary oxygen. After a recovery interval of ≥3 days, respiratory support was repeated with the reciprocal patient‐device interface.

**Results:** Binasal prongs were well tolerated and required less manual positioning or monitoring than the mask. Similar benefits to oxygenation and respiratory mechanics were observed with both patient‐device interfaces. Partial pressure of carbon dioxide (PaCO_2_) was significantly lower with binasal prongs (*P* ≤ .022, Figure 1) during O_2_ supplementation and at all NIV settings. Increased leak was observed during NIV with binasal prongs, but the observed leak (mean 5.3 L/min, 95% CI 2.9‐7.6 L/min) was within acceptable limits for human patients.

**Conclusions and Clinical Importance:** Nasal prongs were well tolerated, effected similar benefits on respiratory function, and appeared to ameliorate hypercapnia observed in previous studies of NIV in foals.

**Figure:**
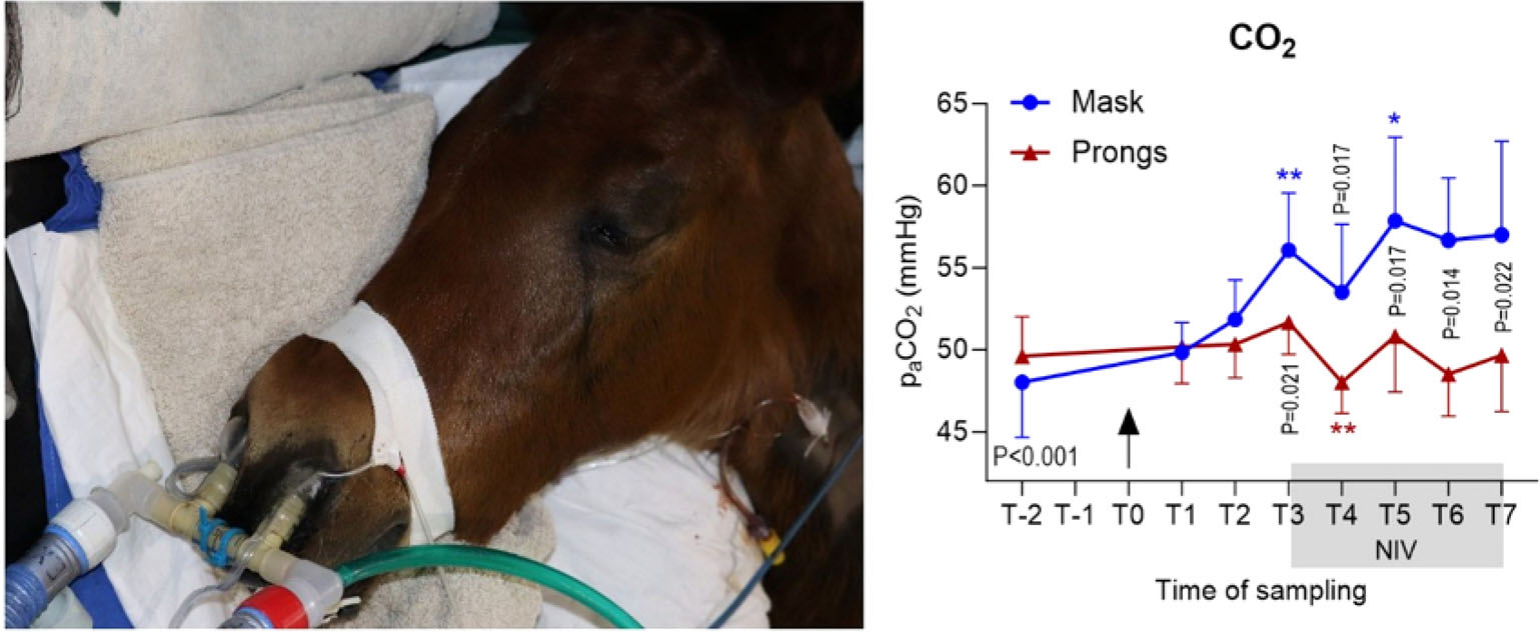
**Figure 1.** Non‐invasive ventilation using binasal prongs (left), and arterial partial pressure of carbon dioxide in foals during NIV delivered by mask or binasal prongs (right).

Results at T‐2 are from standing, unsedated foals. Foals were sedated and placed in lateral recumbency at T0; T1 and T2 represent unassisted ventilation with and without supplementary O_2_; NIV was delivered T4 to T7 at differing pressures, ± oxygen supplementation. Results are shown as mean and 95% CI, with significant differences within treatment indicated (*, *P* < .05; **, *P* < .01), and significant differences between treatments shown at specific time points.

## ABSTRACT E26: Evaluation of digital radiographic measurements for the diagnosis of acute laminitis

### **Georgia Skelton** ^1^; Darko Stefanovski^2^, PhD; Elizabeth Acutt^3^, BSc, BVSc, MS, ACVR‐EDI; Andrew Van Eps^4^, BVSc, PhD, DACVIM

#### ^1^University of Pennsylvania, West Chester, PA, USA; ^2^Associate Professor, Department of Clinical Studies, New Bolton Center, University of Pennsylvania, Kennet Square, PA, USA; ^3^Assistant Professor, Department of Clinical Studies, New Bolton Center, University of Pennsylvania, Kennet Square, PA, USA; ^4^Professor, Department of Clinical Studies, New Bolton Center, University of Pennsylvania, Kennet Square, PA, USA

**Background:** Traditional radiographic measurements of distal phalanx (Pd) displacement are not considered useful for diagnosis and monitoring of acute laminitis.

**Objectives:** Compare the radiographic distance between inner hoof wall and Pd (“lamellar lucent zone;” LLZ) for healthy horses vs. acute laminitis.

**Animals:** Lateromedial forelimb radiographs from healthy (university and client‐owned) and acutely laminitic (client‐owned, hospitalized) mixed‐breed horses were analyzed retrospectively.

**Methods:** Acute laminitis was defined by ≥2 clinical signs (acute multi‐limb lameness; increased digital pulse amplitude; persistently warm hooves) for ≤3 days, without radiographic evidence of palmar rotation (≥3°), remodeling, or obvious distal displacement of the Pd. Radiographic measurements (Figure 1A) were compared blindly between 18 acute laminitis cases (14 sepsis‐related, 4 hyperinsulinemia‐associated) and 32 healthy control horses using a mixed‐effects linear regression model and receiver‐operator characteristic (ROC) curves.

**Results:** Marginal mean [95% confidence interval] LLZ (mm) was increased in acute laminitis compared to control in the proximal (8.8 [8.4‐9.2] vs. 7.3 [6.9‐7.7]), middle (8.9 [8.5‐9.3] vs. 6.9 [6.5‐7.2]), and distal (9.2 [8.6‐9.7] vs. 7.5 [7‐8]) dorsal lamellar regions (*P* < .001) (Figure 1B). With a cutoff of >7.5 mm middle LLZ was 87% sensitive and 91% specific for identification of acute laminitis (ROC area‐under‐the‐curve [AUC] = 0.96). The ratio of LLZ:Pd palmar cortex length was 95% sensitive and 95% specific for acute laminitis with a cutoff >11% (AUC = 0.99) (Figure 1C).

**Conclusions and Clinical Importance:** Radiographic measurements of LLZ are potentially useful for diagnosis of acute laminitis; their utility can be further improved using the ratio to Pd cortex length.

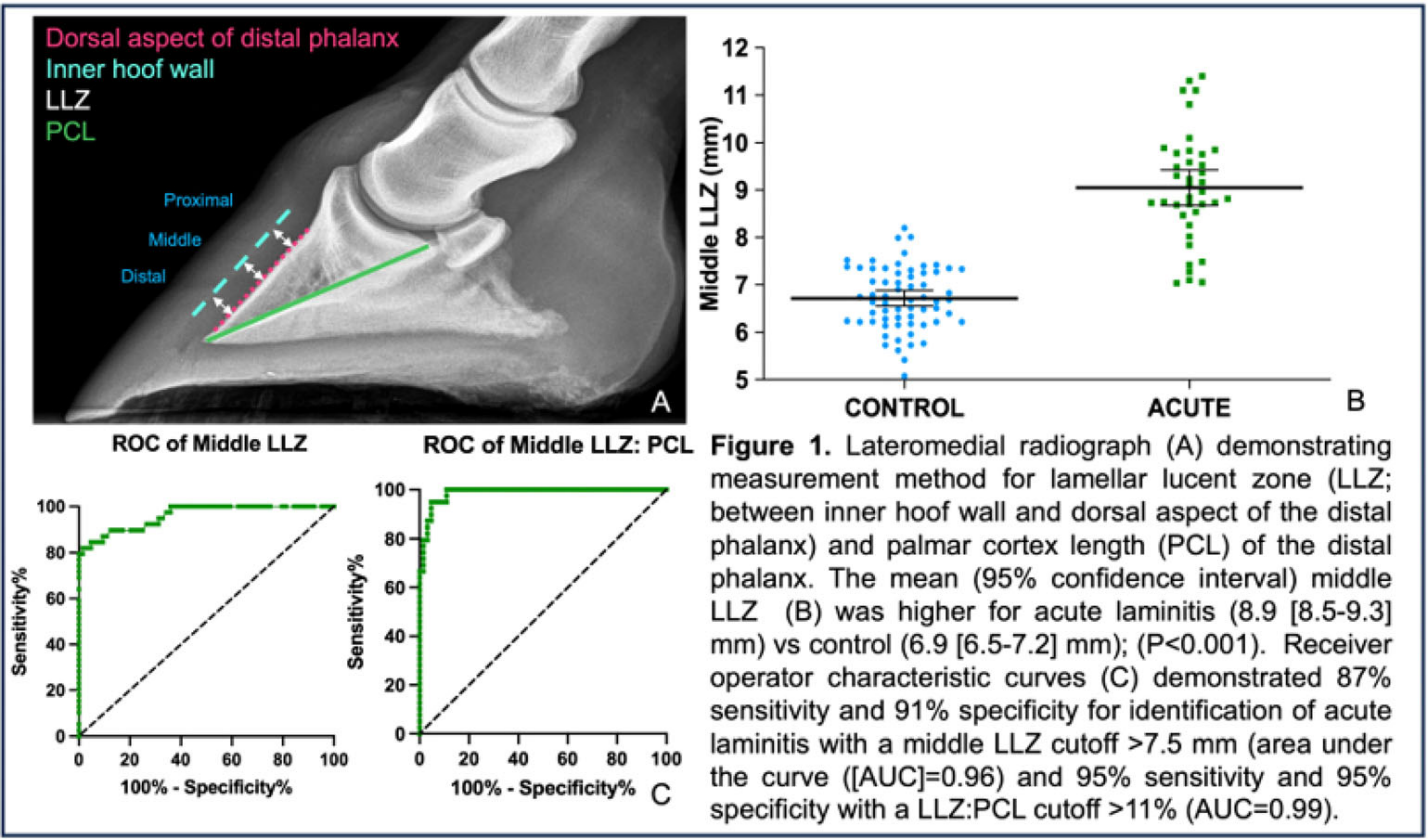

## ABSTRACT E27: Phenylbutazone improves insulin sensitivity and reduces insulin secretion in horses with insulin dysregulation

### **Kate L. Kemp** ^1^; Nicholas K.Y. Yuen^1^, BVSc (Hons), PhD; Jazmine Skinner^2^, BAppSc (Hons), PhD; ^3^François‐René Bertin, DVM, MS, PhD, FHEA, DACVIM (LAIM)

#### ^1^The University of Queensland, Gatton, QLD, Australia; ^2^Lecturer (Animal Science), School of Agriculture and Environmental Science, University of Southern Queensland, Toowoomba, QLD, Australia; ^3^Associate Professor, Equine Internal Medicine, School of Veterinary Science, The University of Queensland, Gatton, QLD, Australia

**Background:** Non‐steroidal anti‐inflammatory drugs (NSAIDs) have been reported to increase insulin secretion in patients with diabetes. Horses with insulin dysregulation (ID) often receive phenylbutazone to manage the pain of hyperinsulinemia‐associated laminitis (HAL).

**Hypothesis/Objectives:** Investigate the effects of phenylbutazone on insulin and glucose dynamics in horses with ID.

**Animals:** 16 light breed horses, including 7 with ID.

**Methods:** In a randomized crossover trial, horses received phenylbutazone (4.4 mg/kg IV daily) or placebo, with a 10‐day washout period between treatments. On day 8 of treatment a modified frequently sampled intravenous glucose tolerance test (mFSIGTT) was performed and on day 9 an oral glucose test (OGT). Insulin, glucose, and incretin concentrations were measured. Paired t tests or Wilcoxon signed‐rank test were used, with *P* < .05 considered significant.

**Results:** In horses with ID, phenylbutazone significantly decreased both glucose area under the curve (AUC; 1198 ± 224.5 vs. 1412 ± 182.6 mmol/L x min, phenylbutazone vs. placebo; *P* = .01) and insulin AUC (17 710 ± 6676 vs. 22 930 ± 8788 μIU/mL x min, phenylbutazone vs. placebo; *P* = .03) during the OGT. There was no significant effect of phenylbutazone treatment on incretins during the OGT. Phenylbutazone improved the tissue insulin sensitivity index (0.56 [0.55‐1.18] vs. 0.39 [0.14‐0.74] × 10^−4^ L/mU/min, phenylbutazone vs. placebo, *P* = .03) during the mFSIGTT. No significant effect was observed in control horses.

**Conclusions and Clinical Importance:** Phenylbutazone improves insulin sensitivity resulting in a decreased insulin secretion, making NSAIDs an attractive pathway for investigation in the management of HAL.

## ABSTRACT E28: Prevalence of pituitary pars intermedia dysfunction and insulin dysregulation and endocrine‐associated clinical signs in ponies

### **Rachel Lemcke** ^1^; Kelly Graber^2^; Steve Grubbs^3^, DVM, PhD, DACVIM

#### ^1^Amwell Data Services LLC, Ringoes, NJ, USA; ^2^Boehringer Ingelheim Animal Health USA, Inc., Clermont, GA, USA; ^3^Boehringer Ingelheim Animal Health USA, Inc., Stweartsville, MO, USA

**Background:** Ponies have been shown to have high rates of endocrine disorders (ED), but ED and endocrine‐associated clinical sign (EACS) frequencies among pony breeds are poorly understood.

**Hypothesis/Objectives:** The objective was to compare PPID and ID status and frequency of EACS within pony breeds.

**Animals:** Ponies (n = 3231) with EACS were tested for ED by their veterinarian.

**Methods:** A retrospective analysis was performed on a 2016‐2023 study of ponies with suspected ED. Ponies enrolled before 2021 were considered PPID positive if spring basal, spring post‐TRH‐stimulated, or fall basal ACTH levels were >35, >110, or >50 pg/mL, respectively; if enrolled after 2020, cutoffs were > 30, >110, or >100 pg/mL, respectively. Ponies were considered ID positive if basal insulin levels >20 μIU/mL. Frequencies of PPID, ID, laminitis, hair coat changes, regional adiposity, decreased athletic performance/lethargy, and recurrent infections were analyzed using chi‐square or two‐way ANOVA.

**Results:** Two‐thirds of each pony breed had at least one ED, with both PPID and ID identified in more than 25% of each breed. ID was more prevalent than PPID in all pony breeds. PPID and ID rates were highest in Hackneys and Shetlands at 62.22 and 79.12%, respectively, while Haflingers had the lowest rate of either ED at 32.76 and 61.49%, respectively. EACS rates were not statistically significantly different among pony breeds (*P* > .9999), but all signs evaluated were statistically associated with endocrine classification (*P* = .0024 to <.001).

**Conclusion/Clinical Importance:** These observations demonstrate the need for in‐depth endocrinology testing and management for ponies.

**Table:** Table showing pony breeds evaluated, and the quantity and percentages of ponies with identified endocrine disorders (eg, PPID and/or ID) per breed

	PPID+/ID+		PPID+/ID−		PPID‐/ID+		PPID‐/ID−			
Pony breed	Qty ponies	Perc.	Qty ponies	Perc.	Qty ponies	Perc.	Qty ponies	Perc.	Total qty ponies	Perc. ponies with endocrine disorder
Connemara	25	33.33	9	12.00	28	37.33	13	17.33	75	82.67
Fjord	26	28.89	9	10.00	30	33.33	25	27.78	90	72.22
Hackney	24	53.33	4	8.89	9	20.00	8	17.78	45	82.22
Haflinger	88	25.29	26	7.47	126	36.21	108	31.03	348	68.97
Icelandic	34	30.36	10	8.93	40	35.71	28	25.00	112	75.00
Miniature	666	46.31	183	12.73	395	27.47	194	13.49	1438	86.51
Pony of the Americas	87	38.50	36	15.93	69	30.53	34	15.04	226	84.96
Shetland	173	50.88	27	7.94	96	28.24	44	12.94	340	87.06
Welsh Cob	25	43.10	2	3.45	20	34.48	11	18.97	58	81.03
Welsh Pony	189	37.88	40	8.02	189	37.88	81	16.23	499	83.77

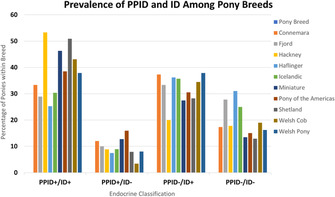

## ABSTRACT E29: Tissue doppler imaging assessment of the left ventricle in healthy standardbred newborn foals

### **Francesca Freccero** ^1^; Fernanda Timbó D'el Rey Dantas^2^, DVM, MS; Viola Cevoli^1^, DVM; Giovanni Romito^3^, DVM, SMIPPV, MSc, PhD, DECVIM‐CA (Cardiology); Laura Menchetti^4^; Carolina Castagnetti^5^, DVM, PhD, DECAR

#### ^1^Università di Bologna, Bologna, BO, Italy; ^2^PhD Candidate, Dipartimento di Scienze Mediche Veterinarie, Università di Bologna, Bologna, BO, Italy; ^3^Senior Assistant Professor, Università di Bologna, Bologna, BO, Italy; ^4^School of Biosciences and Veterinary Medicine, Università di Camerino, Camerino, MC, Italy; ^5^Full professor, Università di Bologna, Bologna, BO, Italy

**Background:** While tissue Doppler imaging (TDI) is used for left ventricular (LV) assessment in horses, its application has not been described in foals.

**Hypothesis/Objectives:** To investigate the feasibility of pulsed‐wave (PW) TDI for characterization of LV radial and longitudinal wall motion in healthy foals, and to describe TDI features during foals' first days of life.

**Animals:** Seven healthy Standardbred newborn foals.

**Methods:** Prospective observational study. Foals underwent complete transthoracic echocardiography without sedation at 24 hours, 72 hours and 5 days of life. In each foal, PW TDI tracings were acquired by multiple views at the level of the interventricular septum and LV free wall to assess radial and longitudinal velocity profiles. Effect of age, body weight, heart rate and echocardiographic views on PW TDI peak velocities, and correlations between parameters were evaluated (linear mixed models, Spearman's rho coefficient).

**Results:** In all foals, PW TDI velocity profiles were acquirable and interpretable. A 5‐wave pattern, characterized by the presence of S1 (peak velocity during isovolumic contraction), S_m_ (peak systolic velocity), E1 (peak velocity during isovolumic relaxation), E_m_ (peak proto‐diastolic velocity) and A_m_ (peak late diastolic velocity) was documented. Interestingly, S_m_ and E_m_ increased with age, E_m_ and A_m_ were positively correlated with heart rate, while S_m_, E_m_ and A_m_ were inconsistently correlated with different echocardiographic views.

**Conclusions and Clinical Importance:** In healthy newborn foals, PW TDI assessment of LV function is feasible, although several variables should be considered for a correct interpretation of velocity profiles, including age.

## ABSTRACT E30: Impact of short distance transportation on horses with and without pars pituitary intermedia dysfunction (PPID)

### **Erica T. Jacquay** ^1^; Amanda Adams^2^; Patricia Harris^3^

#### ^1^University of Kentucky, Lexington, KY, USA; ^2^Gluck Equine Research Center, University of Kentucky, Lexington, KY, USA; ^3^Equine Studies Group, Waltham Petcare Science Institute, Waltham on the Wolds, Leicestershire, UK

**Background:** Unknown how short distance transportation stress impacts horses with PPID.

**Hypothesis/Objectives:** To determine if transportation alters diagnostic results for PPID.

**Animals:** 12 non‐insulin dysregulated horses of mixed light breed were age‐matched and categorized by thyrotropin stimulating hormone (TRH) stimulation test and hypertrichosis score (HS) as PPID (n = 6, T10 TRH >200 pg/mL, HS ≥1) and non‐PPID (n = 6, TRH T10 < 200 pg/mL, HS <1). A further 3 PPID horses (TRH >1200 pg/mL, HS ≥1) were evaluated in a second study (PPID:A).

**Methods:** Horses were transported once by road (1.5 hour round trip: 3‐4 horses/trip). Blood (for ACTH, insulin, and cortisol) and saliva (cortisol) were collected on the day of transportation: 1 hour before loading, directly after unloading plus 15 minutes, 30 minutes, 1 hour, 2 hour and 24 hours post transportation.

**Results:** At unloading and 15 minutes post‐transportation serum cortisol was significantly increased in PPID and non‐PPID horses (*P* < .01), while salivary cortisol was increased in non‐PPID horses (*P* < .05). Plasma ACTH was 2‐4x higher in PPID horses vs non‐PPID horses at unloading (*P* = .02) and 15 minutes post‐transportation (*P* = .02). Two non‐PPID horses had unloading post‐transportation ACTH concentrations above the basal ACTH diagnostic cut‐off for PPID (>40 pg/mL). There were no differences in serum insulin in response to transportation or PPID status. PPID:A horses had increased ACTH concentrations at 15 minutes post‐transportation (*P* < .05); serum cortisol was 3x lower than PPID/non‐PPID horses.

**Conclusions and Clinical Importance:** Caution should be taken when performing diagnostic testing for PPID shortly after short distance transportation due to possible increased ACTH.

## ABSTRACT E31: Fecal microbiota and serum metabolome association with equine metabolic syndrome in connemara ponies

### **Ahmed Al Ansari** ^1^; Nicola Walshe^2^, PhD; Grace Mulcahy^3^; Vivienne Duggan^4^, MVB, PhD, DACVIM, DECEIM

#### ^1^University College Dublin, Belfield, Dublin, Ireland; ^2^Assistant Professor, University College Dublin, Belfield, Dublin, Ireland; ^3^Professor, University College Dublin, Belfield, Dublin, Ireland; ^4^Associate Professor, Equine Clinical Studies, University College Dublin, Belfield, Dublin, Ireland

**Background:** Fecal microbiome and serum metabolome have been studied in human medicine to provide a better understanding of metabolic derangements including diabetes, but studies in equine medicine are limited.

**Hypothesis/Objectives:** This is a case‐control study conducted to identify differences in fecal microbiota and serum metabolites between metabolically normal Connemara ponies and those with equine metabolic syndrome (EMS).

**Animals:** Thirty privately owned Connemara ponies: 15 EMS Phenotype and 15 non‐EMS.

**Methods:** EMS was diagnosed by oral sugar test (OST). 16S rRNA gene sequencing was used to identify the microbial communities in fecal samples. ALDEx2 (ANOVA‐like differential expression) and LinDA (linear model for differential abundance analysis) were used to assess the differences in microbial abundance between groups. Serum metabolites were analyzed using liquid chromatography‐high‐resolution mass spectrometry (LC‐MS). “Weighted” gene co‐expression network analysis (WGCNA) was used for multi‐omics integration of microbiota‐metabolome datasets.

**Results:** The microbiota community composition was significantly different between groups (*P* = .035). EMS ponies showed reduced microbial species richness and evenness compared to non‐EMS ponies. The EMS ponies showed an enrichment pattern of metabolites belonging to triglycerides, along with a reduction pattern of other metabolite classes. Multi‐omics analysis revealed two modules in metabolome and microbiota datasets that were significantly different between the EMS and non‐EMS ponies (*P* < .0001).

**Conclusions and Clinical Importance:** This study suggests that microbiota‐metabolome features differ between Connemara ponies with and without EMS. These results provide significant insights that may assist in the search for novel management methods for this condition.

## ABSTRACT E32: Developing clinical reasoning in veterinary students assessing equine colic—barriers and positive teaching strategies

### Claire E. Dixon

#### Tufts University, North Grafton, MA, USA

**Background:** Clinical reasoning is a vital but complex skill required by veterinarians that assess equine colic. The veterinary student's ability to use clinical reasoning in these cases must be evaluated, as this skillset is ‘content‐specific’.

**Hypothesis/Objectives:** To investigate how veterinary students approach equine acute colic following completion of an alimentary teaching module. The study goal was to discover information that helps or hinders student learning or the development of clinical reasoning skills.

**Methods:** Fifteen third‐year students were recruited to participate in focus groups after completion of an alimentary teaching module. Reflexive thematic analysis was performed on focus group data.

**Results:** Two main themes with six subthemes were developed: (1) How to Help Students (with subthemes ‘as close to real life as possible’, ‘challenging topics/foundational concepts’ and ‘teaching environment’) and (2) Student Struggles (with subthemes of ‘uncertainty’, ‘horses as unicorns’ and ‘clinical reasoning’).

**Conclusions:** Students struggle in developing clinical reasoning skills in many areas of equine colic due to lack of underlying knowledge and case experience. These data support the need for developing further educational tools to advance student skills to an appropriate level for new graduates. Foundational knowledge (anatomy, pathophysiology, parasitology) and realistic case exposures should be incorporated, while fostering positive relationships with clinicians or teachers in a safe learning environment. Strategies to expose students to the ‘gray zone’ of veterinary medicine are important, since uncertainty is a significant yet unavoidable challenge.

## ABSTRACT E33: The effects of *Streptococcus equi equi* status on the upper respiratory bacterial microbiota of horses

### **Ashley G. Boyle**; Elizabeth Nelson; Jane Woodrow; Nagaraju Indugu; Reenu Kashyap; Kapil Narayan; Dipti Pitta; Tom Schaer; Terry Webb

#### Department of Clinical Studies, New Bolton Center, University of Pennsylvania, Kennett Square, PA, USA

**Background:** Exploring the guttural pouch bacterial microbiota during *Streptococcus equi equi* (*S. equi*) infection may determine indicators that can lead to biofilm formation.

**Hypothesis/Objectives:** To assess the effects of *S. equi* status on the equine upper respiratory microbiota.

**Animals:** 10 healthy university‐owned (*S. equi* negative), 9 strangles convalescent client‐owned (*S. equi* negative), and 9 strangles convalescent client‐owned (*S. equi* positive) horses were enrolled.

**Methods:** In this prospective observational study, oral wash (OW), nasopharyngeal lavage (NPL), and guttural pouch lavage (GPL) samples were collected and processed for genomic DNA extraction. PCR amplification and Illumina sequencing was performed followed by data analysis using QIIME2 pipelines.

**Results:** Observed species and Shannon diversity metrics showed significant differences between OW, NPL and GPL samples (*P* = .001). GPL had much higher diversity compared to OW and NPL (*P* = .005). The extent of interaction between commonly present bacterial populations (beta diversity) resulted in a separation by sample type (*P* = .001) and a difference between *S. equi* positive and negative samples (*P* = .046). Firmicutes was the most abundant phylum across all sample types. Proteobacteria were reduced in GPL *S. equi* positive samples. *S. equi* negative OW and NPL samples had more Gemellaceae whereas *Streptococcus* was proportionally increased in *S. equi* positive GPL samples.

**Conclusions and Clinical Importance:** While genera such as Gemellaceae are commonly shared between the upper respiratory tract microbiota, the microbiome associated with the guttural pouch appears distinct with a rich diversity of *Streptococcus*‐related genera. Despite low sample size, *S. equi* positive guttural pouch microbiome differed from negative horses.

## ABSTRACT E34: Unsaddling *Streptococcus equi*—European experiences with a new fusion protein vaccine against strangles

### Andrew S. Waller

#### Intervacc AB, Stockholm, Stockholms Lan, Sweden

**Background:** Strangles, caused by *Streptococcus equi* subspecies *equi*, is one of the most prevalent infectious diseases of horses leading to significant levels of morbidity, mortality and economic cost. In 2022 a recombinant fusion protein vaccine, Strangvac, was launched for sale in Europe. Strangvac does not contain the proteins SEQ2190 or SeM, which are used in a dual‐antigen iELISA that identifies horses exposed to *S. equi* with a reported sensitivity of 93.3% and specificity of 99.3%.

**Hypothesis/Objectives:** We hypothesized that the exposure of vaccinated horses to *S. equi* leads to a positive serological test result, enabling the identification of protected horses.

**Animals:** Blood serum samples from 41 horses were collected by veterinarians attending three outbreaks of strangles to determine if they had been exposed to *S. equi* as part of outbreak investigations.

**Methods:** Serum samples from the case series were analyzed using the dual antigen iELISA for strangles.

**Results:** Five of 10 vaccinated horses tested seropositive in outbreak 1. All three non‐vaccinated clinical cases of strangles and eight of 17 vaccinated horses tested seropositive in outbreak 2 (see Figure). Six of 11 vaccinated horses tested seropositive in outbreak 3.

**Conclusions and Clinical Importance:** All three clinical cases and 19 of 38 (50%) vaccinated horses tested positive for exposure to *S. equi*, but none of the 38 vaccinated horses developed clinical signs of strangles. Our data provide evidence supporting a protective effect of vaccination with Strangvac against natural exposure to *S. equi*.

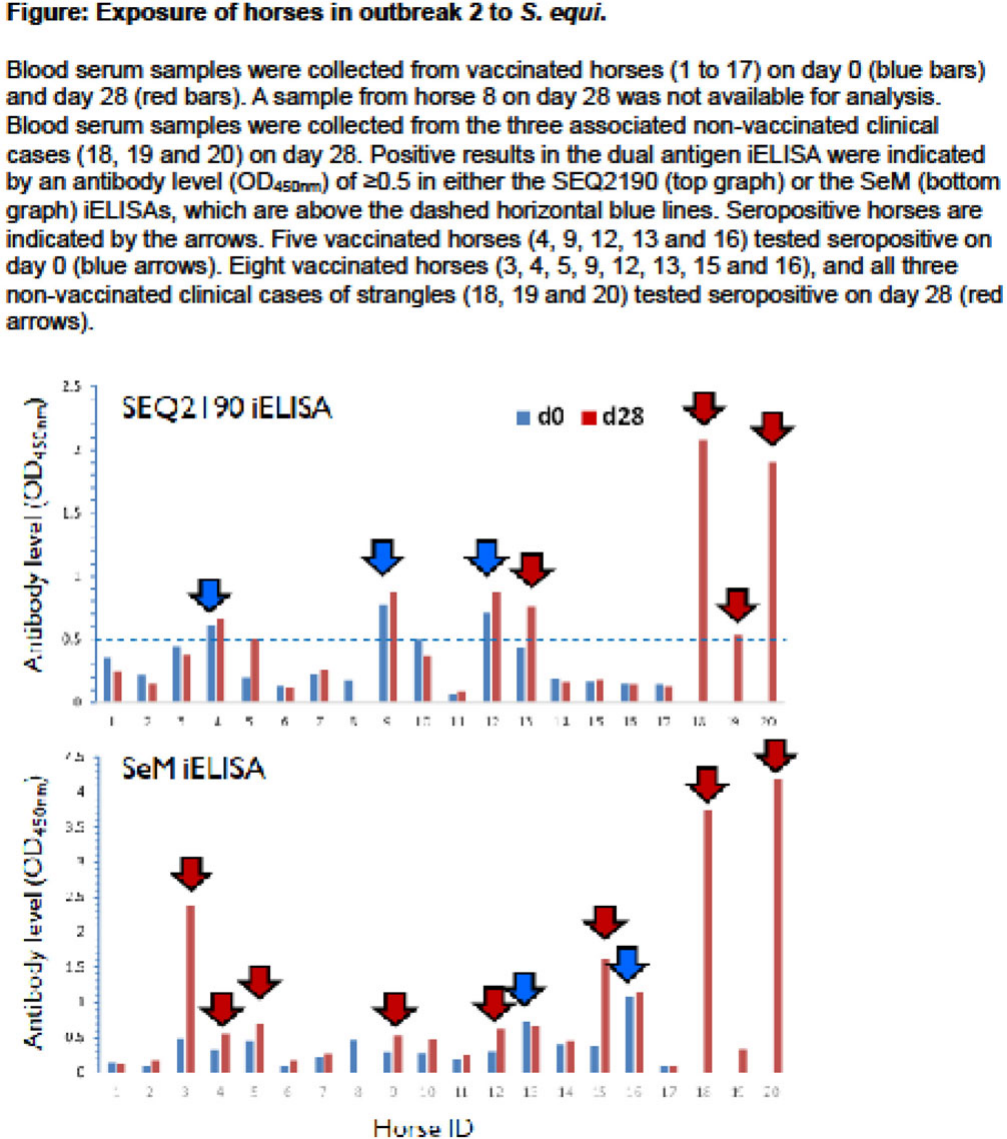

## ABSTRACT E35: Safety of strangvac, a vaccine against *Streptococcus equi*, in European horses

### Andrew S. Waller

#### Intervacc AB, Stockholm, Stockholms Lan, Sweden

**Background:** Strangvac, a new vaccine against strangles, contains eight important proteins of *Streptococcus equi*. Thousands of horses across Europe have been vaccinated with Strangvac since its launch in 2022.

**Hypothesis/Objectives:** We hypothesized that the vaccination of horses with Strangvac induced side effects that were similar to other vaccines.

**Animals:** Feedback on the clinical signs experienced by 451 horses at four leading equestrian centres in Sweden were reported by the responsible veterinarians.

**Methods:** Horses in this case series were vaccinated on three occasions with Strangvac via intramuscular injection and then monitored for clinical signs.

**Results:** 145, 96, 60 and 150 competition horses were vaccinated at farms 1, 2, 3 and 4, respectively. Body temperatures increased in some horses for 1 day. Thirty‐nine horses had stiffness/swelling in their neck that resolved after 2‐7 days. Some horses were dull for a few days post‐vaccination. None of the vaccinated horses developed signs of purpura haemorrhagica or strangles. The veterinarian at farm 3 noted that vaccinated horses were unaffected during an outbreak of *S. zooepidemicus* infection.

**Conclusions and Clinical Importance:** Three of the four farms reported that clinical signs post‐vaccination with Strangvac were similar to those observed following the administration of other vaccines. Although the vet at farm 2 felt that Strangvac led to more side effects, they believed it was safe and continue to use this vaccine to protect their horses. Our data provide further evidence of the safety of Strangvac for the vaccination of horses.

## ABSTRACT E36: Evolution of *S. equi* in the UK: A torrid tale of population replacement by strangulation

### **Andrew S. Waller** ^1^; Abigail McGlennon^2^ ; Jonathan Newton^2^; Kristien Verheyen^3^

#### ^1^Intervacc AB, Stockholm, Stockholms Lan, Sweden; ^2^University of Cambridge, Cambridge, UK; ^3^Royal Veterinary College, Hatfield, Hertfordshire, UK

**Background:** Strangles, caused by *Streptococcus equi*, remains endemic in UK horses. The transmission of *S. equi* from healthy carrier horses that have recovered from the disease, but remain persistently infected, has been proposed to be the primary cause of new outbreaks of disease.

**Hypothesis/Objectives:** We hypothesized that genome sequencing could be utilized to track outbreaks of strangles in the UK, shedding new light on the evolution and transmission of this important pathogen.

**Animals:** With ethical approval (URN20201973‐2), *S. equi* isolates (n = 510) were recovered from clinical samples taken from horses in the UK that were submitted to six UK diagnostic laboratories between 2016 and 2022.

**Methods:** The genomic DNA of isolates was purified, sequenced and the population structure determined by Bayesian Analysis of Population Structure (fastBAPS).

**Results:** Nine fastBAPS groups were identified, although 82% of strains belonged to only two groups (fastBAPS3, n = 230, 45%; fastBAPS5, n = 189, 37%). Over the study period there was a significant increase in the prevalence of fastBAPS3 (7% in 2017 rising to 93% in 2022) and decreasing proportions of fastBAPS5 (69% in 2017 falling to <1% in 2022).

**Conclusions and Clinical Importance:** The rapid change in population structure cannot be explained by transmission from carriers. Instead, most transmission appears to occur following the movement of horses incubating strangles or those that have recovered recently. Our data suggest that interventions such as screening, quarantine and vaccination of horses could have the greatest benefit towards reducing the prevalence of strangles.

## ABSTRACT E37: Effect of changes in pasture water‐soluble carbohydrates in horses with and without insulin dysregulation

### **Morgan J. Askins** ^1^; Patricia Harris^2^, PhD; Erica Jacquay^1^; Brittany Kerley^1^; Margaret McClendon^1^ ; Amanda Adams^1^

#### ^1^M.H. Gluck Equine Research Center, Department of Veterinary Science, University of Kentucky, Lexington, KY, USA; ^2^Equine Studies Group, Waltham Petcare Science Institute, Waltham on the Wolds, Leicestershire, UK

**Background:** Restricted pasture intake is often recommended for horses at risk of laminitis but more information on the effect of changes in pasture water soluble carbohydrate (WSC) content on blood parameters in grazing insulin dysregulated (ID) equids is required.

**Hypothesis/Objectives:** To examine changes in pasture nutrient content and corresponding serum insulin concentrations in ID and non‐ID (NID) horses over a 24‐hour early fall grazing period.

**Animals:** Twelve adult horses (Mean ± SD; age 19.0 ± 3.04 yrs) were classified as ID (n = 6) and NID (n = 6) by history and diagnostic testing, using basal insulin and oral sugar tests.

**Methods:** Horses were group housed on pasture with ad libitum access to forage and water. Peripheral blood and pasture samples were collected every 2 hours for a 24‐hour period. Serum was analyzed for insulin (AIA) and cortisol (RIA) concentrations, and whole blood glucose (glucometer). Pasture samples were analyzed for crude protein, WSC, ethanol soluble sugars, and starch (Equi‐analytical). Two‐way RM ANOVA (Prism) was used.

**Results:** A change in pasture WSC from 8.3% to 11.6%, between baseline 0700 hrs and 1500 hrs, respectively, was correlated with a significant increase (*P* = 0.02) in insulin (delta mean ± SD; 86.04 ± 14.76 μIU/mL) in ID horses but no change in NID horses. Glucose changed overtime in both groups. Cortisol showed the same circadian changes in ID and NID horses.

**Conclusions and Clinical Importance:** Relatively small changes in pasture WSC can lead to significant increases in serum insulin in ID but not NID horses. Time of access should be closely monitored when allowing ID horses to graze.

## ABSTRACT E38: Investigating the impact of an algae‐derived DHA respiratory supplement on equine lower airway parameters

### **Bethanie Cooper** ^1^; Kallie Hobbs^1^, DVM, DACVIM; Kerry O'Donnell^2^; Mary Sheats^3^, DVM, PhD, DACVIM

#### ^1^North Carolina State University, Raleigh, NC, USA; ^2^Veterinary Student, North Carolina State University, Raleigh, NC, USA; ^3^Associate Professor of Primary Care, North Carolina State University, Raleigh, NC, USA

**Background:** Docosahexaenoic acid (DHA) supplementation decreases pro‐inflammatory cytokines in murine airway cells exposed to organic dust. DHA supplementation also decreases clinical signs and inflammation in asthmatic children and horses. The mechanism(s) underlying the anti‐inflammatory effects of DHA on the airway have yet to be fully elucidated. The goal of this study was to investigate the impact of an algae‐derived DHA supplement on equine alveolar macrophage metabolism and the protein and lipid profile of BAL supernatant, to better understand the potential anti‐inflammatory mechanisms of DHA.

**Hypothesis:** Administration of a commercially available algae‐derived DHA respiratory supplement in healthy adult horses will significantly alter alveolar macrophage metabolism, as well as protein and lipid profiles of BAL supernatant.

**Animals:** Ten healthy, university‐owned adult ponies.

**Methods:** A prospective, cross‐over, placebo‐controlled study. Broncho‐alveolar lavage was collected at baseline, following 45 days placebo treatment, and following 45 days oral administration of a commercially available respiratory supplement (14 day washout). Whole blood was collected following placebo treatment and following oral administration of the supplement. Alveolar macrophage oxygen consumption rate, BAL cytology, proteomics and lipidomics were analyzed.

**Results:** Seven horses received both placebo and respiratory supplement. Following supplementation, the ratio of omega 6:omega 3 was significantly decreased and the ratio of DHA:arachidonic acid was significantly increased. Supplementation resulted in an increase in alveolar macrophage metabolism. Analysis of proteomic and lipidomic bioinformatics is ongoing.

**Conclusions:** Oral supplementation of an algae‐derived DHA respiratory supplement increases alveolar macrophage metabolism which could indicate a greater population of M2 (anti‐inflammatory) alveolar macrophages.

## ABSTRACT E39: Search for biomarkers of muscle atrophy in horses and ponies with pituitary pars intermedia dysfunction

### **Nicolas C. Galinelli** ^1^; Nicholas Bamford^1^; Madison Erdody^1^; Skye MacKenzie^1^ ; Patricia Harris^2^; Simon Bailey^1^

#### ^1^Melbourne Veterinary School, The University of Melbourne, Parkville, VIC, Australia; ^2^Equine Studies Group, Waltham Petcare Science Institute, Waltham on the Wolds, Leicestershire, UK

**Background:** Muscle atrophy is increasingly recognized as an important component of pituitary pars intermedia dysfunction (PPID), contributing to loss of use and activity, but the causes are poorly understood.

**OBJECTIVES:** To determine circulating factors associated with muscle atrophy, that may help identify the underlying mechanism(s).

**Animals:** Thirty‐five horses and ponies >15 years old; 13 with PPID and 22 without PPID.

**Methods:** Muscle atrophy and body condition were assessed using validated scoring systems. Plasma IGF‐1, myostatin, activin‐A and a panel of cytokines were measured using single‐plex and multiplex ELISAs. Plasma metabolomics analysis was performed using gas chromatography‐mass spectrometry.

**Results:** Body condition and cresty neck scores were similar in the PPID and non‐PPID groups; however, muscle atrophy scores differed significantly (4‐16 scale; median, PPID: 7 and non‐PPID: 4; *P* = .003). Plasma IGF‐1, myostatin or activin A concentrations did not correlate with muscle atrophy and there were no differences between groups. However, myostatin showed a moderate correlation with body condition score (*P* = .04). In the metabolomic analysis, only L‐arginine showed a statistically significant difference, being lower in animals with muscle atrophy (*P* = .026). Cytokines and chemokines detectable in plasma were not correlated with muscle atrophy or different between groups.

**Conclusions and Clinical Importance:** The specific muscle atrophy scoring system is valuable for evaluating muscle atrophy in PPID cases. Muscle atrophy does not appear to be associated with pro‐inflammatory cytokines or circulating levels of IGF‐1, myostatin, activin A or with specific differences in metabolic pathways.

## ABSTRACT E40: Feeding grain before thyrotropin‐releasing hormone stimulation did not affect ACTH concentration in horses

### **Rhonda M. Hoffman** ^1^; Steven Grubbs^2^, DVM, PhD, DACVIM; John Haffner^3^, DVM

#### ^1^Middle Tennessee State University, Murfreesboro, TN, USA; ^2^Boehringer Ingelheim; ^3^Equine Science Center, Middle Tennessee State University, Murfreesboro, TN, USA

**Background:** The thyrotropin releasing hormone (TRH) stimulation test is a dynamic test for PPID that has been described as a sensitive diagnostic test for PPID. Published studies have reported the feeding effects of hay vs. fasting on TRH diagnostic testing results, but not the evaluation of TRH testing results in grain‐fed vs. hay‐only in PPID positive and negative horses.

**Hypothesis/Objective:** Feeding grain would not affect basal T0‐ACTH or T10‐ACTH results after TRH‐stimulation.

**Animals:** Six PPID‐positive and six PPID‐negative horses of known status were used.

**Methods:** The protocol was approved by the Institutional Animal Care and Use Committee. All horses were fed prairie grass hay as basal diet, or hay plus pelleted grain concentrate meeting NRC requirements, in a crossover design with a 2‐week testing interval. Previous work in this laboratory found consistent basal ACTH and TRH‐stimulation results at 2‐week intervals. Two hours after eating hay‐only or the grain‐fed diet, blood samples were collected before and 10 minutes after 1 mg TRH administered IV. A mixed model with repeated measures analyzed the effect of grain feeding on T0‐ACTH, T10‐ACTH, and the percent increase of ACTH after TRH‐stimulation.

**Results:** Compared to hay only, grain feeding did not affect T0‐ACTH (*P* = .22), T10‐ACTH (*P* = .09), or the percent increase in ACTH (*P* = .18) in PPID‐negative or PPID‐positive horses. No change in PPID classification was observed comparing grain‐fed vs hay‐only in PPID‐positive or PPID‐negative horses.

**Conclusions and Clinical Importance:** Based on these results, horses may be fed grain prior to TRH stimulation testing for PPID.

## ABSTRACT E41: Lymphocyte characterization in the duodenal and rectal mucosal of healthy horses exposed to three diets

### **Marine Rullier**; Daniel Jean; Peggy Moreau; Pierre Hélie; Jean‐Pierre Lavoie

#### Université de Montréal, St‐Hyacinthe, QC, Canada

**Background:** Dietary composition may have an impact on type and number of small intestinal mucosal leucocytes.

**Hypothesis/Objectives:** To quantify B and T lymphocytes in the duodenal and rectal mucosa of horses fed three different diets.

**Animals:** Seven healthy mares (n = 7) were fed for 6 weeks consecutively a diet with 1) a higher fiber content, followed by 2) a higher protein content and 3) a higher lipid content.

**Methods:** Duodenal and rectal biopsies were endoscopically (n = 4‐6) obtained from each horse after each feeding periods. Standard HEPS slides were evaluated by a board‐certified veterinary anatomic pathologist. Histomorphometry was used to count B‐cells (CD20) and T‐cells (CD3) using immunohistochemistry (absolute number per field). A mixed linear model and Benjamini‐Hochberg (post‐hoc) procedures were used to evaluate the effect of diet on B‐ and T‐cell counts.

**Results:** The duodenal and rectal epithelium contains exclusively T lymphocytes (CD3) for all diets. The number of T lymphocytes in the duodenal and rectal lamina propria in horses fed diets with higher protein content was significantly greater than when fed diets with higher fiber (*P* = .025) or higher lipid content (*P* = .009). Horses fed diets with higher protein content had significantly lower numbers of B lymphocytes in the duodenal and rectal mucosa compared to horses fed diets with higher fiber (*P* = .039) or higher lipid content (*P* = .039).

**CONCLUSION AND CLINICAL IMPORTANCE:** These data allow a more finetuned interpretation of lymphocyte numbers in duodenum and rectum biopsies in horses.

## ABSTRACT E42: Characterization of concomitant common variable immunodeficiency and cutaneous or pharyngeal lymphoma in horses

### **Kathleen R. Mullen** ^1^; M. Julia Felippe^2^, MedVet, MS, PhD, DACVIM

#### ^1^Anschutz Medical Campus, University of Colorado, CO, USA; ^2^College of Veterinary Medicine, Cornell University, Ithaca, NY, USA

**Background:** Common variable immunodeficiency (CVID) in horses is a rare, non‐familial condition characterized by late‐onset B cell depletion and/or dysfunction resulting in inadequate antibody production and predisposition to recurrent infections. Diagnosis is based on supporting clinical signs and serum IgG.

**Animals:** Submissions to the Equine Immunology Laboratory, Cornell University College of Veterinary Medicine, 2001‐2024, for horses with CVID and lymphoma (CVID+/LSA+) and horses without CVID and with lymphoma (controls).

**Hypothesis/Objectives:** CVID+/LSA+ cases have lower peripheral blood B cell distributions compared to controls.

**Methods:** A case‐controlled investigation of the clinical history and results for peripheral blood lymphocyte immunophenotyping and serum immunoglobulin concentrations were compiled for CVID+/LSA+ cases and controls.

**Results:** From 113 CVID+ cases, there were 6 pharyngeal (5.3%) and 7 cutaneous (6.2%) lymphoma cases with concomitant hypogammaglobulinemia, whereas 6 controls cases had lymphoma with normal or hypergammaglobulinemia. The 13 CVID+/LSA+ had significantly lower IgG 400 mg/dL (200, 946) [median (range)] than controls 1680 mg/dL (1270, 4800), *P* = .0007. The CD19+ B cell distributions in CVID+/LSA+ were 4.6% (0.2, 23.3) and were not significantly different from controls 20.2% (15.3, 25.0), *P* = .07.

**Conclusions and Clinical Importance:** Horses with lymphoma, particularly of pharyngeal or cutaneous localizations, should be tested for CVID when presenting with hypoglobulinemia and/or recurrent infections. The relationship between CVID and pharyngeal/cutaneous lymphoma warrants further investigation.

## ABSTRACT E44: Long‐term humoral immune protection following west Nile virus infection in horses

### **Csenge Hanna Tolnai** ^1^; Petra Forgách^2^, DVM, PhD; Orsolya Fehér^1^; Orsolya Kutasi^3^, DVM, DECEIM, PhD

#### ^1^University of Veterinary Medicine Budapest, Budapest, Hungary; ^2^Department of Microbiology and Infectious Diseases, University of Veterinary Medicine Budapest, Budapest, Hungary; ^3^Institute for Animal Breeding, Nutrition and Laboratory Animal Science, University of Veterinary Medicine Budapest, Budapest, Hungary

**Background:** In the last three decades West Nile virus (WNV) has become one of the most important viral encephalitic agents worldwide, causing significant numbers of human and equine cases every year by re‐emerging in endemic areas and emerging into new territories. It is considered that following natural WNV infection, survivors develop life‐long immune protection, however, the data are scarce in horses.

**Hypothesis:** WNV infection provides long‐term immunity in asymptomatically infected horses.

**Horses:** 25 asymptomatically infected, un‐immunized, healthy client‐owned horses from Hungary.

**Methods:** In this prospective cohort study neutralizing antibody (nAb) levels of 25 horses were monitored for 5 consecutive years. Serum samples were collected yearly from the selected animals. First, a WNV IgG ELISA was performed, followed by a micro‐virus neutralization assay.

**Results:** Data were logarithmically transformed. The results are summarized in Table 1. Repeated measures ANOVA with a Greenhouse‐Geisser correction determined that mean nAb levels differed statistically significantly across time points (F(2.645; 63.469) = 69.567; *P* < .01.

**Conclusion and Clinical Relevance:** Our results indicate a significant time effect for anti‐WNV titers (Figure 1). Since the level of nAbs provide the best correlate to *Orthoflavivirus* protection, our study indicates that horses might be unprotected against re‐infection, therefore we recommend regular nAb titer testing in endemic areas.

**Table:** Table 1. Number of IgG positive samples and mean nAb levels from 2019 to 2023

Year	Number of samples	Number of WNV IgG positive samples	Mean nAb titers	SD
2019	25	25/25	6.440	2.275
2020	25	24/25	5.918	2.141
2021	25	24/25	5.460	2.131
2022	25	24/25	2.880	1.693
2023	25	24/25	1.680	1.281

**Figure:**
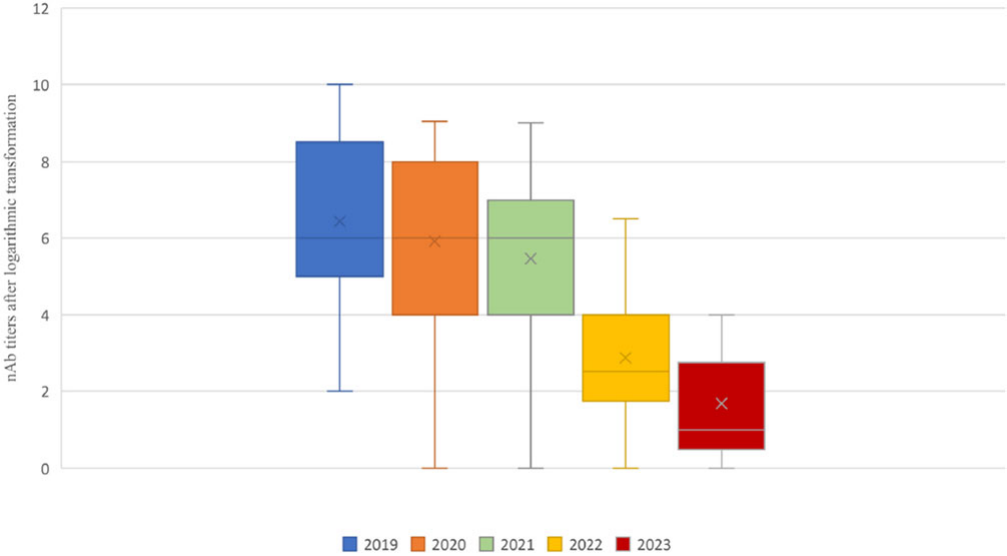
**Figure 1**. Distribution of nAb titers from 2019 to 2023.

## ABSTRACT E45: Serum parathyroid hormone, serum calcium and urinary fractional excretions in horses with nutritional secondary hyperparathyroidism

### **Camilo J. Morales** ^1^; Viviana Castillo‐Vanegas^2^, DVM, MS; K. Gary Magdesian^3^, DVM, DACVIM, DACVECC, DACVCP

#### ^1^University of California‐Davis, Davis, CA, USA; ^2^Scientific Director, VitaLab Laboratory, McDonough, GA, USA; ^3^Professor, Medicine and Epidemiology, University of California‐Davis, Davis, CA, USA

**Background:** There is little reported about the association among parathyroid hormone (PTH), urinary fractional excretion of phosphorus (FeP), and serum calcium (sCa_2_^+^) with clinical signs in horses with nutritional secondary hyperparathyroidism (NSH).

**OBJECTIVES:** To determine the association between concentrations of PTH and sCa_2_^+^, as well as FeP, in horses with clinical and subclinical NSH.

**Animals:** 116 horses in Colombia tested for NSH. Horses were assigned to three groups: (1) increased PTH + clinical signs (n = 61); (2) increased PTH and no clinical signs (n = 25); (3) PTH within the reference range (controls; n = 30).

**Methods:** Retrospective case‐control study. Only horses >2 years of age were included. Clinical horses had facial bone deformation or lameness. Pair‐wise comparisons (for PTH; subclinical vs clinical) were done with a Mann Whitney test. Three‐group comparisons were done with Kruskal‐Wallis and Dunn's multiple comparison tests.

**Results:** The median age was 7.62 (2‐17) years. 83% of horses were female. Paso Finos were the most common breed (56%). PTH was significantly increased in clinical horses (223.9 pg/mL [67.55‐1451.00]) compared to subclinical (117.1 pg/mL [65.50‐523.00]; P < 0.0001).

**Conclusions and Clinical Importance:** Horses with clinical NSH had higher PTH concentrations than horses with subclinical NSH. Horses with NSH had higher FeP as compared to horses with normal PTH. Clinical horses had lower sCa_2_^+^ than controls.

## ABSTRACT E46: Evaluation of endothelial glycocalyx shedding in horses with small intestinal disease

### **Kallie J. Hobbs** ^1^; Elsa Ludwig^1^, DVM, PhD, DACVS‐LA; Anje Bauck^2^, DVM, PhD, DACVS‐LA; Emily Martin^1^, DVM, PhD, DACVS; M. Katie Sheats^1^; David Freeman^2^, DVM, PhD, DACVS‐LA; Liara Gonzalez^1^, DVM, PhD, DACVS‐LA

#### ^1^North Carolina State University, Raleigh, NC, USA; ^2^University of Florida, Gainesville, FL, USA

**Background:** Endothelial glycocalyx (EG) degradation results in vascular hyperpermeability, inappropriate leukocyte adhesion and intravascular coagulation. Small intestinal (SI) EG shedding during inflammatory and ischemic states has been shown to be predictive of morbidity and mortality in human ICU settings. The shedding of EG components such as syndecan‐1, heparin sulfate, and hyaluronan shedding both intravascularly and peritoneally in horses has not been evaluated. Greater understanding of EG dysregulation may contribute to improved clinical management and may have diagnostic utility in horses with SI disease.

**Hypothesis:** Therefore, the main objective of this study was to determine if blood and peritoneal fluid (PF) levels of syndecan‐1, heparin sulfate (HS) and hyaluronan are elevated in horses with small intestinal disease compared to their healthy counterparts.

**Animals:** Twelve horses, grouped as healthy (H; 4), small intestinal inflammation (SII; 4), or small intestinal strangulating (SIS; 4).

**Methods:** In an ongoing prospective study, paired PF and blood samples were obtained from each group and analyzed via equine validated ELISA for syndecan‐1, HS and hyaluronan concentrations.

**Results:** Hyaluronan was significantly increased in the blood (*P* = .0162) and PF (*P* = .0024) of SI disease compared to H horses. Hyaluronan and HS were significantly increased in the blood of SII compared to SIS horses (*P* = .028 and *P* = .028, respectively). Syndecan‐1 was significantly increased in the PF of horses with SIS (*P* = .038).

**Conclusions:** Based on preliminary findings, EG components are elevated in horses with SI disease and may have utility in differentiating horses with SII and SIS disease.

## ABSTRACT E48: Changes in peritoneal fluid associated with colitis in adult horses

### Kate L. Hepworth‐Warren^1^; Breanna Sheahan^2^

#### ^1^North Carolina State University, College of Veterinary Medicine, Raleigh, NC, USA; ^2^North Carolina State University, Raleigh, NC, USA

**Background:** Colitis can cause severe colic in horses and may not cause diarrhea, making it difficult to distinguish from surgical lesions. Additionally, bacterial translocation secondary to reduced mucosal integrity can lead to secondary peritonitis.

**Objectives:** To identify changes in peritoneal fluid secondary to colitis, and to identify the percentage of positive cultures from peritoneal fluid.

**Animals:** 27 adult horses (>2 years of age) with colitis.

**Methods:** Prospective clinical study enrolling horses hospitalized with colitis. Initial laboratory data were recorded including peripheral WBC and neutrophil count, lactate, albumin, glucose and creatinine. Peritoneal lactate and protein were measured and fluid was submitted for culture, cell count, and cytology. Variables were compared between survivors and non‐survivors using a Mann‐Whitney test (*P* ≤ .05). Previously established reference ranges (Brownlow et al. 1981) for peritoneal cell count and protein were compared with colitis cases using unpaired t tests.

**RESULTS/FINDINGS:** 20/27 (74.1%) horses survived to discharge. Median peritoneal cell count was 2.0 × 10^3^ cells/μL (range 0.3‐205.3 × 10^3^ cells/μL). Median peritoneal lactate was 1.9 mmol/L (range 0.4‐14.7 mmol/L). Median peritoneal protein was 2.6 g/dL (range 0‐5.6 g/dL) and median peripheral lactate was 1.4 mmol/L (range 0.6‐13.3 mmol/L). 6/22 (27.3%) peritoneal fluid cultures yielded positive bacterial growth. Peritoneal protein was increased compared to the normal reference range. There were no significant differences between survivors and non‐survivors.

**Conclusions and Clinical Importance:** Increased protein can be present in peritoneal fluid from colitis cases which is not related to survival. Positive bacterial cultures were obtained in several horses but did not influence the outcome.

## ABSTRACT E49: Fecal microbiota dynamics throughout hospitalization in horses with different types of colic

### **Clemence Loublier** ^1^; Hélène Amory^1^; Carla Cesarini^1^; Georges Daube^1^; Laureline Lecoq^1^; Marcio Costa^2^

#### ^1^University of Liège, Liège, Belgium; ^2^University de Montreal, Montreal, QC, Canada

**Background:** Publications assessing changes in fecal microbiota during hospitalization of horses with colic are in their early stages.

**Objectives:** To investigate the dynamics of fecal microbiota during hospitalization of horses with different types of colic, and its association with outcome (mortality).

**Animals:** Horses hospitalized for more than 5 days for different types of colic: inflammatory (INFL), simple (SIMPLE) and strangulated obstructions (STR).

**Methods:** A prospective observational study was carried out, with fecal samples collected on days 1 (admission), 3 and 5 of hospitalization. Bacterial taxonomy profiling was obtained by DNA sequencing (at the genus level). Data were statistically compared between groups (2‐way ANOVA) and LEfSE analysis to identify bacteria significantly different between groups (*P* < .05).

**Results:** 23 horses (9 INFL, 9 SIMPLE, 5 STR) were included. INFL group (all presenting diarrhea) had greater richness (*P* = .0075) and diversity (Shannon, *P* = .0001) than other colic types on day 5, but no differences were found during hospitalization within each colic group. Compositional analysis of bacterial membership was significantly different in the INFL group in regard to SIMPLE and STR (*P* < .001). Treponema was more abundant in the INFL group and unclassified *Acidaminococcaceae* in the SIMPLE group. Furthermore, beta diversity membership was statistically different in survivors (*P* = .001). Increased relative abundances of *Bacilliculturomica* and *Saccharofermentans* were associated with survival.

**Conclusions:** The fecal microbiota of horses with colic seems more influenced by the nature of the digestive disease than by time of hospitalization. Further studies are necessary to predict severity and mortality of horses with colic.

## ABSTRACT E51: Preliminary assessment of the leukocyte coping capacity as stress‐marker in horses with various diseases

### **Dagmar S. Trachsel** ^1^; Nikolaus Huber^2^, DrMedVet; Fatma Graiban Almheiri^3^; Karolína Bábor^4^, MVDr; Vendula Jandová^5^, MVDr

#### ^1^University of Veterinary Medicine, Vienna, Vienna, Austria; ^2^Department for Farm Animals and Veterinary Public Health, University of Veterinary Medicine, Vienna, Vienna, Austria; ^3^Central Veterinary Research Laboratory, Dubai, United Arab Emirates; ^4^Equine Veterinarian; ^5^Equine Internal Medicine Practice

**Background:** Stress represents a serious health and welfare concern as it might induce diseases such as the equine gastric ulcer syndrome (EGUS). Moreover, chronic diseases as orthopedic diseases that cause chronic pain can be a source of stress. The leucocyte coping capacity (LCC) quantifies the capacity of neutrophil granulocytes to produce oxygen radicals (OR). In chronic stress or pain, the capacity of the neutrophils to produce OR is reduced. Therefore, measuring the LCC could be a novel marker for chronic stress in horses.

**Hypothesis/Objectives:** Horses with diseases leading to or caused by stress will have lower LCC than healthy horses.

**Animals:** 46 privately owned horses presented to an ECEIM specialist for gastroscopy.

**Methods:** In this clinical study horses were classified according to the most relevant clinical diagnosis based on clinical, laboratory and gastroscopic findings in following groups: (1) healthy, (2) lame, (3) EGUS, (4) other diseases. The LCC was measured every 5 min over 90 min by a portable chemiluminometer and the values compared among groups with linear mixed effect models for repeated measurements.

**Results:** Results from our models indicate that lame horses had significantly (*P* = .012) lower values for LCC than healthy horses. For horses in the groups EGUS or other diseases the models showed no significant difference (*P* > .05) for the LCC values compared to healthy horses.

**Conclusions and Clinical Importance:** The LCC might be an indicator for stress especially in diseases where the stress is related to chronic pain as associated with chronic lameness.

## ABSTRACT E52: Delayed, severe rhabdomyolysis following uncomplicated anesthesia in six warmblood horses

### **Kate L. Hepworth‐Warren** ^1^; Charlene Knoll^2^; Ana Moreira^3^; Toby Pinn‐Woodcock^4^; Megan Ballou^5^; Kirby Penttila^6^; Dayna Goldsmith^7^; Katarzyna Dembek^5^; Stephanie Valberg^3^

#### ^1^North Carolina State University, College of Veterinary Medicine, Raleigh, NC, USA; ^2^Woodside Equine Clinic, Ashland, VA, USA; ^3^Michigan State University, East Lansing, MI, USA; ^4^Cornell University, Ithaca, NY, USA; ^5^North Carolina State University, Raleigh, NC, USA; ^6^Burwash Equine Services, Calgary, AB, Canada; ^7^University of Calgary, Calgary, AB, Canada

**Background:** Warmblood horses have not been reported to be at high risk for post‐anesthetic myopathy.

**OBJECTIVES:** Document the clinical presentation of severe rhabdomyolysis following uncomplicated anesthesia of 6 Warmblood horses with no history of myopathy.

**Animals:** 6 Warmbloods, 5 geldings, 1 mare, mean age 10 years (range 4‐9 years), weight 613 kg (range 550‐703 kg).

**Methods:** Records were reviewed and pre‐ and post‐anesthetic bloodwork, management, muscle histopathology and post‐mortem results compiled.

**Results:** Severe rhabdomyolysis developed following uncomplicated anesthesia for exploratory celiotomy (3), cervical imaging (1), arthroscopy (1), and intraarticular injection (1). One horse never stood after anesthesia and a second stood but collapsed the following day. At time of euthanasia both horses had hyperlactatemia (12 and 21 mmol/L) and the latter had markedly increased creatine kinase (CK) activity (32 559 U/L). Acute myodegeneration and recumbency developed 7 (CK 193273 U/L), 9 (CK >14 000) and 10 days following anesthesia with prolonged recoveries in 3 horses that culminated in euthanasia. Macrophages, lymphocytes (2) and severe glycogen depletion (1) were evident in skeletal muscle. The surviving horse had prolonged recovery with rhabdomyolysis (CK 29800 U/L) evident 3 days post‐anesthesia and focal myofibers had glycogen depletion at biopsy 13 days post‐anesthesia. Histopathologic evidence of chronic myopathy (internalized myonuclei in mature fibers) was present in two horses.

**Conclusions and Clinical Importance:** Severe rhabdomyolysis can occur in Warmblood horses with no history of myopathy up to 10 days after anesthesia. Fulminant anaerobic glycolysis during anesthesia could have caused the noted glycogen depletion and lactic acidosis.

## ABSTRACT E54: Physical restraint for veterinary procedures—efficacy and welfare effects on horses

### **Claire O'Brien** ^1^; Sharanne Raidal^2^, BVSc, MVSt, PhD, FANZCVS, DECEIM; Olivier Simon^3^, DVM, DECVS, MANZCVS; Gustavo Ferlini Agne^4^, DVM, MAVS, DACVIM; Sam Franklin^5^, BVSc, PhD, FRCVS, DACVSMR; Sara Weaver^6^, BSc, PhD

#### ^1^Charles Sturt University, NSW, Australia; ^2^Professor of Equine Medicine, Veterinary Clinical Centre, Charles Sturt University, NSW, Australia; ^3^Senior Lecturer in Equine Surgery, School of Animal and Veterinary Science, The University of Adelaide, SA, Australia; ^4^Senior Lecturer in Equine Medicine, School of Animal and Veterinary Science, The University of Adelaide, SA, Australia; ^5^Professor, School of Animal and Veterinary Science, The University of Adelaide, SA, Australia; ^6^Research Assistant, School of Animal and Veterinary Science, The University of Adelaide, SA, Australia

**Background:** Physical restraint of horses for veterinary procedures is necessary to allow completion of tasks safely, effectively and without injury to patient or personnel. The restraint used should be of minimal welfare cost to the horse. In the context of veterinary procedures, patient wellbeing is optimized when procedures can be completed efficiently, with minimal distress to the animal.

**Objectives:** This study was designed to compare the physiological effects and efficacy of three commonly used restraint techniques (Figure 1) for upper airway endoscopy in unsedated horses.

**Animals:** 12 university owned teaching horses.

**Methods:** Blocked and randomized interventional study. Horses were subjected to a routine endoscopic assessment of laryngeal function on four occasions and were allocated to each of four restraint methods (nose twitch, ear hold, lip rope, nil) in random order, with 48 hours between interventions. Outcome measures included behavioral scoring, subjective and objective measures of procedural efficacy, heart rate variability, cortisol and β‐endorphin concentrations.

**Results:** Horses demonstrated strong individual differences for procedure tolerance and preferred method of restraint. Subjective procedure scores and objective measures of head movement (Figure 2) were significantly lower with use of a nose twitch, compared to nil restraint.

**Conclusions and Clinical Importance:** There was no evidence that any of the restraint types investigated had a different effect on welfare or wellbeing based on behavioral or physiological outcome variables evaluated in the current study. Use of a nose twitch provided the most effective restraint, but individual horse preferences should influence the method selected.

**Figure:**
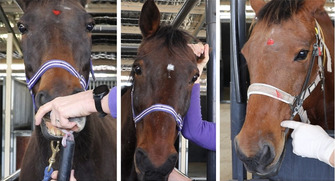
**Figure 1**. Restraint methods evaluated in the current study: lip twitch (left), ear hold (centre) and lip rope (Stableizer).

**Figure:**
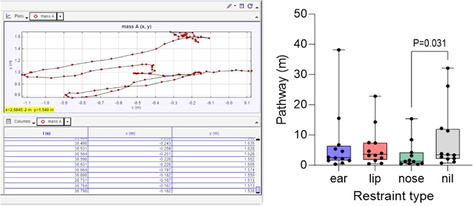
Figure 2.

Representative trace from motion tracking software used to obtain objective measurement of head movement during procedure (left), and results for all restraint types (right). Results are shown as the total distance traveled by horse's head during procedure, with data presented as median (horizontal line), quartiles (box) and range (whiskers), with all results shown. A significant restraint effect was observed, as shown.

## ABSTRACT E55: Tolerance of binasal prongs for delivery of non‐invasive ventilation to foals

### **Sharanne L. Raidal** ^1^; Melanie Catanchin^2^, BVetBiol, BVetSc (Hons 1), GradDipEd (Tertiary); Heidi Lehmann^3^, BVMS, DACVAA; Chris Quinn^3^, BSc (Vet, Hons), BVSc (Hons), MANZCVS, DACVAA, DECVAA; Michael van Diggelen^4^, BVSc (Hons)

#### ^1^School of Agricultural, Environmental and Veterinary Sciences, Charles Sturt University, NSW, Australia; ^2^Lecturer in Veterinary Anesthesia, School of Agricultural, Environmental and Veterinary Sciences, Charles Sturt University, NSW, Australia; ^3^Senior Lecturer in Veterinary Anesthesia, Veterinary Clinical Centre, Charles Sturt University, NSW, Australia; ^4^Veterinarian, School of Agricultural, Environmental and Veterinary Sciences, Charles Sturt University, NSW, Australia

**Background:** Non‐invasive ventilation (NIV) is a method of providing respiratory support without the need for airway intubation. NIV can be delivered by helmet, mask or nasal prongs. To date, studies in foals have used mask delivery, but the mask is poorly tolerated and associated with hypercapnia, possibly associated with the accumulation of carbon dioxide within equipment dead space and/or with expiratory flow limitations.

**Objectives:** This study was conducted to evaluate the tolerance of prototype binasal prongs (Figure 1) in healthy unsedated foals, and following light sedation.

**Animals:** Six healthy foals, <1 week old.

**Methods:** Observational behavior study with NIV delivered at incremental pressures. Tolerance of binasal prongs was assessed using an ethogram to document behavior in unsedated foals, and subsequently following light sedation (diazepam 5 mg IV). Thereafter, foals were subjected to NIV at incremental pressure support and peak end‐expiratory pressure for sequential two‐minute intervals.

**Results:** All foals tolerated NIV through binasal prongs, although increasing airway pressures were associated with increased inspiratory volume, duration of inspiration and air leakage in most foals. These changes preceded discontinuation/intolerance of NIV on the basis of behavior changes consistent with discomfort. Increased circuit leakage was associated with reduced return of expired air to the ventilator and increasing disparity between inspiratory and expiratory times and tidal volumes.

**Conclusions and Clinical Importance:** Binasal prongs were well tolerated and might be suitable for NIV, but design and fitting require further optimization. Behavior and ventilator variables should be monitored to predict patient tolerance of NIV.

**Figure:**
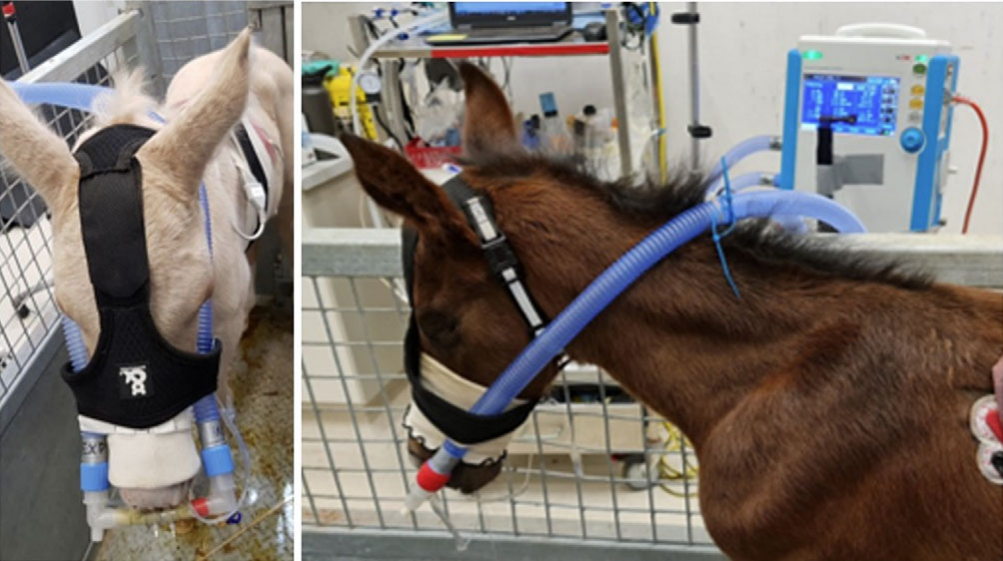
**Figure 1.** Delivery of non‐invasive ventilation to standing, lightly sedated (diazepam 5 mg IV) foals using prototype binasal prongs.

## ABSTRACT E56: Three‐dimensional thoracic electrical impedance tomography of horses during normal and increased tidal volumes

### **David Byrne** ^1^; Nicole Studer^2^, DECVAA; Cristy Secombe^3^, BSc, BVMS, MANZCVS, MVSc (Hons), DACVIM; Alexander Cieslewicz^4^, MSc; Giselle Hosgood^5^, BVSc, MS, PhD, DACVS; Anthea Raisis^6^, BVSc, PhD, DVA, MVClSt, MANZCVSc; Andy Adler^7^, P.Eng.; Martina Mosing^8^, DrMedVet, MANZCVS, DECVAA

#### ^1^Murdoch University, WA, Australia; ^2^Animalius, WA, Australia; ^3^Head of Veterinary and Public Affairs, Australian Veterinary Association; ^4^Morrison Critical Care and Pulmonary; ^5^Professor of Small Animal Surgery, School of Veterinary Medicine, Murdoch University, WA, Australia; ^6^Associate Professor in Veterinary Anesthesia, School of Veterinary Medicine, Murdoch University, WA, Australia; ^7^Research Professor, Systems and Computer Engineering, Carleton University, Ottawa, ON, Canada; ^8^Professor of Veterinary Anesthesia, Anaesthesiology and Perioperative Intensive Care, University of Veterinary Medicine, Vienna, Vienna, Austria

**Background:** Electrical impedance tomography (EIT) is a non‐invasive method of evaluating lung function.

**Objective:** To describe multiplanar reconstruction of two‐plane EIT data and assess correspondence of distribution of ventilation with known anatomical topography.

**Animals:** Twenty clinically healthy research horses.

**Methods:** Two‐plane EIT data were collected in standing sedate horses at baseline (resting) conditions, and during rebreathing. EIT data were reconstructed into 3d EIT whereby tidal impedance variation (TIV), ventilated area, and right‐left (CoV_RL_) and ventral‐dorsal (CoV_VD_) centres of ventilation were calculated in cranial, middle and caudal slices of lung. A two‐factor general linear model was applied with time (baseline and rebreathing), slice and the interaction included as fixed effects. Where significant interaction was found, at *P* < .05.

**Results:** There was a significant interaction of time and slice for TIV (*P* < .0001) with TIV increasing during rebreathing in caudal and middle slices. The ratio of right to left ventilated area was higher in the cranial slice, in comparison to the caudal slice (*P* = .0002). There were significant effects of time and slice on CoV_VD_ whereby the cranial slice was more ventrally distributed than the caudal slice (*P* < .0009 for the interaction).

**Conclusions and Clinical Importance:** The distribution of ventilation in the three slices corresponds with topographical anatomy of the equine lung. This study confirms that 3d EIT represents lung anatomy and changes in ventilation distribution during increased tidal volume breathing in standing sedate horses.

## ABSTRACT EN01: Epidemiology of diabetes mellitus in cats under british primary veterinary care in 2019

### **Oliver Waite**; Ruth Gostelow, BVetMed (Hons), DACVIM, DECVIM‐ca, MVetMed, PhD, PGCertVetEd, FHEA, MRCVS; Rosanne Jepson, BVSc, MVetMed, PhD, DACVIM, MRCVS; Dan O'Neill, MVB, BSc (Hons), MSc (VetEpi), PhD, FRCVS; Emma Wright, BVMedSci (Hons), BVM, BVS (Hons), PGDiPVC, MRCVS

#### Royal Veterinary College, Hatfield, England, UK

**Background:** Diabetes mellitus (DM) is a common feline endocrinopathy, but limited recent epidemiological data exists.

**Hypothesis/Objectives:** Describe the incidence, prevalence, and risk factors for DM among cats under British primary veterinary care (PVC).

**Animals:** Sample of 1053 DM cases from a 1 255 130 cat cohort under British PVC in 2019.

**Methods:** Cross‐sectional analysis of anonymised electronic patient records from VetCompass. The 2019 period prevalence and incidence risk of DM were calculated. Risk factor analysis used multivariable logistic regression modeling.

**Results:** One‐year period prevalence of DM was 0.39% (95% confidence interval [CI]: 0.37‐0.42]). Annual incidence risk was 0.14% (95% CI: 0.13‐0.16). Mean age at DM diagnosis was 11.8 years (SD ± 3.5, n = 371). Median adult bodyweight: 5.93 kg (IQR; 4.57‐711; range: 1.50‐14.45 kg); 63.15% (665/1053) were male and 853/1053 (81.00%) of cats were neutered. Analyzed risk factors for DM are shown in Table 1. Burmese and Burmilla cats had increased odds of DM when compared to crossbred cats; OR: 2.07 (CI: 1.29‐3.31) and OR: 8.30 (CI: 2.59‐26.6), respectively.

**Conclusions and Clinical Importance:** An estimated 0.39% of cats under British PVC are affected by DM. Burmillas are identified at increased risk of DM for the first time, possibly due to descent from Burmese cats. Bodyweight as a diabetic risk factor in relation to breed standard and variation requires further investigation.

**Table:** **Table 1.** Final multivariable logistic regression results in cats with DM in the VetCompass database under PVC in the UK from January 1‐December 31, 2019

Variable	Category	Odds ratio	95% CI	*P* value
Age at diagnosis category (y)	4.5‐9.0	Base		
	0.0‐4.5	0.12	0.08‐0.20	<.001*
	9.0‐13.5	5.11	4.19‐6.24	**<.001**
	13.5‐18.0	9.48	7.79‐11.53	**<.001**
	>18.0	4.77	3.33‐6.83	**<.001**
	Unrecorded	1.12	0.45‐2.78	.814
Sex	Female	Base		
	Male	1.97	1.73‐2.24	**<.001**
	Unrecorded	1.77	0.87‐3.59	.115
Bodyweight (kg)	4.0‐4.9	Base		
	0.0‐2.9	0.57	0.41‐0.80	.001*
	3.0‐3.9	0.84	0.64‐1.10	.195
	5.0‐5.9	1.14	0.91‐1.43	.266
	6.0‐6.9	1.39	1.11‐1.75	**.004**
	7.0‐7.9	1.10	0.84‐1.44	.500
	≥8.0	0.86	0.68‐1.09	.221
	Unrecorded	0.47	0.38‐0.59	<.001
Neuter status	Neutered	Base		
	Entire	0.85	0.72‐1.00	.049*

Variables associated with an increased odds of DM highlighted in bold, variables associated with a reduced odds of DM with *.

## ABSTRACT EN02: Steroid profiles in cats with primary hyperaldosteronism

### **Alice H. Watson** ^1^; Lorna Gilligan^2^; Angela Taylor^2^; Wiebke Arlt^2^; Harriet Syme^1^

#### ^1^Royal Veterinary College, London, UK; ^2^University of Birmingham, Birmingham, UK

**Background:** Liquid chromatography tandem mass spectrometry (LCMS) has been used to improve understanding of the underlying pathophysiology of human endocrinopathies. Multiple steroid excesses have been identified in cats with primary hyperaldosteronism (PHA).

**Hypothesis/Objectives:** Cats with PHA will have alteration in other steroid hormones, in addition to aldosterone.

**Animals:** Residual serum samples from cats with overt primary hyperaldosteronism (PHA, n = 6), and control cats with high (n = 6) or low/normal aldosterone (n = 15) measured by radioimmunoassay.

**Methods:** Case‐control study. Blood samples were analyzed for 20 steroids by LCMS. Kruskal Wallis one way ANOVA with Benjamini and Hochberg correction compared the three groups. Variables that were significant (*P* < .05) were followed up with post hoc pairwise Wilcoxon sign rank‐sum tests.

**Results:** 11 steroids were significantly different between groups (Table 1). Cats with PHA had lower glucocorticoid (including 17‐hydroxyprogesterone, 11‐deoxycortisol and cortisol), pregnenolone and testosterone and higher dihydrotestosterone and 5α‐dihydroprogesterone concentrations than the low/normal aldosterone group. Pregnenolone concentrations were also lower in the PHA group than the high aldosterone group. The high aldosterone group had lower dihydrotestosterone and 5α‐dihydroprogesterone and higher progesterone, mineralocorticoids (deoxycorticosterone, corticosterone), glucocorticorticoids (17‐hydroxyprogesterone, 11‐deoxycortisol) and testosterone than the low/normal aldosterone group.

**Conclusions and Clinical Importance:** Cats with PHA had lower concentrations of glucocorticoids compared with cats with normal aldosterone, whilst cats with high aldosterone had higher glucocorticoids than the cats with normal aldosterone, suggesting differences in the underlying pathophysiology. Marked progesterone excess was not observed in the PHA group. Measurement of multiple steroids may be useful to investigate other endocrinopathies.

**Table:** **Table 1.** Data in the table are median and interquartile range in nanomoles per liter with adjusted *P* values from Kruskal Wallis one way ANOVA with Benjamini and Hochberg repeated comparisons correction

	Low/Normal	High	PHA	*P* value
Aldosterone	0.15 [0.09‐0.22]	0.66 [0.46‐0.76]	3.01 [2.36‐18.95]	.001
Deoxycorticosterone	0.58 [0.28‐1.25]	2.32 [1.68‐3.88]	2.19 [0.70‐2.73]	.02
Corticosterone	9.1 [6.2‐19.1]	35.7 [30.1‐41.6]	16.6 [7.5‐34.5]	.02
Progesterone	1.1 [0.7‐1.4]	1.8 [1.5‐2.2]	0.9 [0.4‐1.3]	.02
17‐hydroxyprogesterone	0.17 [0.12‐0.24]	4.9 [0.33‐0.61]	0.08 [0.08‐0.16]	.014
11‐deoxycortisol	0.8 [0.2‐1.0]	1.6 [1.5‐1.8]	0.3 [0.1‐0.5]	.02
Cortisol	135 [90‐175]	205 [173‐236]	69 [53–87]	.022
Pregnenolone	7.3 [5.1‐8.6]	10.8 [10.4‐14.5]	1.5 [1.1‐2.2]	.015
Testosterone	ND	0.1 [0.1‐0.03]	ND	.02
Dihydrotestosterone	0.03 [0.01‐0.04]	ND	0.05 [0.03‐1.19]	.015
5α‐dihydroprogesterone	1.3 [0.7‐2.7]	ND	0.77 [0.43‐1.21]	.015

Low/normal, low/normal aldosterone on radioimmunoassay; high, high aldosterone on radioimmunoassay; PHA, primary hyperaldosteronism; ND, non‐detectable.

## ABSTRACT EN03: Long‐term safety and efficacy of oral bezafibrate use in dogs with hypertriglyceridemia

### **Marilou Castonguay‐Poirier**; Lyanne Fifle; Romain Huvé; Romain Javard

#### DMVet Veterinary Center, Laval, QC, Canada

**Background:** Bezafibrate (BZF) is effective for the treatment of hypertriglyceridemia in dogs, but there is limited data on its use in the long term.

**Objectives:** To assess the long‐term safety and efficacy of BZF in controlling primary and secondary hypertriglyceridemia in dogs.

**Animals:** 55 client‐owned dogs with hypertriglyceridemia.

**Methods:** Retrospective study. Dogs were treated with BZF at a mean dose of 6 mg/kg [2‐12 mg/kg] and categorized into 3 groups: primary hypertriglyceridemia (PH), and secondary hypertriglyceridemia with stable (SHs) or unstable (SHu) treatment. Serum triglycerides (TG), creatine kinase (CK), and alanine aminotransferase (ALT) were recorded before treatment (T0) and at subsequent follow‐ups (1, 3, 6, 12, and >18 months, as available). Treatment response was classified as complete [TG < 221 mg/dL], partial [TG between 221 and 443 mg/dL or (decreased by >50% T0 and TG > 221 mg/dL)] or no response [TG > 443 mg/L and decreased by <50% T0].

**Results:** All groups showed a significant decrease in TG concentration between the baseline (T0) and the last available value (*P* < .01). The median TG decrease over the study period was 85%, all groups combined. Adverse effects consisted of mild gastrointestinal signs in 2 dogs and a significant increase in ALT (>3× higher reference range) in 1 dog after 6 months of treatment.

**Conclusion and Clinical Importance:** Over the study period, BZF was overall safe and effective in the long term for most dogs with primary and secondary hypertriglyceridemia.

vim24_608

## ABSTRACT EN04: Evaluation of a feline‐optimized TSH assay in cats with hyperthyroidism and with non‐thyroidal illness

### **Camille Brassard** ^1^; Stefanie DeMonaco^2^ , DVM, MS, DACVIM (SAIM); Ashley Wilkinson^3^, DVM, MS, DACVIM (SAIM)

#### ^1^Virginia‐Maryland Regional College of Veterinary Medicine, Blacksburg, VA, USA; ^2^Associate Professor, Veterinary Clinical Sciences, Long Island University, Brooklyn, NY, USA; ^3^Associate Professor, Small Animal Clinical Sciences, Virginia‐Maryland Regional College of Veterinary Medicine, Blacksburg, VA, USA

**Background:** Since approximately 10% of hyperthyroid cats have a normal TT4, TSH may be needed to confirm the diagnosis. Until recently, only a canine TSH assay (cTSH) was available, which cannot differentiate between subnormal and low‐normal TSH concentrations in cats. A novel feline‐optimized TSH assay (fTSH, Truforma Zomedica) differentiates better between euthyroid and hyperthyroid cats, but the effect of non‐thyroidal illness (NTI) is unknown.

**Hypothesis/Objectives:** We aim to compare fTSH and cTSH concentrations among hyperthyroid, NTI, and healthy cats, and to evaluate the sensitivity and specificity of fTSH to diagnose hyperthyroidism.

**Animals:** The study enrolled 102 client‐owned cats, including 37 hyperthyroid, 33 healthy, and 32 NTI cats.

**Methods:** Prospective cross‐sectional study. TT4, cTSH, and fTSH were measured in all cats. Hyperthyroidism was confirmed with thyroid scintigraphy. TSH was compared among groups using Kruskal Wallis followed by Wilcoxon pairwise method. Significance set at *P* < .05.

**Results:** The sensitivity and specificity of fTSH are 78% (95% CI 62%‐90%) and 97% (84%‐100%), respectively. There is a significant difference between hyperthyroid cats and healthy and NTI cats with both assays (*P* < .01).

**Conclusion:** The fTSH is useful to diagnose feline hyperthyroidism because it has a high specificity, identifies normal TSH in healthy cats more often, and appears to not be affected by NTI.

## ABSTRACT EN05: Plasma arginine vasopressin and serum copeptin concentrations under hypo‐, iso‐ and hyper‐osmolar conditions

### **Mathieu Victor Paulin** ^1^; Suraj Unniappan^2^, PhD; Elisabeth Snead^3^, BSc., DVM, MSc, DACVIM (SAIM)

#### ^1^Department of Small Animal Clinical Sciences, Western College of Veterinary Medicine, University of Saskatchewan, Saskatoon, SK, Canada; ^2^Professor, Centennial Enhancement Chair in Comparative Endocrinology, Department of Veterinary Biomedical Sciences, Western College of Veterinary Medicine, University of Saskatchewan, Saskatoon, SK, Canada; ^3^Professor, Small Animal Internal Medicine, Department of Small Animal Clinical Sciences, Western College of Veterinary Medicine, University of Saskatchewan, Saskatoon, SK, Canada

**Background:** Serum copeptin (sCoP), a glycopeptide comprising the C‐terminal part of arginine vasopressin (AVP) prohormone, has mostly replaced the measurement of plasma arginine vasopressin (pAVP) in humans for the clinical investigation of specific polyuria‐polydipsia disorders.

**Objectives:** To investigate sCoP as a potential surrogate of pAVP measurement.

**Animals:** 9 young adult dogs from a research colony.

**Methods:** Prospective longitudinal study. Serum CoP and pAVP were measured under iso‐ (baseline), hypo‐ (water load test [WLT]), and hyper‐ (modified water deprivation test [MDWT]) osmolar conditions. In the WLT, 9 dogs were given 44 mL/kg of warmed tap water via nasogastric tube over 30 minutes, followed by 0.3 ug/kg IV CRI of desmopressin acetate over 20 minutes; pAVP was collected 2 hours after and sCoP 4 hours after tap water administration. In the MDWT, sCoP and pAVP were measured in 8 dogs after either ≥5% body weight loss or a fasting period ≥48 hours.

**Results:** Two dogs were excluded from sCoP analyses since serum samples had marked hemolysis, which interfered with optical density readings. Median pAVP was significantly lower after WLT (19.5 pg/mL) vs. baseline (24.2 pg/mL, *P* = .004). Mean pAVP was significantly higher after MDWT (53.6 pg/mL) vs. baseline (37.9 pg/mL, *P* =0.012). Similarly, mean sCoP was significantly lower after WLT (230 pg/mL) vs. baseline (299 pg/mL, *P* = .035) and significantly higher after MDWT (335 pg/mL) vs. baseline (280 pg/mL, *P* = .029). Correlation between pAVP and sCoP was moderately positive (Pearson r = +0.52, *P* = .019).

**Conclusion and Clinical Importance:** Serum CoP has the potential to be a sensitive surrogate for pAVP measurement in dogs.

## ABSTRACT EN06: Duration of sedation effects on the adrenocorticotrophic hormone stimulation test in healthy dogs

### **Jake Salzman**; Alejandro Esteller‐Vico, DVM, PhD; Luca Giori; Shelly Olin, DVM, DAVIM (SAIM)

#### University of Tennessee, Knoxville, TN, USA

**Background:** Sedation is frequently required in dogs for diagnostic workups and patient safety. Yet, sedation affects serum cortisol levels; butorphanol elevates cortisol, while dexmedetomidine reduces it dose dependently. Understanding the duration of sedation's impact on cortisol concentration is important.

**Hypothesis/Objectives:** To evaluate baseline serum cortisol at time 0 and 6 hours post‐sedation with saline (0.5 mL IV), butorphanol (0.3 mg/kg) and combination of butorphanol and dexmedetomidine (0.3 mg/kg and 5 mcg/kg IV, respectively). Additionally, to compare post‐adrenocorticotrophic hormone (ACTH) cortisol concentration at 7 hours after these sedation protocols.

**Animals:** 12 healthy, castrated, colony beagles.

**Methods:** Randomized, blinded, controlled, repeated‐measure crossover design with a one‐week washout between treatments. Serum cortisol was measured at time 0 and 6 hours post‐sedation with saline, butorphanol, or combination butorphanol and dexmedetomidine. An ACTH‐stimulation test was performed at 6 hours post‐sedation. A mixed model analysis with treatment and time as fixed factors was utilized for significance.

**Results:** Mean serum cortisol concentration was not significantly different at time 0 and 6 hours following saline (1.47; 0.91 μg/dL, respectively; *P* = .69), butorphanol (0.9; 0.95 μg/dL, respectively; *P* = .29), and combination (0.96; 1.62 μg/dL, respectively; *P* = .57). There was no significant difference in post‐ACTH cortisol for saline, butorphanol, or combination treatment (8; 8.66; 8.46 μg/dL, respectively; *P* = .841).

**Conclusions and Clinical Importance:** In healthy dogs following sedation with the aforementioned protocols, cortisol concentration returns to baseline by 6 hours. An ACTH stimulation test started 6 hours post‐sedation is not affected by these drug protocols, which allows for same‐day sedation. Additional studies are needed in dogs with adrenal dysfunction.

## ABSTRACT EN07: Transmucosal glucagon rapidly increases blood glucose concentration in healthy cats

### **Emily A. Cohen** ^1^; Chiquitha Crews^2^, BS, CVT, MS (Microbiology and Biochemistry); Chen Gilor^3^, DVM, DACVIM (SAIM), PhD; Jocelyn Mott^4^, DVM, DACVIM (SAIM); Lauren Porter^5^, DVM; Antonio Tardo^6^, DVM, Resident ECVIM‐CA (IM)

#### ^1^College of Veterinary Medicine, University of Florida, Gainesville, FL, USA; ^2^Biological Scientist II, Department of Small Animal Clinical Sciences, University of Florida, Gainesville, FL, USA; ^3^Associate Professor of Small Animal Internal Medicine, Department of Small Animal Clinical Sciences, University of Florida, Gainesville, FL, USA; ^4^Postdoctoral Associate in Feline and Canine Diabetes Mellitus, University of Florida, Gainesville, FL, USA; ^5^University of Florida, Gainesville, FL, USA; ^6^PhD Student, Veterinary Medical Sciences, Alma Mater Studiorum, University of Bologna, Bologna, Italy

**Background:** Transmucosal glucagon has potential use in at‐home and in‐hospital emergencies to treat life‐threatening hypoglycemia in cats.

**OBJECTIVES:** To evaluate the effect of transmucosal glucagon (Baqsimi™) on blood glucose concentrations (BG) in healthy cats and describe adverse reactions to its administration.

**Animals:** Six healthy, purpose‐bred cats.

**Methods:** Randomized, controlled, crossover study. Transmucosal glucagon was administered intranasally and rectally and compared to intranasal placebo. Blood was collected at −15 and −1 minutes before, and 5, 15, 25, 35, 45 and 60 minutes after glucagon administration for evaluation of BG, plasma glucagon (pGlucagon), and plasma potassium (K+) concentrations. Stress scores and adverse effects were recorded at all time points.

**Results:** pGlucagon in the nasal and rectal groups increased from baseline (median [range]) (12.2 [3.5‐44.1] mmol/L nasal and 6.9 [2.9‐21.1] mmol/L rectal), to 218.5 [7.9‐349.8] mmol/L (*P* = .02) and 349.8 [67.4‐349.8] mmol/L (*P* = .01) respectively, 15 minutes after administration. BG increased from baseline (101 [91‐110] mg/dL) 15 minutes after nasal (137.5 [104‐251] mg/dL, *P* = .006) and rectal (229 [99‐285] mg/dL *P* = .002) administration. K+ decreased from baseline (3.8 [3.6‐4.1] mmol/L nasal and 3.7 [3.5‐3.9] mmol/L rectal) to 3.4 [3.1‐3.6] mmol/L (*P* = .04) at 15 minutes with nasal administration, and with rectal administration, to 3.2 [3.1‐3.6] mmol/L (*P* = .04) at 15 minutes and 3.1 [2.9‐3.4] mmol/L (*P* = .01) at 25 minutes. No significant changes were detected in the placebo group. No serious adverse effects were noted.

**Conclusions and Clinical Importance:** Transmucosal glucagon is effective in raising BG with minimal side effects in healthy cats.

## ABSTRACT EN08: Relationship between hemoglobin A1C and fructosamine concentrations and survival in diabetic dogs

### **Anna L. Kraemer** ^1^; Lily Diamond^2^, BVM, BVS, MRCVS; Ian Ramsey^3^, BVSc, PhD, DSAM, DECVIM, FHEA, FRCVS

#### ^1^University of Glasgow, Glasgow, Scotland, UK; Small Animal Hospital, University of Glasgow, Glasgow, Scotland, UK; ^3^Professor, Internal Medicine, Small Animal Hospital, University of Glasgow, Glasgow, Scotland, UK

**Background:** Hemoglobin A1c (HbA1c) and fructosamine can assist in assessing glycaemic control in dogs with diabetes mellitus (DM). The relationship between these parameters and survival is unknown.

**OBJECTIVE:** To investigate the relationship between HbA1c, fructosamine and survival in dogs with DM.

**Animals:** Forty‐seven client‐owned dogs referred with DM within six months of diagnosis.

**Methods:** Single‐centre retrospective observational study. HbA1c and fructosamine concentrations were measured using previously validated immunoturbimetric and colormetric assays respectively. The averages of values obtained within the first 6 months after the first measurements were calculated. Survival time was defined as survival after the first available measurement. Dogs that were still alive at the time of analysis were censored.

**Results:** There was moderate positive correlation between the average HbA1c and average fructosamine (*r* = 0.609; 95%CI: 0.390‐0.763). Median overall‐survival time for dogs that died during the study period (n = 33) was 366 days (range 0‐1451) with 66.7%, 51.5%, 27.3% and 15.2% of those dogs surviving for 6, 12, 24 and 36 months, respectively. There was no correlation either between the average HbA1c (r = 0.009; 95% CI: −0.335‐0.351) nor the average fructosamine (r = 0.301; 95% CI: −0.047‐0.584) and overall survival. There was also no significant difference in average HbA1c or average fructosamine in dogs being alive or deceased after 6, 12, 24 and 36 months, respectively.

**Conclusions and Clinical Importance:** The results of this study do not support the value of average HbA1c or fructosamine concentrations for predicting survival in this cohort of dogs with DM.

## ABSTRACT EN09: Utilization of glycated hemoglobin as a biomarker to identify and monitor high‐risk groups for diabetes

### **Jeong‐Ho Ha**; Yunho Jeong; Jin‐Ok Ahn; Jin‐Young Chung

#### Kangwon National University Animal Hospital, Kangwon‐do, Republic of Korea

**Background:** In human medicine, hemoglobin A1C (HbA1c) is widely used as a biomarker to monitor diabetic patients. It can also be used as a factor to predict the risk of diabetes for hyperglycemic patients. However, there is a lack of research on the use of HbA1c as a biomarker for screening high‐risk groups for diabetes in the veterinary field.

**Hypothesis/Objectives:** This study was conducted to confirm the clinical usefulness of HbA1c to evaluate the high‐risk group for diabetes.

**Animals:** In total, 60 dogs of different age, sex and various breeds were included.

**Methods:** In this cross‐sectional study, all the dogs were categorized into control and three experimental groups (overweight, elderly, disease [that can contribute to diabetes]). The control group included young and healthy dogs, mainly visited for health checkups or neutering. The HbA1c of all the dogs was estimated and the mean HbA1c concentration of the control and the experimental groups were statistically compared.

**Results:** The level of HbA1c was significantly higher in all experimental groups than control group. The concentration of HbA1c was higher in the overweight group (mean [range]; 3.94 [3.5‐4.3]%; *P* < .001) and the elderly group (3.94 [3.5‐4.3]%; *P* < .001) and the disease group (3.99 [3.5‐4.9]%; *P* < .001) than the control group (3.53 [2.9‐3.8]%).

**Conclusions and Clinical Importance:** In the veterinary field, as in human medicine, HbA1c can be used to evaluate high‐risk groups for diabetes.

## ABSTRACT EN10: Evaluation of serum microRNA‐375 concentration in dogs with diabetes mellitus

### **Jayeon Park**; Hyeongyeong Lee; Dohee Lee; Yeon Chae; Youngjae Yoo; Dongheon Shin; Jiseong Woo; Byeong‐Teck Kang; Taesik Yun; Hakhyun Kim

#### Laboratory of Veterinary Internal Medicine, College of Veterinary Medicine, Chungbuk National University, Ch'ungch'ong‐bukto, Republic of Korea

**Background:** Serum microRNAs (miRNAs) serve as diagnostic and prognostic biomarkers for various diseases. The serum concentration of miRNA‐375 (miR‐375), which is abundantly expressed in pancreatic islet cells, is increased in dogs with experimentally induced pancreatic injury and naturally occurring acute pancreatitis. However, this has not been reported in dogs with diabetes mellitus (DM).

**Objectives:** To compare the expression of serum cfa‐miR‐375 between dogs with DM and healthy dogs and examine changes in serum cfa‐miR‐375 levels after insulin administration in dogs with DM.

**Animals:** Twenty dogs with DM and 18 healthy dogs.

**Methods:** This cross‐sectional study evaluated the relative expression of serum cfa‐miR‐375 using reverse transcription and real‐time PCR. The primary endpoint was the comparison of serum cfa‐miR‐375 expression between dogs with DM and healthy dogs. Prospective cohort study.

**Results:** The mean ± SD (SD) fold change (FC) of serum cfa‐miR‐375 was significantly higher (*P* = .048) in dogs with DM (2.30 ± 2.018) than in healthy dogs (1.294 ± 0.560). The FC of serum miR‐375 was significantly increased (*P* = .01) after treatment (4.017 ± 2.054) than before treatment (2.322 ± 2.608) in dogs with DM. The percentage change in cfa‐miR‐375 levels was positively correlated with the concentration of serum fructosamine post‐treatment (r = 0.62, *P* = .01).

**Conclusions and Clinical Importance:** Serum cfa‐miR‐375 is a potential biomarker for the diagnosis and treatment of canine DM. Additionally, increased serum miR‐375 levels may be associated with direct leakage from the damaged pancreas and pathological glucose regulation in canine DM.

## ABSTRACT EN11: Evaluating efficacy through individualized dosing adjustment of modified radioiodine therapy in feline hyperthyroidism

### **Yeon Chae** ^1^; Jiseong Woo^2^; Dongheon Shin^2^; Taesik Yun^2^; Hakhyun Kim^2^; Byeong‐Teck Kang^2^

#### ^1^Chungbuk University, Ch'ungch'ong‐bukto, Republic of Korea; ^2^Laboratory of Veterinary Internal Medicine, College of Veterinary Medicine, Chungbuk National University, Ch'ungch'ong‐bukto, Republic of Korea

**Background:** Radioiodine therapy is crucial for management of feline hyperthyroidism, targeting hyperactive thyroid cells. Efforts to improve the appropriate administration of radioiodine doses have been studied in cats with hyperthyroidism for decades.

**Hypothesis/Objectives:** The objective of this study was to evaluate the efficacy and safety of individualized dosing adjustment in radioiodine therapy for feline hyperthyroidism, and comparing the outcomes with those achieved through a conventional scoring system.

**Animals:** Forty‐one cats were treated with radioiodine based on individualized dosing adjustment, while 18 cats received radioiodine treatment with a previously reported scoring system.

**Methods:** This study was designed as a comparative cohort study. The individualized dosing adjustment involved the following three‐step process: main staging for incorporating scintigraphy findings with serum T_4_ concentrations, sub‐staging for severity of clinical signs, and dose adjustments based on individual condition. Cats treated using the classic scoring system were administered radioiodine based on a previously reported study.

**Results:** Both treatment groups demonstrated a significant decrease in total T_4_ levels over time (F[4, 136] = 184.2, *P* < 0.001), with no significant differences between groups or interaction effects.

**Conclusions and Clinical Importance:** Individualized dosing adjustment demonstrated efficacy similar to the conventional scoring system in treatment for feline hyperthyroidism. This newly designed treatment method could be valuable for optimizing therapeutic outcomes for radioiodine therapy for feline hyperthyroidism.

## ABSTRACT EN12: Assessing a third‐generation flash glucose monitoring system in nondiabetic dogs with rapidly induced hypoglycemia

### **Carly Patterson** ^1^; Jonathan Lidbury^2^, DVM, PhD, DACVIM (SAIM); Shannon Washburn^3^, DVM, PhD

#### ^1^Texas A&M University, College Station, TX, USA; ^2^Associate Professor, Small Animal Clinical Sciences, Texas A&M University, College Station, TX, USA; ^3^Clinical Professor, Veterinary Physiology and Pharmacology, Texas A&M University, College Station, TX, USA

**Background:** Flash glucose monitoring systems (FGMS) are deployed to monitor diabetic dogs. The newest generation FGMS (FreeStyle Libre3, Abbott) contains the world's smallest glucose sensor pairing with a smartphone app via Bluetooth.

**OBJECTIVE:** To assess the utility of a third‐generation FGMS in nondiabetic dogs during rapidly induced hypoglycemia.

**Animals:** Twenty‐three apparently healthy teaching dogs.

**Methods:** Prospective observational study. FGMS (FreeStyle Libre3, Abbott) were placed on each dog. Regular insulin was administered intravenously and hypoglycemia corrected. Prior to insulin administration and every 10 minutes over a 90‐minute period, serial measurements of interstitial glucose (IG) and blood glucose (BG) were made with a FGMS and a portable blood glucose meter (PBGM; AlphaTrak 3, Zoetis), respectively. Readings of both FGMS and PBGM were compared to chemistry analyzer BG concentrations as reference standard. Analytical and clinical accuracy were assessed.

**Results:** The FGMS did not obtain an IG concentration for 38.6% (89/230) of possible measurements. For measurements with a reference standard BG <100 mg/dL, the FGMS and PBGM were within ±15 mg/dL for 27.9% (24/86) and 51.1% (97/190), respectively. The proportions of readings for the FGMS and PBGM which were not likely to affect clinical outcome were 53.9% (76/141) and 79.6% (179/225), respectively.

**Conclusions and Clinical Importance:** The relatively high number of times the FGMS did not obtain an IG measurement could have been due to multiple phones operating as readers in the same area. In this model of rapidly induced hypoglycemia there was limited agreement between the FGMS and reference standard BG measurements.

## ABSTRACT EN13: Efficiency and safety evaluation of thyroid scintigraphy using small‐field‐of‐view gamma camera in normal cats

### **Taesik Yun** ^1^; Sijin Cha^1^; Yeon Chae^1^; Dongheon Shin^1^; Jinyeong Park^1^; Hakhyun Kim^1^; Sang‐Myeong Lee^2^, DVM, MS, PhD; Mhan‐Pyo Yang^1^; Byeong‐Teck Kang^1^

#### ^1^College of Veterinary Medicine, Chungbuk National University, Ch'ungch'ong‐bukto, Republic of Korea; ^2^Chungbuk National University, Ch'ungch'ong‐bukto, Republic of Korea

**Background:** There is no reporting on efficiency and safety of thyroid scintigraphy with small‐field‐of‐view (SFOV) gamma camera in veterinary medicine.

**Objectives:** To evaluate the efficiency and the radiation safety of SFOV gamma camera for feline thyroid scintigraphy.

**Animals:** Ten healthy cats were used for the study.

**Methods:** Randomized controlled trials. Three staffs participated in this study (operator, staff 1, and staff 2). The operator administered either 2 mCi or 4 mCi of technetium pertechnetate intravenously. At 20, 40, and 60 minutes later injection, thyroid images were obtained with varied acquisition conditions (100 000 counts, 150 000 counts, 200 000 counts, 30 seconds, and 60 seconds). The images were analyzed by calculating thyroid‐to‐salivary ratio (TSR) and thyroid‐to‐background ratio (TBR). Surface and ambient radiations were measured hourly from immediately after the injection up to 6 hours. Cumulative occupational radiation doses were measured during the procedure.

**Results:** The median value of TSR and TBR aligns with the previously reported normal range. There were no significant differences in TSR and TBR between doses and between acquisition conditions. Four mCi group emitted more ambient (*P* < .05) and surface (*P* < .05) radiation than 2 mCi group. Veterinary staff's cumulative occupational radiations were significantly higher in 4 mCi group (*P* < .05).

**Conclusions and Clinical Importance:** The SFOV gamma camera demonstrated adequate image quality even with lower doses and shorter acquisition conditions. The radiation exposure from this procedure seems safe. Therefore, the SFOV gamma camera could be a valuable tool for evaluating the thyroid glands in cats.

## ABSTRACT F01: The effect of flunixin meglumine on viral shedding in calves

### **Trey N. Neyland,** **III** ^1^ ; Michael Apley^2^, DVM, PhD, DACVCP; Brian Lubbers^3^, DVM, PhD, ACVCP; Roman Pogranichniy^4^, DVM, MS, PhD; Leslie Weaver^5^, DVM, MS, DACVIM (LAIM)

#### ^1^College of Veterinary Medicine, Kansas State University, Manhattan, KS, USA; ^2^Professor, Production Medicine and Clinical Pharmacology Frick Professorship, College of Veterinary Medicine, Kansas State University, Manhattan, KS, USA; ^3^Associate Professor, College of Veterinary Medicine, Kansas State University, Manhattan, KS, USA; ^4^Associate Professor Virology, College of Veterinary Medicine, Kansas State University, Manhattan, KS, USA; ^5^Clinical Assistant Professor, Livestock Services, College of Veterinary Medicine, Kansas State University, Manhattan, KS, USA

**Background:** Non‐steroidal anti‐inflammatories (NSAIDs) are a common treatment for pyrexia in cattle with bovine respiratory disease (BRD). NSAIDs have known immunomodulatory effects in cattle with BRD. Minimal research has evaluated the effects that FDA approved NSAIDs have on viral shedding in clinical BRD cattle.

**Hypothesis/Objectives:** The hypothesis of the study was that flunixin meglumine would increase the magnitude and duration of viral shedding in calves inoculated with bovine herpesvirus‐1 (BHV‐1). The secondary objective was to investigate the shedding characteristics of BHV‐1.

**Animals:** Twelve Holstein cross‐bred steer calves, approximately 6 to 8 weeks of age, equally randomized into a treatment (FM) group or control (CON) group.

**Methods:** All calves were inoculated intranasally with approximately 4 mL of 1 × 10^5^ TCID50 of BHV‐1. Nasal swabs for BHV‐1 PCR testing were collected every 24 hours for a minimum of 7 samples post‐initial positive. Calves in the FM group were treated with 2.2 mg/kg of flunixin meglumine intravenously after the first BHV‐1 PCR positive sample. PCR sampling for a calf ceased after 2 consecutive negative PCR samples.

**Results:** Shedding of BHV‐1 ranged from 1 to 17 days for animals in both FM and CON groups. There was no statistical difference in magnitude or duration of BHV‐1 shedding between groups.

**Conclusions and Clinical Importance:** Within the study, intranasal inoculation with BHV‐1 resulted in a wide range of shedding magnitude and duration. Flunixin meglumine administration in BHV‐1 challenged calves did not result in an increase in magnitude or duration of viral shedding as analyzed.

## ABSTRACT F02: Abomasal displacement in pre‐weaned dairy calves—27 cases (2018‐2023)

### **Zuzanna Sikorska** ^1^; Diana Perez‐Solano^2^, DVM; Alexandra Burton^3^, BSc, BVSc, PhD, MRCVS, DACVIM (LAIM)

#### ^1^University of Wisconsin‐Madison, Madison, WI, USA; ^2^Large Animal Internal Medicine Resident, School of Veterinary Medicine, University of Wisconsin‐Madison, Madison, WI, USA; ^3^Clinical Assistant Professor, School of Veterinary Medicine, University of Wisconsin‐Madison, Madison, WI, USA

**Background:** Displacement of the abomasum (DA) is common in lactating adult dairy cattle. Little is known about the prevalence, etiology, treatment, and outcome of DA in young dairy calves. Current peer‐reviewed literature is limited to a few case reports. In our hospital, we encounter cases of DA in pre‐weaned calves.

**Objectives:** To describe the incidence, clinical presentation, diagnosis, ultrasonographic findings, treatment, and outcome of DA in pre‐weaned dairy calves in a referral hospital population.

**Animals:** 27 hospitalized calves.

**Methods:** Retrospective case series. Medical records from January 2018 to December 2023 were reviewed to identify pre‐weaned, dairy cattle under 4 months old, diagnosed with confirmed DA.

**Results:** 27 pre‐weaned calves with DA were identified, all with left displaced abomasum (LDA). Median age was 60.5 days (range, 29‐112 days). Physical examination findings consistent with LDA‐positive percussion and/or succussion were present in 19/27 (70%) of cases. In all calves LDA was confirmed via ultrasonography. Hypochloremic, metabolic alkalosis was detected in 42.3% (11/26). Bronchopneumonia was the most common comorbidity. Overall survival to discharge was 59%. Of 21/27 treated medically, 11 survived. Surgical management was pursued in 6/27 of which 5/6 survived.

**Conclusions and Clinical Importance:** Clinicians should be aware that LDA can occur in pre‐weaned milk‐fed calves. Concurrent systemic disease may predispose dairy calves to development of LDA. Medical management is sufficient in many cases, though surgical correction may be required. With early identification, prognosis is fair.

## ABSTRACT F03: 24‐hour electrocardiographic monitoring for assessment of cardiac arrhythmias in healthy and hospitalized goats

### **Alyssa Sullivan** ^1^; Darcy Adin^2^, DVM, DACVIM (Cardiology); Daniela Luethy^3^, DVM, DACVIM (LAIM)

#### ^1^College of Veterinary Medicine, University of Florida, Gainesville, FL, USA; ^2^Clinical Professor, Cardiology, SACS, College of Veterinary Medicine, University of Florida, Gainesville, FL, USA; ^3^Assistant Professor, Clinical Studies, New Bolton Center, School of Veterinary Medicine, University of Pennsylvania, Kennett Square, PA, USA

**Background:** Information regarding caprine cardiology is limited. The clinical utility of continuous 24‐hour electrocardiographic (ECG) recordings for detection of arrhythmias in hospitalized goats has not been reported.

**Hypothesis/Objectives:** (1) To determine the clinical feasibility of continuous 24‐hour ECG monitoring in goats; (2) to report the frequency of arrhythmias in healthy goats and hospitalized medically ill goats.

**Animals:** 11 healthy goats, 20 hospitalized medically ill goats.

**Methods:** Prospective clinical study. Echocardiograms and continuous 24‐hour ECG recordings were performed on all goats. Electrocardiograms were analyzed for rhythm diagnosis. Continuous data were assessed for normality using Shapiro‐Wilk. *χ*2 was used for VPD comparison between groups.

**Results:** ECG monitors were well‐tolerated in 30/31 goats, with no adverse effects. Twenty‐eight were of sufficient quality for analysis with median readable time of 23 hours (range, 0‐24 hours). All goats had structurally normal hearts. Eleven goats had ventricular arrhythmias (4 healthy, 7 medically ill), consisting of single ventricular premature depolarizations (VPDs) in 7 goats (3 healthy, 4 medically ill), VPDs and ventricular couplets in 2 goats (1 healthy, 1 medically ill), and non‐sustained idioventricular/ventricular ectopic runs in 2 goats (2 medically ill). Median number of VPDs was 0 (range, 0‐9) in healthy goats and 0 (range, 0‐201) in medically ill goats (χ2, *P* = .95).

**Conclusion:** The majority of goats tolerated 24‐hour ECG monitoring well, although a few recordings were poor quality. Ventricular arrhythmias were seen in healthy and medically ill goats but idioventricular/ventricular runs were only seen in medically ill goats.

## ABSTRACT F04: Clinical findings and outcome of goats diagnosed with discospondylitis and vertebral osteomyelitis on computed tomography

### **Alyssa Sullivan** ^1^; Elodie Huguet^1^, DVM, DACVR^1^ ; Ashley Vanderbroek^2^, DVM, DACVS (LA); Shannon Darby^1^, DVM, DACVIM (LAIM); Daniela Luethy^3^, DVM, MPH, DACVIM (LAIM)

#### ^1^College of Veterinary Medicine, University of Florida, Gainesville, FL, USA; ^2^College of Veterinary Medicine, Michigan State University, East Lansing, MI, USA; ^3^School of Veterinary Medicine, University of Pennsylvania, Kennett Square, PA, USA

**Background:** Vertebral infections, including vertebral osteomyelitis, septic physitis, and discospondylitis, are rarely reported in goats, and when reported, have been largely limited to postmortem case reports. The clinical outcome of these patients has not previously been reported.

**Objective:** To describe the clinical findings and outcome of goats diagnosed with vertebral infections via computed tomography.

**Animals:** Five goats diagnosed with vertebral osteomyelitis, septic physitis, and discospondylitis via computed tomography.

**Methods:** Retrospective case series.

**Results:** The most common presenting complaints were progressive weakness, paresis, and recumbency. Three goats were tetraparetic and two goats demonstrated pelvic limb paraparesis. Clinicopathologic findings included leukocytosis, mature neutrophilia, and hyperfibrinogenemia. The most common vertebrae affected were C7‐T1. All five goats had discospondylitis with or without vertebral osteomyelitis and septic physitis. CT evidence of spinal cord compression was appreciated in 4/5 goats. Medical management (antimicrobials, physical therapy, analgesia, supportive care) was attempted in 4 goats, while one goat was euthanized at the time of diagnosis. All four goats that were treated regained ambulatory ability and survived to hospital discharge.

**Conclusions and Clinical Importance:** Despite severity of CT imaging findings, goats with discospondylitis, septic physitis, and vertebral osteomyelitis can successfully return to ambulatory function. Further studies are required to determine ideal treatment regimens.

## ABSTRACT F05: Antimicrobial resistance of fecal *Escherichia coli* from calves fed milk containing antimicrobial residues

### **Véronique Bernier Gosselin** ^1^; Vincent Perreten^2^; Gertraud Schüpbach‐Regula^3^; Mireille Meylan^1^

#### ^1^Clinic for Ruminants, Vetsuisse Faculty, University of Bern, Bern, Switzerland; ^2^Division of Molecular Bacterial Epidemiology and Infectious Diseases, Institute of Veterinary Bacteriology, Vetsuisse Faculty, University of Bern, Bern, Switzerland; ^3^Veterinary Public Health Institute, Vetsuisse Faculty, University of Bern, Bern, Switzerland

**Background:** The feeding of waste milk containing antimicrobial residues (WMA) to calves is associated with increased prevalence of multidrug resistance (MDR) in their commensal flora on large farms. This association may not be generalizable to small farms, on which the calves' exposure to residues may differ from that on large farms.

**Objective:** To evaluate the association between the practice of feeding WMA to calves and MDR of *Escherichia coli* in calf feces on small Swiss dairy farms.

**Animals:** 365 calves without history of antimicrobial therapy since birth, from 58 farms, on 29 of which WMA was fed to calves (WMA farms).

**Methods:** Observational study. 211 pooled calf fecal samples (1‐3 calves/visit) were collected during up to 4 visits at 3‐month intervals. Three *E. coli* colonies per pool were tested for antimicrobial susceptibility by broth microdilution. Data on sampled calves, herd characteristics, management practices, and herd‐level antimicrobial use were obtained.

**Results:** Among samples from WMA farms, 46.7% of samples were from calves that had not received WMA. The proportion of MDR isolates did not statistically differ between WMA farms (26.9%) and non WMA farms (21.1%, *P* = .23). Among all isolates, MDR was associated with herd‐level antimicrobial use in the previous month (vs no use, OR 8.29, 95% CI 1.36‐50.43).

**Conclusions and Clinical Importance:** A low incidence of calves' exposure to WMA might have contributed to the lower proportion of MDR isolates from WMA farms than previously reported. On small farms, WMA feeding may not be determining for MDR prevalence.

## ABSTRACT F06: Evaluating the preliminary bias of a human point of care lactate meter in farm animals

### **Ryan Flynn** ^1,2^, BS, DVM Candidate; Kati Houser^2^, BS, MS, LVMT; Pierre‐Yves Mulon^2^, DVM, DES, DACVS

#### ^1^University of Tennessee, Knoxville, TN, USA; ^2^Large Animal Clinical Sciences, University of Tennessee, Knoxville, TN, USA

**Background:** Plasma lactate concentration can provide useful information in farm animal species. Increased levels of plasma lactate stem from several etiologies including poor perfusion in the body, dehydration, and hypovolemia. Practitioners benefit from devices that give quick and accurate plasma lactate concentrations.

**Objective:** Determine the relationship between lactate measurements from a human POC blood analyzer validated in the use of large animal species vs a human POC lactate meter in farm animal species.

**Methods:** Blood samples were collected from farm animal patients which were presented and needed a blood gas analysis during any point while in hospital. Following sample collection, each sample was run on the device already validated in several large animal species. Using the residual blood, each sample was run on the human POC device that has not been studied in farm animal species. Data was then compiled and analyzed using a commercially available software to statically compare the two devices' results.

**Results:** Currently there have been a total of 70 samples. After analysis, regression identified a relationship of *Y* = 1.178*X* + 0.2338. Bland‐Altman analysis demonstrated a bias of 0.4513 ± 1.712 mmol/L (95% limits of agreement: −2.904 to 3.806) when comparing the human POC device not studied in farm animal species compared to the other POC device.

**Conclusions:** Practitioners should be aware of the bias and variation the human POC device has when reporting plasma lactate concentrations for farm animal species, which are elevated, compared to that of the previously validated device.

## ABSTRACT F07: Pharmacokinetics of single and multidose oral gabapentin in goats

### **Jessie Ziegler** ^1^; Meera Heller^2^; Sherry Cox^3^; Joe Smith^4^

#### ^1^Midwestern University, Phoenix, AZ, USA; ^2^University of California‐Davis School of Veterinary Medicine, Davis, CA, USA; ^3^Biomedical and Diagnostic Sciences, College of Veterinary Medicine, University of Tennessee, Knoxville, TN, USA; ^4^Large Animal Clinical Sciences, College of Veterinary Medicine, University of Tennessee, Knoxville, TN, USA

**Background:** There is a shifting public perception of animal welfare that has increased demand for establishing pain management strategies in livestock. Gabapentin is often utilized in practice to mitigate neuropathic pain. However, there is little pharmacokinetic information to guide its use in goats.

**Hypothesis/Objectives:** To describe the pharmacokinetics of oral gabapentin in goats given as a single dose (SD) and multidose (MD) regimen. An additional goal was to document any adverse effects of oral gabapentin in goats after SD or MD administration.

**Animals:** Six, healthy, adult, non‐lactating female goats.

**Method:** In a non‐randomized, pharmacokinetic study, 6 goats were administered gabapentin orally at a dose of 15 mg/kg for a SD regimen. Following a 3‐week wash‐out period, they were again given gabapentin at 15 mg/kg for a MD regimen (q 12 hours for 6 doses). During the study, blood samples were collected and analyzed for gabapentin concentrations via reversed phase high performance liquid chromatography.

**Results:** After SD administration maximum plasma concentration, time to maximum concentration, and elimination half‐life were: 3.22 μg/mL; 4.49 hr; and 8.15 hr respectively. After MD administration maximum plasma concentration, time to maximum concentration, and elimination half‐life were: 4.56 μg/mL; 2.24 hr; and 45.8 hr respectively. No adverse effects were noted in the study goats.

**Conclusion and Clinical Importance:** The altered elimination half‐life is suggestive of accumulation after MD, and clinicians should consider this when determining dosing strategies for gabapentin in goats. Further research is needed to increase understanding of the use and benefit of gabapentin in goats.

## ABSTRACT F08: The intestinal microbiome of cows with hemorrhagic bowel syndrome compared to healthy cows

### **Christian Gerspach** ^1^; Angelika Schoster^2^, Prof. DrMedVet, DVSc, PhD, DACVIM/DECEIM; Lydia Günther^1^, DrMedVet

#### ^1^Vetsuisse Faculty, University of Zurich, Zurich, Switzerland; ^2^Professor, Equine Clinic, Ludwig‐Maximilians‐Universität München, Munich, Germany

**ABSTRACT**

Hemorrhagic bowel syndrome (HBS) is an acute disease of the small intestine in cattle, associated with segmental hemorrhage and clot formation, leading to intestinal obstruction. Pathogenesis and etiology are currently unknown. Recently, the gastrointestinal microbiome has received increasing attention in association with a variety of diseases. The objective of this study was to compare the microbiome of different gastrointestinal segments of HBS affected cows and healthy cows. Ingesta samples from 10 gastrointestinal segments were collected from cows euthanized due to HBS (n = 5) and healthy cows (n = 40) euthanized at the University of Zurich farm animal hospital for reasons other than gastrointestinal disease. Next generation sequencing (Illumina) of the V4 region of the 16S RNA gene was used to assess the microbial composition. Alpha‐ and beta‐diversity was compared between groups and segments using the Tukey HSD test, and the Steel Dwass test for comparison of the phylogeny. A significant difference in microbiome composition was present in the duodenum, cranial jejunum, and ileum between control and HBS cows on the class and order level, but not the phylum level. HBS affected cattle had a significantly lower alpha‐diversity compared to controls in the abomasum (p 0.5). Cows with HBS have measurable shifts in the microbiota compared to healthy controls, whether this is cause or effect of the disease remains to be determined.

## ABSTRACT F09: The fecal microbiota of calves naturally infected with *Cryptosporidium* spp.

### **Diego E. Gomez** ^1^; Dasiel Obregon^1^; Jennifer MacNicol^1^ ; David Renaud^1^; Lisa Gamsjaeger^2^

#### ^1^University of Guelph, Guelph, ON, Canada; ^2^North Carolina State University, Raleigh, NC, USA

**Background:** Little is known regarding fecal microbial alterations due to *Cryptosporidium* infection in calves.

**Hypothesis/Objective:** Describe and compare the fecal microbiota of *Cryptosporidium*‐infected and uninfected calves with or without diarrhea.

**Animals:** 28 calves.

**Methods:** Case‐control study. Calves with loose or watery feces were considered diarrheic. Feces were collected on the day of the onset of diarrhea. Fecal microbiota was characterized by sequencing of 16S ribosomal RNA gene amplicons. Mixed linear regression, differential abundance and co‐occurrence network analysis were conducted to determine the impact of *Cryptosporidium* spp. infection on the fecal microbiota.

**Results:** The fecal microbial richness was significantly higher in nC‐nD than in C‐nD and C‐D (*P* < .05).

**Conclusion and Clinical Relevance:** Infection with *Cryptosporidium* induces alterations in the composition and assembly of gastrointestinal microbiota regardless of the development of diarrhea.

## ABSTRACT F10: Evaluation of strategic chute‐side point‐of‐care ultrasonography viability for identification of interstitial pneumonia

### **Luis Felipe Barbosa Braga Feitoza** ^1^; Makenna Jensen^1^; Katie Long^1^; Laura Carpenter^1^; Kadyn Nuncio^1^; Brandon Plattner^2^, DVM, PhD, DACVP; Abigail Finley^3^, DVM, PhD, DACVP; Brad White, DVM, MS^1^

#### ^1^Kansas State University—Beef Cattle Institute, Manhattan, KS, USA; ^2^Department of Diagnostic Medicine and Pathobiology, Kansas State University, Manhattan, KS, USA; ^3^Texas A&M School of Veterinary Medicine and Biomedical Sciences, College Station, TX, USA

**Background:** Respiratory disease is the primary cause of mortality in the feedlot cattle industry. Many cases, particularly interstitial pneumonias, lack comprehensive descriptions and understanding. Differentiation between diseases could potentially dictate treatment strategies and management decisions.

**Hypothesis/Objectives:** Strategically utilize point‐of‐care ultrasound (POCUS) on the caudo‐dorsal region of the right lung to determine potential associations between specific imaging parameters at time of treatment and presence of interstitial pneumonia in cattle mortalities.

**Animals:** Commercial beef cross feedlot animals (n = 40, 343 ± 13.8 kg) that were evaluated at chute‐side at time of treatment and also deceased/necropsied from July 10th to July 28th.

**Methods:** Perform modified pulmonary POCUS evaluations on cattle treated for respiratory disease in a commercial feedyard and determine the association with histopathology findings and imaging parameters using a stepwise (backward) multivariate logistic regression on interstitial pneumonia outcomes on deceased cattle from the population that were also evaluated at chute‐side.

**Results:** Multivariate logistic regression identified statistically relevant variables for identification of interstitial pneumonia diagnosis outcome. Variables deemed relevant for this statistical model were B‐line count, ultrasound lung score, and A‐line count (Chi‐square, *P* = .05, *P* = .05, *P* = .0009, respectively). Sex and days on feed variables were used as a fixed‐effect (Chi‐square, *P* = .19, *P* = .03). Variance inflation factor (VIF) showed very low multicollinearity between variables in the model.

**Conclusions:** Variables identified by the model that were collected by using the modified approach to lung ultrasound at chute‐side at the time of treatment showed great potential for future applications of this technology to differentiate interstitial pneumonias from bronchopneumonia.

## ABSTRACT GI01: Autologous oral fecal microbiota transplantation and microbiome recovery after antibiotic treatment: A randomized controlled trial

### **Yi Kwan Lee** ^1^; Jan Suchodolski^2^; Thomas Lendvay^3^; William DePaolo^3^ ; Frederic Gaschen^1^

#### ^1^Department of Veterinary Clinical Sciences, School of Veterinary Medicine, Louisiana State University, Baton Rouge, LA, USA; ^2^Gastrointestinal Laboratory, College of Veterinary Medicine, Texas A&M University, College Station, TX, USA; ^3^Amend Pet (Tend‐Health, Inc.), Seattle, WA, USA

**Background:** Metronidazole is commonly prescribed to dogs with diarrhea and has a substantial negative impact on the gut microbiome and metabolome. The restoration of microbiome following discontinuation of metronidazole can be delayed.

**Objectives:** To describe the impact of 10‐day autologous oral FMT administration on the recovery of fecal microbiome after metronidazole treatment in healthy dogs.

**Animals:** Twenty healthy pet dogs.

**Methods:** Prospective, randomized controlled study. Fecal samples collected from each dog before initiating metronidazole treatment were processed into fecal microbiota transplantation (FMT) capsules using a capsule processing device and stored frozen until administration (1.25‐2.33 g feces/kg PO over 10 days). Dogs were randomly assigned to 2 groups. All dogs received a 7‐day course of metronidazole (12.5 mg/kg PO q 12 h). Subsequently, test dogs received oral FMT capsules for 10 days (D0‐D10), while control dogs only received FMT 24 days after completion of metronidazole treatment (D24‐D34). The fecal microbiome was evaluated using the qPCR‐based fecal dysbiosis index (FDI) at 6 time points. Results were evaluated using mixed‐effects analysis with Sidak's multiple comparisons test and Fisher exact test as appropriate.

**Results:** Metronidazole increased FDI and decreased the abundance of *Clostridium hiranonis* immediately after treatment (D0) in all dogs (*P* = .0001). A higher proportion of control dogs had an abnormal FDI (>0) at D10 (*P* < .05) and decreased C. hiranonis abundance at D10 (*P* < .05) and D17 (*P* < .02) compared to test dogs.

**Conclusions and Clinical Importance:** This study demonstrates the efficacy of autologous oral FMT capsules in accelerating the recovery of gut microbiome after metronidazole treatment.

## ABSTRACT GI02: Impact of omeprazole on esophageal microbiota in dogs using a minimally invasive sampling method

### **Aditi Handa** ^3^; Giovana Slanzon^2^, BS, MS, PhD; Yoko Ambrosini^3^, DVM, MPVM, PhD, DACVIM (SAIM); Jillian Haines^3^, DVM, MS, DACVIM (SAIM)

#### ^3^Veterinary Teaching Hospital, Washington State University, Pullman, WA, USA; ^2^Postdoctoral Researcher, Tropical Plants and Soil Sciences, University of Hawaii, Honolulu, HI, USA; ^3^Associate Professor, Veterinary Clinical Sciences, Washington State University, Pullman, WA, USA

**Background:** The impact of omeprazole on human esophageal microbiome (EM) and its associated side effects have been extensively studied. While the esophageal string test (EST) is a non‐invasive technique established for sampling human EM, its application in assessing the effect of omeprazole on canine EM has not been explored.

**Objectives:** To assess the changes and subsequent recovery of the canine EM following omeprazole treatment, utilizing the EST in awake dogs.

**Animals:** Ten healthy, client‐owned adult dogs.

**Methods:** A prospective longitudinal design was employed, where esophageal samples were initially collected using EST (day 0), involving the oral administration of an EST capsule and subsequent retrieval after 15 minutes for pH‐based segment identification. The dogs were then administered 1 mg/kg omeprazole orally, twice daily for 14 days. Follow‐up EST samplings were conducted on days 15 and 45. Samples were sequenced targeting the V3‐V4 region of the 16Ss rRNA gene. Diversity as well as linear discriminant analysis effect size (LEfSe) analysis were conducted by Zymo Research (Irvine, CA).

**Results:** All dogs tolerated the EST capsules without adverse effects. Diversity analysis revealed no significant alterations in alpha and beta diversity (Bray‐Curtis) across the time points. The predominant phyla identified were Proteobacteria and Bacteroides. Enterobacteriaceae was enriched in samples collected at day 15 (LDA score = 2.8, *P* value = .04).

**Conclusions and Clinical Importance:** Omeprazole therapy did not significantly alter the canine EM in this study. The application of EST in dogs highlights its use as non‐invasive tool for investigating the role of EM in canine esophageal health and disease.

## ABSTRACT GI03: Gastrointestinal microbiome relationship to plasma glucagon‐like peptide‐2 in dogs with idiopathic chronic enteropathies

### **Caylie D. Voudren** ^1^; Erin Mayhue^2^, BS; Michelle Riehm^2^, MS, DVM, DAVIM (SAIM); Maria Jugan^2^, DVM, MS, DACVIM (SAIM)

#### ^1^Veterinary Health Center, Kansas State University, Manhattan, KS, USA; ^2^Clinical Sciences, College of Veterinary Medicine, Kansas State University, Manhattan, KS, USA

**Background:** Gastrointestinal microbiota and metabolic by‐products stimulate glucagon‐like peptide‐2 (GLP‐2) secretion from enteroendocrine cells. GLP‐2 is responsible for intestinal mucosal barrier maintenance. Chronic enteropathy (CE) in dogs is associated with decreased plasma GLP‐2 concentrations and microbiome dysbiosis.

**Objectives:** To determine the association between gastrointestinal microbiota populations and plasma GLP‐2 concentrations in dogs with CE.

**Animals:** 18 client‐owned dogs with uncontrolled idiopathic CE and 16 client‐owned healthy control (HC) dogs.

**Methods:** Untargeted 16S V4 rRNA and targeted (dysbiosis index) microbiome analysis was performed on fecal samples collected prospectively from HC dogs and CE dogs prior to and 1 month after initiation of individualized CE treatment. Plasma GLP‐2 was measured concurrently (ELISA). Diversity indices (Shannon diversity, principal coordinate analysis) and bacterial abundances were compared between groups and time points. Taxa associated with differences in GLP‐2 concentrations among groups were identified using principal component analysis followed by least squares regression.

**Results:** While targeted analysis did not identify taxa associated with plasma GLP‐2 (*R* ^2^ = 0.157; F [7, 38] = 1.007; *P* = .442), untargeted genomic sequencing identified 6 families (*R* ^2^ = 0.319; F [5, 40] = 3.741; *P* = .007) and 19 genera (*R* ^2^ = .276; F [2, 43] = 8.190; *P* = .001) that contributed to group variance in GLP‐2 concentration.

**Conclusion and Clinical Importance:** Gastrointestinal microbiome dysbiosis may contribute to decreased plasma GLP‐2 concentrations in dogs with CE. Given the role of GLP‐2 in maintaining normal intestinal mucosal structure, further exploration into the interrelation of dysbiosis with CE pathophysiology and treatment is warranted.

## ABSTRACT GI04: Internists preferred methods of colonoscopy preparation and perceived success of protocols in dogs and cats

### **Sydney M. Oberholtzer** ^1^; Megan Posukonis^2^, DVM; Tracy Hill^1^, DVM, DACVIM, PhD, DECVIM

#### ^1^University of Minnesota, Minneapolis, MN, USA; ^2^Iowa State University, Ames, IA, USA

**Background:** Best practices for colonoscopy preparation have not been established for dogs and cats. Therefore, small animal internal medicine (SAIM) specialists select protocols based on individual preferences and experiences.

**Objective:** To assess preferred methods of colonoscopy preparation among SAIM specialists.

**Subjects:** 171 SAIM specialists, 143 complete and 28 partial responses.

**Methods:** A Qualtrics survey link was distributed to the American College of Veterinary Internal Medicine SAIM listserv. The survey included 39 questions regarding respondents' preferred colonoscopy preparation protocols and the perceived adequacy of preparation for colonoscopy in dogs and cats.

**Results:** There were 154 unique dog and 142 unique cat protocols reported. When asked how often the preferred protocol produced adequate preparation, 42% (65/154) of dog protocols and 39% (57/143) for cat protocols were reported to achieve adequate preparation over 75% adequate of the time (Figure 1). Among these protocols specifically, the preparation in dogs most frequently used: PEG as a laxative (34/65), a fasting period of 24‐36 hours (24/65), and administration of three enemas prior to colonoscopy (15/65). For cats, preparation protocols most commonly used: PEG laxatives (24/57), a fasting period of 12‐24 hours (28/57), and administration of three enemas (12/57).

**Conclusions:** A wide variety of colonoscopy preparation protocols were reported; most common protocols include PEG, a fasting time of 24‐36 (dogs) or 12‐24 hours (cats), and enemas. A minority of respondents report adequate preparation in at least 75% of their patients, suggesting optimal preparation protocols should be investigated.

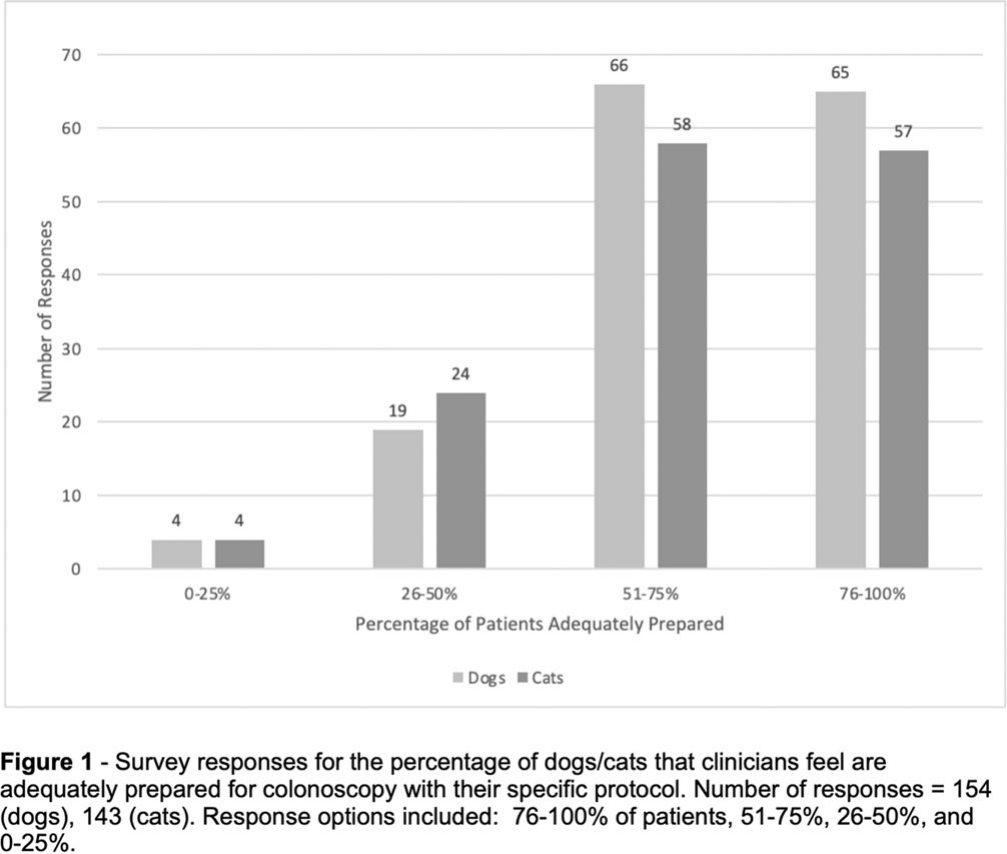

## ABSTRACT GI05: Management of acute diarrhea in dogs: A questionnaire of united states veterinarians

### **Alexis Hoelmer** ^1^; Stuart Walton^2^, BVSc, BScAgr, MANZCVS (SAIM), DACVIM; Domenico Santoro^3^, DVM, MS, DrSc, PhD, DACVD, DECVD, DACVM (Bacteriology/Mycology, Immunology); Justin Shmalberg^4^, DVM, DACVN, DACVSMR

#### ^1^University of Florida, Gainesville, FL, USA; ^2^Clinical Assistant Professor, SAM, Small Animal Clinical Sciences, University of Florida, Gainesville, FL, USA; ^3^Service Chief, Associate Professor, Dermatology, Small Animal Clinical Sciences, University of Florida, Gainesville, FL, USA; ^4^Clinical Professor, Integrative and Mobility Medicine, Department of Comparative, Diagnostic, and Population Medicine, University of Florida, Gainesville, FL, USA

**Background:** Diarrhea is a common clinical presentation, representing approximately 5% of all cases presenting to first opinion veterinary clinics. No clear consensus exists to guide clinicians in either diagnostic or therapeutic strategies, resulting in an array of recommendations regarding how acute diarrhea is managed.

**Hypothesis/Objectives:** To determine current management practices for acute diarrhea in dogs and determine differences in management strategies between disciplines, caseloads, and geographic location.

**Animals:** None.

**Methods:** Prospective anonymous online questionnaire of United States veterinarians. Respondents were asked about their diagnostic and treatment strategies for acute diarrhea (large and small bowel). Descriptive statistics and conditional logistic regression were used to analyze collected data.

**Results:** There were 386 responses. All geographic regions were represented. The highest percentage of respondents were from the northeast (133/386, 34.4%). Localization of diarrhea did not affect diagnostic or therapeutic recommendations for 39.9% (154/386) of respondents. The most frequently utilized diagnostic tools were fecal floatation and/or sedimentation (341/385, 88.3%), followed by complete blood count and/or chemistry (325/384, 84.6%). The most common therapeutic intervention was dietary management (317/374, 84.8%). Seventy percent (263/375, 70.1%) of respondents prescribed antibiotics. Metronidazole was the most common antibiotic prescribed (227/261, 86.9%). Sixty‐five percent (169/261, 64/8%) of respondents reported resolution of diarrhea within 7 days in >75% of cases.

**Conclusion and Clinical Importance:** Regardless of strategy, complete resolution of acute diarrhea was reported in the majority of cases, consistent with previous studies. Currently, antibiotics are commonly used in the treatment of acute diarrhea.

## ABSTRACT GI06: Serum cobalamin and folate concentrations, dysbiosis index, and histopathological findings in cats with chronic enteropathy

### **Katerina T. Moraiti** ^1^; Dimitra Karra^1^; Shelley Newman^2^; Jan Suchodolski^3^; Jörg Steiner^3^; Panagiotis Xenoulis^1,3^

#### ^1^University of Thessaly, Thessaly, Greece; ^2^Newman Specialty VetPath, Hicksville, NY, USA; ^3^Gastrointestinal Laboratory, Texas A&M University, College Station, TX, USA

**Background:** Changes in serum cobalamin and folate concentrations are common in cats with chronic enteropathy (CE).

**Hypothesis/Objectives:** Τo evaluate the association between serum cobalamin and folate with histopathological findings (HF), duration of clinical signs (CS) and dysbiosis index (DI) in cats with CE.

**Animals:** Sixty‐two cats with CE.

**Methods:** All cats underwent upper and lower endoscopy and biopsies were collected for histopathology. Serum cobalamin and folate, and fecal DI were measured in all cats.

**Results:** Twenty‐four of 62 (39%) cats had hypocobalaminemia. Serum cobalamin in cats with small cell lymphoma (SCL) (median: 272 ng/L) was significantly lower than in cats with chronic inflammatory enteropathy (CIE) (median: 913.4 ng/L; *P* = .0143). There was no significant difference in serum cobalamin between cats with mild or severe HF in the ileum (*P* = .96), or with an overall HF that was mild or severe (*P* = .09). However, serum cobalamin was significantly lower in cats with severe HF compared to those with mild HF in the duodenum (*P* = .01). There was a significant weak correlation between serum cobalamin and DI (r = −0.3; *P* = .04) and 29/51 (57%) cats had dysbiosis (DI > 0). Eleven of 62 (18%) cats had decreased (21.6 ng/L) serum folate. There was a significant weak correlation between serum folate and duration of CS (r = 0.3; *P* = .003).

**Conclusions and Clinical Importance:** Hypocobalaminemia was associated with more severe HF in the duodenum but not in ileum (where it is believed to be absorbed) and was negatively correlated with dysbiosis. Increased serum folate was associated with increased duration of CS.

## ABSTRACT GI07: Inflammatory cytokines suppress expression of tight junction and stemness‐related properties in canine intestinal organoids

### **Meg Nakazawa** ^1^; Yoko Ambrosini^1^; Itsuma Nagao^2^

#### ^1^Washington State University, Pullman, WA, USA; ^2^The University of Tokyo, Tokyo, Japan

**Background:** Chronic gastrointestinal disorders, such as inflammatory bowel disease (IBD), demand advanced research models to uncover their origins and potential treatments. To enhance our understanding and explore therapeutic options, it's crucial to create translational models that faithfully replicate key aspects of IBD. Three‐dimensional (3D) canine intestinal organoids, composed of diverse differentiated intestinal epithelial cells, present a promising avenue for such research.

**Objectives:** To investigate the effect of proinflammatory cytokines on canine intestinal epithelial using normal canine organoids.

Animals and **Methods:** Canine organoids were developed using colonic tissues biopsied from three healthy client‐owned dogs. Subsequently, we initiated cytokine treatment (30 ng/mL) and analyzed the immunofluorescent staining of tight junction protein ZO‐1 at 48 hours after exposure and leucine‐rich repeat‐containing G‐protein (Lgr5) gene expression at 24 and 48 hours after exposure, comparing it to non‐treated control organoids.

**Results:** We observed a significant decrease of fluorescent intensity of ZO‐1 after three cytokines (*P* < .001). And we also observe a significant decrease in Lgr5 mRNA quantitative PCR expression at 24 and 48 hours after treatment with IFN‐γ (*P* < .05 and *P* < .001). Conversely, there were no significant differences observed between the TNF‐α‐treated, IL‐1β‐treated and the control group.

**Conclusions and Clinical Importance:** This finding suggests that TNF‐α, IFN‐γ and IL‐1β inhibit the tight junction protein and IFN‐γ inhibits proliferative potential of canine intestinal organoids, indicating the potential therapeutic target of canine IBD. Our ability to observe these epithelial responses using canine organoids underscores the potential utility of these 3D organoids as an in vitro model for canine IBD.

## ABSTRACT GI08: The long‐term in vitro bacterial viability of lyophilized and frozen feline fecal microbial transplantation products

### **Nina Kristen E. Randolph** ^1^; Dubraska Diaz‐Campos, DVM, PhD^2^ ; Joany van Balen^3^, DVM, PhD; Nora Jean Nealon^4^, DVM, PhD; John Rowe^5^, DVM, MS; Lisa Wetzel^6^; Jenessa Winston^2^, DVM, PhD, DACVIM

#### ^1^The Ohio State University, Columbus, OH, USA; ^2^Assistant Professor, Veterinary Clinical Sciences, The Ohio State University, Columbus, OH, USA; ^3^Clinical Microbiology Laboratory Supervisor, Veterinary Clinical Sciences, The Ohio State University, Columbus, OH, USA; ^4^Postdoctoral Scholar, Veterinary Clinical Sciences, The Ohio State University, Columbus, OH, USA; ^5^PhD Student, Veterinary Internal Medicine Resident, Veterinary Clinical Sciences, The Ohio State University, Columbus, OH, USA; ^6^Veterinary Student, Veterinary Clinical Sciences, The Ohio State University, Columbus, OH, USA

**Background:** Fecal microbial transplantation (FMT) is the transfer of feces from a healthy donor into the gastrointestinal tract of a diseased recipient to confer a health benefit. The mechanism in which FMT confers a health benefit is linked to the viability and engraftment of microbes and the correction of dysbiosis.

**OBJECTIVES:** Our study aims to quantitate the colony forming units (CFUs) of microbes within feline FMT products using culture‐based techniques in aerobic and anaerobic environments.

**Animals:** Three screened healthy feline fecal donors each provided three separate fresh fecal samples for processing.

**Methods:** Fecal processing techniques include unprocessed and three double‐centrifuged fecal slurries with the following additives: 0.9% saline, 10% glycerol, and 25% maltodextrin and trehalose (M:D). FMT products were aliquoted for long‐term storage at −20°C, −80°C, and lyophilized for storage at room temperature. Timepoints for CFU/g quantitation include baseline, 1, 3, 6, and 12 months.

**Results:** Across all storage conditions, FMT products preserved with M:D exhibited the lowest mean log10 drop over 12 months. All FMT products, except 10% glycerol and 25% M:D frozen at −80°C, exhibited significant decreases in total CFUs over 12 months (*P* < .01).

**Conclusions and Clinical Importance:** M:D is the superior cryopreservative for feline FMT across all storage conditions. This study provides clinicians with evidence for producing and storing FMT in their own practice. Further research is needed to determine whether increased CFUs translates to microbe engraftment and thus additional clinical benefit.

## ABSTRACT GI09: Ileal and colonic renin‐angiotensin‐aldosterone system (RAAS) receptor dysregulation and fibrosis markers in canine chronic enteropathy

### **Romy M. Heilmann** ^1^; Franziska Dengler^2^, DVM; Denny Böttcher^3^, DVM; Joerg Steiner^4^, DVM, DACVIM (SAIM), DECVIM‐CA, PhD, AGA Fellow; Giacomo Rossi^5^, DVM, PhD, MS, DECZM

#### ^1^College of Veterinary Medicine, University of Leipzig, Leipzig, Germany; ^2^Professor of Physiology and Pathophysiology, Institute of Physiology, Pathophysiology and Biophysics, Vetmeduni Vienna, Wein, Austria; ^3^Institute of Veterinary Pathology, University of Leipzig, Leipzig, Germany; ^4^Professor of Small Animal Internal Medicine, Director of the Gastrointestinal Laboratory, College of Veterinary Medicine and Biomedical Sciences, Texas A&M University. College Station, TX, USA; ^5^University of Camerino, Camerino, Italy

**Background:** Upregulation of circulating RAAS components in dogs with chronic inflammatory enteropathy (CIE) has a signature similar to the phenomenon of “aldosterone breakthrough” and is associated with ileal, but not colonic, electrolyte transporter mRNA overexpression. Aldosterone promotes inflammation, fibrosis, and oxidant damage.

**Objectives:** Investigation of ileal and colonic RAAS receptors and fibrosis markers.

**Animals:** Archived tissue specimens from 7 CIE dogs and 6 apparently healthy controls.

**Methods:** Immunohistochemistry (IHC) evaluation for angiotensin II receptor type I (AGTR1) and type II (AGTR2), epithelial Na^+^‐channel (ENaC), Mas (MasR) and mineralocorticoid receptor (MCR), collagen‐1 and ‐3, and transforming growth factor‐β (TGFβ). IHC‐positive cells (quantified per 62 500 μm^2^ in 5 randomly selected fields of ileal and colonic compartments) were evaluated in CIE vs. healthy dogs and were tested for associations with clinical and histologic findings.

**Results:** Increased AGTR1^+^ cell counts were inversely associated with AGTR2^+^ and MasR^+^ cells and were positively correlated with profibrotic markers (collagen‐1, ‐3, and TGFβ). Local RAAS receptor positivity and fibrosis markers were linked to the severity of clinical signs and inflammatory and structural changes in the ileum and colon. However, MCR^+^ and ENaC^+^ cells differed spatially between both compartments and in relation to local RAAS receptors and fibrosis markers. These changes were more pronounced in dogs requiring immunomodulatory treatment (n = 3) than with food‐responsive disease (n = 4).

**Conclusions and Clinical Importance:** Local RAAS receptor dysregulation in canine CIE reflects a shift towards proinflammatory and profibrotic pathways. Discrepancies with reported mRNA expression levels (eg, for AGTR1) suggest a role of post‐translational variation.

## ABSTRACT GI10: 3D Morphogenesis in canine gut‐on‐a‐chip with healthy and IBD biopsy‐derived organoids

### **Yoko M. Ambrosini** ^1^; Itsuma Nagao^2^; Meg Nakazawa^1^

#### ^1^Washington State University, Pullman, WA, USA; ^2^The University of Tokyo, Tokyo, Japan

**Background:** Limited insights into intestinal bacteria's role in diseases like inflammatory bowel disease (IBD) have been due to a scarcity of effective canine in vitro models. Recent advances in gut‐on‐a‐chip technology offer new possibilities for studying the interaction between intestinal bacteria and epithelium.

**OBJECTIVES:** To create a microfluidic gut‐on‐a‐chip co‐culture system with organoids from both healthy and IBD‐afflicted dogs.

**Animals and Methods:** Colonoids from two healthy and two IBD‐affected dogs were cultured on the gut‐on‐a‐chip, forming villus‐like structures under dynamic conditions. We established co‐cultures with non‐pathogenic *Escherichia coli* (NPE) and compared them with static Transwell (TW) co‐cultures. We assessed epithelial barrier integrity by measuring transepithelial electrical resistance (TEER) and used immunofluorescence staining (IF) to evaluate ZO‐1, a tight junction protein, expression.

**Results:** The gut‐on‐a‐chip models, after 6‐9 days, showed 3D morphogenesis in the form of villus‐like structures in both healthy and IBD dog‐derived cells. IF confirmed ZO‐1 presence, indicating tight junction formation. NPE infection in the TW system significantly reduced TEER (48.2 ± 4.4% at 12 hours and 7.3 ± 5.3% at 24 hours), whereas the gut‐on‐a‐chip maintained TEER levels (100.3 ± 12.8% at 12 hours, 94.9 ± 2.2% at 24 hours, and 88.8 ± 15.0% at 48 hours), preserving 3D structure.

**Conclusions and Clinical Importance:** The canine gut‐on‐a‐chip model successfully replicates villus‐like structures and maintains a 3D epithelial configuration with both healthy and IBD organoids. This platform is a significant step forward for studying bacterial‐epithelial interactions in veterinary and human medicine, offering a dynamic system for co‐culturing live bacteria and preserving epithelial integrity.

## ABSTRACT GI11: Defining the core microbiome in healthy dogs

### **Connie A. Rojas** ^1^; Brian Park^1,2^, PhD; Guillaume Jospin^1,2^, MSc; Jessica Jarett^3^; Elisa Scarsella^4^, PhD; Zhandra Entrolezo^5^; Alex Martin^6^; Holly Ganz^7^

#### ^1^AnimalBiome; ^2^Bioinformatician, AnimalBiome; ^3^Director of Research, AnimalBiome; ^4^Director of Molecular Biology, AnimalBiome; ^5^Molecular Laboratory Manager, AnimalBiome; ^6^Chief of Staff, AnimalBiome; ^7^Chief Science Officer, Co‐Founder, AnimalBiome

**Background:** An understanding of the bacterial species comprising the core microbiome in a large cohort of healthy dogs is lacking but required for informing therapeutic efforts aimed at restoring dysbiotic microbiomes.

**Hypothesis/Objectives:** Identify the bacterial species that constitute the core microbiome in healthy dogs and examine the effects of sex‐neuter status, age, body weight, diet, probiotic intake, and geographic region on this core.

**Animals:** Fecal samples were collected from 286 healthy pet dogs with no current or past physical conditions, no clinical signs, and no reported medication use.

**Methods:** Full‐length PacBio16S rRNA gene sequence data from fecal samples were analyzed in R. Core bacterial species were present in at least 33% of dogs. Generalized additive models were used to correlate two core microbiome metrics with host factors.

**Results:** 27 bacterial species formed part of the core microbiome in healthy dogs, among them *Collinsella intestinalis*, *Megamonas funiformis*, *Peptacetobacter hiranonis*, *Prevotella copri*, *Streptococcus lutetiensis*, and *Turicibacter sanguinis*. Overall, sterilized females had more core taxa (x̄:16.4) than intact males (x̄:15) (GAM *P* < .05). Dogs of intermediate body weight had more core taxa than small or large dogs (GAM *P* < .05). Dogs fed kibble had more core taxa (x̄:17.6) than dogs fed raw (x̄:15.6) or cooked food (x̄:15.08) (GAM *P* < .05). The total sum of core taxa in the fecal microbiome decreased with age. No differences in core microbiome metrics were detected between healthy dogs that did vs. did not receive probiotics.

**Conclusions:** A common core group of bacteria was identified, some of which are known to decrease in abundance with gut dysbiosis.

## ABSTRACT GI12: Concentrations of fecal carbohydrates in dogs with chronic enteropathy

### **Chih‐Chun Chen** ^1^; Amanda Blake^2^; Paula Giaretta^2^; Rachel Pilla^2^; Jan Suchodolski^2^; Katherine Tolbert^2^

#### ^1^Gastrointestinal Laboratory, Department of Small Animal Clinical Sciences, Texas A&M University, College Station, TX, USA; ^2^Texas A&M University, College Station, TX, USA

**Background:** Carbohydrates are not required in the diet but provide nutritive value. A previous study showed increased fecal glucose and other selective carbohydrate concentrations in a subset of dogs with chronic enteropathy (CE) compared to healthy controls (HC) by untargeted mass spectrometry. Therefore, malabsorption was suspected to play a role in the pathogenesis of CE. However, fecal carbohydrate concentrations have not been confirmed by a targeted assay.

**Objective:** Analytically validate a gas chromatography‐tandem mass spectrometry (GCMS) assay for quantification of carbohydrates in feces and describe the concentrations of fecal carbohydrates in dogs with CE and HC.

**Animals:** Feces from 15 CE and 8 HC dogs.

**Methods:** Retrospective cross‐sectional study. Fecal carbohydrates, including glucose, fructose, mannose, galactose, xylose, ribose, arabinose, and rhamnose were measured by GCMS. Mann‐Whitney U tests were used to compare individual fecal carbohydrate concentrations between groups.

**Results:** Median (range) coefficients of variation of intra‐ and inter‐assay were 8.4% (1.2‐24.4%) and 11.4% (1.5‐25.7%). Fecal glucose concentration was significantly higher in CE (median [range]: 9725 [1994‐25 757] ng/mg) compared to HC (4362 [1694‐7299]) dogs (*P* = .03). No significant differences between groups were found in the concentrations of the other carbohydrates.

**Conclusions and Clinical Importance:** A targeted GCMS assay was analytically validated and confirmed previous untargeted metabolomics findings of increased fecal glucose concentration in dogs with CE. The cause and impact of increased fecal glucose in canine CE remains to be determined.

## ABSTRACT GI13: Microbial indole catabolites of tryptophan regulate mucosal barrier function and inflammatory responses in canine colonoids

### **Patrick C. Barko** ^1^; Christopher Zdyrski^2^; Abigail Ralston^2^; Ryan Feauto^2^; Karin Allenspach^3^; Jonathan Mochel^4^; David Williams^5^

#### ^1^University of Illinois, Champaign‐Urbana, Champaign, IL, USA; ^2^3D Health Solutions Inc., Athens, GA, USA; ^3^Department of Pathology, College of Veterinary Medicine, University of Georgia, Athens, GA, USA; ^4^Department of Pathology, College of Veterinary Medicine, University of Georgia, Athens, GA, USA; ^5^Department of Veterinary Clinical Medicine, College of Veterinary Medicine, University of Illinois at Urbana‐Champaign, Champaign, IL, USA

**Hypothesis/Objectives:** Human and rodent studies implicate microbial indole catabolites of tryptophan (MICT) in the regulation of intestinal mucosal barrier function and inflammatory responses. We hypothesized that two MICTs, indolepropionate (IPA) and indolealdehyde (IAld), would ameliorate epithelial barrier dysfunction and pro‐inflammatory responses induced by TNFα.

**Animals:** In vitro investigation using colonic epithelial organoids from a healthy dog (colonoids).

**Methods:** Colonoids were pre‐incubated with IPA (1 mM) or IAld (1 mM), then exposed to TNFα (0.3 nM). Mucosal barrier function was assessed using transepithelial electrical resistance (TEER) and mRNA sequencing was used to compare gene expression responses.

**Results:** TEER (mean ± SD) was significantly decreased following exposure to TNFα (1898.4 ± 340.9 Ω × cm^2^) compared to pre‐exposure baseline (3035.3 ± 265.5 Ω × cm^2^; *P* < .001). TEER was significantly higher in colonoids exposed to IPA + TNFα (2838.9 ± 213.6 Ω × cm^2^; *P* < .001) and IAld+TNFα (2979.2 ± 290.2 Ω × cm^2^; *P* = .001) compared to colonoids exposed to TNFα alone (1898.4 ± 340.9 Ω × cm^2^). Relative expression of genes regulating inflammatory responses and tight junction integrity were significantly altered by stimulation with TNFα. IPA and IAld ameliorated changes in expression of pro‐inflammatory genes induced by TNFα.

**Conclusions and Clinical Importance:** IPA and IAld regulate intestinal permeability and pro‐inflammatory gene expression, thereby ameliorating barrier dysfunction and inflammatory responses induced by TNFα in canine colonoids. These observations provide mechanistic insights to enteric microbiome‐host interactions, their influence on intestinal homeostasis, and novel therapeutic strategies in dogs with chronic enteropathies.

## ABSTRACT GI14: The long‐term in vitro bacterial viability of lyophilized and frozen canine fecal microbial transplantation products

### **Nina Kristen E. Randolph** ^1^; Dubraska Diaz‐Campos^2^, DVM, PhD; Joany van Balen^3^, DVM, PhD; Nora Jean Nealon^4^, DVM, PhD; John Rowe^5^, DVM, MS; Lisa Wetzel^6^; Jenessa Winston^2^, DVM, PhD, DACVIM

#### ^1^The Ohio State University, Columbus, OH, USA; ^2^Assistant Professor, Veterinary Clinical Sciences, The Ohio State University, Columbus, OH, USA; ^3^Clinical Microbiology Laboratory Supervisor, Veterinary Clinical Sciences, The Ohio State University, Columbus, OH, USA; ^4^Postdoctoral Scholar, Veterinary Clinical Sciences, The Ohio State University, Columbus, OH, USA; ^5^PhD Student, Veterinary Internal Medicine Resident, Veterinary Clinical Sciences, The Ohio State University, Columbus, OH, USA; ^6^Veterinary Student, Veterinary Clinical Sciences, The Ohio State University, Columbus, OH, USA

**Background:** Fecal microbial transplantation (FMT) is increasingly utilized in canine medicine. Convenient storage facilitates the use of FMT in practice; however, the long‐term effect of freezing and lyophilization on microbial viability is unknown.

**Objectives:** Our study aims to quantitate the colony forming units (CFUs) of microbes within canine FMT products using culture‐based techniques in aerobic and anaerobic environments.

**Animals:** Three screened healthy canine fecal donors each provided three separate fresh fecal samples for processing.

**Methods:** Fecal processing techniques include unprocessed and three double‐centrifuged fecal slurries with the following additives: 0.9% saline, 10% glycerol, and 25% maltodextrin and trehalose (M:D). FMT aliquots were stored at −20°C, −80°C, and lyophilized for storage at room temperature. Timepoints for CFU/g quantitation include baseline, 1, 3, 6, and 12 months.

**Results:** At the 12‐month timepoint, lyophilized products preserved with M:D yielded significantly greater total CFUs compared with other lyophilized FMT products (*P* < .01). For FMT products frozen at ‐20C, FMT preserved with glycerol and M:D yielded significantly more CFUs than other products (*P* < .01), with no significant difference between glycerol and M:D (*P* = .06). All FMT products, except 10% glycerol frozen at −80°C, exhibited a significant decrease in total CFUs over the 12‐month period (*P* < .01). For all products, storage at −80°C yielded significantly more total CFUs than storage at −20°C (*P* < .01).

**Conclusions and Clinical Importance:** This study provides clinicians with evidence for producing and storing canine FMT in their own practice. Further research is needed to determine whether increased CFUs translates to microbe engraftment and thus additional clinical benefit.

## ABSTRACT GI15: The in vitro bacterial viability of commercially available veterinary fecal microbial transplantation products

### **Nina Kristen E. Randolph** ^1^; Dubraska Diaz‐Campos^2^, DVM, PhD; Joany van Balen^3^; Nora Jean Nealon^4^, DVM, PhD; John Rowe^5^, DVM, MS; Lisa Wetzel^2^; Jenessa Winston^2^, DVM, PhD, DACVIM

#### ^1^The Ohio State University, Columbus, OH, USA; ^2^Veterinary Clinical Sciences, The Ohio State University, Columbus, OH, USA; ^3^Clinical Microbiology Laboratory Supervisor, Veterinary Clinical Sciences, The Ohio State University, Columbus, OH, USA; ^4^Postdoctoral Scholar, Veterinary Clinical Sciences, The Ohio State University, Columbus, OH, USA; ^5^PhD Student, Veterinary Internal Medicine Resident, Veterinary Clinical Sciences, The Ohio State University, Columbus, OH, USA

**Background:** Fecal microbial transplantation (FMT) is the transfer of feces from a healthy donor into the gastrointestinal tract of a diseased recipient to confer a health benefit. FMT is increasingly utilized in veterinary medicine and is offered commercially by AnimalBiome.

**Objectives:** This study aims (1) to quantitate the colony forming units (CFUs) in AnimalBiome FMT products compared to freshly processed FMT and (2) to evaluate the microbial composition across multiple lots of commercial FMT compared to in‐house prepared fresh and lyophilized FMT.

**Animals:** Three lots each of AnimalBiome DoggyBiome (DB), DoggyBiome from raw fed dogs (DBR), and KittyBiome (KB) were evaluated. Freshly processed stool from screened canine and feline fecal donors were used as controls.

**Methods:** FMT products were cultured in aerobic and anaerobic environments. 16 s rRNA amplicon sequencing (V4 region) was performed on FMT products and colonies taken FMT cultures.

**Results:** Freshly processed feces consistently yielded the highest CFUs, but did not significantly differ from in‐house lyophilized FMT (dogs, *P* = .49; cats, *P* = .28). KB and in‐house feline lyophilized products exhibited comparable viability (*P* = .72). In contrast, in‐house canine lyophilized FMT viability was significantly greater than DB and DBR (*P* > .01). Each donor has unique microbial profiles (*P* = .03).

**Conclusions and Clinical Importance:** This study provides clinicians with evidence for the viability of commercially available FMT products compared to freshly processed FMT. Further research is needed to determine the impact of FMT processing on engraftment and clinical outcome.

## ABSTRACT GI16: Twice daily modified cyclosporine for the treatment of chronic pancreatitis in cats

### **Yu‐An Wu** ^1^; Jonathan Lidbury^1^; Samiran Sinha^2^; Joerg Steiner^1^

#### ^1^Gastrointestinal Laboratory, Texas A&M University, College Station, TX, USA; ^2^Department of Statistics, Texas A&M University, College Station, TX, USA

**Background:** A past clinical trial in cats with chronic pancreatitis suggested that once‐daily treatment with cyclosporine is associated with a reduction of serum feline pancreatic lipase immunoreactivity (fPLI) concentration. Whether a higher dosing frequency of cyclosporine is beneficial is of interest.

**Hypothesis:** Twice daily cyclosporine is associated with reduced serum fPLI concentrations in cats with chronic pancreatitis.

**Animals:** Ten client‐owned cats with ≥2‐week history of clinical signs of chronic pancreatitis and two measurements of serum fPLI concentration ≥8.8 μg/L. Cats were excluded if they received immunomodulatory drugs within the previous 3 months.

**Methods:** Non‐controlled clinical trial. Cats received cyclosporine (Atopica 5 mg/kg PO q 12 h) for 21 days. Supportive treatment and treatment for comorbidities were allowed. Serum fPLI concentrations were measured at pre‐treatment baseline and on days 10 and 21 of treatment.

**Results:** Pre‐treatment serum fPLI concentrations were higher than those at day 10 (*P* = .042), but not significantly different from day 21 (*P* = .076). All cats showed a reduction of serum fPLI concentrations on day 10, but 4 cats showed a subsequent increase on day 21, with 3 of those 4 cats having a serum concentration greater than at pre‐treatment baseline (Figures 1 and 2).

**Conclusions and Clinical Importance:** Cats treated with twice daily cyclosporine in conjunction with other supportive care had a reduction in serum fPLI concentrations after 10 days but not 21 days of treatment for some cats. Determining the reasons why some cats have a rebound of serum fPLI concentration warrants further investigation.

**Figure:**
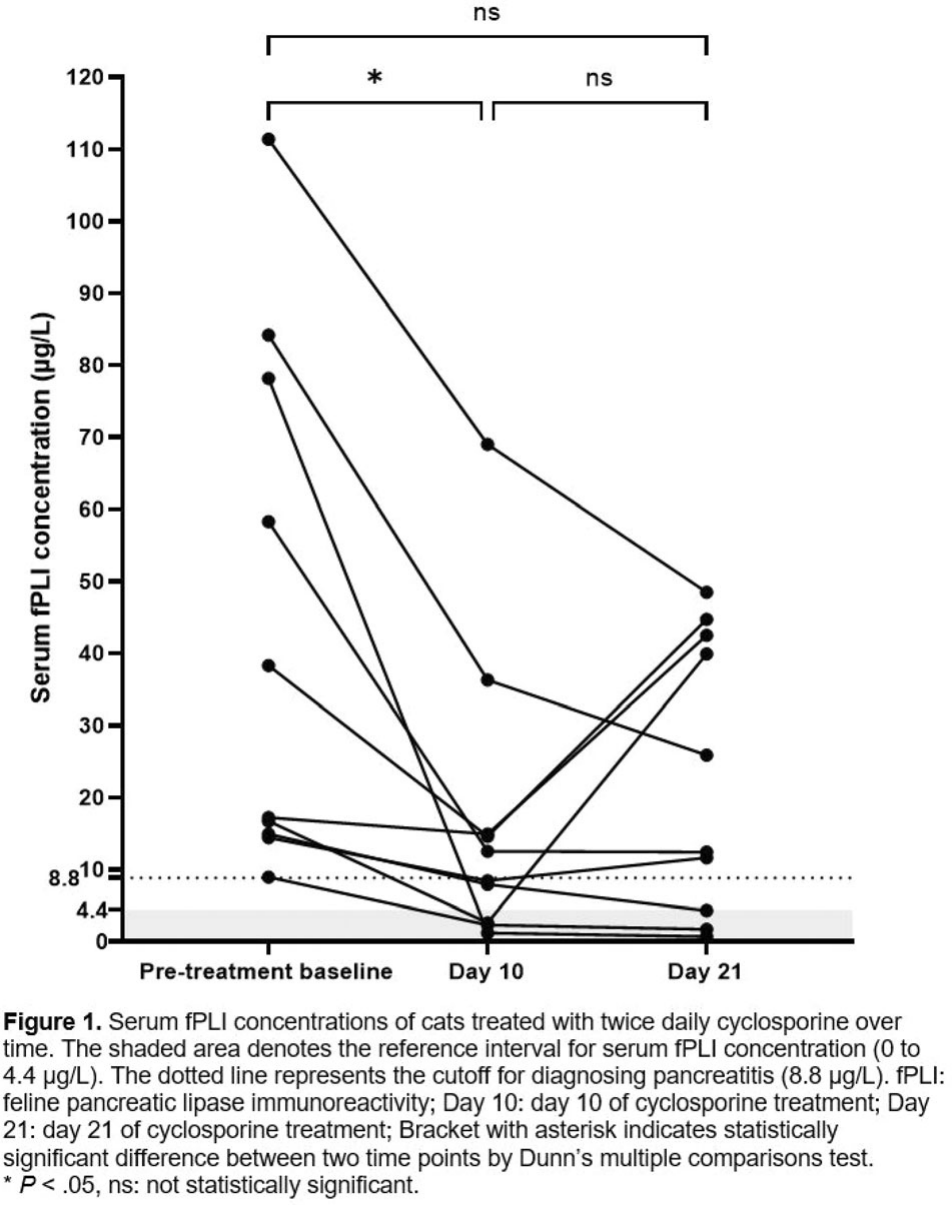
Figure 1.

**Figure:**
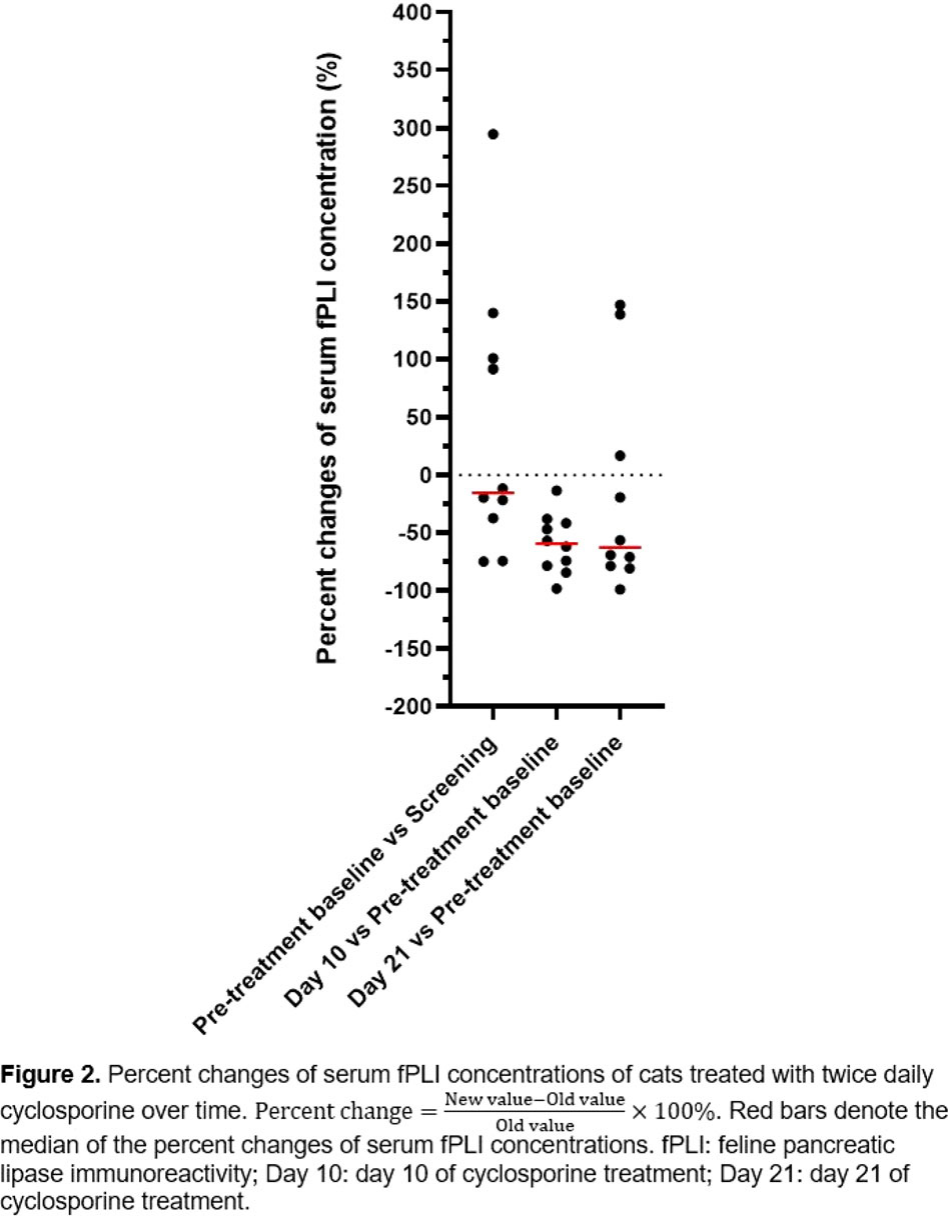
Figure 2.

## ABSTRACT GI17: Reasons for donor attrition in a companion animal stool bank

### **Holly H. Ganz**; Alex Martin; Becky Smith; Rebecca Oliver; Zhandra Entrolezo; Carly Pomeroy; Connie Rojas

#### AnimalBiome, Oakland, CA, USA

**Background:** Stool banks provide screened fecal material used in fecal microbiota transplantation (FMT). There is limited published data available on how to select donors and how often to test them for parasites and pathogens.

**Hypothesis/Objectives:** We performed cohort analysis on cat and dog donors in a stool bank in order to determine patterns in donor longevity, reasons for attrition, ideal testing frequency, and whether this differed over time.

**Animals:** We examined detailed records for 175 healthy cats and dogs who contributed to a stool bank in Northern California from 2018 through 2023.

**Methods:** Donor material was tested monthly for parasites, pathogens, and bacterial composition using Antech/Keyscreen, the Idexx diarrhea panel, and full length 16S rRNA gene sequencing. Reasons for donor attrition were recorded on a monthly basis.

**Results:** Average longevity of the 86 donors who started and left the program by December 2023 was 517 days. 89 donors remained active in the stool bank. The most common reasons for donor attrition were testing positive for *Giardia*, moving out of the area, poor owner compliance, antibiotic exposure, and bacterial dysbiosis. FeCoV, tapeworms, *Cryptosporidium*, and *Campylobacter* were also observed.

**Conclusions:** Stool banks require careful monitoring for parasites and pathogens in order to minimize the probability of transmission to compromised individuals.

## ABSTRACT GI18: Electrolyte imbalances in cats with chronic inflammatory enteropathy

### **Aarti Kathrani** ^1^; Romy Heilmann^2^; Ramona Knoll^2^; Berenice Schneider^2^; Iona Baker^1^

#### ^1^Royal Veterinary College, London, UK; ^2^University of Leipzig; Leipzig, Germany

**Background:** There is limited information on electrolyte imbalances in feline chronic inflammatory enteropathy (CIE).

**Animals:** 328 client‐owned cats from the Royal Veterinary College and University of Leipzig; CIE (139), alimentary small cell lymphoma (29), acute gastrointestinal disease (48) and healthy controls (119).

**Methods:** Retrospective study comparing serum electrolyte concentrations between the 4 groups of cats and with WSAVA gastrointestinal total and fibrosis histopathology scores in cats with CIE. Statistical analyses were performed using the Kruskal‐Wallis test and pairwise comparisons. Significance was defined as *P* < .05).

**Results:** Cats with CIE had higher absolute and relative sodium concentrations than cats with acute gastrointestinal disease (*P* < .001). Cats with CIE had lower absolute and relative sodium, higher absolute and relative potassium and lower sodium: potassium ratio compared to healthy cats (*P* < .001, *P* < .008 and *P* < .002, respectively).

For the CIE group, cats with a duodenal mucosal fibrosis score of 2 had lower relative sodium concentrations compared to cats with a score of 0 (*P* < 0.017), and cats with a score of 2 had lower relative total calcium compared to cats with a score of 1 and 0 (*P* < 0.015). Cats with a colonic mucosal fibrosis score of 1 had higher relative potassium and lower sodium: potassium compared to cats with a score of 0 (*P* < 0.011 and P < 0.005).

**Conclusions and Clinical Importance:** Cats with CIE, particularly those with higher fibrosis scores, have significant electrolyte abnormalities. Further research should aim to determine the pathogenesis for these findings and if intestinal fibrosis could be a therapeutic target in cats with CIE.

## ABSTRACT GI19: Serum concentrations of 7Α‐hydroxy‐4‐cholesten‐3‐one (C4) in dogs with chronic enteropathy

### **Chi‐Hsuan Sung** ^1^; Linda Toresson^2^; Amanda Blake^3^; Paula Giaretta^3^; Rachel Pilla^3^; Jonathan Lidbury^3^; Joerg Steiner^3^; Jan Suchodolski^3^

#### ^1^Texas A&M University, College Station, TX, USA; ^2^Evidensia Specialist Animal Hospital, Helsingborg, Sweden; ^3^The Gastrointestinal Laboratory, Texas A&M University, College Station, TX, USA

**Background:** Abnormal bile acid (BA) metabolism has been observed in dogs with chronic enteropathy (CE). In humans, BA malabsorption (BAM) results in excessive BA loss into the colon, triggering secretory diarrhea. This leads to compensatory BA synthesis from cholesterol. Serum 7α‐hydroxy‐4‐cholesten‐3‐one (C4), a key marker for the rate of BA synthesis, serves as a marker for BAM in humans. However, studies in dogs are limited.

**Objectives:** To develop and analytically validate a liquid chromatography with tandem mass spectrometry (LC‐MS/MS) method to quantify C4 in dog serum.

**Animals:** 17 privately‐owned healthy control dogs (HC) and 15 dogs with CE with partial or no response to food and immunosuppressant trials.

**Methods:** A cross‐sectional retrospective study. The LC‐MS/MS was validated according to FDA guidelines for bioanalytical method validation. Serum C4 quantification was performed using 50 μL of serum samples through LC‐MS/MS. Serum C4 concentrations were compared between groups by Mann‐Whitney *U*‐test.

**Results:** The assay was analytically validated with good linearity (1‐1000 ng/mL, R = 0.999) and adequate coefficients of variation of inter‐assay (median: 1.4%, range: 4.2‐7.9) and intra‐assay (median: 12.1%, range: 7.4‐14.8). Dogs with CE had significantly (*P* = 0.009) higher serum C4 concentrations (median: 53, range: 20‐204 ng/mL) than HC (median: 26, range: 16‐44).

**Conclusions and Clinical Importance:** Increased serum C4 concentrations were found in a subset of dogs with CE that poorly responded to standard treatments, suggesting a compensatory hepatic BA synthesis. This aligns with previous findings of decreased BA transporters in the ileum of dogs with CE and increased fecal BA concentrations.

vim24_636

## ABSTRACT GI20: Initial studies on fecal microbiota transplantation in dogs: an adjunct therapy in canine parvoviral diarrhea

### **Jyoti C. Kalita** ^1^; Amit Prasad^2^, BVSc, MVSc

#### ^1^G.B. Pant University of Agriculture and Technology, Pantnagar, Uttarakhand, India; ^2^Assistant Professor, Department of Veterinary Medicine, G.B. Pant University of Agriculture and Technology, Pantnagar, Uttarakhand, India

**Background:** Fecal microbiota transplantation (FMT) is a novel therapy in the field of gastroenterology and is studied at current times with greater curiosity.

**Objective:** To evaluate the efficacy of FMT as GI microbiome restorative therapy in conjunction with symptomatic therapy in parvoviral cases.

**Animals:** Of 258 confirmed cases of CPV, 12 cases were randomly selected and divided into 2 groups (B and C). Group B was treated with symptomatic therapy, whereas Group C was treated with symptomatic therapy along with FMT as an adjunct therapy.

**Methods:** A randomized case‐control study was carried out where animals of Group C (n = 6) were administered with FMT transplant from day 0 of presentation till day 7. Response to FMT was evaluated on the basis of time taken for resolution of diarrhea, time of hospitalization, clinical scoring, and recurrence of gastroenteritis within 2 months post‐therapy.

**Results:** 66.67% (4/6) dogs of FMT treated group had a quicker resolution of diarrhea, FMT treated dogs had better clinical scores compared to symptomatic therapy group (1 or clinically insignificant vs 4 or mildly diseased), lesser recurrence of diarrhea 2 months post therapy in FMT treated group (16.67% vs 50%). Post FMT one case showed low grade fever (n = 1) and epistaxis (n = 1) which resolved within 24 hours. Mean retention time improved (20.83 ± 2.39) minutes to (140 ± 12.66) on day 7 of the trial (*P* < 0.01).

**Conclusion:** FMT as an adjunct therapy might aid in early resolution of diarrhea in acute diarrhea in dogs.

**Figure:**
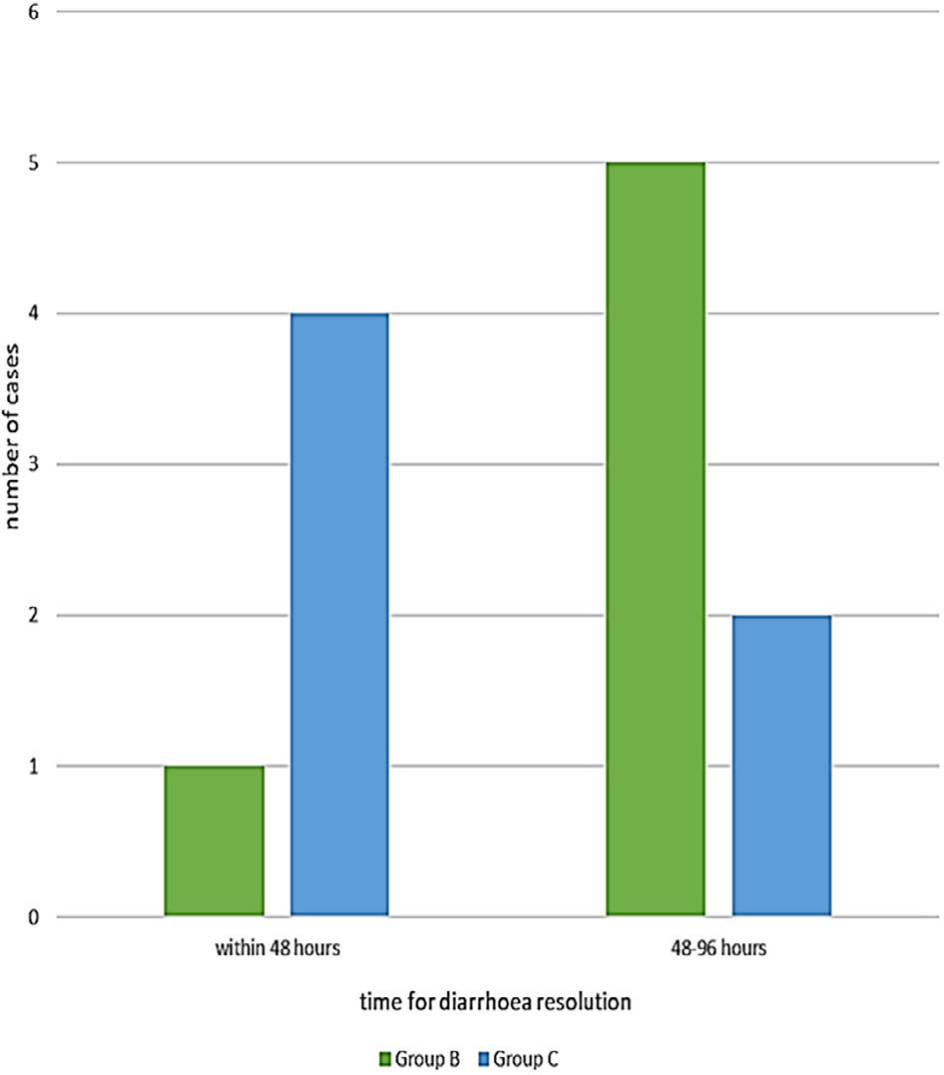
**Figure 1.** Time for diarrhea resolution.

**Table:** **Table 4.11.** Table depicting median (IQR) scores, *p* values and clinical significance of groups B and C from day 0 to day 7

Groups	Median score	IQR	*P* value	Clinical significance of the disease
**Group B**				
Day 0	15.5	1	0.82^a^	100% (severe)
Day 7	1	1	0.02^b^	Clinically insignificant
**Group C**				
Day 0	16	1	0.82^a^	100% (severe)
Day 7	4	1	0.02^b^	Mild

*Statistically significant at *P* < .05.

## ABSTRACT GI21: Effects of prokinetic drugs in dogs with clonidine‐delayed gastric emptying

### **Nicole Akers** ^1^; Ashlyn Harmon^1^; Tammy Dugas^2^, PhD; Levent Dirikolu^2^, DVM, PhD; Stephanie Dennis^3^, DVM; Frederic Gaschen^4^, Dr.med.vet., Dr.habil., DACVIM (SAIM), DECVIM‐CA (IM), AGAF

#### ^1^Louisiana State University, Baton Rouge, LA, USA; ^2^Associate Dean and Professor, Comparative Biomedical Sciences, Louisiana State University, Baton Rouge, LA, USA; ^3^Assistant Professor, Veterinary Clinical Sciences, Louisiana State University, Baton Rouge, LA, USA; ^4^Professor, Veterinary Clinical Sciences, Louisiana State University, Baton Rouge, LA, USA

**Background:** In dogs, delayed gastric emptying (GE) can occur following abdominal surgery or inflammation. While orally administered acetaminophen (AAP) can be used to assess GE, there are currently no clinically applicable methods to document the occurrence of gastroparesis in dogs.

**Objectives:** To evaluate the effects of gastric prokinetics on GE of liquids in dogs treated with clonidine, an alpha‐adrenergic agonist that delays GE, using AAP as a plasma tracer.

**Animals:** 8 healthy purpose‐bred dogs.

**Methods:** Prospective cross‐over study. In a randomized sequence over 6 weeks, each dog received either no treatment, clonidine (0.03 mg/kg SC), azithromycin (2 mg/kg IV) and clonidine, or metoclopramide (0.5 mg/kg SC) and clonidine 1 h before ingesting a liquid meal supplying 25% of the dog's RER mixed with acetaminophen (20 mg/kg). Blood samples were collected preprandially and at 10 time points between 0.5 and 24 hours. Plasma AAP concentrations were obtained using reverse‐phase HPLC. Times to maximum AAP concentration (Tmax) were calculated to estimate GE time, and a 2‐way ANOVA with Sidak‐adjusted multiple comparisons was performed.

**Results:** Clonidine treatment significantly delayed Tmax (*P* < .05). Additional therapy with prokinetics metoclopramide or azithromycin did not shorten Tmax.

**Conclusions and Clinical Importance:** AAP can be used to evaluate GE of liquids. Clonidine increased Tmax for AAP, presumably due to its delaying effect on GE. The clonidine model may not accurately replicate the mechanisms underpinning delayed GE in dogs. However, the documented lack of impact of gastric prokinetics in this study casts some doubt on their effect in clinical cases.

## ABSTRACT GI22: Efficacy of a flavored oral suspension of metronidazole against *Giardia duodenalis* in naturally infected dogs

### **Laure Poincelot**; Philippe Briantais; Charlotte Jamin; Sloane Jones; Delphine Rigaut

#### VIRBAC, Provence‐Alpes‐Cote d'Azur, France

**Background:** *Giardia duodenalis* is a commonly found gastrointestinal protozoan parasite in dogs. Metronidazole is a nitroimidazole antimicrobial with antiprotozoal actions.

**Objectives:** This clinical field study assessed the efficacy of a flavored oral suspension containing 125 mg/mL of metronidazole intended to treat *Giardia duodenalis* infection in dogs.

**Animals:** Client‐owned dogs (n = 129), naturally infected with *Giardia duodenalis* from European countries (Germany, Hungary, Portugal), were enrolled in the study, randomized to one of two groups and included in the effectiveness analysis. In the metronidazole treated‐group, eighty‐nine (89) dogs received a flavored oral suspension containing 125 mg/mL of metronidazole. In the control‐group, forty (40) dogs received a vehicle flavored oral suspension (equivalent to the final formulation of the product minus metronidazole). All dogs received 0.2 mL/kg twice daily for 5 consecutive days (ie, 25 mg/kg of metronidazole twice daily).

**Methods:** The study was a double‐masked vehicle‐controlled randomized and blocked multicenter trial. Fecal samples were collected pre‐treatment on Study Day −3, −2 and − 1 and post‐treatment on Study Day +5, +6 and + 7. A *Giardia* cysts count was calculated as *Giardia* cysts per gram of feces using immuno‐fluorescence antibody test.

**Results:** Metronidazole was significantly more efficacious than the vehicle reducing the baseline cysts counts 220 times more effectively (*P* < .001). Percentage baseline cyst count reduction, calculated for the metronidazole‐treated group, showed a metronidazole effectiveness of 99.92% (Figure 1).

**Conclusion:** The results demonstrated that the flavored oral suspension containing 125 mg/mL of metronidazole dosed at 0.2 mL/kg twice daily for 5 days is efficacious for treating *Giardia duodenalis* in naturally infected dogs.

**Figure:**
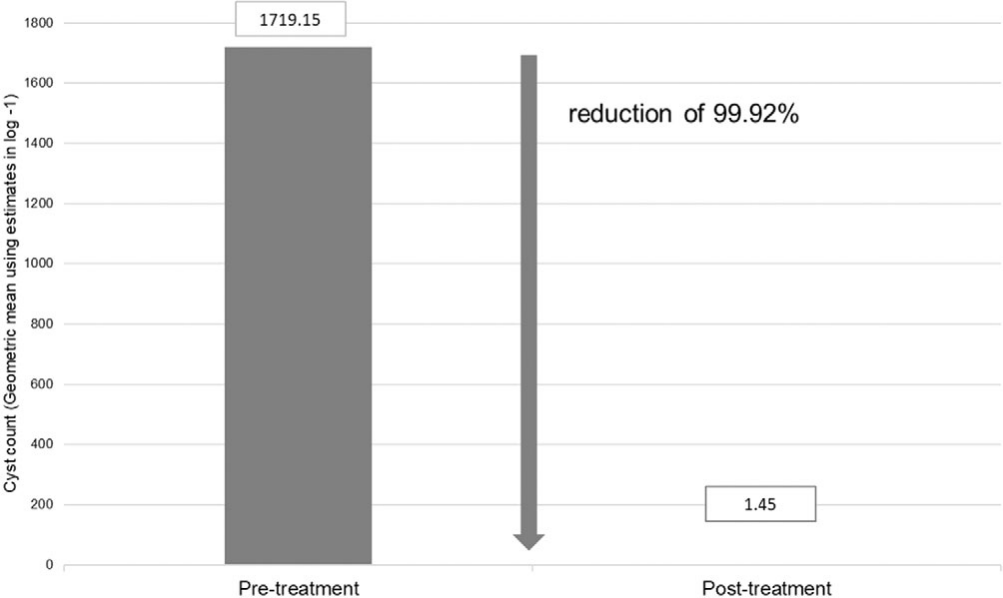
**Figure 1**. Cyst count in treated dogs (n = 89).

## ABSTRACT GI23: Safety evaluation of a flavored oral suspension of metronidazole in dogs

### **Laure Poincelot**; Philippe Briantais; Charlotte Jamin; Sloane Jones; Delphine Rigaut

#### VIRBAC, Provence‐Alpes‐Cote d'Azur, France

**Background:** *Giardia duodenalis* is a commonly found gastrointestinal protozoan parasite in dogs. Metronidazole is a nitroimidazole antimicrobial with antiprotozoal actions.

**Objectives:** This clinical field study assessed the safety of a flavored oral suspension containing 125 mg/mL of metronidazole intended to treat *Giardia duodenalis* infection in dogs.

**Animals:** Client‐owned dogs (n = 180), naturally infected with *Giardia duodenalis* from European countries (Germany, Hungary, Portugal), were enrolled in the study, randomized to one of two groups and included in the safety analysis. In the metronidazole treated‐group, 120 dogs received a flavored oral suspension containing 125 mg/mL of metronidazole. In the control‐group, 60 dogs received a vehicle flavored oral suspension (equivalent to the final formulation of the product minus metronidazole). All dogs received 0.2 mL/kg twice daily for 5 consecutive days (ie, 25 mg/kg of metronidazole twice daily).

**Methods:** The study was a double‐masked vehicle‐controlled randomized and blocked multi‐center trial. A veterinary exam including hematology, biochemistry and urine analyses was performed on Day −3 (pre‐administration) and Day +5 (post‐administration). Owners completed daily health observations from Days 0 through 7. Safety was assessed descriptively by comparing both groups for occurrence of adverse events (AEs).

**Results:** AEs (based on the VeDDRA dictionary) were tabulated in a frequency table for AEs considered related to the products (Table 1). Product‐related AEs were observed in the same frequency (10%) in both groups. Diarrhea and emesis were the most frequently reported signs in both groups. Neutropenia or elevated liver enzymes, signs expected after metronidazole administration, were observed in 0.8% of dogs in the metronidazole treated‐group. Neurological signs were not observed.

**Conclusion:** The results demonstrated that the flavored oral suspension containing 125 mg/mL of metronidazole could be administered with a measured risk of adverse reactions in dogs. The oral suspension offers an accurate weight‐based dose, favoring a safer use of metronidazole.

**Table:** **Table 1.** Related adverse reaction by SOC/PT according to VeDDRA coding in each group

	Treated‐group (n = 120)	Control‐group (n = 60)
	Number of affected dogs (%)	Number of affected dogs (%)
**Related AEs**	**12 (10%)**	**6 (10%)**
**Digestive tract disorders**	**11 (9.2%)**	**4 (6.7%)**
Diarrhea	8 (6.7%)	2 (3.3%)
Emesis	4 (3.3%)	2 (3.3%)
Gastroenteritis	1 (0.8%)	0
Haemorrhagic diarrhea	1 (0.8%)	0
**Investigations**	**3 (2.5%)**	**2 (3.3%)**
Elevated alanine aminotransferase (ALT)	1 (0.8%)	0
Neutropenia	1 (0.8%)	0
Hypoproteinaemia	1 (0.8%)	0
Increased blood urea or creatinine	1 (0.8%)	0
Other abnormal test results	1 (0.8%)	0
Lymphocytosis	0	2 (3.3%)
Neutrophilia	0	1 (1.7%)
Monocytosis	0	1 (1.7%)
Urine abnormalities	0	1 (1.7%)
Haemoglobinuria	0	1 (1.7%)
**Systemic disorders**	**1 (0.8%)**	**2 (3.3%)**
Anorexia	1 (0.8%)	2 (3.3%)
Lethargy	1 (0.8%)	1 (1.7%)
**Behavioral disorders**	**1 (0.8%)**	**0**
Hyperactivity	1 (0.8%)	0

**Figure:**
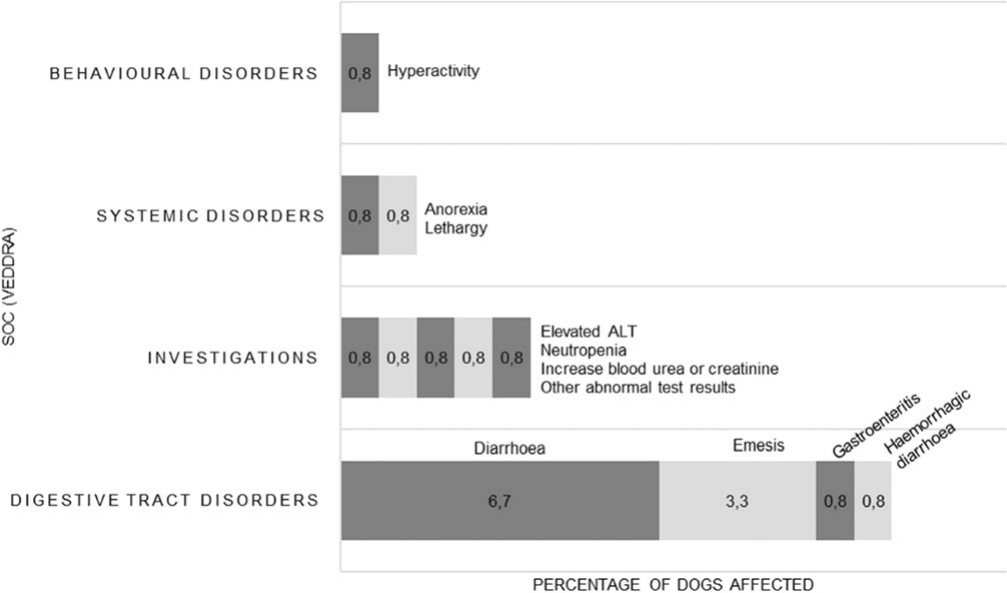
**Figure 1**. Related adverse reaction in metronidazole treated‐group (n = 120).

## ABSTRACT GI24: Prospective evaluation of the prevalence of thromboembolism in dogs with inflammatory protein‐losing enteropathy

### **Nene Oishi** ^1^; Hiroshi Ohta^2^; Masahiro Tamura^3^

#### ^1^Rakuno Gakuen University, Hokkaido, Japan; ^2^Associate Professor, Companion Animal Internal Medicine, Rakuno Gakuen University, Hokkaido, Japan; ^3^Lecturer, Companion Animal Internal Medicine, Rakuno Gakuen University, Hokkaido, Japan

**Background:** Inflammatory protein‐losing enteropathy (iPLE) may predispose dogs to thromboembolism. Little information is available regarding the prevalence of thromboembolism in dogs with iPLE.

**Objectives:** To determine the prevalence of thromboembolism in dogs with iPLE and to compare clinical and clinicopathologic variables between iPLE dogs with and without thromboembolism.

**Animals:** Twenty‐one client‐owned dogs with iPLE.

**Methods:** Dogs definitively diagnosed with iPLE based on standard diagnostic criteria and histopathology were prospectively recruited between January 2019 and December 2023. At the time of gastrointestinal endoscopic examination, thoracic and abdominal computed tomography angiography was performed in dogs with iPLE for the investigation of thromboembolism. Written informed consent was obtained from all owners. Clinical (eg, clinical severity, use of corticosteroids) and clinicopathologic (eg, albumin, coagulation parameters) variables were compared between iPLE dogs with and without thromboembolism (Fisher's exact test for categorical variables, and the Student's t‐test or the Mann‐Whiney U test for continuous variables). Statistical significance was set at P [sic].

**Results:** Thromboembolism was found in 3/21 dogs (14.3%) with iPLE. In these three iPLE dogs, thrombi were found in pulmonary artery, portal vein, and external iliac artery, respectively. Antithrombin activity was significantly lower in iPLE dogs with thromboembolism compared to those without thromboembolism (mean ± SD, 59.3 ± 31.0% vs. 103.7 ± 27.6%; *P* = .02).

**Conclusion and Clinical Importance:** This study prospectively revealed the prevalence of thromboembolism in dogs with iPLE. Decreased antithrombin activity might be associated with thromboembolism in dogs with iPLE.

## ABSTRACT GI25: Investigation of different leukocyte ratios as diagnostic markers in cats with chronic enteropathy

### **Albert E. Jergens** ^1^, DVM, PhD, MS, DACVIM (SAIM), AGAF; Alexandros Konstantinidis^2^, DVM, MSc, PhD; Ashley Griggs^3^; Katerina Adamama‐Moraitou^4^; Margaret Musser^5^; Nectarios Soubasis^4^; Dimitra Pardali^6^; Thodoris Christoforidis^4^; Mathios Mylonakis^4^

#### ^1^Professor, Iowa State University, Ames, IA, USA; ^2^School of Veterinary Medicine, Faculty of Health Sciences, Aristotle University of Thessaloniki, Thessaloniki, Greece; ^3^College of Liberal Arts and Science, Iowa State University, Ames, IA, USA; ^4^Companion Animal Clinic, School of Veterinary Medicine, Faculty of Health Sciences, Aristotle University of Thessaloniki, Thessaloniki, Greece; ^5^Department of Veterinary Clinical Sciences, College of Veterinary Medicine, Iowa State University, Ames, IA, USA; ^6^Diagnostic Laboratory, School of Veterinary Medicine, Faculty of Health Sciences, Aristotle University of Thessaloniki, Thessaloniki, Greece

**Background:** Definitive diagnosis of feline chronic enteropathies (CE), i.e., food‐responsive enteropathy (FRE), steroid‐responsive enteropathy (SRE), and intestinal small cell lymphoma (SCL) is difficult. Leukocyte ratios have been investigated as easily attainable and cost‐effective markers of chronic gastrointestinal inflammation in humans and dogs. Their role in the diagnosis of feline CE has not been reported.

**Aims:** To compare the blood neutrophil‐to‐lymphocyte (NLR), neutrophil‐to‐monocyte (NMR), and lymphocyte‐to‐monocyte (LMR) ratios in healthy cats (HC) and cats with FRE, SRE, and SCL, and to investigate the performance of these ratios as discriminative biomarkers.

**Animals:** Multi‐institutional retrospective analysis of healthy cats (n = 73) and cats diagnosed with FRE (n = 59), SRE (n = 56), and SCL (n = 33) from 2010 to 2022.

**Methods:** Neutrophil, lymphocyte, and monocyte counts were extracted from the routine hematological profiles of all cats. NLR, NMR and LMR were calculated and compared by Mann‐Whitney *U* tests. ROC curves investigated the performance of these ratios as biomarkers at the p < 0.05 level of significance.

**Results:** SCL group (median: 8.26; range: 0.57‐94.71) had higher NLR compared to the SRE (median: 4.84; range: 0.85‐44.60; *P* = .0024) and FRE (median: 3.64; range: 0.53‐113.00; *P* = .028) groups. SCL group (median: 2.47; range: 0.28‐100.00) had lower LMR compared to the SRE (median: 6.00; range: 0.67‐88.00; *P* = .012) and FRE (median: 8.32; range: 0.50‐60.13; *P* = .001) groups. HC had lower NLR (median: 2.24; range: 0.38‐40.00) compared to the FRE, SRE, and SCL groups (all *P* < .001) and higher LMR (median:17.00;range:1.00‐341.00) compared to the FRE, SRE, and SCL groups (all *P* < .001). AUCs differentiated SCL from SRE using NLR (0.65,CI:0.52‐0.77) and LMR (0.66,CI:0.54‐0.79) and FRE from SRE using NMR (0.61,CI:0.51‐0.71).

**Conclusions:** The NLR, NMR and LMR may serve as biomarkers for differentiating phenotypes of feline CE.

## ABSTRACT GI26: A study of real‐time video capsule endoscopy for diagnosing acute vomiting in dogs

### **Dong‐In Jung** ^1^; Young Joo Kim^2^

#### ^1^College of Veterinary Internal Medicine, Gyeongsang National University, Kyongsang‐namdo, Republic of Korea; ^2^Professor, Western University of Health Sciences, Pomona, CA, USA

**Background:** In veterinary medicine, the accurate and timely diagnosis of gastrointestinal disorders in dogs is crucial for effective treatment and improved patient outcomes.

**Hypothesis/Objectives:** This study evaluated the efficiency and sensitivity of real‐time video capsule endoscopy (RT‐VCE) in detecting surgical or nonsurgical gastric lesions in an emergency veterinary setting and to evaluate patient acceptance and the ability of clinicians to make decisions using RT‐VCE data.

**Animals:** Thirteen client‐owned dogs (mean age 5.93 ± 4.27 years, mean weight 21.42 ± 9.53 kg).

**Methods:** Client‐owned dogs with an acute onset of vomiting were enrolled in this study. The dogs received antiemetics and antiacids before undergoing RT‐VCE (MC1200, MiroCam, Intromedic, South Korea). Two clinicians reviewed RT‐VCE images for the quality and presence of lesions to make a clinical decision.

**Results:** RT‐VCE exam was performed in 13 dogs. The time to reach medical decision ranged from 1 to 48 min (mean 23.61 ± 14.04 minutes). The two veterinary clinicians unanimously agreed the interpretation. Six dogs were diagnosed with gastric foreign bodies and underwent surgical retrieval. The other dogs were diagnosed with gastritis, gastric ulcerations, and hemorrhages. None of the dogs vomited from the capsule endoscopes after the procedure and no other adverse effects were observed.

**Conclusions and Clinical Importance:** RT‐VCE is an efficient, sensitive, and well‐tolerated method for identifying acute gastric lesions and foreign bodies in dogs. This study findings suggest that RT‐VCE could provide a fast, accurate, and well‐tolerated method for diagnosing and managing gastric conditions in dogs with acute vomiting.

## ABSTRACT GI27: Pilot study evaluating fecal microbiota and clinical response to novel probiotic in dogs with diarrhea

### **Chand Khanna** ^1^; Jessi Doshier^2^; Fan Yang^3^, PhD; Samuel Stewart^1^, DVM, DACVECC; Rachel Cooper^4^, DVM, DACVIM; Daisy Spear^4^, DVM; Mallory Embree^3^, PhD

#### ^1^Ethos Discovery, San Diego, CA, USA; ^2^Veterinary Specialty Hospital of San Diego (Ethos Veterinary Health), San Diego, CA, USA; ^3^Native Microbials, San Diego, CA, USA; ^4^Massachussetts Veterinary Referral Hospital (Ethos Veterinary Health), Woburn, MA, USA

**Background:** Diarrhea is one of the most common clinical signs that dogs exhibit when presenting to a veterinary clinic. Common treatments are frequently initiated empirically including dietary modification, antibiotics, and/or probiotics.

**Hypothesis/Objectives:** Assessing the utility of a novel therapeutic probiotic for the treatment of diarrhea in dogs, including its impact on the gut microbiota.

**Animals:** Single arm prospective observational pilot study consisting of ten client‐owned adult dogs presenting for diarrhea of any duration with a Purina Fecal Score (PFS) of 4‐7.

**Methods:** Tolerability and therapeutic response to the probiotic were assessed by serial clinical examination and comparison to baseline PFS. Dogs were classified as responders or non‐responders based on improvement of the PFS to <4 at day 7. Fecal samples collected at baseline, day 7 and day 56 of probiotic exposure, underwent illumina amplicon next generation sequencing of 16S rNA gene fragments (V4 region) to assess the diversity and structure of the fecal microbiome in each patient.

**Results:** No adverse events were noted in any dogs receiving probiotics. Clinical improvement in diarrhea was noted in 7/10 dogs. 80% of participants showed enhanced microbiome structure and diversity, aligning more closely with healthy references compared to baseline. Improvement in PFS was associated with an increase in anti‐diarrhea microbes in most responders and a decrease in pro‐diarrhea microbes.

**Conclusions and Clinical Importance:** This pilot study suggests a potential role of a novel probiotic in the management of diarrhea in dogs.

## ABSTRACT GI28: Comparison of clinicalpathological features between healthy yorkshire terriers and those with portosystemic shunt throughout treatment

### **Ana Rita Carvalho Pereira** ^1,2^; Júlia Camargo^1^; Fabio Teixeira^1^; Ayne Hayashi^1^; Julia Matera^1^; Carla Lorigados^1^; Marcia Gomes^1^

#### ^1^School of Veterinary Medicine and Animal Science, University of São Paulo, São Paulo, Brazil; ^2^GastroVet

**Background:** Yorkshire terriers have a heightened susceptibility to portosystemic shunt (PSS), and few laboratory parameters can indicate its progression.

**Objectives:** Comparing routine hematological and biochemical parameters between healthy Yorkshire terriers and Yorkshire terriers with portosystemic shunt at different stages of the disease.

**Animals:** Datasets from a veterinary teaching hospital comprising 22 Yorkshire terriers at the time of diagnosis of extrahepatic portosystemic shunt (PSS), during the pre‐surgical stabilization phase, and post‐surgery. These datasets also included 42 client‐owned healthy adult Yorkshire terriers up to 5 years of age, as controls.

**Methods:** Medical records from Yorkshire terriers with PSS were retrospectively reviewed and compared to datasets of health Yorkshire by non‐parametric methods.

**Results:** In healthy animals, there were higher levels of red blood cells, hemoglobin, hematocrit, hematimetric indices, platelets, total protein, albumin, alanine aminotransferase (ALT), alkaline phosphatase (ALP), urea, and creatinine, and lower levels of total leukocytes, neutrophils, and lymphocytes compared to the diseased animals at the time of diagnosis. After treatment, in post‐clinical sign absence, animals with PSS exhibited an increase in red blood cells, an increase in mean corpuscular volume (MCV), and a decrease in ALP compared to the time of diagnosis. Dogs after surgery showed an increase in hemoglobin, hematocrit, MCV, mean corpuscular hemoglobin (MCH), total protein, albumin, urea, creatinine, and cholesterol; and a reduction in total leukocytes, neutrophils, monocytes, ALT, and ALP.

**Conclusions:** Yorkshires with PSS exhibit differences in clinical pathological variables compared to healthy animals, which are minimized over the course of the treatment.

## ABSTRACT GI29: Diagnosis and therapeutic response of chronic colitis in french bulldogs

### **Ana Rita Carvalho Pereira** ^1,2^; Patricia Ishii^3^, PhD; Paula Giaretta^4^; Camila Goloni^5^, Postdoc; Ricardo Duarte^2,6,7^, DVM, MS, PhD; Fabio Teixeira^8^, PhD; DVM, MS, PhD, FMVZ

#### ^1^School of Veterinary Medicine and Animal Science, University of São Paulo, São Paulo, Brazil; ^2^GastroVet; ^3^Stonewell; ^4^GI Lab, Texas A&M University, College Station, TX, USA; ^5^FCAV/UNESP, Brazil; ^6^AllCare Vet, São Paulo, Brazil; ^7^University Center of the United Metropolitan Colleges (FMU), São Paulo, Brazil; ^8^University of São Paulo, São Paulo, Brazil

**Background:** Granulomatous colitis (GC) is a chronic enteropathy caused by adherent‐invasive strains of *E. coli* (AIEC) which French bulldogs (FB) have a high predisposition. Although, other causes of colitis may cause the same signs.

**Objective:** To compare the clinical and histopathological aspects of FB with chronic diarrhea.

**Animals:** Paraffin blocks selected from 38 FB referred to the veterinary gastroenterology service in São Paulo, Brazil, with chronic diarrhea, from 2014 to 2022. Four animals were excluded due to poor quality of biopsies and other five were excluded for absence of medical records.

**Methods:** Retrospective analysis of medical records was crossed with a blinded evaluation of hematoxylin‐eosin, periodic acid‐Schiff (PAS) and immunohistochemistry (IHC) for *E. coli* by certified veterinary pathologist. Statistical test was used to investigate difference between GC and NGC.

**Results:** GC was identified in 38.2% (13/34) of FB and 61.8% (21/34) were considered non‐GC (NGC): 32.4% had eosinophilic/neutrophilic (NE), 17.6% NI (no inflammatory) and 11.8% LP (lymphoplasmacytic). Average age was 1.3 years in GC (76.9% males) group, and 2.8 years in NGC group (76.2% males). No statistical difference between groups. All animals received drug treatment prior to biopsy, 79.3% antibiotic (n = 23) and 48.0% corticosteroid (n = 14). GC dogs had 20 times more chance to present daily diarrhea them NGCs (OR 20.0 [CI 95%: 3.1; *P* < .0043]).

**Conclusions:** Daily diarrhea is a high indicator of GC in FB with chronic diarrhea. Although diagnosis requires a colon biopsy with PAS and IHC stains to determine the presence of AIEC in the mucosa.

## ABSTRACT GI31: Bile acid dysmetabolism in feline chronic kidney disease is associated with 26 *Peptacetobacter hiranonis* variants

### **Jenessa A. Winston** ^1^; John Rowe^1^, DVM, MS; Stacie Summers^2^; Jessica Quimby^3^

#### ^1^College of Veterinary Medicine, The Ohio State University, Columbus, OH, USA; ^2^Department of Clinical Sciences, College of Veterinary Medicine, Oregon State University, Corvalis, OR, USA; ^3^Department of Veterinary Clinical Sciences, College of Veterinary Medicine, The Ohio State University, Columbus, OH, USA

**Background:** The microbial‐derived secondary bile acid (SBA) ursodeoxycholic acid (UDCA) is decreased in chronic kidney disease (CKD) cats. *Peptacetobacter hiranonis* is the only described SBA producing microbe in cats.

**Hypothesis/Objectives:** Fecal bile acid dysmetabolism occurs in CKD cats, corresponding with reduced abundance of *P. hiranonis*.

**Animals:** Healthy (n = 6) and CKD (n = 28) client‐owned cats.

**Methods:** Prospective, cross‐sectional study. Targeted liquid chromatography and tandem mass spectrometry metabolomics and 16S rRNA gene amplicon sequencing of fecal samples. Silva taxonomy and NCBI nucleotide BLAST were utilized.

**Results:** Bile acid dysmetabolism characterized by 50% SBAs (Bray‐Curtis PERMANOVA p 97% nucleotide identity to *P. hiranonis* 16S rRNA gene sequence were identified. The combined relative abundance of these 26 ASVs was significantly reduced (median 2.1%) in CKD cats with 50% SBAs (median 13.9%, Kruskal‐Wallis FDR adjusted *P* = .0002) and healthy cats with >50% SBAs (median 15.5%, Kruskal‐Wallis FDR adjusted *P* = .0112). Conventional qPCR primers for *P. hiranonis* would have identified 23/26 (88.4%) of the ASVs.

**Conclusions and Clinical Importance:** Fecal bile acid dysmetabolism characterized by <50% SBAs occurs in CKD cats. The dysmetabolism is partially explained by reduced abundance of *P. hiranonis*. Currently, methodologies to detect *P. hiranonis* capture similar microbial genetic signatures, but do not provide information regarding functional genetic potential to produce SBAs.

## ABSTRACT HM01: Metabolic and genomic analyses in a dog with green urine identify a biliverdin reductase defect

### **Jade Peralta** ^1^; Eva Furrow^2^; Urs Giger^3^; Katie Minor^2^; A. Russell Moore^4^

#### ^1^College of Veterinary Medicine and Biomedical Sciences, Colorado State University, Fort Collins, CO, USA; ^2^Department of Veterinary Clinical Sciences, University of Minnesota, Minneapolis, MN, USA; ^3^Vetsuisse Faculty, University of Zürich, Zürich, Switzerland; ^4^Department of Microbiology, Immunology, and Pathology, Colorado State University, Fort Collins, CO, USA

**Background:** In the process of heme degradation, biliverdin reductase catalyzes the conversion of biliverdin to bilirubin. While rare defects in the biliverdin reductase A gene (BLVRA) cause biliverdinuria in human patients, this disorder has not yet been reported in dogs.

**Hypothesis/Objectives:** The objective was to identify biliverdinuria and the pathogenic BLVRA variant in a dog with green urine.

**Animals:** A three‐year‐old mixed breed dog with green urine compared to control dogs, including a database of whole genome sequencing variants.

**Methods:** Besides routine clinicopathological evaluations, urinary mass spectroscopy and whole genome sequencing studies focusing on BLVRA were performed.

**Results:** The mixed breed dog exhibited persistently green urine since juvenile age. The dog also had a chronic regenerative anemia which remained unexplained. Urine metabolic screening of bilirubin pathway metabolites revealed increased biliverdin concentrations. Whole genome sequencing revealed a 19 kb homozygous deletion in the BLVRA gene that spanned the final three of seven exons (ENSCAFT00805017018.1, p.Gly118‐*297del) predicting a major truncation or premature mRNA decay. None of the 671 dogs from 63 breeds in the genomic database were homozygous for the BLVRA deletion.

**Conclusions and Clinical Importance:** This study documents biliverdinuria due to a major BLVRA deletion as the cause of persistent green urine in a dog. To the authors' knowledge, this is the first confirmed case of biliverdinuria in a dog and illustrates the use of targeted metabolic and genomic screening as key diagnostic tools. Genotyping for the BLVRA deletion may help identify other affected dogs.

## ABSTRACT HM02: Temporal associations between vaccination and onset of immune‐mediated hemolytic anemia or thrombocytopenia in dogs

### **Victoria Neale** ^1^; Sophie Broughton^2^; Barbara Glanemann^2^; Barbara Skelly^3^; James Swann^4^; Amanda Paul^5^

#### ^1^Anderson Moores Veterinary Specialists, Winchester, UK; ^2^Department of Clinical Science and Services, Royal Veterinary College, London, UK; ^3^Department of Veterinary Medicine, University of Cambridge, Cambridge, UK; ^4^Columbia Stem Cell Initiative, New York, NY, USA; ^5^Anderson Moores Veterinary Specialists, Winchester, UK

**Background:** Vaccination has been implicated in development of human immune‐mediated diseases, but previous studies investigating similar associations in dogs yielded conflicting results for immune‐mediated hemolytic anemia (IMHA) and thrombocytopenia (ITP).

**Hypothesis/Objectives:** To investigate temporal relationships between onset of IMHA or ITP and vaccination in client‐owned dogs. We hypothesized that the proportion of dogs vaccinated within 1 month would be higher in the IMHA/ITP groups compared to controls.

**Animals:** Client‐owned dogs with non‐associative IMHA (n = 270) and ITP (130) presented to 3 referral hospitals from 2010 through 2022, alongside age‐matched dogs with non‐immune mediated disease (n = 1080 for IMHA, 520 for ITP) presented contemporaneously to the same institutions.

**Methods:** A multicenter retrospective case control study was conducted. Fisher's exact test was used to compare the proportion of dogs with IMHA or ITP that were vaccinated in the month before disease onset compared to control dogs.

**Results:** The proportion of dogs vaccinated within 1 month of diagnosis was significantly higher in the IMHA group (33/270, 12.2%) compared with matched controls (68/1080, 6.3%, *P* = .002). There was no difference in the proportion of dogs with ITP that were vaccinated in the month before diagnosis compared to controls (16/130, 12.3% versus 43/520, 8.3%, *P* = .17). Dogs with IMHA vaccinated in the month before diagnosis were significantly older than those vaccinated at other times, but there were no differences in clinicopathological variables.

**Conclusions and Clinical Importance:** Our findings support a possible temporal association between vaccination and onset of IMHA in dogs, but confirm lack of association for ITP.

## ABSTRACT HM03: Diagnostic assessment of point‐of‐care scanning system integrated with deep‐learning algorithms for canine/feline blood film evaluation

### **Eric Morissette** ^1^; Cory Penn, DVM^2^

#### ^1^Zoetis, Parsippany, NJ, USA; ^2^Head of Imagyst Platforms, GDx Medical Affairs/US Diagnostics and Business Solutions, Global Diagnostics, Zoetis, Parsippany, NJ, USA

**Background:** Comprehensive hematologic assessments in canines and felines involve both quantitative (cell counts) and qualitative (blood smear) analyses. Analyzing blood smears pose challenges due to technique variations, training disparities, workflow complexities, and time constraints impeding routine blood smears review.

**Objectives:** Assess the performance of the Vetscan Imagyst (VS‐I) Blood Smear application, an artificial intelligence algorithm designed for hematologic analysis and compare it to ACVP board‐certified clinical pathologists (CPs). Objectives included: determination of accuracy of monolayer detection, WBC estimate, WBC differential, polychromatophil count, nucleated RBC count, and platelet estimate.

**Method:** Blood smears (119 total, 59 dogs, and 60 cats) were retrospectively collected from Zoetis Reference Labs. The Grundium Ocus 40 was used to scan all slides. A randomized 2 out of 4 CPs and the VS‐I blood smear algorithm evaluated the samples. The agreement between VS‐I and the CPs was assessed.

**Results:** The percentage of samples where VS‐I was within 99% prediction interval with the CPs for each white blood cell class differential ranged from 93.2% to 100% for dogs and 88.3%‐100% for cats (Table 1). The number of samples where VS‐I was within 95% prediction interval with the CPs for each cell class estimated number ranged from 69.5 to 95.0% for dogs and 76.3%‐95.0% for cats. (Table 2).

**Conclusion/Significance:** The VS‐I Blood Smear application demonstrated strong performance with results comparable to ACVP‐board‐certified clinical pathologists hematologic assessments making it a tool for utilization by veterinarians to obtain a comprehensive CBC.

**Table:** **Table 1.** Vetscan Imagyst Blood Smear application performance for samples within 99% prediction interval with ACVP‐certified pathologists for each cell class differential number for canine and feline samples

Species	Cell type	Number of slides within 99% prediction interval	Total slides	%
Canine	Neutrophil %	55	59	93.2%
	Lymphocyte %	57	59	96.6%
	Monocyte %	57	59	96.6%
	Eosinophil %	59	59	100.0%
	Basophil %	59	59	100.0%
Feline	Neutrophil %	53	60	88.3%
	Lymphocyte %	55	60	91.6%
	Monocyte %	57	60	95.0%
	Eosinophil %	59	60	98.3%
	Basophil %	60	60	100.0%

**Table:** **Table 2.** Vetscan Imagyst Blood Smear application performance for samples within 95% prediction interval with ACVP‐certified pathologists for each cell class estimated number for canine and feline samples

Species	Cell class count	Number of slides within 95% prediction interval	Total slides	%
Canine	Est WBC	55	59	93.2%
	Est platelets	52	59	88%
	Est polychromatophil	51	59	86.4%
	Est nRBC	41	59	69.5%
Feline	Est WBC	53	60	88.3%
	Est platelets	48	60	80.0%
	Est polychromatophil	57	60	95.0%
	Est nRBC	44	59	74.6%

## ABSTRACT HM05: The in vitro effects of acidemia and acidemia reversal on coagulation in dogs

### **DoHyeon Yu** ^1^; Youngju Kim^2^; Hyeona Bae^2^

#### ^1^Gyeongsang National University, Kyongsang‐namdo, Republic of Korea; ^2^College of Veterinary Medicine, Gyeongsang National University, Kyongsang‐namdo, Republic of Korea

**Background:** The effect of acidemia on blood coagulation remains inadequately understood in veterinary medicine.

**Objectives:** To assess the effect of in vitro acidification of canine whole blood on coagulation, and to investigate whether acidemia‐induced coagulopathy could be reversed by reversing acidemia.

**Methods:** Citrated whole blood samples were taken from six healthy beagle dogs and categorized based on pH adjustment into: neutral, weak acidemia (WA), strong acidemia (SA), and reversal from SA. Then prothrombin time (PT), activated partial thromboplastin time (aPTT), fibrinogen concentration, conventional thromboelastography (TEG) parameters, and velocity curve (V‐curve) variables of TEG were assessed.

**Results:** The PT, aPTT, and most of TEG parameters showed significant coagulopathy in the SA group when compared to neutral group, with additional significant changes in reaction time (R), clot kinetic (K), maximum amplitude (MA), split point (SP), elasticity (E), thrombodynamic potential index (TPI), and coagulation index (CI) between the SA and WA groups. Among V‐curve variables, the maximum rate of thrombus generation (MRTG) and total thrombus generation (TG) were significantly inhibited in the SA group compared to the neutral group, with significant differences in the time to maximum rate of thrombus generation (TMRTG) between the WA and SA groups. In the reverse group, aPTT, R, K, α‐angle, MRTG, TMRTG, SP, TPI, and CI exhibited significant recovery compared to the SA group.

**Conclusions:** In vitro induction of acidemia in canine whole blood leads to impairment of coagulation profiles, and pH correction can reverse most acidemia‐induced coagulopathy.

## ABSTRACT HM06: Evaluation of circumferential securing tape around blood pressure cuffs on doppler ultrasound blood pressure measurements

### **Alejandra B. Príncipe Martínez** ^1^; Lauren Cochran^1^, DVM, DACVIM (SAIM); Anthony Ishak^1^, DVM, DACVIM (SAIM, Cardiology); Deborah Keys^2^

#### ^1^BluePearl Pet Hospital, FL, USA; ^2^Kaleidoscope Statistics, Athens, GA, USA

**Background:** Secondary fastening methods are used off‐label to secure blood pressure cuffs (BPCs) with worn hook‐and‐loop fasteners. However, the impact of circumferential secondary fasteners on systolic blood pressure readings is unknown.

**Hypothesis/Objectives:** The objective of this study was to compare systolic blood pressure (SBP) measurements between BPCs with and without a secondary fastener in healthy, non‐anesthetized dogs. We hypothesize that SBP measurements would not be clinically different (±10 mmHg) when performed with BPCs secured with hook‐and‐loop or circumferential medical tape.

**Animals:** Twelve healthy blood donor dogs and 16 apparently healthy staff‐owned dogs.

**Methods:** SBP measurements with and without tape on the BPC were performed using Doppler ultrasound. Each method was performed six consecutive times, with the final five values averaged for statistical analyses. Bland‐Altman plots were made, and limits of agreement calculated.

**Results:** The limits of agreement were −40.8 (95% CI −55.6 to 26.0) to 45.6 (95% CI 30.8‐60.4), indicating that the Doppler SBP measured with tape would be expected to be between 40.8 mmHg below to 45.6 mmHg above those measured without tape 95% of the time. The mean bias estimate was 2.4 mmHg (SD = 22.0, 95% CI −6.1 to 11.0), indicating that SBP measurements with tape averaged 2.4 mmHg higher than without tape.

**Conclusions and Clinical Importance:** SBP measurements with secondary fasteners on the BPCs differed by more than 10 mmHg compared to the BPCs standard hook‐and‐loop fasteners, and we rejected our hypothesis. BPCs with dysfunctional hook‐and‐loop fasteners should be replaced due to poor clinical reliability of SBP measurements.

## ABSTRACT HP01: Feasibility of shear wave elastography for inflammation and fibrosis in dogs with hepatic disease

### **Nozomi Shiohara** ^1^; Kensuke Nakamura^1^; Nozomu Yokoyama^1^; Yumiko Kagawa^2^; Yong Bin Teoh^3^; Mitsuyoshi Takiguchi^1^

#### ^1^Hokkaido University, Sapporo, Hokkaido, Japan; ^2^North Lab, Sapporo, Hokkaido, Japan; ^3^University of Saskatchewan, Saskatoon, SK, Canada

**Background:** Shear wave elastography (SWE) is an ultrasound technique, which measures shear wave speed (SWS), an index of viscoelasticity, and dispersion slope (DS), an index of viscosity. In the liver, fibrosis elevates elasticity, while congestion or inflammation elevates viscosity.

**Hypothesis/Objectives:** To investigate the usefulness of SWE to evaluate inflammation and fibrosis in dogs with hepatic disease.

**Animals:** Forty client‐owned dogs which underwent liver biopsy.

**Methods:** In this cross‐sectional prospective study, SWS (m/s) and DS [(m/s)/Hz] were measured using SWE in dogs that underwent surgical or laparoscopic liver biopsy. Stages of inflammation and fibrosis were scored according to the WSAVA diagnostic criteria (inflammation: A score 0‐5, fibrosis: F score 0‐4). Dogs were classified into the following three groups based on A and F scores: Control group (A0 F0), inflammation group (A ≥ 1 F ≤ 1), fibrosis group (F ≥ 2).

**Results:** SWS was significantly higher in fibrosis group (1.93, IQR:1.83‐2.21) than control group (1.36, IQR:1.32‐1.39) and inflammation group (1.45, IQR:1.34‐1.63) but there was no difference between control and inflammation group. DS was significantly higher in inflammation group (14.80, IQR:14.40‐16.50) and fibrosis group (16.50, IQR:14.50‐17.20) than control group (12.25, IQR:11.95‐13.18). DS (AUC = 0.992) was superior to SWS (AUC = 0.717) in discriminating between control group and inflammation group. SWS strongly correlated with F score (*Ρ* = .83) and moderately with A score (*Ρ* = .52). Whereas DS strongly correlated with A score (*Ρ* = .88) and moderately with F score (*Ρ* = .58).

**Conclusions and Clinical Importance:** Elevated SWS reflects fibrosis and elevated DS reflects inflammation. SWE can be useful in assessing inflammation and fibrosis for dogs with hepatic disease.

## ABSTRACT HP02: Detection and phylogenetic analysis of domestic cat hepadnavirus in blood from cats in texas

### **Min‐Chun Chen** ^1^; Yan Ru Choi^2^; Julie Piccione^3^; João Pedro Cavasin^1^; Jonathan Lidbury^1^; Jörg Steiner^1^; Julia Beatty^4^

#### ^1^Gastrointestinal Laboratory, Texas A&M University, College Station, TX, USA; ^2^Jockey Club College of Veterinary Medicine and Life Sciences, City University of Hong Kong, Kowloon, Hong Kong; ^3^Veterinary Medical Diagnostic Laboratory, Texas A&M, College Station, TX, USA; ^4^Department of Veterinary Clinical Sciences, Jockey Club College of Veterinary Medicine and Life Sciences, City University of Hong Kong, Kowloon, Hong Kong

**Background:** Domestic cat hepadnavirus (DCH), a hepatotropic virus related to hepatitis B virus (HBV), has been detected globally with molecular prevalence ranging from 0.2% to 18%. DCH presence in Texas has yet to be investigated.

**Objective:** Determine the prevalence of DCH viremia in cats undergoing diagnostic investigation in Texas.

**Animals:** 400 residual feline EDTA blood samples submitted to the Texas A&M Veterinary Medicine Diagnostic Laboratory between December 2022 and September 2023.

**Methods:** DNA extracted from blood was tested for DCH using qPCR. PCR amplicons obtained by conventional PCR using overlapping primers were used to sequence DCH positive samples.

**Results:** 3/400 samples (0.7%; 95% CI: 0.2%‐2%) tested positive for DCH DNA, with viral loads of 2.78 × 10^6^, 6.08 × 10^2^, and 2.96 × 10^4^ copies/mL. The low prevalence of DCH viremia precluded risk factor analysis. Whole genome sequence of DCH was obtained from one DCH‐positive sample. The Texas DCH strain has the closest nucleotide similarity (98.5%) to a DCH strain from Hong Kong. In phylogenetic analysis, the Texas strain clustered with the Australian 2016 Sydney DCH (MH307930), DCH Hong Kong (OP643862), and two DCH strains from Italy (OQ859620, OQ859619).

**Conclusions and Clinical Importance:** The prevalence of DCH viremia in Texas of 0.7% was similar to those previously reported in North America (0.2%), South America (1.67%), and Japan (0.78%). Phylogenetic analysis suggests the sequenced DCH strain in Texas is closely related to DCH strains in Australia, Hong Kong, and Italy.

## ABSTRACT HP03: Comprehensive gene expression analysis in gallbladder mucosal epithelial cells of dogs with gallbladder mucocele

### **Itsuma Nagao** ^1^; Tomoki Motegi^2^; Yuko Goto‐Koshino^1^; Kazuyuki Uchida^1^; James Chambers^1^; Kenji Baba^3^; Hirotaka Tomiyasu^1^

#### ^1^The University of Tokyo, Tokyo, Japan; ^2^Division of Computational Biomedicine, Department of Medicine, Boston University School of Medicine, Boston University, Boston, MA, USA; ^3^Yamaguchi University, Yamaguchi, Japan

**Background:** Gallbladder mucocele (GBM) is one of the most common diseases that occur in canine gallbladder. Although the pathogenesis of GBM remains unclear, we have recently reported that the excessive accumulation of mucin in the gallbladder is not due to its overproduction by gallbladder epithelial cells (GBECs).

**Hypothesis/Objectives:** We hypothesized that the changes in the functions of GBECs other than the production of mucin were associated with the pathogenesis of GBM. The objective was set to investigate the abnormalities in gene expression profiles of GBECs in cases with GBM.

**Animals:** Fifteen dogs with GBM and eight dogs euthanized for reasons other than gallbladder diseases were included.

**Methods:** GBECs were isolated from gallbladder tissues, and RNA was extracted from GBECs. RNA‐seq was performed using the samples of three GBM cases and three dogs with normal gallbladder, and the gene expression profiles were compared between the two groups. The differences in mRNA expression levels of the extracted differentially expressed genes (DEGs) were validated between the two groups using the samples of 15 GBM cases and eight dogs with normal gallbladder.

**Results:** Comparison of gene expression profiles by RNA‐seq extracted 367 DEGs, including ANO1, a chloride channel associated with changes in mucin morphology, and HTR4, which regulates the function of chloride channels. ANO1 and HTR4 were confirmed to be downregulated in the GBM group by RT‐qPCR.

**Conclusions and Clinical Importance:** The results of this study suggest that GBM can be developed by the decreased function of chloride channels expressed in GBECs.

## ABSTRACT HP04: Point‐of‐care viscoelastometric evaluation of dogs with congenital intrahepatic portosystemic shunts

### **Floris C. Dröes** ^1^; Christine Rutter^2^, DVM, DACVECC; Adrian Tinoco Najera^3^; Robert Phillips^3^; Joerg Steiner^4^; Jonathan Lidbury^5^

#### ^1^Gastrointestinal Laboratory, School Of Veterinary Medicine and Biomedical Sciences, Texas A&M University, College Station, TX, USA; ^2^Clinical Associate Professor, Emergency and Critical Care Service, Small Animal Clinical Sciences, Texas A&M University, College Station, TX, USA; ^3^Graduate Assistant Research, Gastrointestinal Laboratory, Small Animal Clinical Sciences, Texas A&M University, College Station, TX, USA; ^4^Regents Professor, University Distinguished Professor, Dr. Mark Morris Chair in Small Animal Gastroenterology and Nutrition, Director of Gastrointestinal Laboratory, Gastrointestinal Laboratory, Small Animal Clinical Sciences, Texas A&M University, College Station, TX, USA; ^5^Associate Professor in Small Animal Internal Medicine, Associate Director for Clinical Services, Assistant Department Head of Research and Graduate Studies, Gastrointestinal Laboratory, Small Animal Clinical Sciences, Texas A&M University, College Station, TX, USA

**Background:** Abnormal liver function that occurs in dogs with congenital portosystemic shunts (CPSS) can lead to various hemostatic abnormalities. Plasma‐based coagulation tests cannot reliably identify abnormalities such as hypercoagulability and hyperfibrinolysis.

**Objective:** Evaluate hemostasis in dogs with intrahepatic CPSS (IHPSS) using a point‐of‐care viscoelastic coagulometer (VCM Vet) and compare the findings to traditional plasma‐based coagulation tests.

**Animals:** 42 healthy control dogs (HC) and 19 dogs with IHPSS.

**Methods:** Observational cross‐sectional study. Prothrombin time (PT), partial thromboplastin time (PTT), concentrations of fibrinogen and D‐dimers, antithrombin activity (AT), platelet count (PLT), and viscoelastic coagulometer parameters were concurrently evaluated before shunt attenuation. Results were compared between HC and IHPSS dogs using Mann‐Whitney U‐tests or t‐tests, as appropriate, with corrections for multiple comparisons.

**Results:** In 7 of 10 (70%) IHPSS dogs PT was prolonged, in 2/10 (20%) PTT was prolonged, and 4/19 (21.1%) showed viscoelastometric abnormalities that were classified as hypocoagulable 3/19 (15.8%), hypercoagulable 1/19 (5.3%), and hyperfibrinolytic 2/19 (10.5%). AT and PLT were significantly decreased, and clotting time significantly increased in IHPSS dogs compared to HC (Table 1).

**Conclusions and Clinical Importance:** Some IHPSS dogs showed viscoelastometric abnormalities. This suggests a variety of hemostatic states, such as hypercoagulability and hyperfibrinolysis, that might not be reliably identified by plasma‐based coagulation testing in these patients.

**Table:** **Table 1.** Plasma‐based coagulation test results and viscoelastometric parameters in healthy dogs (HC) and dogs with intrahepatic congenital portosystemic shunts (IHPSS). *P* < .0036 is considered significant after correction for multiple comparisons

Test parameter	HC n = 42 (Mean ± SD or median [IQR])	IHPSS n = 19 (Mean ± SD or median [IQR])	*P* value
Prothrombin time (seconds)	7.7 ± 0.1	9.8 ± 0.3	<.0001*
Partial thromboplastin time (seconds)	12.7 [10.6‐13.9]	19.9 [15.6‐25.1]	<.0001*
Fibrinogen (mg/dL)	168 [141‐194]	141 [133‐179]	.1016
D‐Dimers (ng/mL)	327 [209‐467]	524 [222‐852]	.1896
Antithrombin (%)	136 ± 2	68 ± 5	<.0001*
Platelets (x 10^3^/μL)	214 ± 9	152 ± 13	.0007*
Clotting time (seconds)	334 [301‐357]	387 [362‐466]	<.0001*
Clot formation time (seconds)	197 [176‐232]	196 [161‐237]	.9751
Alpha	54 [50‐56]	51 [46‐59]	.1904
Amplitude at 10 minutes	20.4 ± 0.6	21.6 ± 0.9	.2562
Amplitude at 20 minutes	26.9 ± 0.7	28.3 ± 1.0	.2310
Maximum clot formation	33.2 ± 0.8	33.7 ± 1.2	.7430
Lysis index at 30 minutes (%)	100 [100‐100]	100 [100–100]	.0073
Lysis index at 45 minutes (%)	100 [100‐100]	100 [99‐100]	.0018*

## ABSTRACT HP05: Hepatic copper accumulation in dogs: an exploratory study on assessment of dietary factors

### **Deborah E. Linder** ^1^; Cynthia Webster^2^

#### ^1^Tufts University, MA, USA; ^2^Cummings School of Veterinary Medicine, Tufts University, North Grafton, MA, USA

**Background:** The incidence of canine copper associated hepatopathy has increased. While genetic predispositions for hepatic copper accumulation exist, environmental exposure through dietary excess could be a risk factor.

**Hypothesis/Objectives:** To describe demographics and hepatic histopathology in dogs with hepatic biopsy copper quantification and to explore dietary risk factors in a cohort of dogs with normal (NHC) and high hepatic copper (HHC).

**Animals:** Retrospective study of 302 dogs with hepatic biopsies including copper quantification (2014‐2023) at an academic institution.

**Methods:** Breed, sex, spay‐neuter status, age, histopathologic findings, and hepatic copper quantification by atomic absorption spectroscopy were recorded. Diet histories were obtained from records and phone interview for 2 cohorts of dogs with NHC (n = 36) and HHC (n = 33).

**Results:** 171/302 (56%) of dogs had HHC (>400 PPM) and these dogs often had inflammatory/fibrotic disease. There was no difference in age, sex, or neuter status between NHC or HHC dogs. 34% of HHC dogs were mixed breed. More HHC dogs (66%) than NHC dogs (13%) drank private well water (*P* < .004. Most dogs had a history of multiple diets, treats, and supplements; however, more HHC dogs (45%) than NHC dogs (28%) had a history of high copper ingredients throughout their diet.

**Conclusions and Clinical Importance:** There is a high incidence of HHC levels in many breeds. Retrospectively quantifying accurate dietary copper intake was challenging but helped to define relevant information to gather in future prospective studies including drinking water sources, geographic information, and copper content from current and previously fed diets, treats, and supplements.

## ABSTRACT HP06: Untargeted profiling of serum metabolites in dogs with chronic hepatitis

### **Adrian Tinoco‐Najera** ^1^; Robert Phillips^1^; Floris Dröes^1^; Amanda Blake^1^; Patricia Ishii^1,2^; João Cavasin^1^; Joerg Steiner^1^; Jan Suchodolski^1^; Jonathan Lidbury^1^

#### ^1^Gastrointestinal Laboratory, Texas A&M University, College Station, TX, USA; ^2^Stonewell Gastrointestinal Vet, São Paulo, Brazil

**Background:** Definitive diagnosis of chronic hepatitis (CH) in dogs requires histopathological assessment. Noninvasive biomarkers of CH would be beneficial. Metabolomics facilitates the description of disease pathophysiology, progression, and therapeutic response at the metabolic level.

**Objective:** Describe differences in the serum metabolomic profiles of dogs with CH in comparison to healthy control (HC) dogs and dogs with chronic enteropathy (CE).

**Animals:** Serum samples from 16 dogs with CH, 16 dogs with CE, and 16 HC dogs.

**Methods:** Retrospective cross‐sectional study. Untargeted metabolomic analysis was performed by ultra‐high performance liquid chromatography/tandem accurate mass spectrometry. Univariate analyses among CH, CE, and HC dogs were performed using Kruskal‐Wallis tests. P‐values were adjusted for multiple comparisons. Significance was set at q < .05.

**Results:** A total of 140 out of 959 metabolites were significantly different among groups. Twenty‐six metabolites were uniquely increased in the CH group. The most remarkable peak intensities included aromatic amino acids (phenylalanine [q = 0.008]), organic acids (formiminoglutamate [q = 0.003]), phospholipids (sphinganine 1‐phosphate [q = 0.002]), cofactors (quinolinate [q = 0.02]), peptides (gamma glutamylphenylalanine [q = 0.001]), and nucleotides (3‐ureidoisobutyrate [q = 0.0002]). Principal component analysis and heat dendrograms revealed distinctive clustering and metabolomic profiles among the three groups.

**Conclusions and Clinical Importance:** Dogs with CH have a distinct serum metabolite profile compared to dogs with CE or HC dogs. Targeted assessment of these differentially expressed metabolites is necessary to confirm this and to determine their clinical utility.

## ABSTRACT HP07: Evaluation of gallbladder motility and cholestasis by hepatobiliary scintigraphy in dogs with sludge

### **Jiesong Woo**; Dong Seop Lee; Hyein Jung; Yeon Chae; Yeongjae Yoo; Taesik Yun; Hakhyun Kim; Byeong‐Teck Kang

#### Laboratory of Veterinary Internal Medicine, College of Veterinary Medicine, Chungbuk National University, Ch'ungch'ong‐bukto, Republic of Korea

**Background:** Gallbladder (GB) sludge has been proposed to be related to decreased GB motility and induce cholestasis in dogs. Hepatobiliary scintigraphy is known as a non‐invasive diagnostic method for assessing gallbladder function and cholestasis, but it has not been studied in dogs with biliary sludge.

**Hypothesis/Objectives:** This study aimed to quantitatively compare GB motility and cholestasis in dogs with GB sludge to control dogs using 99mTc‐mebrofenin hepatobiliary scintigraphy.

**Animals:** Sixteen healthy controls and ten dogs with either mobile or immobile biliary sludge were prospectively enrolled.

**Methods:** All dogs received 2–3 mCi of 99mTc‐mebrofenin intravenously, followed by dynamic imaging for 10 minutes and static imaging at 30, 45, and 60 minutes. After the acquisition of 60‐minute images, dogs were fed high fat diet and additional images were taken 30‐ and 60‐minutes post‐meal.

**Results:** Dogs with immobile biliary sludge had significantly lower EF30 (%) (mean ± SD: 24.39 ± 20.08; *P* = .048) and EF60 (41.06 ± 18.82; *P* = .002) compared with controls (EF30, 50.16 ± 19.22; EF60, 71.47 ± 14.43). The immobile sludge group also had significantly delayed *T* ^max^ (9.36 ± 1.86; *P* = .018) and T^½^ (median [range]: 12.20 [12‐45]; *P* = .028), which demonstrates hepatic clearance, compared to the control (*T* ^max^, 5.22 ± 2.26; T^½^, 10.50 [6‐15]).

**Conclusions and Clinical Importance:** This is the first study to quantitatively demonstrate decreased gallbladder ejection fraction and hepatic clearance using hepatobiliary scintigraphy in dogs with immobile sludge.

## ABSTRACT HP08: Evaluation of serum immunoglobulin g in dogs diagnosed and treated for immune‐mediated chronic hepatitis

### **Tarini V. Ullal** ^1^; Sarah Shropshire^2^, DVM, PhD, DACVIM (SAIM); Jennifer Hawley^3^; David Twedt^4^, DVM, DACVIM (SAIM)

#### ^1^University of California‐Davis, Davis, CA, USA; ^2^Assistant Professor, Clinical Sciences, Colorado State University, Fort Collins, CO, USA; ^3^Clinical Sciences, Colorado State University, Fort Collins, CO, USA; ^4^Professor Emeritus, Clinical Sciences, Colorado State University, Fort Collins, CO, USA

**Background:** Serum immunoglobulin G (IgG) is a marker of auto‐immune hepatitis in humans and could have application in dogs with Immune‐mediated chronic hepatitis (ICH).

**Hypothesis/Objectives:** Evaluate the utility of serum IgG to (1) differentiate ICH from copper‐associated chronic hepatitis (CAH) and (2) monitor treatment response in dogs with ICH. Serum IgG was expected to be elevated in ICH dogs and decrease with treatment.

**Animals:** Nineteen client‐owned dogs with ICH and 15 dogs with CAH.

**Methods:** Dogs were prospectively diagnosed with ICH or CAH based on histologic criteria and treated for 6 months. Dogs with ICH were treated with cyclosporine ± copper‐restricted diet if hepatic copper was ≥1000 μg/g dw. Dogs with CAH were treated with D‐penicillamine and a copper‐restricted diet. Serum IgG was measured using a radial immunodiffusion kit at baseline, 1, 3, and 6 months into treatment. A Wilcoxon‐rank sum test compared IgG at baseline between dogs with ICH and CAH. A mixed effects model on log transformed data with Tukey's pairwise comparisons evaluated IgG at baseline compared to each timepoint during treatment.

**Results:** Serum IgG (mg/dL) [median (Q1‐Q3)] was not significantly different at baseline between dogs with ICH [2680 (2028‐3176)] and CAH [2197 (1952‐4324)] (*P* = .84). Serum IgG minimally increased from baseline to 6 months (*P* = .047) in dogs with ICH and did not change during treatment of CAH (*P* = .29).

**Conclusions and Clinical Importance:** Serum IgG was not a useful marker to diagnose or monitor dogs with ICH.

## ABSTRACT HP09: Evaluation of coagulation parameters in dogs with immune‐mediated chronic hepatitis and copper‐associated chronic hepatitis

### **Tarini V. Ullal** ^1^; Sarah Shropshire^2^, DVM, PhD, DACVIM (SAIM); David Twedt^3^, DVM, DACVIM (SAIM)

#### ^1^University of California‐Davis, Davis, CA, USA; ^2^Assistant Professor, Clinical Sciences, Colorado State University, Fort Collins, CO, USA; ^3^Professor Emeritus, Clinical Sciences, Colorado State University, Fort Collins, CO, USA

**Background:** Coagulation derangements have been previously reported in dogs with chronic hepatitis.

**Objectives:** Determine frequency of coagulation abnormalities in a cohort of immune‐mediated chronic hepatitis (ICH) and copper‐associated chronic hepatitis (CAH) dogs undergoing pre‐ and post‐treatment liver biopsies.

**Animals:** Client‐owned dogs diagnosed with ICH (18) or CAH (14).

**Methods:** Dogs were diagnosed with ICH or CAH following a liver biopsy and treated for 6 months. Clotting times (PT and PTT) and fibrinogen were measured at 1, 3, and 6 months. A liver biopsy was repeated at 6 months. Prolonged PT or PTT was defined as >1.25 times upper limit of normal. Descriptive statistics were performed.

**Results:** At baseline, all dogs had normal clotting times except for 1 CAH dog that had prolonged clotting times and hypofibrinogenemia. Hypofibrinogenemia and hyperfibrinogenemia was observed in 6/32 and 2/32 dogs, respectively. At 6 months, all dogs had normal PT/PTT and 15/32 (47%) had hyperfibrinogenemia (12 ICH and 3 CAH). At the baseline biopsy, Gelfoam was used for hemostasis in 7/32 (22%) dogs of which 1/7 had a coagulation derangement (fibrinogen <60 mg/dL). At the 6 month biopsy, Gelfoam was used for hemostasis in 3/32 (9%) of which all 3 dogs had normal PT/PTT and hyperfibrinogenemia.

**Conclusions and Clinical Importance:** Prolonged clotting times, hypofibrinogenemia, and bleeding complications requiring Gelfoam were uncommon in this cohort of ICH and CAH dogs. Hyperfibrinogenemia was found in >50% of dogs post‐treatment and was more common in ICH than CAH.

## ABSTRACT HP10: Urinary copper:zinc ratios in dogs with immune‐mediated chronic hepatitis and copper‐associated hepatitis

### **Tarini V. Ullal** ^1^; Sarah Shropshire^2^, DVM, PhD, DACVIM (SAIM); David Twedt^3^, DVM, DACVIM (SAIM)

#### ^1^University of California‐Davis, Davis, CA, USA; ^2^Assistant Professor, Clinical Sciences, Colorado State University, Fort Collins, CO, USA; ^3^Professor Emeritus, Clinical Sciences, Colorado State University, Fort Collins, CO, USA

**Background:** Urinary copper: zinc ratios (Cu:Zn) could be a valuable biomarker of copper‐associated chronic hepatitis (CAH).

**Objectives:** Compare urinary Cu:Zn between: (1) CAH, immune‐mediated chronic hepatitis (ICH), and healthy control dogs (2) pre‐ and post‐treatment in dogs with CAH.

**Animals:** 18 dogs with CAH, 18 dogs with ICH, 11 healthy controls.

**Methods:** Urinary Cu and Zn were measured prospectively using flame atomic absorption spectroscopy. Dogs with CAH had hepatic copper ≥1500 ppm, copper pigment granulomas, and minimal to no interface hepatitis. Dogs with ICH had interface hepatitis. Dogs with CAH were treated with D‐penicillamine (10‐15 mg/kg PO q 12) and a copper‐restricted diet for 6 months and re‐biopsied. Wilcoxon rank sum test compared Cu:Zn between ICH, CAH, and healthy controls at diagnosis. Wilcoxon signed rank test compared Cu:Zn pre‐ and post‐treatment in dogs with CAH. A Spearman's correlation examined the relationship between Cu:Zn and hepatic copper in dogs treated for CAH.

**Results:** Urinary Cu:Zn was higher at diagnosis of CAH and ICH compared to healthy dogs (*P* = 0.002), but no different between CAH and ICH (*P* = .40). Urinary Cu:Zn post‐treatment of CAH was significantly lower compared to pre‐treatment (*P* = .01) and no different to healthy dogs (*P* = .5), but did not correlate with hepatic copper post‐treatment (*P* = .29, *r* ^2^ = 0.32).

**Conclusions and Clinical Importance:** Urinary Cu:Zn did not differentiate between CAH and ICH, but ratios significantly lowered during treatment of CAH. Urinary Cu:Zn could be a useful marker to monitor treatment response in dogs with CAH.

## ABSTRACT ID01: Evaluation of new immunotherapy in cats with feline infectious peritonitis being treated with EIDD‐2801 antiviral

### **Petra Cerna**; Steven Dow; McKenna Willis; Kaci Shaw; Jennifer Hawley; Michael Lappin

#### Colorado State University, Fort Collins, CO, USA

**Background:** Antiviral drugs such as GS‐441524 and EIDD‐2801 have been successful in treatment of feline infectious peritonitis (FIP), but only remdesivir is currently legally available in the USA; antiviral drugs can act synergistically with immunomodulatory treatments to improve patient outcome and survival in different viral diseases.

**Hypothesis/Objectives:** To determine the efficacy of oral liposome‐toll‐like receptor (TLR3 and TLR9) agonist complex (LTC) as an adjunct treatment of cats being treated with EIDD‐2801.

**Animals:** Eleven client‐owned cats.

**Methods:** Placebo‐controlled clinical trial in cats diagnosed with FIP.

**Results:** Eleven cats diagnosed with FIP have completed 12 weeks of therapy with repeated bloodwork (7 cats in LTC and 4 cats in placebo group). Six cats were effusive (5 peritoneal and 1 pleural effusion), 1 cat had dry abdominal form and 4 cats had ocular FIP. At diagnosis, the median serum total protein was 8.3 g/dL (IQR 7.5‐9.8), median albumin 2.3 g/dL (IQR 2.2‐2.5), median globulins 6.0 g/dL (IQR 5.0‐7.4) and median albumin: globulin (A:G) ratio was 0.4 (IQR 0.3‐05). At the 12 weeks recheck, the median serum total protein was 7.1 g/dL (IQR 6.9‐7.4), median albumin 3.4 g/dL (IQR 3.2‐3.7), median globulins 3.7/dL (IQR 3.4‐4.1), and median A:G ratio was 0.9 (IQR 0.8‐1.0). All cats are clinically doing well. The only reported side effect was hypersalivation in one cat treated with EIDD‐2801 and placebo, all the other cats tolerated the therapy well.

**Conclusions and Clinical Importance:** This preliminary data suggest that these medications are useful in successful treatment of FIP without severe side effects.

## ABSTRACT ID02: Nucleoside analogue EIDD‐1931 in the treatment of naturally occurring feline infectious peritonitis

### **Alex Kennedy** ^1^; Joanna White^2^, BVSc, PhD, MVS (Epi), DAVCIM

#### ^1^Small Animal Specialist Hospital, NSW, Australia; ^2^Internal Medicine Specialist, Small Animal Specialist Hospital, NSW, Australia

**Background:** Current antiviral therapy for feline infectious peritonitis (FIP) has limited availability and can be cost prohibitive. The nucleoside analogue EIDD‐1931 is an effective inhibitor against feline infectious peritonitis virus (FIPV) serotype I and II in vitro.

**Hypothesis:** EIDD‐1931 is an effective treatment option for cats with naturally occurring FIP.

**Animals:** Nine client‐owned cats diagnosed with effusive or noneffusive FIP including neurological involvement.

**Methods:** Prospective clinical trial. Cats were administered EIDD‐1931 orally twice daily for 12 weeks. A complete response was defined as resolution of all abnormalities associated with FIP. Clinical variables, hematology, biochemistry, and imaging findings were monitored during treatment and after discontinuing treatment.

**Results:** Six cats with effusive FIP and three cats with noneffusive FIP (median age 1.0 years, range 0.5‐7.9) were treated with EIDD‐1931 (median dose 18.75 mg/kg PO q 12, range 12.0‐23.8) for 12 weeks in eight cats and 14 weeks in one cat. All cats showed a complete response to treatment. Adverse effects included transient neutropenia (three cats), elevated alanine transaminase (transient in three, persistent in one cat), broken whiskers (one cat) and suspected treatment induced relative hyporexia (six cats). Adverse effects were not dose dependent. Follow up was available for 91‐231 days post treatment discontinuation. Relapse in one cat, 70 days after EIDD‐1931 was discontinued, responded to repeat treatment (25 mg/kg twice daily).

**Conclusions and Clinical Importance:** EIDD‐1931 administered at 15‐20 mg/kg PO q 12 for 12 weeks is a feasible treatment option for naturally occurring FIP. Adverse effects may be more common than treatment with GS‐441524.

## ABSTRACT ID03: Treatment response, course of improvement, and follow‐up information with molnupiravir treatment for feline infectious peritonitis

### **Shino Yoshida** ^1^; Mei Sugawara‐Suda^2^, DVM, PhD; Kazuyoshi Sasaoka^2^, DVM, PhD; Noboru Sasaki^2^, DVM, PhD; Nozomu Yokoyama^3^, DVM, PhD; Kensuke Nakamura^4^, DVM, PhD, DAiCVIM (Internal Medicine); Keitaro Morishita^4^, DVM, PhD, DAiCVIM (Internal Medicine); Sangho Kim^3^, DVM, PhD, DJCVS (Small Animal Surgery); Takafumi Sunaga^3^, DVM, PhD; Mitsuyoshi Takiguchi^5^, DVM, PhD, DAiCVIM (Internal Medicine)

#### ^1^Washington State University, Pullman, WA, USA; ^2^Assistant Professor, Veterinary Teaching Hospital, Hokkaido University, Sapporo, Hokkaido, Japan; ^3^Assistant Professor, Department of Veterinary Clinical Sciences, Hokkaido University, Sapporo, Hokkaido, Japan; ^4^Associate Professor, Department of Veterinary Clinical Sciences, Hokkaido University, Sapporo, Hokkaido, Japan; ^5^Professor, Department of Veterinary Clinical Sciences, Hokkaido University, Sapporo, Hokkaido, Japan

**Background:** Limited information is available regarding the treatment course with molnupiravir treatment for feline infectious peritonitis (FIP).

**Hypothesis/Objectives:** To clarify the clinical course and relapse rate of molnupiravir treatment for FIP.

**Animals:** Cats diagnosed with FIP and treated with molnupiravir.

**Methods:** Retrospective study at Hokkaido University Veterinary Teaching Hospital between January 2023 and January 2024.

**Results:** Eleven cats were eligible for inclusion. Six cats had pleural or peritoneal effusion. Two cats showed neurological abnormalities at the diagnosis. The median initial dosage of molnupiravir was 13.0 mg/kg q 12 h (range, 10.0‐15.0 mg/kg q 12 h). While one cat died in 11 days, ten cats completed an 84‐day course of treatment. The dosage was increased in two cats, and the median final dosage of molnupiravir was 13.6 mg/kg q 12 h (range, 10.0‐17.2 mg/kg q 12 h). In cats that did not require an increase in dosage, improvement of hyporexia, lethargy, pyrexia, high serum amyloid A (SAA), effusion, and lymph node enlargement were seen within 15 days after treatment initiation. Also, the owners noticed an improvement in clinical signs within a week (2‐6 days) in five cases. Albumin: globulin ratio remained <0.6 in four cats. After 84 days of treatment, follow‐up periods exceeded 84 days (median 113 days; range, 85‐271 days), and no relapse was reported.

**Conclusions and Clinical Importance:** In the treatment of FIP with molnupiravir, clinical signs, pyrexia, high SAA, effusion, and lymph node swelling were improved within 15 days. No relapse was observed during the study.

**Figure:**
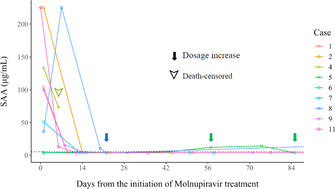
Change in serum amyloid A (SAA) during an 84‐day course of treatment. After the initiation of treatment, SAA normalized within 15 days for responsive cases without dosage increase. The dotted line shows the reference range (<5.5 mcg/mL; Fiju DRI‐CHEM IMMUNO AU10V; Fujifilm).

## ABSTRACT ID04: *Klebsiella pneumoniae* in pets: carbapenem resistance surveillance and invasive disease in veterinary care

### **Constanca Pomba** ^1^; Laura Fernandes^2,4^, DVM, Msc; Joana Da Silva^2^, Msc; Juliana Menezes^2^, Msc; Cátia Marques^3,4,5^, PhD; Cátia Caneiras^5^

#### ^1^University of Lisbon, Lisbon, Portugal; ^2^PhD student, Antibiotic Resistance Lab, Centre for Interdisciplinary Research in Animal Health (CIISA), Faculty of Veterinary Medicine, University of Lisbon, Lisbon, Portugal; Associate Laboratory for Animal and Veterinary Sciences (AL4AnimalS), Lisbon, Portugal; ^3^Researcher, Antibiotic Resistance Lab, Centre for Interdisciplinary Research in Animal Health (CIISA), Faculty of Veterinary Medicine, University of Lisbon, Lisbon, Portugal; Associate Laboratory for Animal and Veterinary Sciences (AL4AnimalS), Lisbon, Portugal; ^4^Molecular Veterinary Diagnostic Laboratory, GeneVet, Carnaxide, Portugal; ^5^Faculty of Veterinary Medicine, Lusófona University, Lisbon, Portugal; ^5^PhD Pharm, Assistant Professor, Microbiology, Laboratory of Microbiology Research in Environmental Health (EnviHealthMicro Lab), Institute of Environmental Health (ISAMB), Faculty of Medicine, University of Lisbon, Lisbon, Portugal; Institute of Preventive Medicine and Public Health, Faculty of Medicine, University of Lisbon, Lisbon, Portugal

**Background:** The emergence of *Klebsiella pneumoniae* carrying carbapenemase‐genes with associated hypervirulence, causing invasive infections, is a concern in human health; its impact in veterinary medicine however remains unknown.

**Objective:** To assess the presence of carbapenemase‐producing *K. pneumoniae* causing invasive infections in companion animals (CA).

**Animals:** The study was performed on a collection of 4293 Enterobacterales isolates obtained during 2020 to mid‐2023 from samples submitted for microbiological analysis originating from CA with suspected infection.

**Methods:** Beta‐lactam resistance was screened using CRE (carbapenemase‐resistant Enterobacterales) Agar selective media (CRS), and confirmation of species as *K. pneumoniae* was performed by PCR. Positive isolates were further evaluated by disc diffusion for resistance to other beta‐lactams; a string test was performed to screen for hypervirulence. Carriage of carbapenemases was confirmed by PCR and sequencing.

**Results:** A total of 107 CRS‐positive isolates were PCR‐confirmed as *K. pneumoniae* (2.5% of all isolates). CRS *K. pneumoniae* isolates originated from UTIs (52.3%; n = 56/107), skin and soft tissue infections (39.3%; n = 42/107), and respiratory infections (8.4%; n = 9/107). Six isolates were carbapenemase gene carriers, identified as two carrying the blaKPC‐3 gene, three blaOXA‐181, and one blaOXA‐48 which was previously undetected in CA in Portugal. Three CRS isolates were string test positive but lacked detectable carbapenemase genes.

**Conclusions:** Low prevalence and absence of inclusion and/or harmonization of routine screening methods for carbapenem resistance in veterinary clinical microbiology laboratories may be hindering epidemiological surveillance of this relevant virulent and resistant phenotype of this pathogen with important animal and public health impact.

## ABSTRACT ID05: A preliminary insight on the molecular epidemiology of feline immunodeficiency virus in Portugal, Europe

### **Constanca Pomba** ^1^; Cátia Marques^2^, DVM, PhD; Sílvia Honrado^3^, MSc; Ana Sofia Bolas^4^, PhD; Inês Leal^5^, Msc; Ana Duarte^6^, DVM, PhD

#### ^1^University of Lisbon, Lisbon, Portugal; ^2^Assistant Professor, Clinics, Associate Laboratory for Animal and Veterinary Sciences (AL4AnimalS), Lisbon, Portugal; Faculdade de Medicina Veterinária, Universidade Lusófona, Avenida do Campo Grande, Lisboa, Portugal; ^3^Centre for Interdisciplinary Research in Animal Health, Faculdade de Medicina Veterinária, Universidade de Lisboa, Avenida da Universidade Técnica, Lisboa, Portugal; ^4^Molecular Veterinary Diagnostic Laboratory, GeneVet, Carnaxide, Portugal; ^5^Centre for Interdisciplinary Research in Animal Health, Faculdade de Medicina Veterinária, Universidade de Lisboa, Avenida da Universidade Técnica, Lisboa, Portugal; ^6^Researcher, Virology, Centre for Interdisciplinary Research in Animal Health, Faculdade de Medicina Veterinária, Universidade de Lisboa, Avenida da Universidade Técnica, Lisboa, Portugal; Associate Laboratory for Animal and Veterinary Sciences (AL4AnimalS), Lisbon, Portugal

**Background:** The feline immunodeficiency virus (FIV) causes a lifelong infection in domestic cats being endemic worldwide. FIV has a relatively high evolutionary rate which interferes with vaccination and molecular diagnostic tools.

**Hypothesis/Objectives:** This study aimed to characterize and evaluate the genetic diversity of FIV in mainland Portugal and Azores, Europe.

**Animals:** This study included 54 urban and rural stray cats from mainland Portugal, and 8 from São Miguel Island, Azores, that tested positive for FIV.

**Methods:** Samples were obtained between 2009 and 2022. A total of sixty‐two FIV‐positive blood samples were submitted to PCR and Sanger sequencing of the V3‐V5 region (previously submitted to Genbank). A phylogenetic analysis was conducted using Mega version 7.

**Results:** The resulting phylogenetic tree displayed an increasing genetic diversity of FIV subtype B, mainly due to the inclusion of FIV sequences from the Azores islands. A new phylogenetic cluster including new continental Portuguese sequences was also identified, and named subtype F.

**Conclusions and Clinical Importance:** To our knowledge, this is the first study considering FIV isolates from the Azores Islands besides continental Portugal confirming regional genetic diversity. Updated genetic data is key to determine which FIV subtypes are predominant and for the development of accurate molecular diagnostic methods for the screening and monitoring of cats infected by FIV, particularly in their first 6 months of life and on very rare occasions of later stages of FIV infection where antibodies levels are too low to be detected.

## ABSTRACT ID06: Coexpression of complement‐regulating factors and FCoV‐antigen in feline infectious peritonitis

### **Anne Hoenl** ^1^; Sandra Felten^2^, DECVIM, DrMedVetHabil; Katharina Erber^1^, DrMedVet; Michele Bergmann^1^, DrMedVetHabil; Katrin Hartmann^1^, DrMedVetHabil, DECVIM; Kaspar Matiasek^1^, DrMedVetHabil, AM‐ECVN

#### ^1^LMU Munich, Munich, Germany; ^2^VetSuisse Zurich, Zurich, Switzerland

**Background:** Feline infectious peritonitis (FIP) caused by feline coronavirus (FCoV) infection resembles a multisystemic hyperinflammatory syndrome that involves the complement cascade. Complement activation however appears to spare FCoV‐infected cells and therefore does not effectively contribute to virus clearance.

**Hypotheses/Objectives:** To evaluate whether FCoV‐infected cells are protected by autocrine/paracrine expression of complement‐regulating factors.

**Animals/Tissues:** The study enrolled postmortem tissues of 31 cats with PCR/IHC‐confirmed FIP.

**Methods:** Sections of formalin‐fixed, paraffin‐embedded tissue from FIP lesions underwent immunohistochemical double‐staining for (1) FCoV antigen plus (2a) complement‐regulating factors (CRF) CD46 and CD59 as well as (2b) complement factors C1q and C9. Marker expression was scored semiquantitatively and their spatial relationship was statistically evaluated.

**Results:** All 31 cats presented with FCoV‐positive foci, of which a majority co‐expressed both CRFs CD46 and CD59 (*P* < .05) albeit to a variable extent. C1q and C9 were not seen in or next to FCoV‐positive cells. Moreover, the higher the CRF expression, the larger was the distance to C1q‐ and C9‐positive lesions (*P* < .05).

**Conclusions and Clinical Relevance:** Complement‐regulating factors indeed seem to protect FCoV‐infected cells from complement‐mediated cytolysis and virus clearance. Likewise, CRFs suppress activation of C1 complex that initiates early cleavage cascades of the classical pathway as much as they suppress C9 polymerization and formation of the membrane attack complex. No such suppression is seen in prototypic FIP lesions distant to infected cells, where complement activation contributes to tissue damage.

## ABSTRACT ID07: Fecal microbial transplant for parvovirus in the outpatient setting interim analysis: a randomized controlled trial

### **Amanda R. Gimenez** ^1^; Meghan Hoel^2^; Erin Lashnits^3^, MS, DVM, PhD, DACVIM

#### ^1^University of WisconsinCMadison School of Veterinary Medicine, Madison, WI, USA; ^2^Graduate Research Assistant, University of Wisconsin‐Madison School of Veterinary Medicine, Madison, WI, USA; ^3^Principal Investigator, Internal Medicine, University of Wisconsin‐Madison School of Veterinary Medicine, Madison, WI, USA

**Background:** Canine parvoviral enteritis (CPVE) can have high morbidity and mortality in dogs without access to intensive inpatient care. Fecal microbial transplant (FMT) shows promise in inpatient settings, enema‐delivered FMT can be logistically difficult in outpatient settings.

**Hypothesis/Objectives:** To evaluate commercially available oral fecal microbial transplant capsules (AnimalBiome DoggyBiome Gut Restore Supplement) as a practical, low‐cost treatment of CPVE. The hypothesis was that oral FMT would improve survival compared to placebo in dogs with CPVE otherwise treated with standardized outpatient care.

**Animals:** 57 dogs diagnosed with CPVE at Austin Pets Alive (Austin, TX) from 9/17/22 to 8/5/2023:32 control, 25 FMT‐treated.

**Methods:** Randomized controlled trial. Staff administering treatments were blinded. The treatment group received oral FMT capsules and control received placebo for 1, 2, or 4 days. The primary outcome was survival to discharge, with secondary outcomes: time to resolution of symptoms.

**Results:** At the time of planned interim analysis, survival was 97% in controls (31/32) compared to 80% for FMT‐treated dogs (20/25, *P* = .1041). There were no statistically significant differences in proportion of dogs with vomiting (30% controls vs. 50% FMT‐treated, *P* = .1816) or time to resolution of diarrhea (median 4.5 days controls, 8 days FMT‐treated, *P* = .1962) or inappetence (median 4 days controls, 6 days, *P* = .2612).

**CONCLUSION:** FMT‐treated dogs had overall lower survival, though this difference was not statistically significant. Continued enrollment is needed to determine if oral FMT could in fact be harmful in this setting.

## ABSTRACT ID08: Molecular investigation of tick‐borne pathogens from 6141 dogs and 682 cats in South Korea

### **Jimin Park** ^1^; Doosung Cheon^2^; Jinsun Kim^2^; Soyeon Park^1^; Yein Oh^3^, DVM, PhD, DAiCVIM

#### ^1^Kyungpook National University, Daegu, South Korea; ^2^Postbio, Sunhwagung‐ro, Namyangju‐si, Gyeonggi‐do; ^3^Assistant Professor, College of Veterinary Medicine, Kyungpook National University, Daegu, South Korea

**Background:** Tick‐borne infections are globally increasing due to the effects of climate change, trend mirrored in South Korea.

**Hypothesis/Objectives:** This retrospective study aims to determine the detection rate of 9 major tick‐borne infectious disease pathogens using real‐time PCR test in dogs and cats in South Korea.

**Animals:** 6141 blood specimens from dogs and 682 blood specimens from cats with anemia or suspected tick‐borne infections were analyzed.

**Methods:** Real‐time PCR data submitted to a veterinary diagnostic laboratory for tick‐borne infectious diseases testing in South Korean dogs and cats from January 2022 to September 2023 were extracted.

**Results:** In dogs, the most common pathogen was *Babesia gibsoni* (17.99%, n = 1105). Hemotropic *Mycoplasma* (0.85%, n = 52), SFTS virus (0.64%, n = 39), *Anaplasma* spp. (0.59%, n = 36), *Hepatozoon* spp. (0.57%, n = 35), and *Rickettsia* spp. (0.07%, n = 4) were also detected. *Leptospira* spp., *Bartonella* spp., and *Borrelia burgdorferi* were not found. In cats, Hemotropic *Mycoplasma* (1.8%, n = 12) and SFTS virus (0.6%, n = 4) were the most common, while *Anaplasma* spp., *Babesia* spp., and *Hepatozoon* spp. each had one case (0.1%, n = 1). *Leptospira* spp., *Bartonella* spp., *Rickettsia* spp., and *Borrelia burgdorferi* were not detected.

**Conclusion and Clinical Importance:** Proactive and continuous monitoring is essential for providing appropriate diagnosis and treatment in response to evolving infection trends.

## ABSTRACT ID09: Molecular prevalence of upper respiratory tract infection pathogens in dogs and cats in South Korea

### **SoYeon Park** ^1^; Doosung Cheon^2^; Jinsun Kim^2^; Jimin Park^1^, DVM; Yein Oh^3^, DVM, PhD, DAiCVIM

#### ^1^Kyungpook National University, Daegu, South Korea; ^2^Postbio, Sunhwagung‐ro, Namyangju‐si, Gyeonggi‐do; ^3^Assistant Professor, College of Veterinary Medicine, Kyungpook National University, Daegu, South Korea

**Background:** The prevalence of respiratory pathogens may differ based on geographical regions, leading to potential differences in empirical treatment approaches.

**Hypothesis/Objectives:** To assess the prevalence of infectious agents associated with upper respiratory tract infection (URTI) in dogs and cats in South Korea.

**Animals:** 1370 respiratory specimens from dogs and 4481 respiratory specimens from cats were analyzed.

**Methods:** Retrospective assessment of real‐time PCR data of specimens submitted to a veterinary diagnostic laboratory for testing respiratory diseases in dogs and cats in the South Korea from January 2022 to September 2023. Molecular investigation was performed on 26 infectious agents in dogs and cats, respectively.

**Results:** In dogs, a total of 21 infectious agents were detected. The most common pathogen was *Pseudomonas aeruginosa* (26.1%, n = 358), followed by *Mycoplasma* spp. (24.7%, n = 338), *Bordetella bronchiseptica* (17.9%, n = 245), *Streptococcus* spp. (16.9%, n = 231), and *Mycoplasma cynos* (7.7%, n = 105). In cats, a total of 18 infectious agents were detected. The predominant pathogen was *Pseudomonas aeruginosa* (34.7%, n = 1556), followed by *Mycoplasma* spp. (26.5%, n = 1188), *Mycoplasma felis* (22.2%, n = 997), feline calicivirus (22.1%, n = 990), and feline herpes virus (13.7%, n = 616).

**Conclusion and Clinical Importance:** This investigation provides valuable insights into the prevalence of respiratory pathogens in dogs and cats. The observed variations underscore the importance of tailoring empirical therapeutic approaches based on the specific pathogens identified, offering valuable information for clinicians in the region.

## ABSTRACT ID10: Assessing antimicrobial resistance in companion animals at referral hospital: the impact of antimicrobial stewardship strategies

### **Tomoki Motegi** ^1^; Dai Nagakubo^2^, PhD, DVM, MS; Shingo Maeda^3^, PhD, DVM, MS; Tomohiro Yonezawa^3^, PhD, DVM, MS; Ryouhei Nishimura^4^, PhD, DVM, MS; Yasuyuki Momoi^5^, PhD, DVM, MS

#### ^1^Chobanian & Avedisian School of Medicine, Boston University, Boston, MA, USA; ^2^Project Assistant Professor, Veterinary Medical Center, The University of Tokyo, Tokyo, Japan; ^3^Associate Professor, Clinical Pathology, The University of Tokyo, Tokyo, Japan; ^4^Professor, Surgery, The University of Tokyo, Tokyo, Japan; ^5^Professor, Clinical Pathology, The University of Tokyo, Tokyo, Japan

**Background:** Antimicrobial stewardship (AS) is crucial for reducing antimicrobial resistance. However, the factors of inappropriate antimicrobial use are complex, and effective interventions have to be found in each hospital.

**Hypothesis/Objectives:** We hypothesized that a key issue was a lack of awareness and understanding of the current circumstances of antimicrobial resistance. Our objective was to evaluate whether in‐hospital AS, designed to elucidate current resistance trends and inform appropriate countermeasures, could effectively reduce antimicrobial resistance.

**Animals:** We conducted a comprehensive analysis on 1635 strains subjected to antimicrobial sensitivity testing, along with 28 843 antibiotic prescriptions. This assessment included evaluating the existing antimicrobial resistance patterns from the sensitivity tests and analyzing the changes in antibiotic use in our AS based on prescriptions by year.

**Methods:** Our approach involved creating an in‐hospital antibiogram to track resistance spread, using Gram stains to estimate bacterial species, and recommending effective antibiotics. We specifically addressed the suspected inappropriate use of antibiotics, such as carbapenems and fluoroquinolones, suggesting alternatives. Furthermore, lectures on proper antibiotic use in clinical settings were delivered to interns from 2019.

**Results:** The interventions led to a 50% reduction in antibiotic prescriptions, with carbapenems usage dropping by 89% and reductions of injectable and oral enrofloxacin by 80% and 79%, respectively. Remarkably, the antibiogram showed a significant presence of ESBL‐producing *E. coli*, which decreased from 53% to 22% incidence.

**Conclusions and Clinical Importance:** Our findings underscore that AS programs, emphasizing in‐hospital education on appropriate antibiotic use and antibiotic selection based on antibiogram data, can significantly curb antimicrobial resistance.

## ABSTRACT ID11: Period‐prevalence and distribution of *Babesia* species exposure in thrombocytopenic dogs in the upper midwest

### **Lisa G. Kim** ^1^; Amanda Brooks^2^, CVT; Amy Elbe^3^, CVT, LAT; Shelby Ilkenhans^3^; Jessica Pritchard^2^, VMD, MS, DACVIM (SAIM); Nina Zitzer^4^, DVM, PhD, DACVP; Erin Lashnits^2^, MS, DVM, PhD, DACVIM (SAIM)

#### ^1^University of Wisconsin‐Madison, Madison, WI, USA; ^2^Department of Medical Sciences, School of Veterinary Medicine, University of Wisconsin‐Madison, Madison, WI, USA; ^3^Clinical Studies Department, School of Veterinary Medicine, University of Wisconsin‐Madison, Madison, WI, USA; ^4^Department of Pathobiological Sciences, School of Veterinary Medicine, University of Wisconsin‐Madison, Madison, WI, USA

**Background:** Babesiosis in dogs is thought to be rare in the upper Midwest, so infections in this geographic region may go undetected or misdiagnosed. Because of the potential for *Babesia* species geographic range to expand with climate and land‐use change and globalization in pet travel, it is important to provide updated estimates of exposure in dogs.

**Hypothesis/Objectives:** To estimate the period‐prevalence of *Babesia* exposure in thrombocytopenic dogs, and in future to determine if exposure in this non‐endemic area is associated with thrombocytopenia.

**Animals:** Serum and EDTA whole blood samples from 418 dogs presented to University of Wisconsin Veterinary Care (UWVC) with (n = 209) and without (n = 209) thrombocytopenia over a one‐year period (March 2021‐March 2022).

**Methods:** Descriptive cross‐sectional study. Broad and species‐specific PCR of whole blood, and indirect fluorescent antibody (IFA) assay and enzyme‐linked immunological assay (ELISA) of serum were performed to detect *Babesia* infection and exposure.

**Results:** Based on preliminary results, no thrombocytopenic dogs were *Babesia* spp. PCR positive from whole blood. Nine thrombocytopenic dogs (4.3%) were seroreactive on IFA and/or ELISA.

**Conclusions and Clinical Importance:** While approximately 4% of thrombocytopenic dogs in the upper Midwest had serologic evidence of *Babesia* exposure, none had PCR evidence of active infection. Future work will evaluate the seroprevalence of *Babesia* exposure in non‐thrombocytopenic dogs from the same geographic region to determine if *Babesia* exposure is associated with thrombocytopenia and should, therefore, be considered among differentials even in dogs with unknown travel history in this non‐endemic area.

## ABSTRACT ID12: Successful in‐hospital treatment of FIP with FDA‐approved Remdesivir (Veklury) in 5 cats

### **Erin Lashnits** ^1^; Amy Elbe^2^; Anita Fothergill^2^; Robert Kirchdoerfer^2^; Annika Quint^2^; Phoenix Shepherd^2^

#### ^1^School of Veterinary Medicine, University of Wisconsin‐Madison, Madison, WI, USA; ^2^University of Wisconsin‐Madison, Madison, WI, USA

**Background:** Feline infectious peritonitis (FIP) is a fatal disease of cats caused by feline coronavirus; until recent years there was no effective treatment. However, the recently developed antiviral drug remdesivir can reverse the clinical progression of FIP and lead to long‐term remission. Since remdesivir was approved by the FDA in April 2022 it is available legally to veterinarians for off‐label use in cats.

**Objectives:** To describe treatment protocols and outcomes of 5 cats with FIP treated in‐hospital with an initial course of injectable remdesivir (Veklury).

**Animals:** 5 cats.

**Methods:** Descriptive case series.

**Results:** 5 cats were treated for FIP with remdesivir between 2/2023‐12/2023. Remdesivir was administered at doses between 10 and 22 mg/kg via intravenous continuous rate infusion over 1‐8 hours, repeated every 12‐24 hours for 1‐4 days. No adverse effects were noted during administration. Following initial remdesivir treatment, all five cats were reported by owners to have received additional at‐home treatment with unlicensed crowd‐soured supplements marketed as GS‐441524. At the time of writing, 3/5 cats have completed treatment (treatment duration 84‐114 days) and remain in remission with complete response off antiviral medications; 2/5 cats remain in treatment at‐home (treatment duration at time of writing 37 and 42 days) both with partial response.

**Conclusions and Clinical Importance:** Initial treatment of FIP with IV remdesivir (Veklury) was feasible and effective in these cats. Future research should investigate whether initial IV remdesivir treatment improves survival or hastens recovery in critically ill cats with FIP compared to current practice using unlicensed GS‐like supplements alone.

## ABSTRACT IM01: Large‐scale retrospective study of vaccine‐associated adverse events in cats

### **Bianca A. Lara** ^1^; Lynn Guptill^1^; George Moore^1^; JoAnn Morrison^2^; Mike Yang^3^

#### ^1^Purdue University, West Lafayette, IN, USA; ^2^Mars Veterinary Health; ^3^Banfield Pet Hospital

**Background:** This study reassesses the rate of vaccine‐associated adverse events (VAAE) and risk factors for VAAE in pet cats. The results may help inform veterinarians in the application of feline vaccination guidelines and help decrease risk of disease spread and outbreaks.

**Objective:** To determine the rates of VAAE in cats post vaccination. To determine possible risk factors associated with VAAE. We hypothesize that there will be a low rate of VAAE.

**Animals:** Total of 1 543 413 vaccinated cats.

**Methods:** Retrospective cohort study of medical records obtained from Banfield Pet Hospital in the United States. Cats included those vaccinated with FVRCP, FeLV, and/or rabies vaccine from 2015 to 2022. Medical records were searched for VAAEs using selected diagnosis codes (i.e., vaccine reaction, allergic reaction, dyspnea, vomiting, etc.). Information was gathered about VAAE within two time windows: 0‐3 and 4‐15 days post vaccination.

**Results:** 28263 (0.8%) of cats vaccinated had a reported adverse reaction. VAAEs were most commonly reported within 3 days of vaccination. Increasing the number of vaccines administered at the vaccination visit was not associated with increased VAAE risk. Most VAAEs were associated with the rabies vaccine, and vaccines administered with rabies vaccine also had higher percentage of VAAEs than other combinations or sole vaccines.

**Conclusions:** Although overall VAAE rates were low, veterinarians should consider the finding that rabies vaccine may be more commonly associated with VAAE when creating vaccination strategies.

## ABSTRACT IM02: In vivo effects of methadone administration on immune function in healthy dogs

### **Lauren Chittick** ^1^; Jared Jaffey^2^, DVM, MS, DACVIM (SAIM); Charles Veltri^3^, PhD; Charlotte Bolch^4^, PhD; Tian Zhou^5^, MS; Imani Carswell^6^, DVM; Heather Perkins^7^, DVM; Anderson da cunha^8^, DVM, MS, DACVAA

#### ^1^Midwestern University, Glendale, AZ, USA; ^2^Assistant Professor, Specialty Medicine, College of Veterinary Medicine, Midwestern University, Glendale, AZ, USA; ^3^Associate Professor, College of Veterinary Medicine, Midwestern University, Glendale, AZ, USA; ^4^Associate Director, Office of Research and Sponsored Programs, College of Veterinary Medicine, Midwestern University, Glendale, AZ, USA; ^5^Statistical Analyst, Office of Research and Sponsored Programs, College of Veterinary Medicine, Midwestern University, Glendale, AZ, USA; ^6^Veterinarian, Specialty Medicine, College of Veterinary Medicine, Midwestern University, Glendale, AZ, USA; ^7^Intern, Specialty Medicine, College of Veterinary Medicine, Midwestern University, Glendale, AZ, USA; ^8^Hospital Director, Specialty Medicine, College of Veterinary Medicine, Midwestern University, Glendale, AZ, USA

**Background:** Methadone is a commonly used opioid in dogs, though its immunological effects are poorly characterized.

**Objectives:** Determine whether methadone administration affects leukocyte phagocytic function, oxidative burst, and cytokine production and if immune function is associated with plasma methadone concentrations.

**Animals:** Ten owned healthy dogs.

**Methods:** Prospective randomized, placebo‐controlled, open‐label, crossover study. Dogs were randomized to receive either methadone (0.3 mg/kg IV) or placebo (0.9% NaCl IV) once every 6 hours for 24 hours. Dogs were crossed over to the alternative treatment following a 7‐day washout period. Blood was collected at baseline (ie, before treatment administration; (T0) and then 10 minutes (T1), 6 hours (T2), and 24 hours (T3) after treatment administration. Immune function tests and plasma methadone concentrations were measured at all time points (T0‐T3). Plasma methadone concentrations were measured with liquid chromatography quadrupole time‐of‐flight mass spectrometry. Whole blood cultures were performed with exposure to PBS, LPS, and LTA. Canine specific multiplex assay was used to measure TNF‐α, IL‐6, and IL‐10 concentrations in supernatant. Granulocytic and monocytic (GM) phagocytosis and oxidative burst were evaluated via flow cytometry with previously validated assays.

**Results:** There was a moderate inverse association between the percentage of GM undergoing oxidative burst and plasma methadone concentrations (r = −0.52; 95% CI: −0.78 to −0.11; *P* = .02). No time‐dependent or between treatment differences in immune function results were identified after adjustments for multiple comparisons (*P* > 0.05).

**Conclusions and Clinical Importance:** Methadone may have immunologic effects in dogs but requires additional investigation.

## ABSTRACT IM03: Effect of cyclosporine on activated T‐cell interleukin‐2 expression in canine hepatic tissue

### **Yi‐Jen Chang** ^1^; Robert Wills^2^, DVM, PhD, DACVPM; Lakshmi Narayanan^3^, PhD; Todd Archer^4^, DVM, MS, DACVIM; Barbara Kaplan^5^, PhD; Gregory Pharr^5^, MS, PhD; Matthew Ross^2^, PhD; Andrew Mackin^6^, BVMS, MVS, DVS, DACVIM

#### ^1^Mississippi State University, Mississippi State, MS, USA; ^2^Professor, College of Veterinary Medicine, Mississippi State University, Mississippi State, MS, USA; ^3^Licensing Analyst, University of Virginia, Charlottesville, VA, USA; ^4^Doctor, Bluff City Veterinary Specialists; ^5^Associate Professor, College of Veterinary Medicine, Mississippi State University, Mississippi State, MS, USA; ^6^Professor, Head of Department, College of Veterinary Medicine, Mississippi State University, Mississippi State, MS, USA

**Background:** Cyclosporine is an immunosuppressive agent widely used in dogs. Pharmacodynamic effects on canine hepatic tissue have not been evaluated.

**Hypothesis/Objectives:** Our objective was to evaluate activated interleukin‐2 (IL‐2) gene expression in canine hepatic tissue after incubation with cyclosporine. Our hypothesis was that IL‐2 expression would be inhibited by cyclosporine in a concentration‐dependent fashion.

**Animals:** Normal hepatic tissue harvested from four canine cadavers.

**Methods:** Hepatic tissues were harvested, and then homogenized and digested. Lymphocytes were isolated and incubated in 0, 10, 100, 500, and 1000 ng/ml of cyclosporine, in duplicate, for 1 hour. Subsequently, lymphocytes were activated with phorbol‐12‐myristate‐13‐acetate and ionomycin, and incubated for 5 hours. After RNA extraction, IL‐2 gene expression was measured by quantitative reverse transcription PCR, and cycle threshold (Ct) values were recorded. One‐way ANOVA and polynomial analysis were used for statistical analysis.

**Results:** Ct values for IL‐2 expression increased significantly as cyclosporine concentration increased from 10 (*P* = .0019) to 100 ng/ml (*P* = .00167), with no significant differences between concentrations of 0 and 10 ng/ml (*P* = .9999), 100 and 500 ng/ml (*P* = 0.1670), and 500 and 1000 ng/ml (*P* = .9618). A positive correlation was observed between IL‐2 expression Ct value and cyclosporine concentration (*P* < .01).

**Conclusions and Clinical Importance:** Cyclosporine significantly inhibited IL‐2 gene expression in canine ex‐vivo hepatic tissue in a concentration‐dependent fashion. Further in vivo studies are necessary to determine the clinical significance of these findings.

**Figure:**
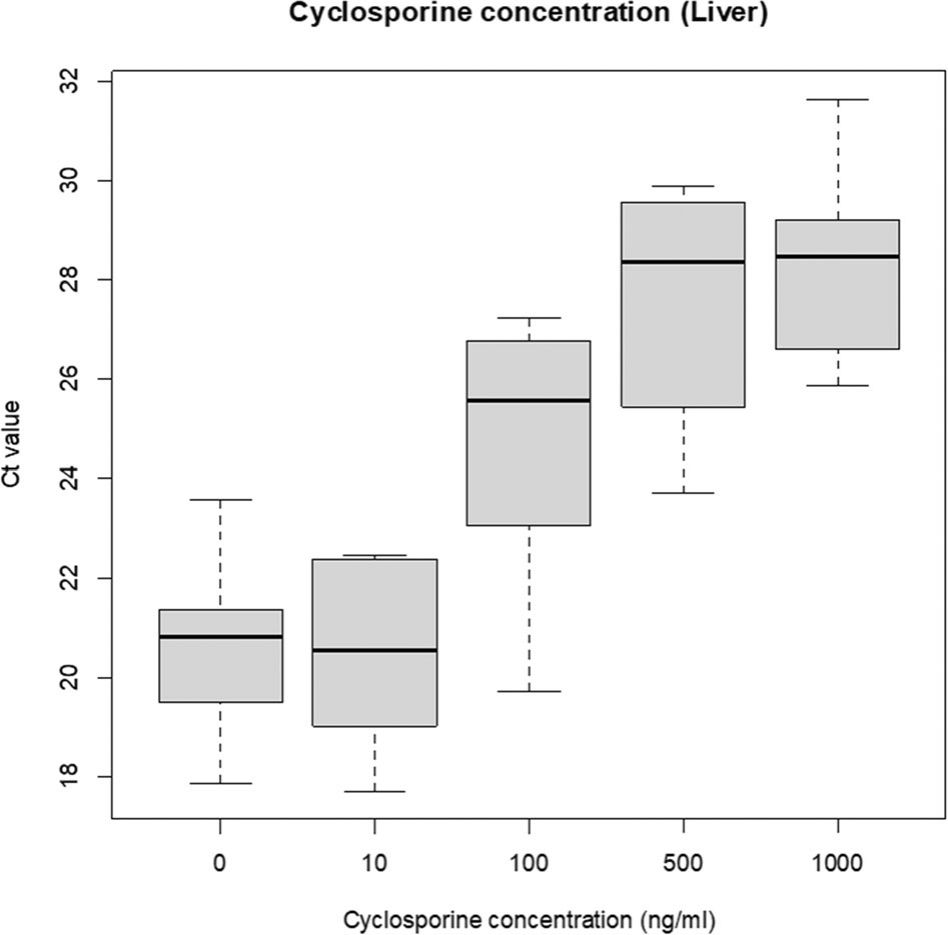
The box and whiskers plot illustrates the cycle threshold (Ct) value of interleukin‐2 gene expression of hepatic lymphocytes after activation with PMA and ionomycin and incubation with 0, 10, 100, 500, and 1000 ng/ml cyclosporine. Bold middle lines in the boxes represent medians, and the top and bottom line of the boxes show the first (25%) and third quartiles (75%) for each group. The whiskers show the range for each group. Note: An **increase** in Ct values corresponds to a **decrease** in gene expression.

## ABSTRACT IM04: Canine parainfluenza one year duration of immunity of a combination BbPi oral vaccine following challenge

### **Rhonda L. LaFleur**; Haley Classe; Sarah Wiechert

#### Merck Animal Health, Rahway, NJ, USA

**Background:** Canine infectious respiratory disease complex is a highly contagious disease caused by multiple bacterial and viral pathogens. A combination oral vaccine containing an attenuated strain of *Bordetella bronchiseptica* and a modified live strain of canine parainfluenza virus (CPI) has recently been introduced to the market to provide broader protection against the disease complex.

**Objective:** The duration of immunity was determined in dogs following challenge with virulent CPI 1 year after vaccination.

**Animals:** Forty‐six, 7‐week‐old antibody profile defined purpose‐bred beagles were used in the study.

**Methods:** Dogs were randomly assigned to 2 treatment groups. On study day 0, dogs were orally vaccinated with either a placebo vaccine (n = 23) or the bivalent test vaccine (n = 23). Neutralizing CPI antibody titers were evaluated throughout the study. One year following vaccination, all dogs were challenged with virulent CPI by the intranasal route. Following challenge, dogs were observed for 14 days for clinical signs of disease, and nasal swabs were collected for 10 days to evaluate duration of shedding.

**Results:** The median duration of CPI viral shedding in the placebo‐vaccinated control group was 6 days, which was significantly longer than the 1 day observed in the vaccinated treatment group (*P* = .001). In addition, the vaccinated treatment group demonstrated significant reduction in viral load.

**Conclusions:** Oral vaccination with a combination oral vaccine containing *Bordetella bronchiseptica* and CPI is effective at significantly reducing CPI viral shedding at least 1 year following vaccination.

## ABSTRACT IM05: Up‐regulation of CCR5 and CCL5 may contribute to chronic low‐grade inflammation in aging dogs

### **Selena K. Tavener** ^1^; Kiran Panickar^2^, PhD

#### ^1^Science & Technology Center, Hill's Pet Nutrition, Topeka, KS, USA; ^2^Director, Life Science Laboratory, Hill's Pet Nutrition, Topeka, KS, USA

**Background:** Chronic age‐related inflammation is a common risk factor for developing inflammatory conditions. Functional immunity declines with age causing immunosenescence. However, aging is also characterized by increased low‐grade innate immune activation contributing to inflamm‐aging.

**Hypothesis:** Increased expression of pro‐inflammatory cytokines and chemokines contribute to inflamm‐aging in older canines.

**Animals:** Healthy canines housed in Hill's animal colony and divided into two groups: young (n = 17, 2‐4 yr) and old (n = 15, 10‐13 yr).

**Methods:** Total circulating RNA was used to assess gene expression using NanoString nCounter platform and analyzed using the nSolver software.

**Results:** When comparing older canines to young dogs, there was significant up‐regulation in CCL5, CCL4, CCR5, IL‐15, TNF, NF‐κB1 (p105), NF‐κB2 (p100), IK**B**Kε, Akt1, JAK3, STAT1 (*P* < .05), and a non‐significant increase in CCL3 and CD40LG.

**Conclusions and Clinical Importance:** CCL4 and CCL3 are known to form dimers or larger aggregates with CCL5 in inflammatory conditions. CCR5 is a receptor for CCL3, CCL4, and CCL5. Il‐15 and CD40 contribute to CCL5 production. The CCL5/CCR5 axis is hypothesized to augment vascular dysfunction in aging. CCR5/CCL5 and TNF‐alpha are known to activate the NF‐kB pathway. CCL5 also activates JAK3 in T‐cells and NK‐cells, and JAK3 subsequently activates multiple pathways including the PI3/Akt and JAK/STAT. Our results indicate importance of the CCL5/CCR5 axis in aging through activation of multiple downstream signaling pathways. Down‐regulation of the CCL5/CCR5 axis through nutrition may be an attractive therapeutic strategy to attenuate immune‐mediated inflammation in aging dogs.

## ABSTRACT IM06: Inhibition of multiple signaling pathways may attenuate mTOR and promote healthy aging in older dogs

### **Selena K. Tavener** ^1^; Kiran Panickar^2^, PhD

#### ^1^Science & Technology Center, Hill's Pet Nutrition, Topeka, KS, USA; ^2^Director, Life Science Laboratory, Hill's Pet Nutrition, Topeka, KS, USA

**Background:** mTOR is composed of mTORC1, a promotor of cell growth and metabolism, and mTORC2, a regulator of cell proliferation and survival. mTOR dysregulation often increases with age and attenuation of mTOR increases lifespan and slows age‐related diseases.

**Hypothesis:** There is increased mTOR activation in leukocytes in aging dogs.

**Animals:** Healthy canines housed in Hill's animal colony and divided into two groups: young (n = 6, 2‐3 yr) and old (n = 6, 10‐13 yr).

**Methods:** Gene expression was assessed from total circulating RNA using Qiagen's PCR Array‐ Dog mTOR Signaling. Data were analyzed using the ΔΔCt method.

**Results:** There was a significant increase in mLST8, RPS6KA1, EIF4EBP2, Akt1, GSK3B, MAPK3, MAPKAP1, a non‐significant increase in PIK3CB and MAPK1, but no significant increase in RAPTOR, RICTOR, or DEPTOR in older canines when compared to young.

**Conclusions and Clinical Importance:** mLST8 plays a key role in phosphorylating mTORC1 effectors including members of S6K and 4EBP families. Attenuating mTORC1‐S6K1 or mTORC1‐4EBP1 signaling is reported to improve muscle health. Activation of mTORC1 can occur via PI3K/Akt/GSK3 and MAP kinase pathways. Both signaling pathways converge on TSC2 and phosphorylate it to subsequently activate mTORC1. mLST8 is also a component of mTORC2 and MAPKAP1 is a subunit of mTORC2. Components of mTOR complexes and signaling in leukocytes were upregulated in aging dogs and we have previously demonstrated increased level of circulating inflammatory cytokines in aging dogs. Lowering the activation of mTORC1 and mTORC2 through nutrition may be beneficial in reducing age‐related inflammatory conditions in canines.

## ABSTRACT IM07: Use of eltrombopag‐based combination treatment for immune‐mediated thrombocytopenia in dogs

### **Jeongmin Lee** ^1^; Jiwwong Her^2^, DVM, MS, DACVECC; Young‐Wook Cho^1^, DVM; Ji‐Hyun Han^1^, DVM; So‐Hee Jeong^1^, DVM; So‐Young Park^1^, DVM, MS; Yeon‐Ju Kim^1^, DVM; Gi‐Ppeum Lee^1^, DVM, MS; Woo‐Seok Yang^1^, DVM; Dae‐Geon Han^1^, DVM; Ji‐Yun Kim^1^, DVM; Sang‐Woon Lee^1^, DVM; Jae‐Hyeon Seo^1^, DVM; Youn‐Seo Jeong^1^, DVM

#### ^1^Korea Animal Medical Center; ^2^College of Veterinary Medicine, Ohio State University, Columbus, OH, USA

**Background:** Eltrombopag, a thrombopoietin receptor agonist, increases platelet counts in humans with immune‐mediated thrombocytopenia (IMT). However, studies on the effectiveness of eltrombopag in veterinary medicine are lacking.

**Hypothesis/Objectives:** It is hypothesized that eltrombopag‐based combination treatment would more effectively correct platelet counts in dogs with primary IMT.

**Animals:** Twenty‐six client‐owned dogs with IMT.

**Methods:** A retrospective observational study was conducted. IMT was diagnosed based on platelet counts.

**Results:** Twenty‐six dogs included in the study, with six in the eltrombopag treatment group and twenty in the control group. There was no significant difference in the time to reach a platelet count ≥40 000/μL between the treatment group (median 4 days, range 3.75‐8) and the control group (median 4.5 days, range 3‐5.75) (*P* = 0.710). Furthermore, there was no significant difference in transfusion requirements between groups (*P* = 0.407). Of the total 26 dogs, 5 (19%) died, with 1/6 in the treatment group and 5/20 in the control group. No significant difference in mortality was observed between the treatment group and the control group (odds ratio 8%, 95% confidence interval [0.7‐89.1], *P* = 0.856).

**Conclusions and Clinical Importance:** Administration of eltrombopag demonstrated no significant difference in dogs with IMT who were concurrently administered prednisone, mycophenolate mofetil, vincristine, and human intravenous immunoglobulins.

## ABSTRACT N01: Effect of a probiotic on seizure frequency in dogs with idiopathic epilepsy receiving antiepileptic drugs

### **Zoe E. Bailey** ^1^; Starr Cameron^1^, MS, BVetMed, DACVIM (Neurology); Jessica Pritchard^1^, VMD, MS, DACVIM (SAIM); Michael Liou^2^; Rey Chi^1^, DVM; Amy Elbe^1^, CVT, LAT

#### ^1^School of Veterinary Medicine, University of Wisconsin−Madison, Madison, WI, USA; ^2^Department of Statistics; University of Wisconsin−Madison

**Background:** In people with epilepsy, the microbiota‐gut‐brain axis has been identified as a target in reducing seizure frequency, and probiotics have been found to decrease seizure frequency and severity. No large‐scale prospective studies have been performed in dogs with idiopathic epilepsy (IE).

**Hypothesis/Objectives:** Evaluate the effect of a probiotic (Visbiome Vet) on seizure frequency in dogs previously diagnosed with IE receiving antiepileptic drugs (AEDs).

**Animals:** Forty‐two client‐owned dogs with IE.

**Methods:** A prospective, placebo‐controlled, masked, crossover clinical trial over a 9 or 12‐month period (2 arms). After an initial 3‐month observational period, each owner was given 3 months of a placebo capsule (Visbiome Vet without the proprietary blend of probiotics) to administer to their dog. Afterwards, each owner was given 3 months of probiotic capsules (Visbiome Vet) to administer to their dog. Owners were masked as to if the capsules were placebo or probiotic. Seizures were logged by the owners.

**Results:** Twenty‐one dogs with IE were included in the final analysis. Mean seizure frequency during the observational period was 8.6 seizures/month (95% CI = 4.9‐15.2), during placebo administration was 10.3 seizures/month (95% CI = 6.2‐17.2), and, in the preliminary analysis, was 6.9 seizures/month (95% CI = 3.5‐13.6) during probiotic administration. When using a *t*‐test to compare seizure frequency during the observational period to the periods of placebo and probiotic administration, there was no significant improvement to seizure frequency (*P* = .75 and *P* = .73, respectively).

**Conclusions and Clinical Importance:** The preliminary results of this study indicate probiotics do not significantly reduce seizure frequency in dogs with IE.

## ABSTRACT N02: Integrated endoscopic mini‐hemilaminectomy and thoracolumbar lateral corpectomy in cadaveric dogs

### **Megan Wolfe** ^1^; Lisa Bartner^2^, DVM, MS, DACVIM (Neurology); Eric Monnet^3^, DVM, PhD DACVS, DECVS, FAHA; Katie Neal^4^, DVM

#### ^1^Colorado State University, Fort Collins, CO, USA; ^2^Associate Professor, Neurology/Neurosurgery, Colorado State University, Fort Collins, CO, USA; ^3^Professor, Small Animal Surgery, Colorado State University, Fort Collins, CO, USA; ^4^Resident, Diagnostic Imaging, Colorado State University, Fort Collins, CO, USA

**Background:** Minimally invasive neurosurgery is a developing field. Thoracolumbar lateral corpectomy (TLLC) is a documented technique for decompression of intervertebral disc herniations in dogs, but information about minimally invasive TLLC in veterinary medicine is lacking.

**Hypothesis/Objectives:** This study evaluated the feasibility of endoscopic TLLC at various intervertebral disc spaces and with variable patient positioning using cadavers. We hypothesized that targeted surgical dimensions could be achieved at all intervertebral disc spaces from T11‐12 through L3‐4, and that angled positioning would change surgical dimensions.

**Animals:** Eight cadaver dogs (15‐30 kg).

**Methods:** Preoperative CT scans were performed for surgical planning. Target corpectomy dimensions were defined as 25% of vertebral body (VB) length, 50% of VB height, and 67% of vertebral canal diameter. Using the EasyGo! II system, endoscopic mini‐hemilaminectomy and corpectomy were performed from T11‐12 through L3‐4. Corpectomy dimensions were measured as a percentage of total vertebral dimensions on postoperative CT.

**Results:** 36 surgeries were completed. Mean ± SD corpectomy dimensions were 24.17 ± 8.63% of VB length, 48.48 ± 16.17% of VB height, and 68.94 ± 27.73% of vertebral canal diameter. Targeted dimensions were achieved at all intervertebral disc spaces (Table 1). Intervertebral disc space did not significantly affect corpectomy length (*P* = .827), height (*P* = .621), or depth (*P* = .994). Angled positioning significantly affected corpectomy length and height (Table 2).

**Conclusions and Clinical Importance:** Minimally invasive TLLC can be performed from T11‐12 through L3‐4. Angled positioning affects some surgical dimensions. This pilot study will guide future applications to clinical patients.

**Table:** **Table 1.** Corpectomy dimensions at each intervertebral disc space

Intervertebral disc space	% Vertebral body length	% Vertebral body height	% Vertebral canal diameter
T11–12	29.42 ± 7.12	57.87 ± 10.94	69.11 ± 33.08
T11‐13	25.14 ± 7.16	53.07 ± 18.24	67.08 ± 21.92
T13‐L1	22.60 ± 9.40	47.82 ± 14.68	65.23 ± 30.54
L1‐2	24.27 ± 9.29	39.77 ± 12.76	67.04 ± 30.04
L2‐3	21.74 ± 7.35	49.08 ± 17.57	72.99 ± 25.19
L3–4	24.76 ± 8.43	48.79 ± 16.21	73.60 ± 21.47
**All sites (n = 36)**	**24.17 ± 8.63**	**48.48 ± 16.17**	**68.94 ± 27.73**

Up to six surgeries were performed on each cadaver at T11‐12 (n = 4), T12‐13 (n = 5), T13‐L1 (n = 8), L1‐2 (n = 7), and L3‐4 (n = 5), alternating laterality on the left and right side of the cadaver. Data are reported as mean ± SD.

**Table:** **Table 2**. Corpectomy dimensions with various surgical positioning

Dimensions	Sternal recumbency	Angled away from surgeon	P value
% Vertebral body length	30.44 ± 6.15	20.63 ± 7.77	0.001
% Vertebral body height	59.75 ± 36.72	42.11 ± 14.88	0.001
% Vertebral canal diameter	59.30 ± 11.54	74.39 ± 18.95	0.002

Cadavers were positioned either in sternal recumbency (n = 3) or angled approximately 30‐60° away from the surgeon (n = 23). Data are reported as mean ± SD A p value <0.05 was considered significant.

## ABSTRACT N03: Single‐dose pharmacokinetics of intranasal levetiracetam in healthy dogs

### **Jessica Wagner** ^1^; Lauren Forsythe^2^, PharmD, DICVP; Kari Foss^3^, DVM, MS, DACVIM (Neurology); Jennifer Reinhart^4^, DVM, PhD, DACVIM (SAIM), DACVCP

#### ^1^University of Illinois at Urbana‐Champaign, Champaign, IL, USA; ^2^Assistant Professor of Social and Administrative Pharmacy, University of Findlay, Findley, OH, USA; ^3^Associate Professor, Neurology & Neurosurgery, Veterinary Clinical Medicine, University of Illinois at Urbana‐Champaign, Champaign, IL, USA; ^4^Assistant Professor, Small Animal Internal Medicine, Veterinary Clinical Medicine, University of Illinois at Urbana‐Champaign, Champaign, IL, USA

**Background:** Cluster seizures and status epilepticus in dogs are emergencies requiring rapid treatment. Intranasally delivered benzodiazepines have been shown to be effective for early seizure cessation. The pharmacokinetics of longer acting antiseizure drugs administered intranasally in dogs have not been previously investigated.

**Hypothesis/Objectives:** To describe the single‐dose pharmacokinetics of levetiracetam administered intranasally (IN) to healthy dogs compared to intravenous (IV) administration. The investigators hypothesized that IN levetiracetam has pharmacokinetic properties similar to IV administration.

**Animals:** Nine healthy dogs.

**Methods:** Dogs were prospectively enrolled in a randomized crossover study to receive a single 30 mg/kg dose of intravenous (100 mg/mL solution, commercial formulation) or intranasal (460 mg/mL solution, compounded formulation) levetiracetam. Serial serum samples were collected over 24 hours. Following a 7‐day washout period, this was repeated using the other formulation. Serum concentrations were quantified using LC‐MS. Pharmacokinetic analyses were performed using non‐compartmental methods. Comparisons between the formulations were made using a Wilcoxon signed‐rank test.

**Results:** C_max_ was 14.6 ± 5.4 μg/mL with a T_max_ of 2.3 ± 1.5 hours. The time to achieve therapeutic concentration (5 μg/mL) for IN was 0.34 ± 0.22 hours (IV 0.25 hours; *P* = 0.250) and remained above this threshold for 6.57 ± 3.17 hours (IV 9.77 ± 2.11 hours; *P* = 0.047). The bioavailability for IN administration was 70 ± 27.4%.

**Conclusions and Clinical Importance:** Levetiracetam reaches therapeutic concentrations rapidly when delivered intranasally and may be viable for emergent treatment of seizures when IV access is not available.

## ABSTRACT N04: Accuracy of a canine and feline CT‐guided 3D‐printed stereotactic brain biopsy guide using dental anchors

### **Mathieu Boutin** ^1^; Aude Castel^2^, DV, IPSAV, MSc, DACVIM (Neurology); Thomas Parmentier^3^, DMV, IPSAV, PhD, DACVIM (Neurology); Steeve Chantrel^4^, PhD Ing

#### ^1^Université de Montréal, Montréal QC, Canada; ^2^Assistant Professor and Head of the Neurology and Neurosurgery Department, Neurology and Neurosurgery, Université de Montréal, Montréal QC, Canada; ^3^Assistant Professor of Neurology and Neurosurgery, Neurology and Neurosurgery, Université de Montréal, Montréal QC, Canada; ^4^CEO, Infineis

**Background:** If few user‐friendly, precise, and non‐invasive stereotactic brain biopsy devices exist, the cost of the most advanced ones can be prohibitive and the risks of a breach in sterility and altered accuracy during placement remain bothersome.

**Hypothesis/Objectives:** To evaluate the accuracy of a novel, affordable computed tomography (CT)‐based, three‐dimensional (3D) printed stereotactic brain biopsy device for dogs and cats, using dental anchors (Infineis patent).

**Animals:** Cadavers of 4 dogs and 4 cats with varying head morphologies to assess the device's performance across different skull types and biopsy locations were imaged by CT.

**Methods:** Experimental study. Three target points (superficial: right frontal lobe, median: left caudate nucleus, deep: right piriform lobe) were selected per cadaver based on CT images allowing 3D printing of a headframe including three biopsy guides. Deviation between pre‐established targets and actual needle placement was calculated for each location and a 95% confidence interval (CI) for mean placement error was obtained. Differences between species and depth were evaluated using a Student's *t*‐test and Spearman's rank correlation respectively.

**Results:** Mean target point deviation was 1.24 mm (range: 0.16‐2.84 with a 95% CI of 1.01‐1.47 mm). Deviations for superficial, median, and deep target points were 1.11 mm (range: 0.60‐1.78), 1.24 mm (range: 0.16‐2.84) and 1.36 mm (range: 0.71‐2.07), respectively. No significant differences in accuracy between target point locations and species were observed (*P* = 0.30 and p = 0.93 respectively).

**Conclusions and Clinical Importance:** This 3D printed stereotactic brain biopsy device, employing dental anchors, demonstrated a high accuracy across a range of biopsy depth and species.

**Figure:**
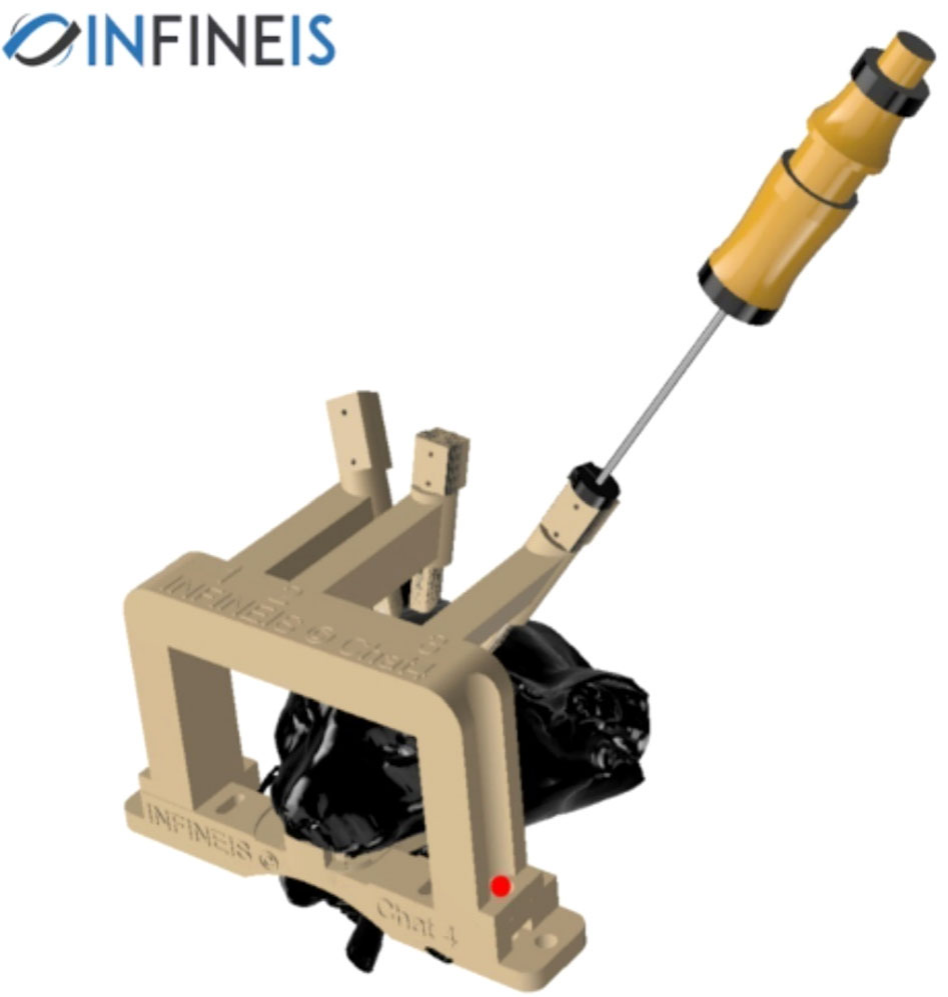
**Figure 1.** A 3D‐rendered model of a feline head featuring the custom‐made, CT‐based, 3D‐printed stereotactic brain biopsy device secured with dental anchors (Infineis Patent). The arch with the needle guides is connected to the mouth place using screws (red dot on the image) and can be detached and sterilized for use in surgery. It can then easily be reattached without compromising sterility during the surgical procedure.

**Figure:**
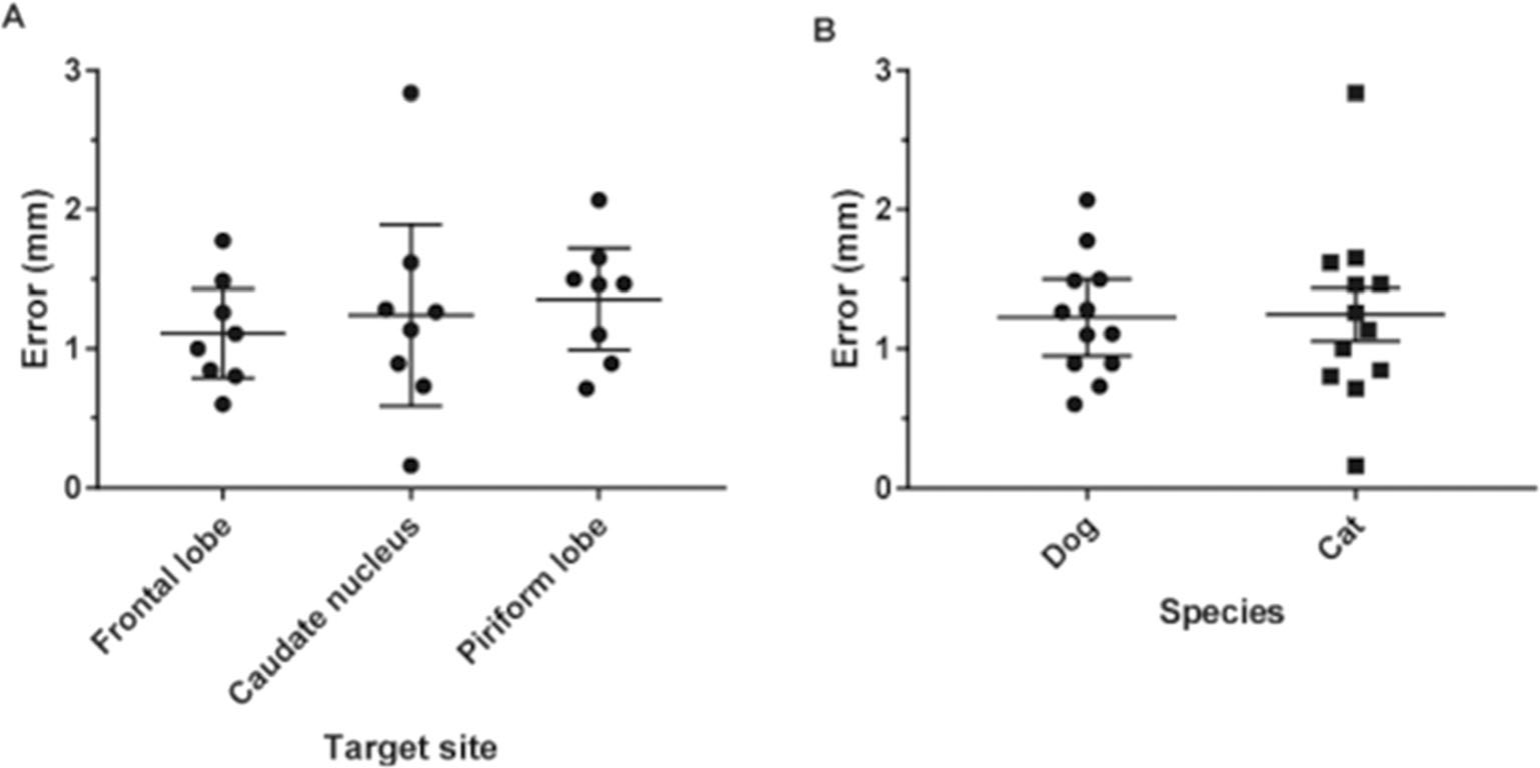
**Figure 2.** Error between planned and actual target points. Deviation from planned and actual target points was compared between depth of location (superficial: frontal lobe, medium: caudate nucleus and deep: piriform lobe) (A) and species (B). No statistically significant differences were observed (*P* = 0.30 and *P* = 0.93, respectively). Points represent individual measurements. The middle horizontal bars represent the means and the whiskers represent to 95% confidence interval of the mean.

## ABSTRACT N05: Perioperative assessment of electroencephalography in dogs with congenital portosystemic shunts

### **Adrien Dupanloup** ^1^; William Culp^1^, VMD, DACVS; Peter Dickinson^2^, BVSc, PhD, DACVIM (Neurology); Marguerite Knipe^1^, DVM, DACVIM (Neurology); Christine Toedebusch^1^, DVM, PhD, DACVIM (Neurology)

#### ^1^University of California‐Davis, Davis, CA, USA; ^2^School of Veterinary Medicine, University of California−Davis, Davis, CA, USA

**Background:** There are no known biomarkers to identify dogs at risk of developing seizures following surgical attenuation of congenital portosystemic shunts (CPS).

**Objectives:** To evaluate the association between perioperative electroencephalographic (EEG) findings and the development of post attenuation seizures (PAS).

**Animals:** 25 client‐owned dogs with CPS.

**Methods:** Cohort study. Dogs undergoing CPS attenuation underwent ambulatory EEG prior to and following surgery. EEG background activity and presence of paroxysmal discharges were assessed qualitatively. Quantitative analysis of randomly selected epochs included evaluation of the mean dominant frequency (MDF), and relative power of delta, theta, alpha, and beta frequency bands.

**Results:** Three dogs developed PAS. PAS occurred within 7 days in two dogs. These dogs had qualitative electroencephalographic abnormalities prior to attenuation and onset of neurological signs (paroxysmal discharges, persistent high amplitude theta background in both sleep and wakefulness, absence of sleep transients). Both dogs developed refractory seizures and progressive encephalopathy and were euthanized. One dog developed seizures at 35 days post attenuation and survived >1 year. Qualitative preoperative EEG was normal for 22 dogs without PAS. Quantitative analysis was available for 16 dogs without PAS, with mean MDF ± SD was 10.5 ± 0.3 Hz. MDF of dogs with PAS fell outside the MDF range for non‐PAS dogs (10.0‐10.9 Hz).

**Conclusions:** Based on preliminary results, EEG could provide useful diagnostic biomarkers to identify high‐risk dogs undergoing attenuation of CPS.

## ABSTRACT N06: Prognostic utility of F‐waves in paraplegic dogs with absent pain perception from intervertebral disc extrusion

### **Go Togawa** ^1^; Stephanie Thomovsky^2^, DVM, MS, DACVIM (Neurology), CCRP; R. Timothy Bentley^3^, BVSc (Dist) DACVIM (Neurology) MRCVS; Melissa Lewis^4^, VMD, PhD, DACVIM (Neurology)

#### ^1^Purdue University, West Lafayette, IN, USA; ^2^Associate Professor, Purdue University, West Lafayette, IN, USA; ^3^Professor, University of Liverpool, Liverpool, England; ^4^Associate Professor, North Carolina State University, Raleigh, NC, USA

**Background:** Approximately 50%‐60% of paraplegic deep pain negative (DPN) dogs with thoracolumbar intervertebral disc extrusion (TL‐IVDE) recover ambulation after surgery. F‐wave duration has been associated with injury severity in TL‐IVDE but the relationship to outcome is unknown.

**Hypothesis/Objectives:** Evaluate the prognostic utility of F‐waves in DPN dogs with TL‐IVDE.

**Animals:** Nineteen client‐owned dogs presented between 2021 and 2024.

**Methods:** Multicenter prospective, observational study. Acutely, paraplegic DPN dogs with TL‐IVDE managed surgically were included. F‐waves were performed within 24 hours postoperatively. F‐wave parameters included persistence, minimum latency, duration, amplitude, conduction velocity, F ratio, F:M ratio, and after‐discharge activity (scored from 0/none to 3/severe). Outcome was categorized as successful or unsuccessful with success defined as recovery of pain perception and independent ambulation at 3‐months postoperatively. Wilcoxon rank‐sum test compared F‐wave variables between dogs with a successful or unsuccessful outcome.

**Results:** Seven dogs had a successful outcome and 10 dogs were unsuccessful; 2 dogs were removed from analysis. In dogs with unsuccessful outcomes compared to those with successful outcomes (data displayed as median (range)), mean F‐wave duration was longer (30.2 ms (11.4‐54.6) vs. 18.2 ms (10.8‐27.3), *P* = .011), minimum latency was shorter (9.3 ms (5.7‐12) vs. 11.7 ms (9.2‐14.6), *P* = .04), maximum F‐wave amplitude was larger (1210 μV (400‐2375) vs. 577 μV (470‐1210), *P* = .04), and after‐discharge activity score was higher (2.6 (0.13‐3.0) vs. 0.4 (0.0‐2.5), *P* = .019).

**Conclusions and Clinical Importance:** F‐waves could aid in predicting outcome in DPN dogs with TL‐IVDE treated surgically.

## ABSTRACT N07: Diffusion‐weighted imaging of intracranial ring‐enhancing lesions

### **Adrien Dupanloup** ^1^; Peter Dickinson^2^, BVSc, PhD, DACVIM (Neurology)

#### ^1^University of California−Davis, Davis, CA, USA; ^2^Professor Neurology/Neurosurgery, Surgical, and Radiological Sciences, School of Veterinary Medicine, University of California−Davis, Davis, CA, USA

**Background:** Magnetic resonance imaging (MRI) of ring‐enhancing lesions results from various diseases, including infection, neoplasia, inflammation, and vascular etiologies. Differentiation based on standard MRI sequences can be challenging.

**Hypothesis:** Diffusion‐weighted imaging (DWI) of ring‐enhancing infectious disease will be highly restricted and allow differentiation from other ring‐enhancing etiologies.

**Animals:** 18 infectious, 54 noninfectious cases.

**Methods:** Retrospective study. Records were reviewed for MR studies with DWI and post gadolinium T1‐weighted ring‐enhancing lesions with histopathological diagnoses or a microbiological diagnosis of brain infection. Apparent diffusion coefficient (ADC) maps were generated. Normalized ADC values (rADC) were calculated using ADC values from lesional and contralateral brain (CB) regions of interest (rADC = ADClesion/ADCCB).

**Results:** Histopathological diagnoses of abscess were made in 16 cases. Two infectious cases had microbiological diagnosis only. Median rADC was significantly lower for intraparenchymal bacterial abscesses (0.54, n = 9) compared to ring‐enhancing gliomas (1.69, n = 18; *P* < .0001), non‐infectious inflammatory lesions (1.53, n = 12; *P* = .001), and other types of neoplasm (0.91, n = 12; *P* = .01). There was no significant difference between rADC for intraparenchymal abscess and intraparenchymal hemorrhage (0.55, n = 12; *P* = .73).

Median rADC was significantly lower for intraparenchymal bacterial abscesses (0.54, n = 9) compared to extraparenchymal bacterial abscesses (2.5, n = 6; *P* = .002), and intraparenchymal fungal abscesses (1.2, n = 3; *P* = .01). With exclusion of hemorrhagic lesions, an rADC of 0.61 had a sensitivity/specificity of 78%/90% for bacterial abscess.

**Conclusions and Clinical Importance:** DWI may differentiate bacterial abscess from other common ring‐enhancing lesions and abscess of fungal origin. Some uncommon neoplasia and hemorrhagic lesions may mimic intraparenchymal bacterial abscess on DWI.

## ABSTRACT N08: Canine meningiomas: surgical resection versus radiation therapy

### **Rachel Geiger** ^1^; Sheila Carrera‐Justiz^2^; Giunio Bruto Cherubini^3^; Joan Coates^4^; Steven De Decker^5^; Alex Forward^6^; Tom Harcourt‐Brown^7^; Daisuke Ito^8^; Nicholas Jeffery^2^; Marc Kent^9^; Charles Maitz^4^; Joseph Mankin^1^; Ada Naramor^4^; Karanbir Randhawa^10^; Jishnu Rao Gutti^2^; Elena Scarpante^11^; Lauren Smith‐Oskrochi^1^; Catherine Stalin^12^; Nathaniel van Asselt^10^; Holger Volk^5^; Joel White^10^

#### ^1^Texas A&M University, College Station, TX, USA; ^2^University of Florida, Gainesville, FL, USA; ^3^Dick White Referrals; ^4^University of Missouri, Columbia, MO, USA; ^5^The Royal Veterinary College, London, UK; ^6^Davies Veterinary Specialists; ^7^University of Bristol, Bristol, UK; ^8^Nihon University, Tokyo, Japan; ^9^University of Georgia, Athens, GA, USA; ^10^University of Wisconsin, Madison, WI, USA; ^11^Dick White Referrals; ^12^University of Glasgow, Glasgow, UK

**Objectives:** Comparison of survival following surgery or radiotherapy for intracranial meningioma in dogs using causal effects analysis, which optimizes treatment comparisons in observational data.

**Animals:** Total of 328 dogs, included if intracranial meningioma was the primary differential diagnosis on MRI and treated by surgery or definitive‐intent radiation. Primary outcome was all‐cause death.

**Methods:** Multi‐institutional retrospective study. Demographic data, treatment allocation, date and survival at last follow‐up was extracted from medical records from 2007 to 2023 at nine university veterinary hospitals (USA, n = 5; UK, n = 3, Japan, n = 1) and two private referral centers in England. Tumor volume was estimated using Osirix and expressed as a proportion of brain size.

**Results:** 134 (40.9%) dogs received surgery and 194 (59.1%) radiotherapy. Baseline demographics were similar between groups: median age 11 versus 9 years, median weight 19.8 versus 26.1 kg; median ratio of lesion to brain size 1758.9 versus 2523.1, in radiotherapy and surgery groups, respectively. Of 257 dogs with pretreatment seizures, 142 were treated surgically and 115 with radiotherapy. Caudotentorial lesions (n = 72) (versus rostrotentorial [n = 182]) were preferentially distributed towards radiotherapy (n = 66). Univariable Cox regression demonstrated a hazard ratio of 1.79 (95%CI: 1.37‐2.35) for surgery versus radiotherapy. Causal effects analysis indicated survival time after radiotherapy alone of 919 days and, on average, when also adjusting for age, tumor location and size, preoperative seizures, a reduction of 392 (95%CI: 654‐130) days associated with surgery.

**Conclusions and Clinical Importance:** In dogs treated for intracranial meningioma, radiotherapy was associated with a longer survival than surgery.

## ABSTRACT N09: Evaluation of plasma biomarkers in cats with and without evidence of feline cognitive dysfunction

### **Lizabeth C. Lueck** ^1^; Starr Cameron^2^; LaTasha Crawford^2^; Niwako Ogata^3^; Hsin‐Yi Weng^3^; Tessa Arendt^4^; Amy Elbe^2^; Lauryn Hahn^2^; Barbara Bendlin^2^; Henrik Zetterberg^5^; Gillian McLellan^2^

#### ^1^Department of Medical Sciences, School of Veterinary Medicine, University of Wisconsin‐Madison, Madison, WI, USA; ^2^University of Wisconsin‐Madison, Madison, WI, USA; ^3^Purdue University, West Lafayette, IN, USA; ^4^Texas A&M University, College Station, TX, USA; ^5^UK Dementia Research Institute

**Background:** Characteristic neuropathologic features of dementia in the human brain, including β‐amyloid (Aβ) in extracellular plaques and phosphorylated tau (pTau181) in neurofibrillary tangles, have been identified in older cats. However, these changes have not yet been directly correlated to clinical signs associated with feline cognitive dysfunction syndrome (FCDS).

**Objective:** Determine the association between FCDS and biomarkers of dementia‐related neurodegeneration in geriatric cats.

**Methods/Materials:** Owners completed a FCDS questionnaire and plasma samples were assayed for dementia‐associated biomarkers [Aβ40, Aβ42, pTau181, neurofilament light chain (NfL), and glial fibrillary acidic protein (GFAP)]. Geriatric cats ≥12 years of age had biochemistry, CBC, urinalysis, total T_4_, blood pressure, and neurologic and ophthalmologic examinations performed to screen for comorbidities. Young cats ≤6 years old underwent neurologic examinations and evaluation.

**Results:** One hundred and three geriatric cats and 31 young cats were prospectively enrolled. A significant positive correlation was identified between both Aβ40 and NfL plasma concentrations and FCDS survey scores (95% confidence interval, 0.012‐0.359 and 0.145‐0.470, respectively). Positive correlations were also identified between Aβ42 and GFAP plasma concentrations and FCDS survey scores but were not significant. NfL plasma concentrations showed the strongest association with FCDS scores and the largest difference between geriatric cats with and without comorbidities. pTau181 could not be quantified in feline plasma, serum, or CSF samples.

**Conclusion:** The biomarkers Aβ40, Aβ42, NfL, and GFAP were quantified in cat plasma, and Aβ40 and NfL plasma concentrations were significantly correlated with FCDS scores generated from owner survey responses.

## ABSTRACT N10: Vaccination and seasonality as risk factors for development of auto‐immune meningoencephalitis in dogs

### **Maria K. Johnson** ^1^; Rebecca Windsor^2^, DVM, DACVIM (Neurology); Jessica Schmidt^2^, DVM, DACVIM (Neurology); David Raczek^2^, DVM, DACVIM (Neurology); George Moore^3^, DVM, PhD, DACVIM (SAIM), DACVPM (Epi)

#### ^1^Wheat Ridge Animal Hospital; ^2^Veterinary Neurologist, Wheat Ridge Animal Hospital; ^3^Professor, Purdue University, West Lafayette, IN, USA

**Background:** Vaccination and seasonality are inconsistently reported risk factors for autoimmune diseases in dogs and humans. Prospective studies evaluating these risks in a large number of dogs with meningoencephalitis of unknown origin (MUO) and steroid‐responsive meningitis arteritis (SRMA) are lacking.

**Objectives:** To prospectively evaluate the association between vaccination and season and development of new‐onset MUO or SRMA.

**Animals:** 191 client‐owned dogs diagnosed with MUO (n = 172) or SRMA (n = 19) at a single private practice between August 2021 and July 2023.

**Methods:** Dogs were enrolled after meeting established diagnostic criteria for MUO or SRMA. Signalment, body weight, vaccination history, and season of onset of neurologic signs were recorded. Vaccination window was divided into 45‐day time intervals and statistically compared for MUO and SRMA (total and individually). Season of onset (winter, spring, summer, fall) was also compared.

**Results:** 50 (26.2%) dogs received no vaccinations in the previous year. Of the 141 dogs vaccinated in the previous year, more (24.8%) received vaccine(s) in the 45 days preceding clinical signs compared to any other 45‐day interval in the preceding year (*P* = .083). Vaccines administered most frequently included distemper/adenovirus/parvovirus/parainfluenza (25.7%), *Bordetella* (17.1%), and *Leptospira* (17.1%). There was no statistical difference between MUO and SRMA when evaluated separately. Cases were slightly more common in the spring (28.8%) and least common in the summer (19.9%) (*P* = .635).

**Conclusions and Clinical Importance:** This study demonstrated no overall statistical risk for development of MUO/SRMA post vaccination; the preceding 45 days was the highest risk window in vaccinated dogs. There was no seasonal risk for MUO or SRMA.

## ABSTRACT N11: A randomized controlled clinical trial of senolytic and NAD ^+^ precursor in aged companion dogs

### **Katherine E. Simon** ^1^; Katharine Russell^2^, DVM; Zachary Anderson^1^; Alejandra Mondino^1^, DVM, PhD; Chin‐Chieh Yang^1^, DVM; Beth Case^1^; Christine Whitley^1^, RVT; Emily Griffith^1^, PhD; Margaret Gruen^1^, DVM, MVPH, PhD, DACVB; Natasha Olby^1^, MB, PhD, MRCVS, DACVIM (Neurology)

#### ^1^North Carolina State University, Raleigh, NC, USA; ^2^Southeast Veterinary Neurology

**Background:** Companion dogs, like humans, experience age‐related cognitive decline and mobility loss. These changes begin at a molecular level and targeting cellular senescence and NAD^+^ depletion, both molecular hallmarks of aging, may delay clinical progression in senior dogs.

**Hypothesis/Objectives:** A combination of senolytic and NAD^+^ precursor (LY‐D6TM) will improve cognition and activity over a 3‐month period.

**Animals:** Seventy companion dogs (age > 10 years) with mild to moderate cognitive impairment.

**Methods:** This randomized controlled clinical trial tested two doses of LY‐D6TM (half, full) against placebo. Primary outcomes were change in canine cognitive dysfunction rating (CCDR) score (completed by caregiver) and in physical activity monitor counts. Dogs were evaluated at baseline, 1,3 and 6 months, with 3 months as the primary endpoint. Repeated measures ANOVA was used to compare changes in outcome over time with *P* < 0.05 indicating significance. Covariates were incorporated when significant.

**Results:** After enrollment, two dogs died and nine dogs were excluded from analysis (noncompliance, significant health changes and inability to complete visits), leaving 20 dogs in the placebo group, 21 in half dose, and 18 in full dose at 3 months. There was a significant difference in CCDR score over time across treatment groups, with those receiving LY‐D6TM decreasing more than placebo. (Table 1). There was no difference between groups in activity level across time. (Table 2).

**Conclusions and Clinical Importance:** A combination of NAD^+^ precursor and senolytic shows evidence of improving cognitive health in aged dogs.

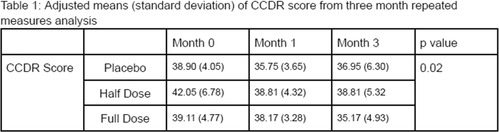

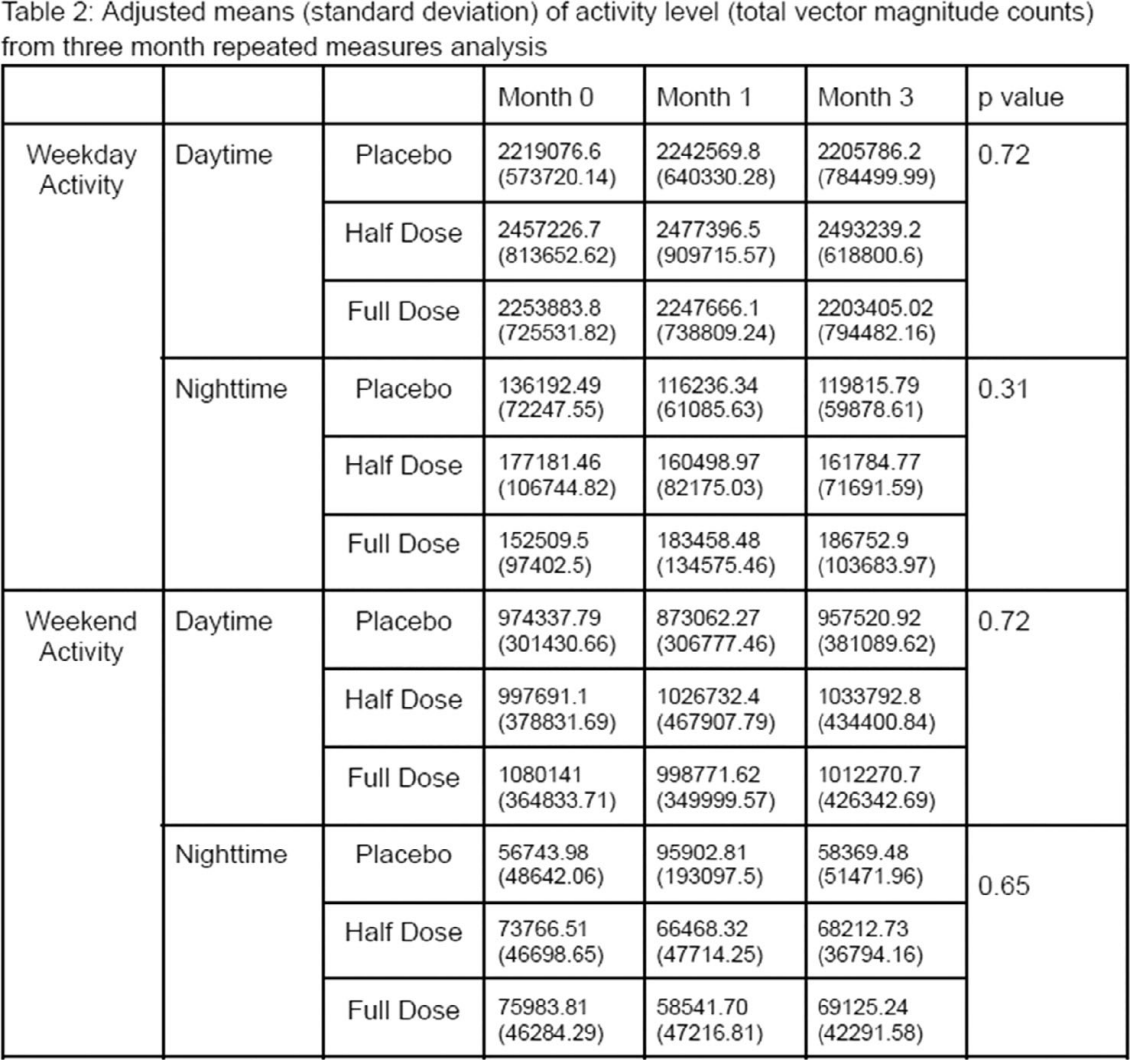

acvim24_576.

## ABSTRACT N12: Creation of a breed‐specific neurologic disease online database for veterinary practitioners

### **Kat A. Dalzell** ^1^; Rodney Bagley^2^, DVM, DACVIM (Neurology and Internal Medicine), CCRP, CVA; Avinash Bevoor^3^; Sara Shivapour^4^, DVM; Amanda Zanger^5^

#### ^1^Iowa State University, Ames, IA, USA; ^2^Professor, Neurology and Rehabilitative Medicine, Clinical Sciences, Iowa State University, Ames, IA, USA; ^3^Veterinary Student, Iowa State University, Ames, IA, USA; ^4^Clinician, Iowa State University, Ames, IA, USA; ^5^United States Army

**Background:** Breed‐related neurologic disease identification expands notably each year. Currently, no peer‐reviewed repository exists for clinicians to succinctly compare clinical signs and pertinent information to support a presumptive clinical diagnosis.

**Objective:** The goal of this project is to generate an open‐access repository of breed‐related neurologic diseases, including clinically relevant information to aid in the identification of these diseases in clinical practice.

**Methods:** The American and United Kennel Club registered breed lists were utilized, supplemented with additional breeds known to have breed‐related neurologic conditions. Disease names were collected from published texts describing canine neurologic disease. The following publicly available internet platforms were accessed: Pubmed, Web of Science, Google Scholar, and Google search platforms. The keywords searched included: breed and (neurologic disorder, nervous, disease) or (clinical signs) or (additional neurologic terms). Retrieved information was stored in Excel format categorized by breed and disease, with updated searches performed weekly. Original peer‐reviewed manuscripts (written in English) were downloaded and reviewed. Data extracted included breed, number of animals affected, sex, age of onset of signs, clinical signs, and other pertinent clinical information.

**Results:** Over 411 breeds were reviewed for peer‐reviewed evidence of neurologic disease, with over 1500 scholarly citations subsequently identified and stored in an Excel‐based system.

**Conclusions and Clinical Importance:** An open‐access, contemporary, single‐site, and easily searchable repository of canine neurologic disease will assist primary care clinicians and practicing neurologists with comparison of the immediate clinical presentation to historically reported information and subsequently aid clinical diagnosis.

## ABSTRACT N13: S100B as a potential biomarker of non‐infectious inflammatory central nervous system diseases in dogs

### Joonghyun Song; Bokyung Kim

#### Department of Veterinary Internal Medicine, College of Veterinary Medicine, Chungnam National University, Daejeon, Korea

**Background:** At present, there is no established biomarker for diagnosing non‐infectious inflammatory central nervous system diseases. S100B, an astrocytic protein, is associated with inflammatory processes in the central nervous system.

**Hypothesis:** We hypothesized that S100B would be elevated in dogs with MUO compared to healthy controls (HC) and idiopathic epilepsy (IE), suggesting its potential as a biomarker for MUO.

**Animals:** Forty‐one client‐owned dogs. 14 dogs with MUO, 13 dogs with IE, and 14 healthy dogs.

**Methods:** A case‐control study. S100B was estimated using a canine‐specific enzyme‐linked immunosorbent assay kit. Nonparametric and inferential statistics were used.

**Results:** Cerebrospinal fluid (CSF) S100B levels were significantly increased in MUO dogs compared to both HC (*P* = .000) and IE (*P* = .005). Serum S100B levels were also significantly higher in MUO dogs compared to HC (*P* = .000) and IE (*P* = .000). There is no significant difference in S100B levels between HC and IE in both CSF (*P* = .323) and serum (*P* = .155). A significantly strong correlation was found between S100B levels in CSF and serum (rs = 0.652, *P* = .000). In receiver operating characteristic (ROC) curve analysis for MUO versus control with CSF S100B, a cutoff value of 699.11 pg/mL showed 85.7% sensitivity, 84.2% specificity; at 835.82 pg/mL, sensitivity was 71.4%, specificity 100%. For serum S100B, a cutoff value of 782.39 pg/mL had 100% sensitivity, 84.2% specificity, and 977.06 pg/mL showed 85.7% sensitivity, 100% specificity.

**Conclusions:** Both CSF and serum S100B could be potential diagnostic biomarkers for MUO.

## ABSTRACT N14: Preliminary investigation of paraspinal musculature magnetic resonance imaging (MRI) characteristics in canine degenerative myelopathy

### **Croix Griffin** ^1^; Alexandra Scharf^2,3^, DVM, PhD, DACVR; Joan Coates^4^, DVM, MS, DACVIM; Sarah Moore^1,5^

#### ^1^College of Veterinary Medicine, The Ohio State University, Columbus, OH, USA; ^2^Adjunct Assistant Professor; Staff Radiologist, Large Animal Diagnostic Imaging; ^3^Antech Imaging Services, University of Pennsylvania, New Bolton Center, Kennett Square, PA, USA; ^4^Neurology, Professor of Neurology and Neurosurgery, Department of Veterinary Medicine and Surgery, College of Veterinary Medicine, University of Missouri, Columbia, MO, USA; ^5^Director of BluePearl Science; Adjunct Professor of Neurology and Neurosurgery, BluePearl Science

**Background:** Changes in T2‐weighted (T2W) MRI signal intensity of paraspinal musculature can serve as an imaging biomarker in amyotrophic lateral sclerosis (ALS) and may reflect intramuscular fat accumulation or edema. Given disease similarities to canine degenerative myelopathy (DM), exploration of this phenomenon in DM is warranted.

**Hypothesis:** Dogs with DM will demonstrate increased T2 signal intensity in the paraspinal musculature compared to controls.

**Animals:** Dogs previously imaged by MRI; autopsy confirmed with DM (7) and controls (6).

**Methods:** Retrospective, blinded, single observer, pilot investigation using images acquired on a 3.0 T scanner. Spinal cord diameter and T2 signal intensity of longissimus (l.) thoracis (T8) and l. lumborum (L1) were measured in a sagittal plane. Triplicate measurements assessed intra‐rater agreement. Data were expressed as median (range). Wilcoxon rank sum tests compared signalment data and muscle T2 signal intensity between groups, p.

**Results:** There were no significant group differences in age, weight, or adiposity (Table 1). ICC was excellent for all MRI measurements (r > 0.94). Spinal cord diameter (mm) at T13 was smaller (*P* = .035) in DM‐affected dogs (3.7; 3.4‐4.5) than controls (4.4; 3.9‐4.9). Muscle T2 signal intensity was higher in DM‐affected dogs compared to controls at all sites, but when scaled to background signal significance remained only in the right l. lumborum (Table 2).

**Conclusions and Clinical Importance:** These findings invite investigation in a larger cohort to confirm true differences in DM‐affected dogs, expand muscle measurements to additional imaging planes, and correlate with disease severity.

**Table:** **Table 1.** Group characteristics and comparisons for dogs with autopsy confirmed DM (7) and control dogs (6) *P* < .05 is significant

Parameter	DM‐affected (range)	Control (range)	*P* value
Breeds included	Boxer dog (2), Chesapeake Bay retriever (2), Pembroke Welsh corgi (2), Mixed breed dog	Mixed breed dog (2), Boxer dog (2), Chesapeake Bay retriever, Labrador retriever	Not assessed
Median body weight (kg)	27.9 (9.9‐39.1)	21.9 (5.7‐35.0)	.5991
Median age (years)	10.0 (8.9‐13.5)	6.8 (5.0‐11.1)	.0804
Adiposity (subcutaneous fat height in mm at the level of T8)	7.5 (0.8‐9.7)	3.3 (0.7‐9.2)	.4691

**Table:** **Table 2.** T2W muscle intensity (unitless value) measured in the right (R) and left (L) longissimus *(*l.) thoracis and l. lumborum muscles of DM‐affected (n = 7) and controls (n = 6), expressed as raw value (signal intensity) and scaled to background intensity (scaled intensity). *P* < .05 is significant

Parameter	DM‐affected (range)	Control (range)	*P* value
**Signal intensity**			
l. thoracis (R)	862.1 (602‐1609)	479.3 (310‐699.6)	.0047
l. thoracis (L)	741.0 (594‐1828)	418.0 (304‐661.7)	.0023
l. lumborum (R)	1188 (538‐2185)	510.0 (313‐687)	.0221
l. lumborum (L)	800.0 (413‐1927)	447.0 (256‐760)	.0221
**Scaled intensity**			
l. thoracis (R)	2.67 (1.632‐5.162)	2.58 (1.527‐3.074)	.2949
l. thoracis (L)	2.47 (1.525‐4.001)	2.26 (1.469‐2.804)	.1807
l. lumborum (R)	3.05 (1.886‐12.51)	2.03 (1.550‐2.887)	.0350
l. lumborum (L)	2.64 (1.507‐4.589)	1.86 (1.306‐4.222)	.4452

## ABSTRACT N15: Cerebrospinal fluid concentrations of calcitonin gene related peptide in dogs with chiari‐like malformation

### **John Macri**; Natasha Olby; Courtney Whicker

#### North Carolina State University, Raleigh, NC, USA

**Background:** Cavalier King Charles Spaniels (CKCS) have a high prevalence of Chiari‐like malformation and syringomyelia (CMSM). Dogs with CMSM display signs of neuropathic pain and itch. Imaging findings often do not correlate with severity of signs. Neuropeptides including calcitonin gene related peptide (CGRP) have been implicated in neuropathic pain.

**Hypothesis:** Cerebrospinal fluid (CSF) concentrations of CGRP (C‐CGRP) will correlate with CMSM clinical sign severity.

**Animals:** Twenty‐nine CKCS.

**Methods:** CKCS underwent pain and itch quantification by owners (questionnaires) and investigators (physical examination), a brain and cervical spine MRI, and lumbar CSF collection. CSF was frozen to −80 °F. C‐CGRP in CSF were measured using an ELISA assay (MyBiosource, San Diego, CA). Dogs were grouped by presence of syrinx, pain, and itch, and C‐CGRP were compared between groups using Wilcoxon Rank Sum; C‐CGRP and owner reported pain score were evaluated using linear regression analysis.

**Results:** 15/29 CKCS had SM on MRI, 13/29 were painful, and 12/29 were scratching. C‐CGRP was significantly higher in painful dogs (median 114.3, range 11.6‐238.3 pg/mL) and dogs with itch (111.67, 38.5‐234.8 pg/mL) compared to non‐painful dogs (72.57 pg/mL, 0‐266.2 pg/mL) (*P* = .025) and dogs without itch (79.7, 0‐266.2 pg/mL). There was a significant correlation between owner pain score and C‐CGRP (R2 = 18%, *P* = 0.033). No significant difference in C‐CGRP was noted between SM+ (99, 0‐226.6 pg/mL) and SM‐ (102.2, 5.9‐266.2 pg/mL) dogs (*P* = .25).

**Conclusions:** CSF CGRP concentration is elevated in CKCS exhibiting pain and itch. CGRP may contribute to neuropathic pain in CMSM and could be a target for therapeutic intervention.

## ABSTRACT N16: Recovery of ambulation in dogs with T3‐L3 and L4‐S3 myelopathies following hemilaminectomy for acute IVDE

### Stephanie Marzullo

#### BluePearl Pet Hospital North Dallas, Lewisville, TX, USA

**Background:** Limited and conflicting information is published about whether neurolocalization affects recovery of ambulation following hemilaminectomy for acute intervertebral disc extrusion (IVDE).

**Objective:** Evaluate recovery of ambulation in dogs with T3‐L3 myelopathies versus L4‐S3 myelopathies following acute IVDE in canine patients 4‐6 weeks after surgery.

**Hypothesis:** Dogs with T3‐L3 myelopathies would be more likely to be ambulatory after 4‐6 weeks compared to those with L4‐S3 myelopathies.

**Animals:** 147 client‐owned dogs from one referral hospital obtained over a two‐year period (2020 to 2021) that had a hemilaminectomy and returned for a follow‐up visit 4‐6 weeks later.

**Methods:** Dogs were separated into two groups based on neurolocalization, T3‐L3 or L4‐S3 myelopathy. Comparisons were made by assigning a modified Frankel score (MFS) at three time points: at the time of pre‐operative exam, prior to discharge from surgery, and at a recheck exam 4‐6 weeks after surgery. The highest MFS score (MFS 6) indicated the dog was neurologically normal, whereas MFS 0 indicated the dog was paraplegic without nociception.

**Results:** Neurolocalization of signs did not have a significant association with MFS prior to discharge (*P* = .419; *P* = .603 multivariable) or at recheck (*P* = .485; *P* = .656 multivariable). Being older at time of surgery significantly increased the odds of having a lower MFS at the 4‐6 week recheck (*P* = .018). Suspected spinal shock significantly increased the odds of having a lower MFS prior to discharge (*P* = .008).

**Conclusions and Clinical Importance:** Neurolocalization was not associated with recovery of ambulation following hemilaminectomy.

## ABSTRACT N17: Clinical outcome and side effects of procarbazine in 67 dogs with presumptive meningoencephalitis

### **Savannah Giannasi** ^1^; Elizabeth Parsley^2^, DVM, DACVIM (Neurology); Laura Harvey^3^, DVM, DACVIM (Neurology); Stephanie Pumphrey^4^, DVM, PhD, DACVO; Dominik Faissler^5^

#### ^1^Cummings School of Veterinary Medicine at Tufts University, North Grafton, MA, USA; ^2^Faculty, Neurology and Neurosurgery, Cummings School of Veterinary Medicine at Tufts University, North Grafton, MA, USA; ^3^Compass Veterinary Neurology and Imaging; ^4^Faculty, Ophthalmology, Cummings School of Veterinary Medicine at Tufts University, North Grafton, MA, USA; ^5^Faculty, Neurology and Neurosurgery, Cummings School of Veterinary Medicine at Tufts University, North Grafton, MA, USA

**Background:** Meningoencephalomyelitis of unknown etiology (MUE) encompasses a group of idiopathic and non‐infectious inflammatory diseases affecting the central nervous system (CNS). Procarbazine is a recognized therapeutic option for MUE able to cross the blood brain barrier, but limited data exist regarding its effectiveness in dogs.

**Objectives:** To elucidate the clinical outcomes and side effects in dogs with a clinical diagnosis of MUE treated with procarbazine and prednisone.

**Animals:** 67 client‐owned dogs.

**Methods:** Retrospective study of dogs with a presumptive diagnosis of MUE treated with prednisone (median dose 2.4 mg/kg/day) and procarbazine (median dose 40.2 mg/m2).

**Results:** Treatment with prednisone and procarbazine resulted in a median survival time of 1923 days (range 45‐3026 days). At the time of data collection, 27/67 (40.2%) of dogs had spontaneously died (n = 1) or been euthanized (n = 26). The cause of death included MUE (n = 8), side effects (n = 5), other (n = 10), and unknown cause (n = 4). Hematologic abnormalities were the most common side effect occurring in 37/67 of dogs (55.2%) with 4/67 (6.0%) being euthanized due to significant myelosuppression. Suspected retinal degeneration was identified in 9/67 (11.9%) of dogs.

**Conclusions and Clinical Importance:** Treatment with prednisone and procarbazine resulted in a longer median survival time than previously reported treatment protocols for MUE. Hematologic abnormalities were the most common side effect and underscore the need for frequent blood work and clinical monitoring in dogs treated with procarbazine. This study identified retinal toxicity as a possible side effect of procarbazine not previously reported in dogs.

**Table:** **Table 1:** Serum uremic toxin concentrations before and after administration of Porus One. Data are displayed in median ng/ml (range)

	Average baseline (Days −56, −28, and 0)	Average after Porus One (Days 28 and 56)	*P* value	
Indoxyl sulfate	1922 (1030‐4876)	1592 (944‐4669)	.03	
p‐Cresol sulfate	11 487 (1906‐39 913)	6956 (277 ng/ml–30 345)	.005	
	Day 0	Day 28	Day 56	*P* value
Indoxyl sulfate	1689 (1257‐2862)*	1422 (664‐4192)*	1616 (1157‐4322)	.01*
p‐Cresol sulfate	12 720 (268‐34 341)*	7850 (263‐27 758)*	8778 (290‐33 824)	.008*

## ABSTRACT N18: Assessment of recommended cerebrospinal fluid volume collection in dogs and cats

### **Bruno Benetti Giunta Torres** ^1^; Luana Ribeiro^2^, PhD student; Ítalo Iara^2^, PhD student; Aude Castel^3^, DEV, MSc, DACVIM (Neurology)

#### ^1^Federal University of Goiás; ^2^UFG; ^3^Adjunct Professor, University of Montreal

**Background:** A previous recommendation to limit cerebrospinal fluid (CSF) collection to 0.2 mL/kg to avoid complications such as subdural hemorrhages has not been validated in dogs and cats.

**Hypothesis/Objectives:** The 0.2 mL/kg recommendation for the maximal amount of CSF collection can be safely exceeded for diagnostic purposes.

**Animals:** Client‐owned dogs and cats with neurological diseases requiring CSF collection for diagnosis purposes.

**Methods:** Retrospective study. Cases were included when information on the amount of CSF collected, and potential post‐procedural complications was available. CSF collection was performed at the cerebellomedullary cistern in all patients until the flow stopped naturally. Fourteen samples were divided into groups: lower (G1) vs. higher (G2) than 0.20 mL/kg of CSF collected. Between‐group comparison for signalment, body weight (BW), CSF analysis, and complications was performed using unpaired Student's *t*‐tests.

**Results:** G1 contained six samples (five dogs) and G2 contained eight samples (four dogs, and two cats) with one dog per group having more than one CSF tap at different times. BW (*P* = .0134) and age (*P* = .0007) in G1 (25.4 ± 13.5 Kg; 106 ± 36,7 mo) were higher than in G2 (5.2 ± 3.9 Kg; 29 ± 28 mo). The mean CSF amount per kg of BW was lower for G1 (0.13 ± 0.03 mL/Kg) than G2 (0.37 ± 0.17 mL/Kg) (*P* = .006), although the total final volume was similar. Iatrogenic blood contamination was observed in one dog per group. No complications or neurological deterioration were observed in either group. CSF analysis was similar across groups.

**Conclusions and Clinical Importance:** CSF can be safely collected until the flow stops naturally allowing collection of an adequate amount for diagnostic purposes.

## ABSTRACT N19: Accuracy of urinary dipsticks for glucose and protein determination in cerebrospinal fluid of dogs

### **Aryanne R. Ottoboni** ^1^; Luana Ribeiro^1^; Gladston Filho^1^; Paulo Gonçalves^1^; David Matta^1^; Adilson Damasceno^1^; Danieli Martins^1^; Aude Castel^2^; Bruno Torres^1^

#### ^1^UFG; ^2^University of Montreal, Montreal, QC, Canada

**Background:** Urinary dipsticks (UD) provide a bedside test to estimate protein and glucose concentration in cerebrospinal fluid (CSF) in humans. However, only one study suggests it as a screening test for estimating CSF proteins but not glucose in dogs.

**Hypothesis/Objectives:** Evaluate the precision of UD to assess proteins and glucose concentration in CSF samples of dogs with CNS disorders compared to the standard method.

**Animals:** 22 samples of CSF from dogs with CNS diseases.

**Methods:** CSF proteins and glucose were measured with UD and biochemistry (pyrogallol red and glucose oxidase reaction, respectively) in each sample. Results were converted into scores to allow comparison between methods. A proportion of divergence between methods and its confidence interval were calculated using the z‐test, with a significance of 0.05. The sensitivity (Se), specificity (Sp), positive (PPV), and negative predictive (NPV) values, and accuracy (Ac) of UD were determined for two cut‐off levels of CSF proteins (15 mg/dL and 30 mg/dL) and glucose (40 mg/dL and 100 mg/dL).

**Results:** The proportion of divergence between methods for CSF proteins was 64% (CI: 44‐84%) and CSF glucose was 73% (CI: 54‐91%). Only eight CSF proteins and four CSF glucose had equal results for the two methods. UD had better results as a screening test when the cut‐off level was 15 mg/dL for proteins (Se: 78.9%; Sp: 66.7%; PPV: 93.7%; NPV: 33.3%; Ac: 77.3%) and 40 mg/dL for glucose (Se: 89.5%; Sp: 33.3%; PPV: 89.5%; NPV: 33.3%; Ac: 81.8%).

**Conclusions and Clinical Importance:** The urinary dipstick is unreliable to estimate CSF proteins or glucose.

## ABSTRACT N20: Metabolic acidosis associated with zonisamide administration in dogs with idiopathic epilepsy: A prospective study

### Quentin Reuet

#### Montreal University, Montreal, Canada

**Background:** Zonisamide(ZNS) is commonly used to treat idiopathic epilepsy in dogs. While acid‐base imbalances are well‐known side effects of ZNS in humans, little is known about such alterations in dogs.

**Hypothesis/Objectives:** Evaluate and characterize the occurrence of acid‐base imbalances in epileptic dogs treated with ZNS and test for correlations with ZNS blood level.

**Animals:** Fourteen client‐owned dogs with confirmed or suspected idiopathic epilepsy receiving ZNS at standard doses.

**Methods:** Prospective longitudinal study. Acid‐base parameters, including bicarbonates, PCO2, base excess(BE), chloremia, anion gap, urinary and blood pH, were compared at baseline(T0), 1‐2 weeks(T1), and 10‐14 weeks(T2) post‐ZNS initiation. ZNS serum level was measured at T1 and/or T2. Metabolic acidosis (MA) criteria were bicarbonates<20 mmol/L or BE<‐4 mmol/L. Data were assessed for normality using a Shapiro‐Wilk test and variables were compared using a paired t‐test or a Wilcoxon signed‐rank test. Spearman rank correlation was used to evaluate correlations between ZNS level and other variables. A *P*‐value<.05 was considered significant.

**Results:** All dogs developed MA after treatment initiation. Bicarbonates (mean T0: 22.4 mmol/L vs. T1: 17.6 mmol/L, p < 0,0001 and T2: 17.6 mmol/L, *P* < .0001) and BE (mean T0: −1.5 mmol/L vs. T1: −6.4 mmol/L, *P* < .0001 and T2: −6.2 mmol/L, *P* = .0002) were significantly decreased whereas chloremia (median T0: 116.5 mmol/L vs T1: 120 mmol/L, *P* = .013 and T2: 119 mmol/L, *P* = .026) increased at T1 and T2 compared to T0. No symptoms of MA other than transiently decreased appetite in 4 dogs were reported. ZNS level was not correlated with any acid‐base parameters.

**Conclusions and Clinical Importance:** ZNS administration in dogs is associated with the development of MA.

## ABSTRACT NE01: Epidemiological characteristics and risk factors associated with neurological manifestation of canine distemper virus

### **Bruno Benetti Giunta Torres** ^1^; Italo lara^2^; Heloisa l. Freire^2^ (she/her/hers); Luana S. Ribeiro^2^ (she/her/hers); Karolina M. Menezes^2^ (she/her/hers); Paulo A. Gonçalves^2^ (she/her/hers); David H. Matta^2^ (he/him/his)

#### ^1^Federal University of Goiás, Goiânia, GO, Brazil; ^2^Universidade Federal de Goias, Goiânia, GO, Brazil

**Background:** Canine distemper virus (CDV) infection persists as one of the most lethal diseases in domestic dogs in tropical countries.

**Hypothesis/Objectives:** Identify the epidemiological characteristics and potential risk factors associated with CDV infection in dogs with neurological manifestations.

**Animals:** Seventeen CDV‐infected dogs with neurological signs and 343 dogs with other CNS diseases.

**Methods:** Retrospective study (2018‐2022). CDV‐infected dogs were confirmed by immunochromatography antigen test, RT‐PCR, and/or Lentz corpuscle observation. Dogs with other CNS diseases were included in a control group. Age, breed, weight, sex, and neuter status were compared between groups by logistic regression (*P* < .05), log‐likelihood method, and odds ratios were calculated. Clinical signs, seasonality, and vaccination protocols were described. Prevalence, mortality, lethality, survival, and time until death were calculated.

**Results:** Younger dogs had more probability of having neurological signs caused by distemper (*P* = .00690; OR = ‐0.01438). Shih‐Tzu (*P* = .00007; OR = 1.53774) and Lhasa Apso (*P* = 0.000264; OR = 1.76084) were more prone to develop neurological manifestations due to CDV than other breeds. Most of the CDV‐infected dogs showed multifocal (10/17) and associated extra‐neural signs (16/17). The most common neurological sign was motor deficit (13/17), and only one‐third (6/17) showed myoclonia. Autumn was the season with the highest occurrence (8/17). Many CDV‐infected dogs had updated vaccination protocol (6/17). Prevalence, mortality, and lethality were 4,72%, 2,22%, and 47,05%, respectively. Mean survival was 187 ± 12 months, and the death occurred on average after 74 ± 12 months.

**Conclusions and Clinical Importance:** The epidemiological characteristics and risk factors identified constitute important tools for better prevention of canine distemper infection in endemic regions.

## ABSTRACT NE02: Diagnosis and management of movement disorder (paroxysmal dyskinesia) in small‐breed dogs

### **Woo‐Jin Song**; Jeongbae Choi; Yunhee Joung; Minji Kim; Ujin Kim; Unghui Kim; Sooyoung Son; Young Min Yun

#### Jeu National University, Jeju City, South Korea

**Background:** Paroxysmal dyskinesia (PD) is a subtype of movement disorder characterized by recurrent, self‐limiting abnormal and involuntary movements. In human medicine, PD is categorized into three distinct types based on the triggering behavior for symptom manifestation, and genetic mutations. However, in veterinary medicine, given the involvement of numerous breeds, the application of human criteria becomes ambiguous due to the presence of diverse genetic mutations. Also, studies of PD are limited.

**Objectives:** To describe how PD was diagnosed through video data and questionnaires filled out by owners, as well as how the patients were managed nutritionally and pharmacologically.

**Animals:** Four small‐breed dogs diagnosed with PD.

**Methods:** Case series.

**Results:** This case series describes four dogs with paroxysmal dyskinesia (PD) presented for abnormal movement episodes. All patients were diagnosed by recorded video of episode and assessing motor activity, consciousness, duration, pre‐ or post‐episodic behavior and the presence of autonomic signs. Magnetic resonance imaging studies in two dogs were unremarkable. Gluten free diet along with acetazolamide administration for two dogs, and gluten free diet for two dogs was given for treatment trial. The frequency of abnormal movement episode decreased in all patients.

**Conclusion and Clinical Relevance:** PD can be diagnosed through detailed symptom description using videos and questionnaires. Once diagnosed, nutritional and medical management can be attempted for these dogs.

## ABSTRACT NM01: Evaluation of a commercially available water supplement to promote hydration in clinically ill cats

### **Maria Peralta** ^1^; Amy Nichelason^2^, DVM

#### ^1^University of Wisconsin‐Madison, Madison, WI, USA; ^2^Clinical Assistant Professor, Primary Care, School of Veterinary Medicine, University of Wisconsin‐Madison, Madison, WI, USA

**Background:** Rehydration in clinically ill cats typically requires intravenous or subcutaneous fluids, adding cost and stress for clients and patients. Purina Hydra Care is a palatable oral hydration supplement that increases water consumption in healthy cats. We hypothesize that it improves hydration in clinically ill cats.

**Objectives:** The study aims were to determine whether Hydra Care improves hydration measures in clinically ill cats, invokes a minimum fluid intake of 30 ml/kg/day and improves quality of life (QoL) assessments.

**Animals:** 13 clinically ill cats eligible for outpatient management were enrolled.

**Methods:** Prospective single arm clinical trial. Cats were offered Hydra Care per the label. The amount of Hydra Care and water consumed was measured. Serum osmolality, BUN, albumin, PCV/TP, USG, and clinical assessment were performed at baseline and 48 hr alongside an owner QoL assessment.

**Results:** All cats exceeded the minimum fluid intake to enhance hydration (>30 ml/kg/day) although there were no significant improvements in biochemical measures of hydration. Cats drank a median of 39 ml/kg/day of Hydra Care™ which was significantly greater than water (*P* = .007). All cats appeared better hydrated (*P* = .004) with improved QoL scores (*P* = .001).

**Conclusions:** Hydra Care™ was preferred over water in clinically ill cats. Hydra Care™ intake was comparable to the volume of SC fluids typically administered to marginally hydrated cats. While laboratory hydration measures did not significantly change over 48 hr, clinical hydration and QoL scores improved. This highlights the utility of HydraCare™ as an alternative to subcutaneous fluids in clinically ill cats.

## ABSTRACT NM02: Evaluating copper and zinc solubility in canine diets using an in vitro model

### **Kyle German** ^1^; Terry Engle^2^, PhD; Huey Yi Loh^2^; Camille Torres‐Henderson^3^, DVM, DABVP, DACVIM (nutrition)

#### ^1^Colorado State University Teaching Hospital, Fort Collins, CO, USA; ^2^Department of Animal Science, Colorado State University, Fort Collins, CO, USA; ^3^ Veterinary Teaching Hospital, Colorado State University, Fort Collins, CO, USA

**Background:** An increase in reported cases of copper hepatitis in dogs and the absence of established safe upper limits for copper content in dog food necessitate understanding copper composition of diets. Examining copper solubility allows for a broader understanding of factors that may contribute to this disease.

**Objective:** The study aim was to assess the solubility of copper and zinc in dog food using a previously validated in vitro digestion model.

**Methods:** A total of 63 diets, including both prescription and over‐the‐counter varieties, underwent simulation of gastric digestion by using HCL and pepsin, followed by small intestinal digestion with digestive enzymes and buffers to assess copper and zinc solubility during each phase. Analysis also compared solubility percentages among different diet types to see if other characteristics of the diet could affect copper absorption.

**Results:** Preliminary results show the median copper concentration in solution post gastric digestion was 0.265 ppm, while following intestinal digestion, it was 0.297 ppm. The median zinc concentration post gastric digestion was 5.50 ppm and decreased to 1.604 ppm after intestinal digestion. The median solubility of copper after intestinal digestion between diets was 48.3% and ranged from 29.2% to 92.4%, revealing a wide range in solubility percentages.

**Conclusion:** These results offer insights into the dynamics of copper and zinc solubility during digestion in dog food using an in vitro model. This emphasizes the need for further exploration into factors influencing copper solubility and how it may contribute to hepatic copper accumulation in dogs.

## ABSTRACT NM03: Assessment of lean, fat, and total body mass changes with age in dogs and cats

### **Allison P.** **McGrath** ^1^ ; Leslie Hancock^2^; Cheryl Stiers^3^; Elizabeth Morris^4^

#### ^1^Hill's Pet Nutrition; ^2^Chief Medical Officer, Hill's Pet Nutrition; ^3^Clinical Research Technician, Hill's Pet Nutrition; ^4^Senior Scientist, Hill's Pet Nutrition

**Background:** Age‐related changes in body composition, specifically a loss of lean mass and gain in fat mass, have been associated with negative health effects in dogs and cats. This retrospective study aimed to establish a baseline for how body composition changes with age and life stage.

**Hypothesis/Objectives:** To use body composition data from dual‐energy X‐ray absorptiometry (DEXA) scans to characterize changes in lean, fat, and total body mass with age in dogs and cats.

**Animals:** 7088 observations from 1331 colony‐housed dogs and 6635 observations from 1111 colony‐housed cats.

**Methods:** Historical DEXA data from 2006 to 2023 were analyzed using a linear model with orthogonal contrasts to determine the relationship between age and lean, fat, and total mass.

**Results:** Age had a significant effect on all body composition measures in both dogs and cats (*P* < .001). In dogs, lean mass peaked at age 5 and generally decreased from age 5 onward, while fat mass on average increased until age 12 before declining. In cats, lean mass peaked at age 5 and generally decreased from age 5 onward, while fat mass on average increased until age 8 and generally decreased from age 8 onward.

**Conclusions and Clinical Importance:** By establishing a baseline of how lean, fat, and total body mass differ with age in dogs and cats, veterinarians may use these data to better assess body composition in patients, prepare for age‐related changes such as sarcopenia and obesity, which may begin earlier than currently expected, and measure the impact of preventative interventions.

## ABSTRACT NM04: Utility of FitBark to monitor activity in obese dogs undergoing a structured weight loss program

### **Jenessa A. Winston** ^1^; Hailey Lenis^2^; Nora Jean Nealon^2^; Adam Rudinsky^2^; Hannah Klein^1^; Valerie Parker^2^

#### ^1^College of Veterinary Medicine, The Ohio State University, Columbus, OH, USA; ^2^Department of Veterinary Clinical Sciences, College of Veterinary Medicine, The Ohio State University, Columbus, OH, USA

**Background:** The obesity epidemic is associated with canine mobility issues. Wearable activity monitors (eg, FitBark) are increasingly used to evaluate mobility. While weight loss is widely believed to be associated with improved mobility, there are limited studies using activity monitors to examine mobility changes during structured weight loss programs in dogs.

**Hypothesis/Objectives:** This project aimed to examine relationships between weight loss, activity levels, and scores on the validated multidimensional canine quality of life survey (QoL) and Liverpool osteoarthritis in Dogs (LOAD) questionnaire. We hypothesized that weight loss would improve QoL and LOAD scores while increasing activity assessed via FitBark.

**Animals:** Twenty‐five client‐owned obese dogs underwent a 24‐week structured weight loss program as part of the Canine SLIM clinical trial.

**Methods:** Owners completed LOAD surveys every 3 weeks and QoL every 12 weeks. Daily activity was tracked with FitBark devices. QoL scores and fold changes of FitBark activity and LOAD scores were examined for changes over time with repeated measures analysis of variance with significance defined as *P* < .05.

**Results:** Weight loss improved physical dimension of QoL scores when comparing baseline to week 24 (Friedman test, *P* = .0075). Significant changes in LOAD scores were noted between week 12‐24 (Holm‐Sidak's test, *P* < .02). No significant differences in weekly FitBark activity were observed.

**CONCLUSIONS/CLINICAL IMPORTANCE:** These results demonstrate that weight loss improves owner perceived physical QoL while maintaining mobility. The FitBark device provides a user‐friendly platform to monitor activity in obese dogs undergoing weight loss.

## ABSTRACT NM05: Therapeutic renal diets differentially influence ionized and total calcium in cats with early‐stage renal disease

### **Elizabeth Morris** ^1^; Jean Hall^2^, DVM, PhD, DACVIM (SAIM); Dale Fritsch^3^, MS; Kim Wilson^4^, PhD

#### ^1^Hill's Pet Nutrition; ^2^Carlson College of Veterinary Medicine, Oregon State University, Corvallis, OR, USA; ^3^Hill's Pet Nutrition, Inc; ^4^Hill's Pet Nutrition

**Background:** Chronic kidney disease (CKD) is a known risk factor for hypercalcemia in cats. Mechanistically, phosphorus restricted diets have also been implicated, potentially because phosphorus restriction increases the Ca:P ratio.

**Hypothesis/Objectives:** The primary objective of this study was to evaluate the impact of different therapeutic renal foods on ionized (iCa) and total (tCa) calcium in cats with early‐stage CKD.

**Animals:** Twenty colony‐housed cats with stage 1 or 2 CKD were enrolled.

**Methods:** Two diets formulated for renal disease were utilized in a randomized, 140‐day crossover study. One food provided 1.5 g/Mcal phosphorus and a Ca:P ratio of 1.2 (M‐R); the other provided 1.1 g/Mcal phosphorus and Ca:P of 2.0 (H‐R). Blood and urine samples were collected on days 0, 28, 86, and 140 before and after crossover. Data were analyzed using a linear mixed model with fixed effects of diet, period, day, and associated interactions.

**Results:** At baseline, all cats had iCa within the normal reference interval (1.10‐1.30 mmol/L). At d 28 and thereafter, cats fed H‐R had higher iCa (1.48 ± 0.04 mmol/L) compared with cats fed M‐R (1.26 ± 0.01 mmol/L; *P* < .001). Results were similar for tCa (normal reference interval: 8.80 to 10.00 mg/dL; A: 9.29 ± 0.10 mg/dL; B: 11.36 ± 0.36 mg/dL; *P* < .001).

**Conclusions and Clinical Importance:** These data suggest that therapeutic renal diets may impact calcium status in cats with early‐stage CKD, but the effect is formulation‐dependent. Cats fed M‐R maintained normal iCa and tCa, suggesting it is a safe and well‐accepted option for cats with early‐stage renal disease.

## ABSTRACT NM06: Prevalence and risk factors of feline obesity in client‐owned cats in Goiânia, Brazil

### **Ana Rita Carvalho Pereira** ^1,2^; Danilo Silva^3^; Fabio Teixeira^1^; Emmanuel Arnhold^3^; Ana Rita Pereira^1^; Vivian Pedrinelli^4^; Mariana Yukari Porsani^5^

#### ^1^School of Veterinary Medicine and Animal Science, University of São Paulo, São Paulo, São Paulo, Brazil; ^2^GastroVet; ^3^Universidade Estadual de Goiás, nápolis, GO, Brazil; ^4^NutricareVet, Brazil; ^5^Anclivepa‐SP, Belenzinho, São Paulo, SP, Brazil

**Background:** Obesity is a disease with various consequences. Few studies have assessed the prevalence of feline obesity, especially those conducted through home visits.

**Objectives:** To ascertain the prevalence of obesity among client‐owned cats in Goiânia (Goiás, Brazil) and investigate obesity‐related risk factors.

**Methods:** A cross‐sectional study was conducted, encompassing cats and their owners, with household sampling carried out through geographical stratification. The animals were categorized based on their body condition score (BCS) as lean, ideal, overweight, or obese. The obesity prevalence was calculated. Association between BCS categories and characteristics of the animals, owners, environment, and cat handling practices was examined using Chi‐square analysis (*α* = 5%).

**Animals:** A total of 1043 households were visited, and 188 cats from 80 households were included, averaging 2.35 cats per household.

**Results:** Body condition scoring revealed: 30.3% underweight, 41% ideal weight, 20.2% overweight, and 8.5% obese cats. Factors associated with BCS (higher obesity proportions) included: reproductive status (neutered, *P* = .002), sex and reproductive status (neutered males, *P* = .012), age (cats between 7 and 10 years; *P* = .046), age at neutering (neutering up to 1 year old, *P* = .013), vaccination (annually, *P* = .008), veterinary visits (more visits, *P* = .009). Owners who perceived themselves as consuming little had the highest frequency of obese cats (*P* = .035), and household type (house or apartment) influenced BCS (*P* = .002).

**Conclusion:** The prevalence of overweight and obese cats was 28.7%. Assessing feline obesity prevalence through home sampling is crucial for understanding environmental factors influencing health and implementing targeted interventions.

## ABSTRACT NU01: A pilot study of burst wave lithotripsy for treatment of obstructive ureteroliths in cats

### **Adam Hunt** ^1^; Adam Maxwell^2^; Jody Lulich^1^, DVM, PhD, DACVIM; Michael Bailey^2^; Kaizer Contreras^3^; Ga Won Kim^3^; Marissa Torre^2^, DVM, DACVIM; Michael Borofsky^2^; Eva Furrow^2^, VMD, PhD, DACVIM

#### ^1^University of Minnesota, Minneapolis, MN, USA; ^2^Department of Urology, School of Medicine, University of Washington, Seattle, WA, USA; Center for Industrial and Medical Ultrasound, Applied Physics Laboratory, University of Washington, Seattle, WA, USA; ^3^Center for Industrial and Medical Ultrasound, Applied Physics Laboratory, University of Washington, Seattle, WA, USA

**Background:** Ureteroliths are a major source of morbidity and mortality in cats. In human trials, burst wave lithotripsy (BWL) is safe and efficacious for extracorporeal treatment of ureteroliths.

**Hypothesis/Objectives:** The objective of this study was to obtain pilot data on the safety and efficacy of BWL for treatment of obstructive ureteroliths in cats.

**Animals:** Three cats with obstructing ureteroliths.

**Methods:** Phase 1 clinical trial. BWL was performed under general anesthesia using ultrasound guidance. Adverse events were recorded and characterized by severity and relationship to BWL. Technical success was defined as resolution of obstruction, urolith passage, or apparent fragmentation.

**Results:** One cat had bilateral obstructions, and two had unilateral obstructions. In total, four ureters and seven ureteroliths were treated with BWL. Two cats required a second treatment due to persistent (1) or worsening (1) obstruction; the worsening of obstruction was classified as a major adverse event possibly related to BWL. Following BWL, fragmentation was apparent in six of seven ureteroliths, of which three passed into the bladder prior to discharge. All cats had a reduction in serum creatinine (pretreatment concentrations of 1.6, 2.0, and 2.4 mg/dL and post treatment concentrations of 1.5, 1.7, and 1.3 mg/dL, respectively). All cats survived to discharge.

**Conclusions and Clinical Importance:** Based on preliminary data, BWL shows promise as a minimally invasive therapy for obstructing ureteroliths in cats. In cats with multiple ureteroliths, more than one treatment might be necessary. Additional cases and follow up are warranted to determine how BWL outcomes compare to current treatment modalities.

## ABSTRACT NU02: Estimates of urinary calcium excretion in dogs with and without calcium oxalate urolithiasis

### **Danielle E.** **LaVine** ^1^ ; Emily Coffey^2^, DVM, PhD, DACVIM (SAIM); Jody Lulich^3^, DVM, PhD, DACVIM (SAIM); Jennifer Granick^4^, DVM, PhD, DACVIM (SAIM); Eva Furrow^5^, VMD, PhD, DACVIM (SAIM)

#### ^1^University of Minnesota, Minneapolis, MN, USA; ^2^Assistant Professor of Small Animal Medicine, University of Minnesota, Minneapolis, MN, USA; ^3^Director, MN Urolith Center, University of Minnesota, Minneapolis, MN, USA; ^4^Associate Professor, University of Minnesota, Minneapolis, MN, USA; ^5^Associate Professor of Small Animal Medicine, University of Minnesota, Minneapolis, MN, USA

**Background:** Excessive urine calcium excretion (hypercalciuria) impacts management of calcium oxalate (CaOx) urolithiasis in dogs. Fractional excretion of calcium (FeCa) and urine calcium‐to‐creatinine ratios (UCaCr) estimate hypercalciuria, but more data is needed on how well they discriminate between dogs with and without CaOx urolithiasis.

**Hypothesis/Objective:** To determine the performance of FeCa and UCaCr in predicting CaOx status.

**Animals:** 121 client‐owned, normocalcemic dogs (42 CaOx cases, 79 controls).

**Methods:** Analytical, cross‐sectional study. Fasting urine calcium and creatinine and blood ionized calcium and creatinine concentrations were obtained. FeCa and UCaCr were calculated and compared by CaOx status with Wilcoxon rank‐sum tests. Performance was determined at multiple thresholds, and receiver operating characteristic curves were generated. Potential predictors of log‐transformed FeCa and UCaCr (eg, sex, breed, age, blood ionized calcium concentration) were evaluated with multivariable regression. Spearman's rank correlation was run for FeCa and UCaCr.

**Results:** FeCa and UCaCr were greater in CaOx cases (median = 0.81% and 0.060, respectively) than controls (median = 0.50% and 0.032, respectively; *P* < .001 for both). Table 1 shows performance at multiple thresholds. FeCa and UCaCr were highly correlated. (rho = 0.94, *P* < .001), and both were lower in males (estimate = −0.70 and −0.64, *P* = .002 and .005 respectively).

**Conclusions and Clinical Importance:** FeCa or UCaCr perform moderately well for identifying CaOx cases; those with high values might benefit from therapy to reduce hypercalciuria. Given their high correlation, determination of both appears unnecessary. Lower values in males support the need for sex‐specific thresholds.

**Table:** Table 1

Thresholds	Sensitivity (95% CI)	Specificity (95% CI)	PPV (95% CI)	NPV (95% CI)
FeCa (%)				
0.4	0.81 (0.69‐0.93)	0.41 (0.30‐0.52)	0.42 (0.36‐0.48)	0.80 (0.68‐0.91)
**0.55**	**0.74 (0.59‐0.85)**	**0.57 (0.46‐0.68)**	**0.48 (0.40‐0.56)**	**0.8 (0.71‐0.9)**
0.6	0.64 (0.50‐0.78)	0.65 (0.54‐0.75)	0.49 (0.40‐0.59)	0.77 (0.70‐0.85)
0.8	0.50 (0.36‐0.64)	0.72 (0.62‐0.82)	0.49 (0.37‐0.61)	0.73 (0.67‐0.80)
UCaCr (mg/mg)				
0.03	0.79 (0.64‐0.91)	0.47 (0.35‐0.58)	0.44 (0.38‐0.51)	0.80 (0.71‐0.89)
0.05	0.60 (0.45‐0.74)	0.70 (0.59‐0.80)	0.51 (0.40‐0.62)	0.76 (0.69‐0.84)
**0.06**	**0.57 (0.43‐0.71)**	**0.75 (0.64‐0.85)**	**0.55 (0.43‐0.67)**	**0.77 (0.7‐0.83)**
0.07	0.38 (0.24‐0.52)	0.84 (0.75‐0.91)	0.55 (0.40‐0.71)	0.72 (0.67‐0.77)

Performance of fractional excretion of calcium (FeCa) and urine calcium to creatinine ratios (UCaCr) for prediction of calcium oxalate urolithiasis in dogs. Optimal thresholds, as determined by receiver operating characteristic curves, are in bold. FeCa—fractional excretion of calcium, NPV—negative predictive value, PPV—positive predictive value, UCaCr—urinary calcium to creatinine ratio.

## ABSTRACT NU03: Kidney injury biomarkers are more correlated with proteinuria than serum creatinine in telmisartan treatment study

### **Alisa S. Berg** ^1^; Andrew Specht^2^; Rebeca Castro^2^; Shir Gilor^2^; Kirsten Cooke^2^; Autumn Harris^2^

#### ^1^Small Animal Hospital, University of Florida, Gainesville, FL, USA; ^2^College of Veterinary Medicine, University of Florida, Gainesville, FL, USA

**Background:** Urine biomarkers such as neutrophil gelatinase associated lipocalin (uNGAL) and gamma‐glutamyl transpeptidase (uGGT) may be more sensitive for acute kidney injury caused by telmisartan treatment than increases in serum creatinine (sCr).

**Objective:** Planned interim analysis of prospective cohort study to determine if urine biomarkers provide evidence of kidney injury not apparent with sCr after telmisartan.

**Animals:** Seven of 11 proteinuric study dogs starting treatment with telmisartan and four of seven control dogs with chronic kidney disease had all samples available at time of interim analysis.

**Methods:** Urine protein to urine creatinine ratio (uP:uC), uNGAL, uNGAL:uC, uGGT, uGGT:uC, sCr and other parameters were evaluated at baseline (T0) and 3.5 m ± 1 m later (T1). Normality was assessed by Shapiro‐Wilks. Data was compared within and between groups using a *t*‐test for normally distributed and Man‐Whitney U for non‐normally distributed data. Correlation between biomarkers and sCr or uP:uC were evaluated using Spearmann correlation.

**Results:** There were no significant within‐group differences between T0 and T2 (no evidence of adverse telmisartan effect). The study group had lower sCr and higher uP:uC at both time points (*P* < .005). There was significant positive correlation between uP:uC vs. uNGAL:uC (rho = 0.864, *P* < .001) and uGGT:uC (rho = 0.920, *P* < .001) that is not primarily related to urine concentration.

**Conclusions:** Interim analysis showed greater differences between study and control groups at baseline than expected and no evidence of adverse treatment effect. However, results suggest that proteinuria may be associated with elevated kidney injury biomarker levels.

## ABSTRACT NU04: Retrospective evaluation of complications associated with ultrasound‐guided percutaneous renal biopsy in dogs

### Jasmine K. Zaibek

#### The Schwarzman Animal Medical Center

**Background:** Renal biopsy is a diagnostic for assessment of proteinuria and renal disease. Major reported complications in 2005 were hemorrhage and death. Despite changes in technique there has not been a re‐evaluation of complications associated with this procedure.

**Objective:** To evaluate major complications associated with percutaneous ultrasound guided renal biopsy in dogs and to identify risk factors contributing to these complications.

**Animals:** 76 dogs undergoing percutaneous ultrasound‐guided renal biopsy.

**Methods:** Multicenter, retrospective, observational study.

**Results:** Major complications were death and hemorrhage. Of the dogs that died none were associated with the procedure. Severe hemorrhage appeared in 6/76 (7.8%) of patients. Pearson Chi‐squared testing identified a positive correlation between elevated creatinine and severe hemorrhage (*P* = .08). Dogs with a creatinine ≥5 mg/dL had a higher probability of hemorrhage (OR = 2.5). There was a positive correlation between older age and severe hemorrhage (*P* = .01). Hypertension (*P* = .14) and weight (*P* = .8) were not associated with an increased risk of complications. Hospitalized patients had a higher probability of hemorrhage (OR = 1.7) compared to outpatients.

**Conclusions:** Ultrasound guided percutaneous renal biopsies have a low occurrence of severe hemorrhage in dogs and can safely be performed on an out‐patient basis. Patient variables have the potential to impact occurrence of complication and should be considered in the decision to pursue biopsy.

## ABSTRACT NU05: Urinary protein banding patterns as potential biomarkers of non‐neoplastic prostatic diseases in intact male dogs

### **Sara Wilkes** ^1^; Jessica Hokamp^2^; Deborah Keys^3^; Ewan Wolff^4^

#### ^1^BluePearl Pet Hospital; ^2^International Veterinary Renal Pathology Service, Texas A&M University, College Station, TX, USA; ^3^Kaleidoscope Statistics; ^4^BluePearl Portland, Portland, OR, USA

**Background:** No prior study has investigated urinary proteins as potential biomarkers of prostatic disorders in intact male dogs.

**Hypothesis/Objectives:** We hypothesized that urine from intact male dogs with non‐neoplastic prostatic diseases would contain significantly greater electrophoretically resolved protein bands compared with healthy intact and neutered male dogs.

**Animals:** 19 client‐owned healthy neutered male dogs (neutered control), 16 client‐owned unaffected intact male dogs (intact control), 5 client‐owned intact male dogs with clinical signs of non‐neoplastic prostatic disease (benign prostatic hyperplasia or prostatitis) confirmed via cytology (intact affected).

**Methods:** Prospective cohort study. Urine proteins were electrophoretically resolved into low‐molecular‐weight (LMW, <40 kDa), intermediate molecular weight (IMW, 40‐66 kDa), high‐molecular‐weight (HMW, 66‐200 kDa), and very high‐molecular‐weight (VHMW, >200 kDa) protein bands. Negative binomial regressions were used to estimate incident rate ratios (95% CI) and to test for significant differences in numbers of protein bands in each molecular weight region between groups.

**Results:** Urine from intact affected dogs contained significantly more LMW bands (*P* < .001), IMW bands (*P* < .001), HMW bands (*P* = .001), and VHMW bands (*P* = .004) compared to intact controls and significantly more LMW bands (*P* < .001) in intact controls than neutered controls.

**Conclusions and Clinical Importance:** While further exploration is warranted, increased urine protein bands across all molecular weight categories may serve as biomarkers for prostatic disorders in intact male dogs. These bands may include prostate‐specific proteins such as canine prostate‐specific esterase (CPSE).

## ABSTRACT NU06: Determining within‐individual and between‐subject biological variation in urine ammonia to creatinine ratio in healthy adult dogs

### **Riley J. Claude** ^1^; Autumn Harris^1^, DVM, DACVIM (SAIM); Andrew Specht^1^, DVM, DACVIM; Penelope Reynolds^1^, PhD; Rebeca Castro^2^, BS; Kirsten Cooke^2^, DVM, DACVIM

#### ^1^University of Florida, Gainesville, FL, USA; ^2^Biological Scientist, University of Florida, Gainesville, FL, USA

**Background:** Impaired renal ammonia production plays a significant role in the pathogenesis of metabolic acidosis in patients with chronic kidney disease (CKD) and may have prognostic value. Measurement of urinary ammonia, reported as urine ammonia‐to‐creatinine ratio (UACR) is the gold standard for evaluation. The biological variability of UACR and its impact on diagnostic utility is unknown in healthy dogs and those with CKD.

**Hypothesis/Objectives:** The primary aim of this study was to determine intra‐individual and between‐subject biological variation of UACR within‐day and week‐to‐week in healthy adult dogs.

**Animals:** Twenty‐eight adult, client‐owned dogs considered healthy based on history, physical examination, serum chemistry, and urinalysis.

**Methods:** Prospective observational cohort study. Daily collection of urine samples from each dog at 0, 4, and 8 hours, repeated once weekly for six weeks. Urinary ammonia and creatinine concentrations were measured using commercially available enzymatic assays and used to calculate UACR. Ammonia, creatinine and UACR were analyzed using linear hierarchical mixed models. Repeatability was quantified by calculating intraclass correlation coefficient.

**Results:** Average week to week values were relatively stable (ammonia *P* = .33, creatinine *P* = .85, UACR *P* = .42). Within‐day repeatability was poor because of strong effects of time of day on creatinine.

**Conclusions and Clinical Importance:** Within‐individual UACR in healthy dogs showed minimal variation week‐to‐week. Time of day in which samples are collected impacts UACR individual repeatability and should be standardized between measurements.

## ABSTRACT NU07: Utilizing the ellik evacuator during cystoscopic retrieval of uroliths in 12 dogs: a descriptive study

### **John A. Shamoun** ^1^; Shelly Vaden^2^, DVM, PhD, DACVIM; Patty Secoura^3^

#### ^1^College of Veterinary Medicine, North Carolina State University, Raleigh, NC, USA; ^2^Professor, Department of Clinical Sciences, College of Veterinary Medicine, North Carolina State University, Raleigh, NC, USA; ^3^Lead Technician, Small Animal Internal Medicine, College of Veterinary Medicine, North Carolina State University, Raleigh, NC, USA

**Background:** Urocystolithiasis is a common problem in dogs and approximately half of canine uroliths are nondissolvable. Novel management options are continuously being explored to improve current urolith retrieval strategies.

**Hypothesis/Objectives:** To describe a previously underreported technique using cystoscopy and the Bard Ellik Evacuator (EE) suction device to assist in minimally invasive retrieval of canine uroliths, and to describe patient demographic data, procedure type and duration, and clinical outcomes.

**Animals:** 12 client‐owned dogs presented with urocystolithiasis.

**Methods:** In this descriptive study, records of dogs undergoing cystoscopic urolith retrieval utilizing the Ellik Evacuator were evaluated retrospectively. Retrieval using EE is described in detail. Patient and clinical data are reported; for quantitative variables, the median and range are provided.

**Results:** 12 dogs underwent 13 cystoscopic procedures utilizing EE. Most (10/12) dogs were spayed females; eight different breeds were represented. The median age was 10 y (range 2‐11 y), and median weight was 8 kg (range 5.5‐37 kg). In 8/13 procedures, transurethral cystoscopy alone using EE ± wire basket and/or grasping forceps was performed (median procedure duration 36 minutes; range 16‐52 minutes). In 5/13 procedures, additional interventions including laser lithotripsy, perineal approach, etcetera were performed as indicated (median procedure duration 110 minutes; range 42‐144 minutes). No significant complications were noted; complete retrieval of uroliths was documented in 12/13 cases.

**Conclusions/Clinical Importance:** Use of the EE during cystoscopy is described, and the device may aid in minimally invasive retrieval of uroliths. Further investigation is needed to determine its benefit relative to current interventions.

## ABSTRACT NU08: Feline small intestine regional distribution of mRNA expression of phosphate cotransporters and intestinal alkaline phosphatase

### **Jason P. Bestwick** ^1^; Jonathan Elliott^2^, MA Vet MB PhD, Cert SAC Dip ECVPT MRCVS; Isabel Orriss^3^, BSc PhD FHEA; Rebecca Geddes^4^, MA VetMB GPCert (FelP), MVetMed PhD DACVIM (SAIM) MRCVS

#### ^1^Royal Veterinary College, London, England, UK; ^2^Professor of Veterinary Clinical Pharmacology, Comparative Biomedical Sciences, Royal Veterinary College, London, England, UK; ^3^Associate Professor, Comparative Biomedical Sciences, Royal Veterinary College, London, England, UK; ^4^Clinical Science and Services, Royal Veterinary College, London, England, UK

**Background:** Dietary phosphate restriction is the mainstay of management for cats with chronic kidney disease (CKD), however, molecular mechanisms of feline intestinal phosphate metabolism/transport remain unexplored.

**Hypothesis/Objectives:** (1) determine mRNA expression of 3 phosphate cotransporters (NaPi2b/SCL34A2, PiT1/SLC20A1 and PiT2/SLC20A2) and intestinal alkaline phosphatase (IAP/ALPI; catalyst of phosphate monoester hydrolysis, thereby aiding intestinal phosphate absorption) in feline small intestine; (2) qualitatively describe IAP distribution.

**Animals:** Intestinal samples acquired, with informed consent, during post‐mortem examination from 16 client‐owned cats euthanised for health reasons.

**Methods:** Quantitative PCR was used to assess relative gene expression (RGE) of SLC34A2, SLC20A1, SLC20A2 and ALPI in RNAlater‐fixed intestinal samples (14 cats), using ribosomal protein S7 as a reference gene. Formalin‐fixed paraffin‐embedded intestinal samples (15 cats) were stained for IAP using a naphthol‐azo‐dye‐based histochemistry.

**Results:** SLC34A2 RGE increased sequentially from duodenum to ileum, whereas SLC20A1 and SLC20A2 RGE was highest in the jejunum (Figure 1). ALPI expression was not significantly different between intestinal regions (Figure 1). IAP staining was positive at the level of the brush border in all intestinal regions, but results were negatively affected by delays in sample harvesting or prolonged storage in formalin.

**Conclusions and Clinical Importance:** The regional variation of active phosphate cotransporters in the small intestine differs from rodents and thus appears unique to the cat. IAP is expressed ubiquitously (mRNA and protein) throughout the feline small intestine. These molecules could represent future therapeutic targets for reducing intestinal phosphate absorption in cats with CKD.

**Figure:**
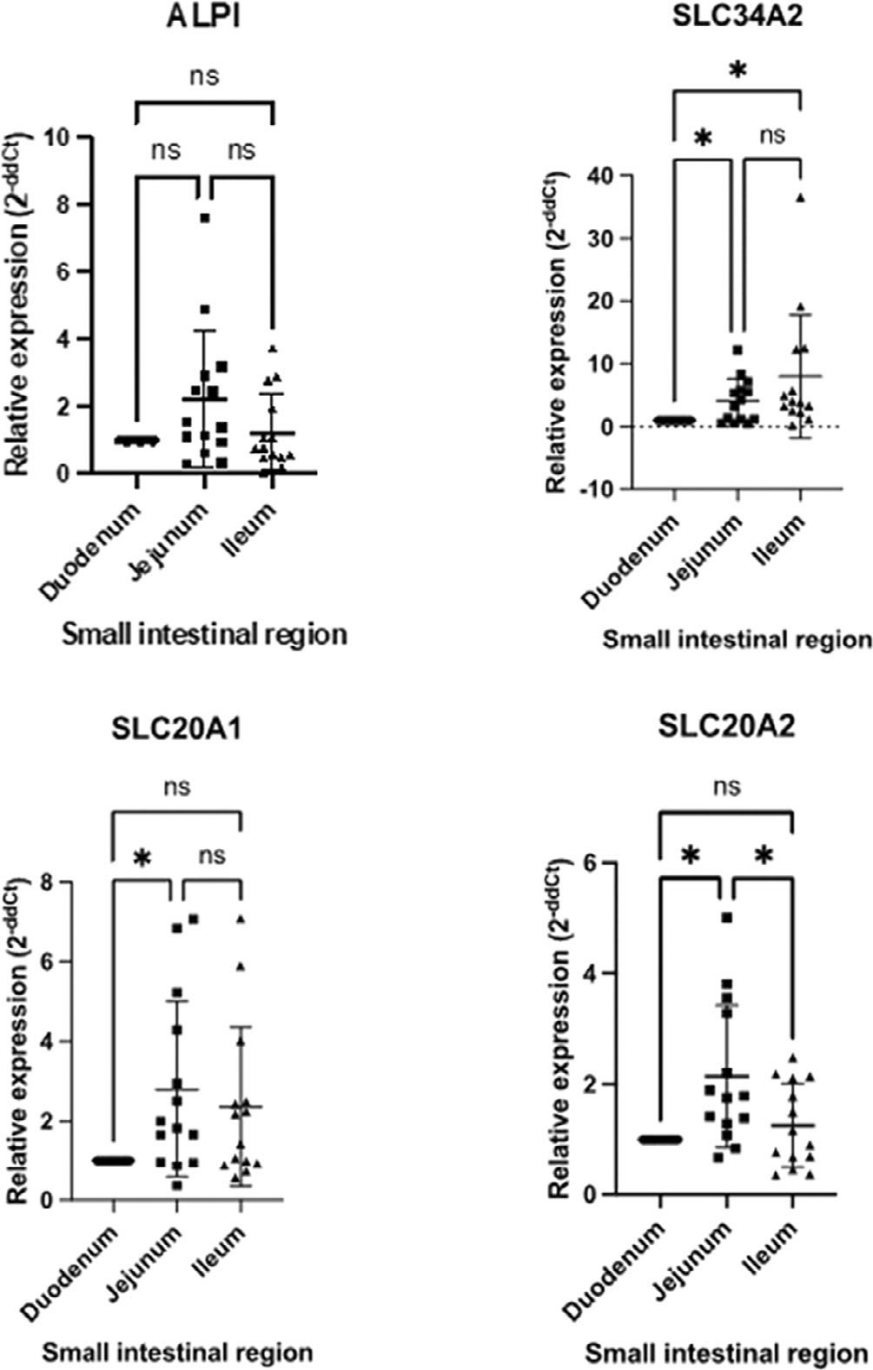
**Figure 1.** Plots showing relative gene expression of ALPI (A), SLC34A2 (B), SLC20A1 (C) and SLC20A2 (D) in the different regions of the feline small intestine. Relative gene expression calculated by the delta‐delta Ct method, using ribosomal protein S7 as a reference gene, and duodenal samples as ‘calibrator samples’ to which relative gene expression was expressed. Horizontal lines represent mean values and whiskers ±1 SD. Asterisks mark statistical significance (ns = *P* > .05, * = *P* ≤ .05; one‐way repeated measures ANOVA and post hoc analysis using Tukey's Test).

## ABSTRACT NU09: Evaluation of the circulating renin‐angiotensin‐aldosterone system in cats with surgically induced chronic kidney disease

### **Jane HC Huang**; Bianca Lourenço; Chad Schmiedt; Amanda Coleman

#### ^1^University of Georgia, Athens, GA, USA

**Background:** The renin‐angiotensin‐aldosterone system (RAAS) plays a key role in chronic kidney disease (CKD) in multiple species; however, little is known about this system in cats with CKD.

**Objectives:** To describe the classic and alternative circulating RAAS pathway components in cats with CKD, and to evaluate their correlation with CKD progression and amlodipine administration.

**Animals:** 15 cats with surgically induced CKD and 15 healthy cats with comparable age and sex ratios.

**Methods:** Observational case‐control study. Serum equilibrium concentrations of angiotensin I, II, III, IV, 1‐5, and 1‐7, and aldosterone were evaluated using liquid chromatography‐mass spectrometry twice (6 months apart) for CKD cats and once for healthy cats. Data were analyzed using linear mixed models or Mann‐Whitney U test, when appropriate. CKD progression was defined as >25% increase in serum concentrations of creatinine, symmetric dimethylarginine, or both within a 10‐month follow‐up period.

**Results:** Relative to healthy cats, CKD cats had significantly higher serum aldosterone concentrations (median, 205.3 vs. 399.1 PMol/L; *P*‐value <.01). No differences in angiotensin peptide concentrations were found. Among CKD cats, amlodipine‐treated cats had higher concentrations of all angiotensins and aldosterone. In CKD cats with follow‐up data available, those who experienced progression (n = 3) had lower baseline aldosterone concentrations compared to those who did not (n = 9) (median 162.2 vs. 430.4 pmol/L; *P*‐value = .021).

**Conclusions and Clinical Importance:** Amlodipine administration was associated with activation of the circulating classic and alternative RAAS pathways. Further study is warranted to clarify the correlation between circulating aldosterone and CKD progression in cats.

**Figure:**
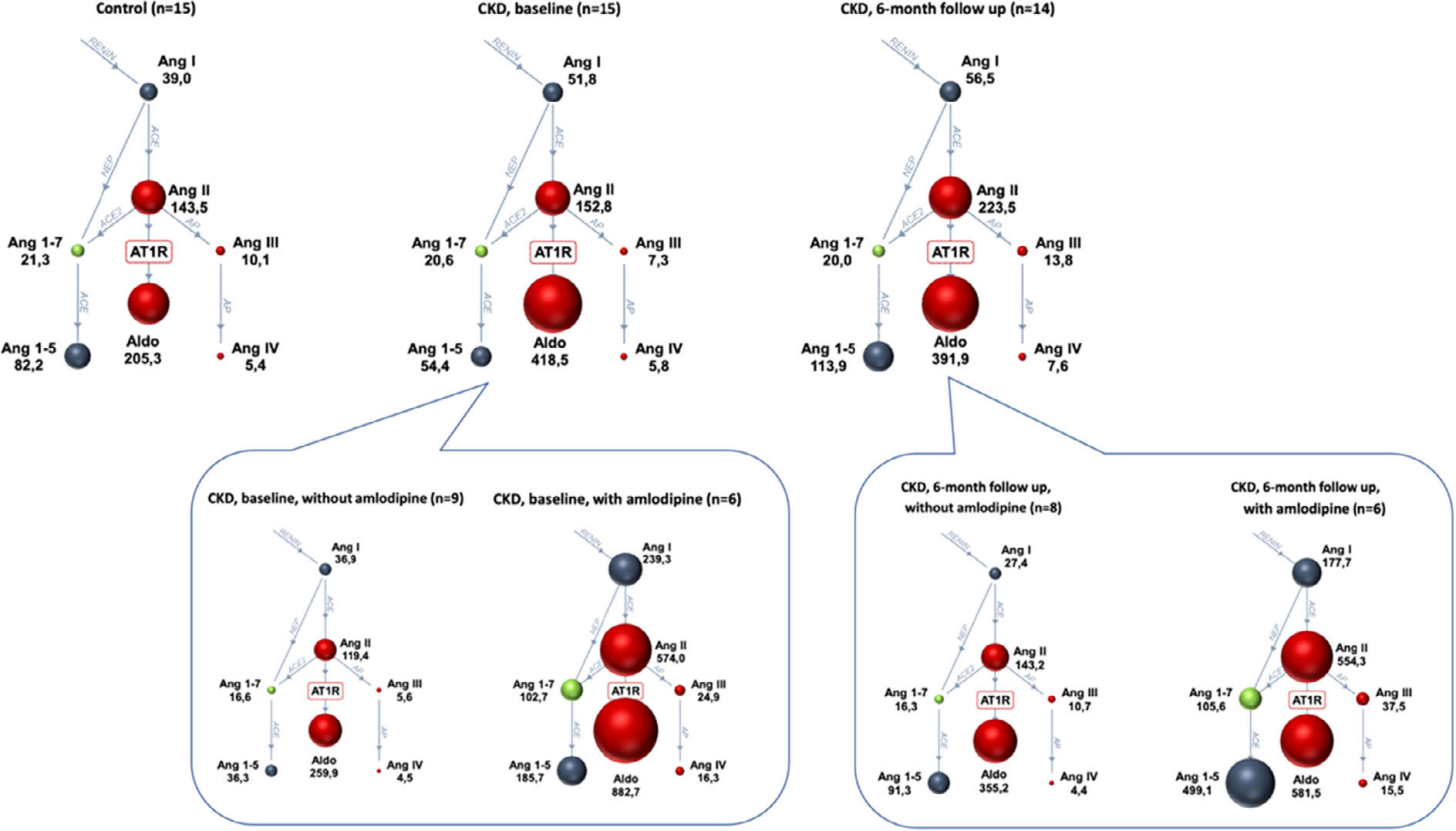
**Figure 1.** Median concentrations of measured circulating renin‐angiotensin‐aldosterone (RAAS) components (pmol/L) in cats with surgically induced chronic kidney disease (CKD) and in healthy cats of comparable age and sex ratio. Cats with CKD previously diagnosed with systemic arterial hypertension had been receiving treatment with amlodipine (0.625‐2.5 mg/cat PO q24 h) chronically. ACE—angiotensin‐converting enzyme, ACE2—angiotensin‐converting enzyme 2, Aldo—aldosterone, Ang—angiotensin, AP—aminopeptidase, AT1R—angiotensin II type 1 receptor, NEP—neutral endopeptidase (neprilysin).

## ABSTRACT NU10: Urine protein electrophoresis in dogs with systemic inflammatory response syndrome

### **Rankyung Jung** ^1^; Hyeona Bae^2^; DoHyeon Yu^2^

#### ^1^Animal Medical Center, Gyeonsang National University, Gyeongsangnam‐do, South Korea; ^2^Professor, Animal Medical Center, Gyeongsang National University, Gyeongsangnam‐do, South Korea

**ABSTRACT**

Sulfate‐polyacrylamide gel electrophoresis (SDS‐PAGE) is an objective method for qualitative protein detection. The objective of this study was to utilize SDS‐PAGE in the analysis of proteinuria in canine systemic inflammatory response syndrome (SIRS) and to evaluate its potential as a prognostic indicator by comparison with clinicopathologic parameters and acute patient physiologic and laboratory evaluation scores (APPLEfast and APPLEfull). Thirty‐one dogs with renal proteinuria were categorized into two groups: SIRS (n = 10) and non‐SIRS (n = 21), and electropherograms were generated following urine SDS‐PAGE. The molecules were classified into low‐molecular‐weight molecules (LMW) (The LMW to HMW ratio (LHR) was statistically higher in the SIRS group compared to the non‐SIRS group (*P* < .001). Canine SIRS may induce renal tubular damage. In SIRS dogs, proteinuria can be an early indicator of kidney damage, and an increase in tubular damage could indicate a negative prognostic factor in dogs with proteinuria.

## ABSTRACT NU11: Osteogenic phenotype in feline renal mineralization: Implications for kidney stone prevention

### **Nuttha Hengtrakul**; Eva Furrow; Michael Borofsky; Ferenc Toth; Jody Lulich

#### University of Minnesota, Falcon Heights, MN, USA

**Background:** Feline nephroliths pose an imminent threat of ureteral obstruction and acute kidney injury. Some nephroliths form over papillary mineralization. Papillary mineral that is exposed to the urinary space provides a preformed surface for heterogeneous nucleation at levels below oversaturation. This mechanism of stone formation might not be effectively prevented by strategies that reduce urine saturation. Understanding the causes of renal parenchymal mineralization might unveil new treatment targets to prevent stone formation and preserve renal function.

**Hypothesis/Objectives:** Besides oversaturation of calcium salts, feline renal mineralization is associated with expression of an osteogenic phenotype.

**Samples:** Eighteen kidneys with medullary mineralization (10 with and 8 without nephroliths) were obtained from 18 cats undergoing necropsy.

**Methods:** To determine if osteogenic proteins co‐localize with papillary mineralization, tissue sections were examined using immunohistochemistry for five osteogenic markers (osteopontin, osteocalcin, bone morphogenic protein‐2, runt‐related transcription factor‐2, and tissue non‐specific alkaline phosphatase) and minerals (Von Kossa).

**Results:** Osteopontin and osteocalcin co‐localized with mineral deposits in 18 and 12 kidneys, respectively. Co‐localization of other osteogenic proteins and minerals were found in two or fewer kidneys. There were no differences in co‐localization of osteogenic protein expression and mineralization between the kidneys with and without nephroliths.

**Conclusions and Clinical Importance:** These results provide evidence that cell‐mediated osteogenic‐like processes might contribute to renal mineralization in cats. Targeting these processes could offer a new approach to prevent kidney stone formation at its origin.

**Table:** **Table 1**. Co‐localization of papillary mineralization and osteogenic proteins in feline kidneys with and without nephroliths

Osteogenic proteins	Nephrolith (# Positive/Total)	No nephrolith (# Positive/Total)	Total	*P*‐value*
Osteopontin	10/10	8/8	18/18	1
Osteocalcin	5/10	7/8	12/18	0.15
Bone morphogenic protein‐2	2/10	0/8	2/18	0.48
Tissue non‐specific‐alkaline phosphatase	0/10	1/8	1/18	0.44
Runt‐related transcription factor‐2	0/10	0/8	0/18	1

## ABSTRACT NU12: Investigation of a novel low‐dose sedation protocol for canine urodynamic studies

### **Susan V. Carr** ^1^; McKenna Willis^2^; Michael Lappin^3^, DVM, PhD, DACVIM

#### ^1^Colorado State University, Fort Collins, CO, USA; ^2^Research Associate, Colorado State University, Fort Collins, CO, USA; ^3^Professor, Department of Clinical Sciences, Colorado State University, Fort Collins, CO, USA

**Background:** Urodynamics including cystometrogram (CMG) and urethral pressure profile (UPP) should accurately represent the function of the bladder and urethra. Patient cooperation is required for urinary catheter placement and accurate pressure recordings. Any sedation protocol should have minimal effect on urodynamic variables. Previous studies by Rawlings (2001), Richter (1985), and Byron (2003) highlight some of these issues.

**Hypotheses:** Low‐dose propofol bolus will allow urinary catheterization. The detrusor reflex will be maintained, and CMGs will be comparable between repetitions. The protocol will permit cooperation for a satisfactory UPP to be recorded.

**Animals:** Six healthy four‐year‐old female sexually intact research colony beagles.

**Methods:** A prospective interventional study recording urodynamics in each dog after low‐dose propofol, then repeated after a five‐week interval. 2 mg/kg propofol was administered intravenously and urinary catheterization attempted. Recovery from sedation, defined as the ability to maintain sternal recumbency and at least 10 minutes from drug administration, was required before commencing CMG. The bladder was filled until either a detrusor reflex or an intravesical pressure of 40 cm H_2_O was achieved. This was followed by UPP measurements.

**Results:** Successful urinary catheterization and sufficient cooperation in all dogs was achieved. Detrusor reflex activity was recorded in 2/12 CMG procedures. Threshold volume varied. A satisfactory UPP tracing was recorded in 5/12 procedures.

**Conclusions and Clinical Importance:** Although low‐dose protocol produced satisfactory patient cooperation, maintenance of a detrusor reflex was unreliable and urodynamic measurements were variable, which may impact clinical assessment of bladder and urethral function.

## ABSTRACT NU13: Phosphate kinetics during intermittent hemodialysis in dogs with acute kidney injury

### **Carrie A. Palm**; Larry Cowgill; Luke Wittenburg

#### School of Veterinary Medicine, University of California‐Davis, Davis, CA, USA

**Background:** Intermittent hemodialysis (IHD) is used for management of dogs with high grade acute kidney injury (AKI). Phosphate removal is a critical part of IHD in these dogs, but kinetics have not been described.

**Objectives:** To describe plasma phosphate kinetics in dogs receiving IHD for AKI.

**Animals:** 7 client‐owned dogs.

**Methods:** Dogs undergoing IHD for AKI were enrolled during their first 2 IHD treatments with client consent. Plasma creatinine, urea, and phosphate concentrations were measured before IHD treatment, serially during the treatment, and 2 hours post‐treatment. Descriptive statistics were performed.

**Results:** Median IHD treatment time was 300 minutes (interquartile range [IQR], 300‐346 minutes). For treatment 1, the median urea reduction ratio (URR) was 48% (IQR, 47‐55%). The pre‐ and post‐treatment median plasma phosphate concentration was 17.7 mg/dL (IQR, 15.2‐19.2 mg/dL) and 10.3 mg/dL (IQR, 9.3‐11.1 mg/dL), respectively, representing a phosphate reduction of 40% (IQR, 38‐42%). Two hours post‐treatment, median plasma phosphate concentration was 10.7 mg/dL (7.9‐13.7 mg/dL). For treatment 2, the median URR was 75% (IQR, 75‐77%). Median plasma phosphate concentration was 12.7 mg/dL (IQR, 12.2‐15.0 mg/dL) and 4.2 mg/dL (IQR, 3.6‐4.7 mg/dL) pre‐ and post‐treatment, respectively. Plasma phosphate concentrations decreased by 69% (IQR, 51‐72%). The median plasma phosphate concentration was 5.5 mg/dL 2 hours post‐treatment (IQR, 5.3‐6.3 mg/dL).

**Conclusions and Clinical Importance:** In dogs with AKI undergoing IHD, plasma phosphate concentrations decrease, then rebound soon after treatment cessation. This preliminary evaluation of phosphate kinetics in dogs undergoing IHD may help guide dialysis prescription as it relates to phosphate management.

## ABSTRACT NU14: Serum lipidomic and metabolomic profiling in miniature schnauzer dogs with and without calcium oxalate urolithiasis

### **Emily Coffey** ^1^; Andres Gomez^1^; Lauren Baker^2^; Nicole Tate^1^; Jody Lulich^1^; Eva Furrow^2^

#### ^1^University of Minnesota, White Bear Lake, MN, USA; ^2^University of Wisconsin, Madison, WI, USA

**Background:** Calcium oxalate urolithiasis is associated with various metabolic disorders, including dyslipidemia, in humans and dogs. Improved understanding of the underlying metabolic derangements is necessary to advance urolithiasis management strategies.

**Hypothesis/Objectives:** The objective of this study was to identify differences in serum lipidomic and metabolomic profiles between Miniature Schnauzers with and without calcium oxalate urolithiasis.

**Animals:** Miniature Schnauzers with (n = 15, cases) and without (n = 27, controls) a history of calcium oxalate urolithiasis.

**Methods:** Serum lipidomics and untargeted metabolomics were performed using ultra‐high performance liquid chromatography/tandem mass spectroscopy. Normalized lipid species and metabolites were compared between cases and controls using Student's *t*‐tests with correction for multiple comparisons. Metabolites with biological links to calcium oxalate urolithiasis were designated as “high priority” and further analyzed with clustering.

**Results:** None of 956 lipid species were identified as different between cases and controls. Three of 802 metabolites were lower in cases: 10‐undecenoate, N‐delta‐acetylornithine, and glutarate (Praw < .001 and *q* < .10 for all). Cluster analysis of high priority metabolites identified five cases with a distinct pattern, characterized by lower citrate and higher phosphate, glycine, and hippurate.

**Conclusions and Clinical Importance:** The absence of differentiating lipid species between cases and controls does not support dyslipidemia as a major risk factor for calcium oxalate urolithiasis in Miniature Schnauzers. The metabolites that discriminated between cases and controls are linked to nutrition and might reflect dietary differences. Distinct metabolic subsets of stone formers might exist within the breed.

## ABSTRACT NU15: Immune complex‐mediated glomerulonephropathy in Australian and New Zealand dogs

### **Joanna White**, BVSc, MVSc (Epi), DAVCIM (SAIM), PhD

#### Small Animal Specialist Hospital, North Ryde, NSW, Australia

**Background:** The prevalence of immune complex‐mediated glomerulonephropathy (ICGN) in dogs with proteinuric kidney disease is approximately 50% in the United States and Europe. In Australia and New Zealand, the prevalence of ICGN in these dogs is unknown.

**Objectives:** To determine the prevalence of ICGN in dogs biopsied for proteinuric kidney disease in Australia and New Zealand.

**Animals:** 50 client‐owned dogs.

**Methods:** Retrospective case series. Reports from renal biopsies submitted to the Texas and International Veterinary Renal Pathology Services from dogs in Australia and New Zealand between 2007 and 2023 were reviewed. Dogs were included if the biopsy was performed for investigation of proteinuric kidney disease where the urine protein to creatinine ratio (UPCR) was >1.0. Clinical data were reviewed and descriptive statistics created.

**Results:** Among 50 dogs (median age, 6 years; interquartile range [IQR], 4‐10 years) with proteinuric renal disease (median UPCR, 4.1; IQR, 2.8‐9.0; median serum creatinine concentration, 2.0 mg/dL; IQR, 1.1‐3.4 mg/dL), 15 dogs (30%) had ICGN and 35 (70%) had non‐ICGN. The most common category of ICGN was membranoproliferative glomerulonephritis (5/15; 33%). Glomerulosclerosis was the most common category of non‐ICGN (17/35; 49%).

**Conclusions and Clinical Importance:** The prevalence of ICGN is lower in Australian and New Zealand dogs biopsied for proteinuric kidney disease compared to the United States and Europe, potentially due to lower prevalence of infectious disease such as tickborne disease. The lower prevalence of ICGN highlights the importance of renal biopsy in these dogs to optimize treatment.

## ABSTRACT NU16: Vitamin D metabolite profiles in cats with chronic kidney disease compared to healthy cats

### **Lauren Reynolds** ^1^; Jessica Quimby^2^, DVM, PhD, DACVIM (SAIM); Valerie Parker^3^, DVM, DACVIM (SAIM, Nutrition)

#### ^1^Veterinary Medical Center, The Ohio State University, Columbus, OH, USA; ^2^Professor, Department of Clinical Sciences, The Ohio State University, Columbus, OH, USA; ^3^Professor, Clinical, Department of Clinical Sciences, The Ohio State University, Columbus, OH, USA

**Background:** Chronic kidney disease–mineral and bone disorder (CKD‐MBD) is characterized in part by vitamin D dysregulation, yet comprehensive vitamin D metabolite profiles have yet to be elucidated in cats with CKD. Evidence‐based recommendations for vitamin D supplementation are lacking.

**Hypothesis/Objectives:** The primary objective was to investigate comprehensive vitamin D metabolite profiles (25‐hydroxyvitamin D3 [25D], 1,25‐dihydroxyvitamin D3 [1,25D], D, 24,25‐dihydroxyvitamin D3 [24,25D], and 3‐epi‐25‐hydroxyvitamin D3 [3‐epi‐25D]) in cats with CKD compared to healthy controls. We hypothesized that all vitamin D metabolites would be lower in cats with CKD compared to healthy cats and that vitamin D metabolites would be negatively correlated with serum creatinine.

**Animals:** Client‐owned cats with naturally occurring CKD (n = 42) and healthy cats from a research colony (n = 12) were included.

**Methods:** Retrospective cohort study. All serum vitamin D metabolites were measured via liquid chromatography‐mass spectrometry (LC‐MS/MS). Data were analyzed using a Mann‐Whitney *U* test and Spearman correlation.

**Results:** Median (range) concentrations for 25D, 1,25D, 24,25D, and 3‐epi‐25D in cats with CKD were 30.0 ng/mL (12.2‐68.8 ng/mL), 136.8 pg/mL (38.6‐367.8 pg/mL), 10.9 ng/mL (2.7‐32.5 ng/mL), and 9.1 ng/mL (2.2‐30.4 ng/mL), respectively. Compared to healthy cats, all vitamin D metabolites were significantly higher in cats with CKD (*P* < .05; Table 1). A weak positive correlation was found between creatinine and 25D, 24,25D, and 3‐epi‐25D (r: 0.28‐0.32; *P* < .05).

**Conclusions and Clinical Relevance:** These results do not mirror what has been demonstrated in people or dogs with CKD‐MBD. This study does not support the utility of calcitriol supplementation in cats with CKD.

**Table:** **Table 1.** Median (range) concentrations of serum vitamin D metabolites in cats with chronic kidney disease (CKD) and healthy cats

Vitamin D metabolites	Cats with CKD (n = 42)	Healthy cats (n = 12)	*P* value
25‐hydroxyvitamin D_3_ (ng/mL)	30.0 (12.2–68.8)	23.5 (14.8‐35.9)	.025
1,25‐dihydroxyvitamin D_3_ (pg/mL)	136.8 (38.6–367.8)	88.2 (71.5‐154.9)	.044
24,25‐dihydroxyvitamin D_3_ (ng/mL)	10.9 (2.7–32.5)	5.35 (1.2‐15.9)	.006
3‐epi‐25‐hydroxyvitamin D_3_ (ng/mL)	9.1 (2.2–30.4)	6.9 (4.4‐9.1)	.014

## ABSTRACT NU17: The effect of porus® one on uremic toxin concentrations in cats with chronic kidney disease

### **Rene E. Paschall** ^1^; Jessica Quimby^1^, DVM, PhD, DACVIM (SAIM); Bianca Lourenco^2^, DVM, MSc, PhD, DACVIM; Stacie Summers^3^, DVM, PhD, DACVIM (SAIM); Chad Schmiedt^2^, DVM, DACVS

#### ^1^The Ohio State University, Columbus, OH, USA; ^2^University of Georgia, Athens, GA, USA; ^3^Oregon State University, Corvallis, OR, USA

**Background:** Serum uremic toxin concentrations (UT) markedly increase in cats with chronic kidney disease (CKD) and can have deleterious consequences. Porus One is an oral adsorbent that binds UT precursors in the gut.

**Objectives/Hypothesis:** Assess the effect of Porus One on UT in cats with CKD.

**Animals:** 14 purpose‐bred cats (13 IRIS Stage 2, 1 IRIS Stage 3).

**Methods:** Prospective cross‐sectional study utilizing cats with remnant kidney model‐induced CKD. Cats were treated with standard of care (Days ‐56 to 0) and then 500 mg Porus One PO q24h (Days 0 to 56) in food. Serum concentrations of indoxyl sulfate (IDS) and p‐cresyl sulfate (pCS) were measured (Days ‐56, ‐28, 0, 28, and 56) by LC/MS/MS. Doppler blood pressure (BP), complete blood cell count, biochemistry, urinalysis, and urine protein: creatinine ratio were measured (Days ‐56, 0, and 56). UT and clinicopathologic variables were compared before and after administration of Porus One.

**Results:** Averaged serum IDS and pCS concentrations were significantly decreased after administering Porus One relative to averaged baseline concentrations (Table 1). Concentrations of ICS and pCS at Day 28 (but not Day 56) significantly decreased relative to Day 0. BP significantly decreased (*P* = .03), and serum bicarbonate concentrations significantly increased (*P* = .03) after administering Porus One. Serum creatinine concentrations were significantly increased at Day 56 relative to Day ‐56 (*P* = .002) but not relative to Day 0.

(VIN editor: Table not provided.)

**Conclusions and Clinical Importance:** Porus One decreased UT despite mild progression of disease.

## ABSTRACT NU18: Evaluation of the effect of bilirubinuria on urine dipstick results

### **Amanda Alli** ^1^; Xiaojuan Zhu^1^, PhD; Sarah Schmid^2^, DVM, DACVIM (SAIM), Assistant Professor

#### ^1^University of Tennessee, Knoxville, TN, USA; ^2^Department of Small Animal Clinical Sciences, University of Tennessee, Knoxville, TN, USA

**Background:** Dry reagent strips (urine dipsticks, UDS) are an essential component to a complete urinalysis. Despite UDS manufacturers warning that pigmenturia can influence results, the effect of bilirubinuria is unknown.

**Objectives:** Determine the effect of bilirubinuria on UDS analytes and urine specific gravity (USG).

**Samples:** Thirteen urine samples without pigmenturia from client‐owned dogs. Eight pooled residual clinical samples submitted for a urinalysis and four pooled samples from healthy dogs.

**Methods:** Urine aliquots were spiked with a bilirubin standard (bilirubin conjugate disodium salt [Sigma Aldrich] in water; SG 1.00) to obtain concentrations of 0, 0.5, 1, 2, 4, 8, and 20 mg/dl. Visual and automated (Siemens CLINITEK Status) UDS evaluations were performed. Agreement between visual and automated readings was compared with interclass correlation coefficients (ICC). Correlation between urine bilirubin concentration and UDS analyte readings were evaluated using Spearman's rank correlation.

**Results:** Agreement between visual and automated UDS results were excellent for bilirubin (ICC = 0.946) and blood (ICC = 0.914), good for pH (ICC = 0.854) and protein (ICC = 0.886), moderate for glucose (ICC = 0.550), and poor for ketones (ICC < 0.001).

**Conclusions/Clinical Importance:** Bilirubinuria is unlikely to alter interpretation of UDS analytes in a clinical setting.

## ABSTRACT NU19: Analytical validation of an assay for measurement of uremic toxins in dog and cat serum

### **Amanda B. Blake** ^1^; Stacie Summers^2^; Jessica Quimby^3^; Joerg Steiner^1^; Jonathan Lidbury^1^; Jan Suchodolski^1^

#### ^1^Gastrointestinal Lab, Texas A&M University, College Station, TX, USA; ^2^Oregon State University, Corvallis, OR, USA; ^3^The Ohio State University, Columbus, OH, USA

**Background:** Indoxyl sulfate (IS) and p‐cresol sulfate (PCS) are uremic toxins produced by bacterial breakdown of tryptophan and tyrosine in the gastrointestinal tract followed by sulfotransferase conversions in the liver and gut mucosa. They have been associated with chronic kidney disease (CKD) severity and progression in dogs and cats.

**Objective:** Analytically validate an assay for quantitation of IS and PCS in canine and feline serum.

**Animals:** Excess serum samples submitted for routine diagnostic evaluation from dogs and cats, and serum from CKD cats, were used.

**Methods:** The liquid chromatography tandem mass spectrometry assay was validated according to FDA guidelines for bioanalytical method validation. Isotopically labeled internal standards were utilized for each analyte.

**Results:** The working range of the assay for IS and PCS was 1‐40 000 ng/ml for both, covering the common biological range in healthy and CKD animals. The assay was precise and accurate (CV% < 12%). Extracted samples were stable on reinjection up to 72 hours (CV% < 10.2%). As little as 10 μl of serum was able to be used with accurate quantitation. Dilution and spiking recovery had observed to expected ratios between 80% and 120% apart from one low spike sample at 130%. Lipemia did not affect measurement in canine serum.

**Conclusions and Clinical Importance:** The validated assay provides a wide working range. The low sample volume requirement of 10 μl increases the feasibility of routine IS and PCS measurement in dogs and cats with CKD.

## ABSTRACT NU20: Characterization of cardiac disease in cats with ureteral obstruction undergoing subcutaneous ureteral bypass device placement

### **Claire Cassou** ^1^; Christina Plante^2^, DVM, DACVIM (Cardiology); Catherine Vachon^3^, DVM, DVSc, DACVIM, Fellow IR; Marilyn Dunn^3^, DVM, MVSc, DACVIM, Fellow IR

#### ^1^Centre Hospitalier Universitaire Universitaire, Faculté de Médecine Vétérinaire de Saint‐Hyacinthe, Saint‐Hyacinthe, QC, Canada; ^2^Clinician Teacher, Cardiology CHUV Saint‐Hyacinthe, University of Montreal, Saint‐Hyacinthe, QC, Canada; ^3^Clinician Teacher, Interventional Medicine, CHUV Saint‐Hyacinthe, University of Montreal, Saint‐Hyacinthe, QC, Canada

**Background:** Little information exists on the prevalence and outcomes of cardiac disease (CD) in cats presented with benign feline ureteral obstruction (FUO) treated by SUB device placement.

**Objectives:** Characterize CD, evaluate perioperative and long‐term outcomes, and determine the ability of echocardiography to predict risk of fluid overload (FO) in cats presented for SUB placement.

**Animals:** 104 cats presented for SUB placement between 2010 and 2022 at the CHUV.

**Methods:** Retrospective evaluation of medical records included signalment, examination, FO, survival time, serum creatinine pre‐operatively and postoperatively, and echocardiographic variables. Cats were classified as: no echocardiogram [NE], presence of CD [ED+] or absence of CD [ED‐].

**Results:** Echocardiography was performed in 71% of cats and prevalence of CD was 46%. Mean survival time of ED+ was significantly shorter (*P* = .015) than NE. ED‐ had a significantly greater decrease in serum creatinine from admission to discharge, and 1 month postoperatively compared to ED+ or NE. FO occurred in 37% of cats and survival time was significantly shorter than cats without FO (739 ± 250 days vs. 1106 ± 213 days; *P* = .024). There was no correlation between LA/Ao ratio nor LAmax value and the development of FO (*P* = .151 and *P* = .710), nor survival time (*P* =.393 and *P* = .065).

**Conclusions and Clinical Importance:** CD is a common comorbidity and may negatively influence postoperative recovery of renal function and survival in cats presented for SUB placement. Measurement of LA/Ao ratio and LAmax values were not predictive of FO.

## ABSTRACT NU21: Comparing defecation frequency between cats with and without chronic kidney disease

### **Zachary M. George** ^1^; Jessica Quimby^2^, DVM, DACVIM (SAIM), PhD; Katelyn Brusach^3^, BS, PhD Candidate; Lina Lim^4^, DVM; Sarah Jones^5^, DVM; Adam Rudinsky^6^, DVM, DACVIM (SAIM)

#### ^1^College of Veterinary Medicine, The Ohio State University, Columbus, OH, USA; ^2^Professor, Department of Veterinary Clinical Sciences, College of Veterinary Medicine, The Ohio State University, Columbus, OH, USA; ^3^Department of Veterinary Clinical Sciences, College of Veterinary Medicine, The Ohio State University, Columbus, OH, USA; ^4^Small Animal Internal Medicine Research Inten, Department of Veterinary Clinical Sciences, College of Veterinary Medicine, The Ohio State University, Columbus, OH, USA; ^5^Small Animal Internal Medicine Research Inten, Department of Veterinary Clinical Sciences, College of Veterinary Medicine, The Ohio State University, Columbus, OH, USA; ^6^Associate Professor, Department of Veterinary Clinical Sciences, College of Veterinary Medicine, The Ohio State University, Columbus, OH, USA

**Background:** Cats with chronic kidney disease (CKD) are at higher risk for presenting with constipation.

**Hypothesis/Objectives:** The purpose of this study was to objectively measure defecation frequency in cats with and without CKD. It was hypothesized that cats with CKD would defecate less frequently than healthy cats.

**Animals:** Cats with stable IRIS stages 2‐4 CKD (n = 9) and healthy control cats (n = 9) with no known history of overt constipation.

**Methods:** Prospective observational study. Serum biochemistry, CBC, T4, urinalysis, and blood pressure was performed in all cats to confirm health status. The Petivity Smart Litter Box Monitor System was utilized to collect real‐time defecation data. Data was collected via the Petivity app for 30 days during which medical management and husbandry remained consistent. A Mann‐Whitney *U* test was performed to compare the total number of days without defecation between CKD cats and healthy cats over 14, 21, and 30 days.

**Results:** CKD cats had significantly more total days without defecation than healthy cats at 14 (*P* = .001), 21 (*P* = .0004), and 30 days (*P* = .0003). Median total days without defecation across 14, 21, and 30 days was 5 (1‐8), 7 (3‐9), and 10 (5‐13), respectively, for CKD cats compared to 0 (0‐4), 2 (0‐4), and 2 (0‐7), for healthy cats.

**Conclusions and Clinical Importance:** Cats with CKD defecate less than healthy cats which may be an indicator of subclinical constipation. This information could allow for earlier interventions and therapies, possibly preventing episodes of overt constipation.

## ABSTRACT NU22: The effect of feeding on urine ammonia levels in cats with and without CKD

### **Anna Panyutin** ^1^; Autumn Harris^2^, DVM, DACVIM; Lina Lim^3^, DVM; Jessica Quimby^4^, DVM, PhD, DACVIM; Ashlie Saffire^5^, DVM, DABVP

#### ^1^The Ohio State University Veterinary Medical Center, Columbus, OH, USA; ^2^Department of Small Animal Clinical Sciences, College of Veterinary Medicine; ^3^Internal Medicine Specialty and Research Intern, Veterinary Medical Center Internal Medicine Service, The Ohio State University, Columbus, OH, USA; ^4^Veterinary Medical Center Internal Medicine Service, The Ohio State University, Columbus, OH, USA; ^5^Faithful Friends Veterinary Clinic, Dublin, OH, USA

**Background:** Impaired ammonia excretion plays a role in the pathogenesis of metabolic acidosis in CKD, and may have prognostic value. Gold standard evaluation involves measurement of ammonia excretion as urine ammonia‐to‐creatinine ratio (UACR). The influence of eating on UACR is unknown.

**Hypothesis/Objectives:** To investigate the effect of fasting vs. feeding on urine ammonia levels in cats with and without CKD. We hypothesized that UACR would increase after feeding and this effect would be blunted in cats with CKD.

**Animals:** Cats with stable IRIS stages 1‐4 CKD (n = 13) and healthy cats (n = 10).

**Methods:** Randomized, prospective cross‐over study. CBC, serum biochemistry, T4, and urinalysis were performed in all cats to confirm health status. Urine was collected at two visits approximately 1 week apart; once 2‐3 hours post meal, and once fasted. Urinary ammonia and creatinine concentrations were measured using commercially available assays and used to calculate UACR.

**Results:** UACR was significantly lower in fed (median = 0.77, range 0.11‐6.59) versus fasted state (median = 2.55, range 0.49‐9.58) in CKD cats (*P* = .01). There was no difference in control cats (fed median = 4.305, range 0.19‐11.54; fasted median 4.98, range 0.65‐10.81) (*P* = .49). There was a significant negative correlation between serum creatinine and UACR in both the fed (r = −0.84; *P* = .0006) and fasted state (r = −0.92; *P* < .0001).

**Conclusions and Clinical Importance:** Prandial state affects UACR in cats with CKD and should be kept consistent in longitudinal sampling.

## ABSTRACT NU23: Comparison of urine sample preparation methods in recovering urine sediment elements from canine/feline samples

### **Anonda Haskin**, DMV; Mary Lewis, MS, DVM, DACVP (Clinical); Eric Morissette, BSc, DVM, DACVP (Clinical); Kristin Owens, DVM, DACVP (Clinical); Cory Penn, DVM

#### Zoetis

**Background:** In‐clinic analysis of warm, fresh urine provides rapid results of urine sediment findings. A simple, standardized preparation method would aid in performing urine sediments in‐clinic.

**Hypothesis/Objectives:** The Vetscan Imagyst preparation method (VSI‐prep) will recover similar numbers of red blood cells, white blood cells, struvite crystals, calcium oxalate dihydrate crystals, squamous epithelial cells, other epithelial cells, hyaline casts, non‐hyaline casts, and rod and cocci bacteria as determined by manual microscopic examination by ACVP‐boarded clinical pathologists (ACVP‐CPs) as compared to reference lab preparation method (RL‐prep).

**Animals:** No animals were used in this study. Urine samples included a mix of client‐owned dogs and cats undergoing urinalysis for any reason submitted to Zoetis Reference Laboratories. Some samples were artificially created by spiking donor urine with necessary elements (eg, RBC from whole blood). A total of 213 paired urine samples consisting of 116 canine (54.5%) and 97 feline (45.5%) were evaluated.

**Methods:** Two 1 mL aliquots were made from each urine sample, processed via VSI‐prep or RL‐prep, and blindly evaluated via manual microscopy by the same 2 ACVP‐CPs. RL‐prep served as the gold standard for comparison. ACVP‐CPs recorded results for urine elements as an average of 10, 40X fields. Percent semi‐quantification class agreement was calculated between methods and evaluated for each ACVP‐CP. Species were combined for analysis.

**Results:** For elements, aggregate % agreement ranged between 76.6% and 88.9% (Table 1). Individual % agreements varied (Table 1).

**Conclusions and Clinical Importance:** VSI‐prep recovered similar numbers of urine sediment elements in canine and feline urine sediment samples as RL‐prep.

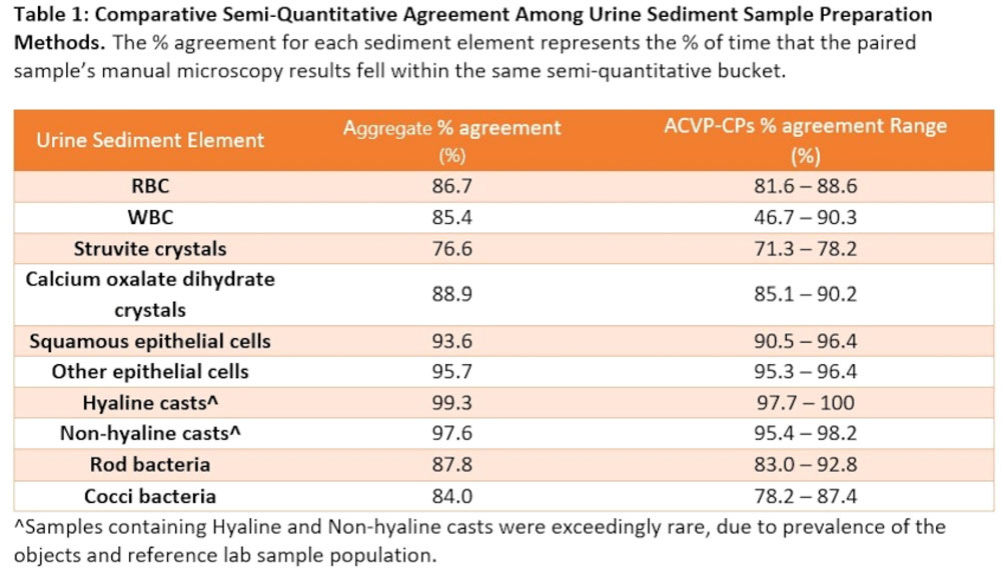

## ABSTRACT NU24: Pathophysiology of hyperammonemia from acute kidney injury or acute on chronic kidney disease in cats

### **Katelyn** **McFadden** ^1^ ; Mary Labato^2^; Deborah Linder^2^; Cynthia Webster^2^

#### ^1^Baton Rouge Veterinary Referral Center, Baton Rouge, LA, USA; ^2^Cummings School of Veterinary Medicine at Tufts University, North Grafton, MA, USA

**Background:** Hyperammonemia occurs in cats with kidney disease although the pathophysiology is poorly understood.

**Hypothesis/Objectives:** Hyperammonemic cats with acute kidney injury or acute on chronic kidney disease (AKI/AoCKD) will have decreased concentrations of plasma amino acids involved in the urea cycle, lower blood pH and decreased body (BCS) and muscle condition score (MSC) compared to cats with kidney disease without hyperammonemia.

**Animals:** Twenty cats presenting to a referral hospital with AKI/AoCKD were prospectively enrolled.

**Methods:** Cats were scored for BCS and MCS and a fasted blood sample was analyzed for blood ammonia, pH, BUN, creatinine, and phosphorus. Plasma was frozen at −80°C and shipped for amino acid analysis.

**Results:** Median age and weight were 12 yrs (3‐14 yrs) and 4.5 Kg (3‐5.6 kg), respectively. Median BCS and MCS were 5/9 (range 3‐7) and 2 (range 1‐3), respectively. Seven cats were hyperammonemic (311 uM±230 uM). Median creatinine, BUN and phosphorus were significantly higher in hyperammonemic cats and BCS (median 4/9) was significantly lower (*P* < 0.05). MCS and blood pH were not different between the 2 groups. Plasma citrulline (74±44 nmol/mL vs. 24±9 nmol/mL) and ornithine (21±6.7 nmol/mL vs. 13±7.7 nmol/mL) were significantly increased in hyperammonemic cats versus cats with normal blood ammonia, respectively (*P* < 0.05) while there was no difference in plasma arginine concentrations.

**Conclusions and Clinical Importance:** Hyperammonemia in cats with AKI/AoCKD is associated with an underweight BCS but not with acidosis, low MCS or a deficiency in key urea cycle amino acids. Increases in citrulline and ornithine may reflect decreased kidney function.

## ABSTRACT NU25: Deep learning artificial intelligence (AI) for rapid and reliable evaluation of canine/feline urine sediment samples

### **Cory D. Penn** ^1^, DVM; Mary Lewis^2^, MS, DVM, DACVP (Clinical); Anonda Haskin^2^, DVM; Eric Morissette^2^, BSc, DVM, DACVP (Clinical); Kristin Owens^2^, DVM, DACVP (Clinical)

#### ^1^Medical Affairs, Zoetis Global Diagnostics; ^2^Zoetis

**Background:** Urine sediment exam is vital to a complete urinalysis. A deep learning AI tool for in‐clinic, rapid, and consistent evaluation of urine sediment samples is currently lacking.

**Hypothesis/Objectives:** Vetscan Imagyst (VSI) AI Urine Sediment will accurately identify red blood cells, white blood cells, struvite crystals, calcium oxalate dihydrate crystals, and rod and cocci bacteria in agreement with digital review by ACVP‐boarded clinical pathologists (ACVP‐CPs).

**Animals:** No animals were used in this study. Urine samples included a mix of client‐owned dogs and cats undergoing urinalysis for any reason submitted to Zoetis Reference Laboratories. Some samples were artificially created by spiking donor urine with necessary elements (eg, RBC from whole blood). A total of 175 urine samples consisting of 98 canine (56%) and 77 feline (44%) were evaluated.

**Methods:** Samples were prepped via the VSI prep method, scanned, and evaluated digitally by 2 randomized, blinded ACVP‐CPs who recorded results for urine elements as an average of 10, 40X fields. Algorithm performance was calculated as compared to ACVP‐CP consensus, which served as gold standard.

**Results:** The VSI AI Urine Sediment algorithm reliably identified urine sediment elements. Sensitivity and specificity for urine sediment object classes ranged from 73% to 98% and 76% to 99%, respectively (Table 1). Combined bacteria identification had a PPV of 91%.

**Conclusions and Clinical Importance:** VSI AI Urine Sediment algorithm tested in this study was comparable to ACVP‐CPs in the identification of elements in urine sediment. In‐clinic utilization of VSI AI Urine Sediment can provide a diagnostic tool for urine sediment evaluation.

**Table:** **Table 1.** Algorithm performance. Sensitivity and specificity of Vetscan Imagyst AI dermatology v2.0 Algorithm as compared to ACVP‐CP consensus

Object class	Sensitivity	Specificity
Rod bacteria	83%	76%
Cocci bacteria	86%	82%
*Malassezia* incl. on keratinocyte	92%	87%
Neutrophils containing bacteria	77%	86%
Neutrophils normal & degenerate	95%	95%
Eosinophils	93%	94%
Lymphocytes	86%	85%
Macrophages	96%	84%

## ABSTRACT NU26: Quantification of serum leptin concentration in cats with and without chronic kidney disease

### **Jessica M. Quimby**; Katelyn Brusach, BS, PhD Candidate; Sarah Lorbach; Eline Nijveldt; Sarah Jones; Zach George; Rene Paschall; Adam Rudinsky; Ramiro Toribio

#### The Ohio State University, Columbus, OH, USA

**Background:** Leptin is a satiety‐inducing hormone secreted by adipose tissue and eliminated by the kidney. Renal dysfunction has been shown to increase serum leptin concentrations, which may cause appetite dysregulation. Studying appetite regulating hormones, such as leptin, will improve our understanding of inappetence in chronic kidney disease (CKD) cats.

**Objectives/Hypothesis:** To evaluate serum leptin concentrations in cats with and without CKD. We hypothesized that serum leptin concentrations will be significantly higher in cats with CKD compared to clinically normal cats.

**Animals:** 30 cats with stable CKD (IRIS Stages 1‐4) and 12 normal cats.

**Methods:** Serum biochemistry, CBC, T4, and urinalysis were performed to confirm health status and IRIS stage. Exclusion criteria included administration of an appetite stimulant (mirtazapine, gabapentin) within the past 24 hours, a body score condition >7/9, and diabetes, or other uncontrolled systemic disease. Fasted serum samples were collected and analyzed using a Rat Leptin ELISA Kit (RAB0335, Millipore) according to assay instructions. This kit was validated for feline use.

**Results:** Serum leptin concentrations were significantly higher in cats with CKD (median = 13.0 ng/mL, range = 0.104 ng/mL‐162 ng/mL) compared to clinically normal cats (median = 2.3 ng/mL, range = 0.065 ng/mL‐90.6 ng/mL) (*P* = 0.03). Serum leptin concentrations were not significantly correlated with serum creatinine (r = 0.246, *P* = .12).

**Conclusion:** Increased serum leptin concentrations in cats with CKD could result in appetite dysregulation and may contribute to weight loss and decreased appetite.

## ABSTRACT NU27: Effects of urine biochemical parameters in use of preservative collection tubes and preservative free tubes

### **Kayla Dunn** ^1^; Liz Mora^2^, BAS, RVT; Dennis Chmiel^3^, DVM, MBA; Tianna Crane^4^, RVT; Becky Gallant^5^, BS, CVT; Matthew Krecic^1^, DVM, MS, MBA, DACVIM

#### ^1^MySimplePetLab; ^2^Lead Lab Technician, MySimplePetLab; ^3^CoFounder, Chief Lab & Innovation Officer, MySimplePetLab; ^4^Head of Lab, MySimplePetLab; ^5^Head of Tech and Automation, MySimplePetLab

**Background:** Storage conditions may affect urine biochemical parameters. Urine samples in preservative‐containing sample tubes may negate the effects of storage conditions.

**Objective:** To compare biochemical results and specific gravity (SG) for urine stored over 5 days in commercially available chlorhexidine preservative tubes and preservative‐free (PF) plastic tubes.

**Methods:** For 8 pooled canine urine samples, we prepared 32 aliquots each (4 aliquots per sample set) for preservative tubes and PF tubes. Samples were analyzed immediately after adding urine to preservative and PF tubes; other aliquots were stored at room temperature and then designated aliquots were analyzed 3 and 5 days later. We used a urine strip reader and a refractometer.

**Results:** Bilirubin, blood, ketone, and leukocytes were unchanged or varied slightly over day, volume, and tube. Considering storage over time, the same 5/8 samples in PF tubes had both varying pH and 1‐2‐unit semi‐quantitative differences in total protein (TP) but less variable urine protein‐to‐creatinine (UPC) ratios. Similarly, TP varied by 2 units for 4 samples in preservative tubes. Glucose varied in 3 samples in PF tubes and in 1 sample in preservative tube. Considering 1 versus 7 mL urine volumes irrespective of day, 3‐4 samples in preservative tubes had at least 2‐unit differences for TP; UPC ratios varied slightly. Urine SGs were unaffected clinically.

**Conclusion:** Urine samples stored over 5 days at room temperature in tubes with and without chlorhexidine preservative yielded variable TP. The preservative tubes may not provide an advantage over PF tubes for urine biochemical parameters.

## ABSTRACT NU28: Feline chronic kidney disease is associated with significant caregiver burden

### Lina Lim^1^; Jessica Quimby^2^, DVM, PhD, DACVIM; Sarah Caney^3^, BVSc, PhD, DSAM (Feline), MRCVS

#### ^1^College of Veterinary Medicine, The Ohio State University, Columbia, OH, USA; ^2^Professor, Vice‐Chair, Research and Scholarship Small Animal Internal Medicine, Veterinary Clinical Sciences, College of Veterinary Medicine, The Ohio State University, Columbia, OH, USA; ^3^Vet Professionals

**Background:** Caregiver burden has been documented in management of chronic diseases in companion animals, but its impact on feline chronic kidney disease (CKD) has not been evaluated.

**Hypothesis/Objectives:** To investigate caregiver burden in feline CKD using the abbreviated Zarit Burden Interview (ZBI) adapted and validated for use in pets.

**Animals:** Survey completed by caregivers of CKD cats IRIS stages 1‐4 (n = 180).

**Methods:** Descriptive survey study. Caregivers managing a CKD cat or having previous recent experience were recruited through social media to anonymously participate in an online Qualtrics survey. The abbreviated ZBI was incorporated into the survey to validate assessment of caregiver burden. A ZBI score of 18 or higher represents significant burden.

**Results:** 46% of caregivers reported a ZBI score of 18 or higher, representing significant caregiver burden. ZBI was significantly higher in IRIS Stages 3 & 4 (median 18, range 3.00‐46.00) versus IRIS Stages 1 & 2 (median 14, range 2‐42) (*P* = .01). ZBI was significantly higher in caregivers who rarely or never shared care burden (median 19, range 3‐46) versus those who regularly shared (median 14, range 2‐38) (p = 0.01). Those who spent 30 minutes or longer (median 19, range 4‐46) had significantly higher ZBI versus those who spent less than 30 minutes (median 11.5, range 2‐42) to care for their CKD cat (*P* ≤ .0001). ZBI significantly increased when 4 or more medications were given versus 3 or fewer (*P* ≤ .0001).

**Conclusions and Clinical Importance:** Significant caregiver burden exists in feline CKD.

## ABSTRACT NU29: Evaluation of a salivary urea nitrogen test in cats with lower urinary tract outflow obstructions

### Amber Carson^1^; Deborah Yee^2^, DVM; Sydnie Stroebel^1^; Lisa Thompson^1^, DVM, ACVECC; Joseph Cyrus^1^, DACIM

#### ^1^Pulse Veterinary Specialists and Emergency, Sherwood Park, AB, Canada; ^2^Rotating Intern, Pulse Veterinary Specialists and Emergency, Sherwood Park, AB, Canada

**Background:** Urethral obstruction (UO) can be a fatal disease in male cats. Prolonged hospitalization and re‐obstruction are common. Non‐invasive, and cost‐effective method of testing the kidney function at home could help owners seek veterinary care earlier. A commercially available feline saliva urea nitrogen (SUN, Kidney‐Chek, SN Biomedical Inc., Canada) test is available.

**Objectives:** The purpose of this study is to determine if this salivary test can detect azotemia in cats with UO.

**Animals:** Adult (>1 year of age) male domestic cats with UO presenting to the emergency service at a single center were enrolled in this study. UO was defined as a firm, non‐expressible bladder on physical examination (and a bladder greater than 4 cm on ultrasound when performed). Cats that received fluid therapy or had a urinary catheter/cystocentesis in the past 72 hours were excluded from the study.

**Methods:** Cats with urethral obstructions were unblocked, and over the course of hospitalization, their blood urea nitrogen (BUN) and blood creatinine was assessed every 24 hours. A saliva urea nitrogen test was run simultaneously. The study was done in accordance with VICH guidelines.

**Results:** In cats with urinary obstructions, the SUN and BUN were positively correlated (*Ρ* = 2.5×10^−5^, *P* < .05) using Spearman's correlation. SUN and creatinine were also positively correlated (*Ρ* = 4.5×10^−6^, *P* < .05). SUN and BUN were comparable when normal, borderline, and abnormal parameters were used.

**Conclusion and Clinical Importance:** The salivary urea nitrogen test can be used to evaluate azotemia in cats with urinary outflow obstruction.

## ABSTRACT NU30: Potential for extending the chloramphenicol dosing interval for canine urinary tract infections

### Elayna Anderson^1^; Kate KuKanich^2^ ; Astrid Carcamo‐Tzic^2^; Mark Papich^3^; Butch KuKanich^2^

#### ^1^College of Veterinary Medicine, Kansas State University, Manhattan, KS, USA; ^2^Kansas State University, Manhattan, KS, USA; ^3^North Carolina State University, Raleigh, NC, USA

**Background:** The ideal frequency of oral chloramphenicol administration for canine urinary tract infections is undetermined.

**Objective:** To determine the canine urinary excretion protocol of chloramphenicol and optimize a dosing protocol.

**Animals:** Seven healthy male intact purpose‐bred Beagles and six healthy client‐owned dogs of various breeds.

**Methods:** Each dog received a single oral 50 mg/kg dose of chloramphenicol. Urine was collected at baseline, 6, 8, 12, and 24 hours after drug administration.

**Results:** At 8 hours, mean urine concentration of chloramphenicol from all dogs was 266.9 mcg/mL (90% CI 136.2‐397.7 mcg/mL) but was lower in Beagles (mean 52.8 mcg/mL, 90% CI 8.5‐97.0) than client‐owned dogs. At 12 hours, mean urine concentration from all dogs was 110.5 mcg/mL (90% CI 36.4‐184.7 mcg/mL) and again was lower in Beagles (mean 10.6 mcg/mL, 90% CI 1.4‐19.8 mcg/mL) than client‐owned dogs (mean 227.1 mcg/mL, 90% CI 101.1‐353.1 mcg/mL). The urine half‐life was similar for Beagles (1.84‐3.84 h) as for client‐owned dogs (1.78‐3.04 h).

**Conclusions and Clinical Importance:** These findings justify the current common practice of dosing chloramphenicol 50 mg/kg PO q 8 hours for canine UTI, rather than the labeled dose of q 6 hours. However, all client‐owned dogs maintained therapeutic concentrations well above 8 mcg/mL, the wild‐type cutoff target MIC for uropathogenic *E. coli*, for 12 hours, suggesting that q 12‐hour dosing might be appropriate for non‐Beagle dogs with susceptible lower urinary tract infections. A clinical trial in dogs with urinary tract infections is needed as well as further investigation into potential breed differences.

## ABSTRACT NU31: Retrospective evaluation of risk factors for kidney injury after angiotensin‐converting enzyme inhibitor treatment in dogs

### **Minju Baek**; Yelim Lee; Dongseop Lee; Jinyeong Park; Yeon Chae; Byeong‐Teck Kang; Taesik Yun; Hakhyun Kim

#### Laboratory of Veterinary Internal Medicine, College of Veterinary Medicine, Chungbuk National University, Cheongju, South Korea

**Background:** Angiotensin‐converting enzyme inhibitors (ACEi) are frequently prescribed in dogs. One of the potential adverse effects of ACEi is acute exacerbation of azotemia, which is generally well tolerated. However, evaluation of renal function is recommended 1 to 2 weeks after ACEi are added because severe worsening of renal function can occur in some dogs.

**Hypothesis/Objectives:** To identify risk factors for development of kidney injury (KI) in dogs treated with ACEi for cardiac diseases, proteinuria, and systemic hypertension.

**Animals:** A total of 156 client‐owned dogs receiving ACEi were included.

**Methods:** Serum creatinine (sCr) concentration was determined at the initial presentation and first reevaluation to detect and stage KI (increase in sCr ≥0.3 mg/dL). Demographic data, serum chemistry data such as total protein, albumin, blood urea nitrogen, creatinine, symmetric dimethylarginine, glucose, triglyceride, and total cholesterol, and serum electrolyte concentrations at first presentation were evaluated, and multivariable modeling was performed to identify risk factors for KI after treatment with ACEi.

**Results:** Kidney injury was identified in 27/156 (17%) dogs after treatment with ACEi. Kidney injury was Grade 1 in 17 dogs, Grade 2 in 2 dogs, and Grade 3 in 8 dogs. Only concurrent administration of furosemide (odds ratio, 5.05; 95% confidence interval, 2.05‐12.4; *P* = .00) and pre‐existing azotemia (odds ratio, 3.12; 95% confidence interval, 1.28‐8.03; *P* = .01) were associated with KI in dogs receiving ACEi.

**Conclusions and Clinical Importance:** Although KI is uncommon and mild, ACEi should be cautiously prescribed in dogs who are receiving furosemide or have pre‐existing azotemia.

## ABSTRACT NU32: Episioplasty reduces the incidence of urinary tract infections and perivulvar pyoderma in dogs

### **Leah Ramsaran**; JD Foster, VMD, DACVIM (SAIM)

#### Friendship Hospital for Animals

**Background:** Episioplasty is a surgical procedure that removes excess skin surrounding a recessed vulva. Episioplasty is a recommended treatment for patients with recurrent urinary tract infections (UTIs) or perivulvar pyoderma attributed to the vulvar recession. Large studies examining the efficacy of episioplasty are unavailable. The aim of this study was to determine the effect of episioplasty on the incidence of UTIs and perivulvar pyoderma.

**Hypothesis/Objectives:** We hypothesized that episioplasty would lead to a significant reduction in frequency of perivulvar pyoderma and UTIs after surgical correction.

**Animals:** 190 client‐owned female dogs with a recessed vulva who underwent episioplasty surgery.

**Methods:** Multicenter retrospective study of dogs undergoing episioplasty surgery. Frequency of UTI and presence of perivulvar pyoderma were recorded within 6 and 12 months pre‐ and post‐surgery. Wilcoxon matched‐pairs signed‐ranked test was used to compared number of UTI pre‐ and post‐surgery. Fischer's exact test was used to compare the frequency of pyoderma pre‐ and post‐surgery.

**Results:** The median number of UTIs occurring 6 months and 1 year prior to episioplasty was significantly decreased from 1.5 and 2 episodes, respectively, to 0 and 0 after episioplasty.

**Conclusions and Clinical Importance:** Episioplasty was associated with a reduction in the frequency of UTI and occurrence of perivulvar pyoderma in patients with a recessed vulva. This surgery may be an effective treatment when a recessed vulva causes these complications.

## ABSTRACT NU33: Retrospective evaluation of hypoalbuminemia in proteinuric dogs

### **Jamie S. Hart** ^1^; JD Foster^2^, VMD, MS, DACVIM (SAIM)

#### ^1^Friendship Hospital for Animals; ^2^Internist, Nephrology and Urology, Friendship Hospital for Animals

**Background:** Glomerular disease results in proteinuria, predominantly albuminuria. Hypoalbuminemic renal disease is associated with increased mortality. Correlation between the magnitude of proteinuria, assessed by urine protein: creatinine ratio (UPC), and the development of hypoalbuminemia in dogs is unknown.

**Objective:** To determine the frequency of hypoalbuminemia in dogs with renal proteinuria and to evaluate for correlation with UPC.

**Animals:** 181 dogs with suspected renal proteinuria.

**Methods:** Retrospective study of dogs that had concurrent urinalysis and serum chemistry analyzed between August 2021 to August 2023. Dogs with tubular, pre‐renal, and post‐renal proteinuria, liver disease, or non‐renal causes of hypoalbuminemia were excluded. Spearman correlation was performed between UPC and serum albumin concentration. Frequency of hypoalbuminemia (serum albumin <2.7 g/dL) was calculated. P < 0.05 was considered significant.

**Results:** 44 of 181 dogs (24.3%) were hypoalbuminemic. Correlation between UPC and serum albumin did not reach significance (*P* = .09), but was significantly correlated (*P* = .0037, *r* = −0.2519) when evaluating dogs with UPC >1. In dogs with UPC >0.5, there was a significant difference (*P* = .01) between the frequency of hypoalbuminemia among quartiles, Q1 31%, Q2 11%, Q3 17%, and Q4 38%. Dogs with proteinuria of UPC >1, there was a nonsignificant trend (*P* = .26) in the frequency of hypoalbuminemia between quartiles; Q1 12.5%, Q2 21%, Q3 24%, Q4 33%.

**Conclusions and Clinical Importance:** UPC was weakly correlated with serum albumin in dogs with UPC >1, but not when those with UPC 0.5‐1 were included. The observed trend of increased frequency of hypoalbuminemia with worsening proteinuria warrants further study including albuminuria measurement.

## ABSTRACT NU34: The effect of Porus one on amino acid concentrations in cats with chronic kidney disease

### **Rene E. Paschall** ^1^; Jessica Quimby^1^; Bianca Lourenco^2^; Stacie Summers^3^; Chad Schmiedt^2^; Jan Suchodolski^4^; Amanda Blake^4^

#### ^1^The Ohio State University, Columbus, OH, USA; ^2^University of Georgia, Athens, GA, USA; ^3^Oregon State University, Corvallis, OR, USA; ^4^Texas A&M University, College Station, TX, USA

**Background:** Porus One is an oral adsorbent that binds uremic toxin precursors in the gut. The effect of Porus One on serum amino acid concentrations (sAA) has not been described.

**Objectives/Hypothesis:** Assess the effect of Porus One on sAA concentrations in cats with CKD. We hypothesized no significant effect on sAA would be observed.

**Animals:** 14 purpose‐bred cats (13 IRIS Stage 2, 1 IRIS Stage 3).

**Methods:** Prospective cross‐sectional study utilizing cats with remnant kidney model‐induced CKD. Cats were treated with consistent standard of care and diet (Days −56 to 0) and then 500 mg Porus One PO q24h (Days 0 ‐28) in food. Twenty‐one essential and non‐essential sAA were measured on Days ‐56, ‐28, 0, and 28 using an ion exchange chromatography amino acid analyzer (Biochrom 30+). sAA were compared between all time points with a Friedman test.

**Results:** There was significant variability in baseline sAA between Days −56, −28, and day 0. Tryptophan was significantly increased at Day 28 (median 68.5 μM, range 49.9‐86.6) in comparison to Day 0 (median 60.9 μM, range 45.4‐78.1) (*P* = .003). Arginine was significantly decreased at Day 28 (median 100.0 μM, range 67.3‐132.9) in comparison to Day 0 (median 115.8 μM, range 63.4‐153.0) (*P* = .03).

**Conclusions and Clinical Importance:** Minimal changes in sAA occurred after administration of Porus One which are unlikely to be clinically meaningful. Clinicians should anticipate little effect on sAA in the short‐term with Porus one supplementation.

## ABSTRACT O01: Hypoxia‐inducible factor 1α expression in canine urothelial carcinoma

### **Nicole H. Gibbs**; Mario Sola; Deepika Dhawan; Deborah Knapp; Andrew Woolcock, DVM, DACVIM (SAIM)

#### Purdue University, Lafayette, IN, USA

**Background:** Tumor microenvironment and variable gene expression play an important role in tumor growth and can mediate chemotherapy resistance. Hypoxia‐inducible factor (HIF‐1α) is increased in urothelial carcinoma (UC) in people and is negatively correlated with survival. Urothelial carcinoma (UC) is the most common urinary bladder neoplasia in dogs, but HIF‐1α has not been investigated in this canine tumor.

**Hypothesis/Objectives:** We hypothesized that HIF‐1α will be increased in canine UC when compared to normal canine urinary bladder tissue.

**Animals:** Ten dogs with confirmed UC, and 10 dogs in the control group with normal urinary bladders.

**Methods:** Normal urinary bladder or UC was confirmed on hematoxylin‐eosin stained tissue and uroplakin‐3 IHC. Immunohistochemistry was then performed on paraffin sections though incubation with the primary antibodies against HIF‐1α. Immunolabeled slides were examined and a semiquantitative immunoreactivity score was applied in which intranuclear labeling of HIF‐1α was considered positive (0‐3+; Table 1).

**Results:** Positive intranuclear labeling for HIF‐1α was observed in 5 (50%) of the UC samples, and was scored as 1+ in these 5 with less than 10% of cells displaying positivity. All of the normal bladder tissue displayed intranuclear labeling for HIF‐1α, with 9/10 scored 1+, and the tenth being 2+ with moderate intensity.

**Conclusions and Clinical Importance:** There is variable expression of HIF‐1α in dogs with UC, with more consistent expression of HIF‐1α seen in normal canine bladder tissue. Further investigation of this transcription factor through RNA sequencing of canine UC tissue for genes encoding for HIF‐1α is ongoing.

**Table:** **Table 1.** A semiquantitative immunoreactivity score (IRS) was used to evaluate the immunohistochemical labeling of canine UC and healthy urinary bladder tissue using a primary antibody against hypoxia‐inducible factor (HIF‐1α)

Percentage of positive cells (POPC)	Intensity of labeling (IOL)	Immunoreactivity score (IRS) (IRS = POPC × IOL)
0: No labeling	0: No labeling	0: Total of score of 0
1: <10%	1: Mild labeling	1+: Total score < 3
2: <50%	2: Moderate labeling	2+: Total score < 6
3: <80%	3: Intense labeling	3+: Total score < 12
4: >80%		

Scores were assigned based on the percentage of positive cells (POPC) and intensity of labeling (IOL). The product of these two scores was used to assign an IRS of 0, 1+, 2+, or 3+. This IRS has been previously used in canine mammary tumors (Madej JA, Madej JP, Dziegiel P, et al. Expression of hypoxia‐inducible factor‐1α and vascular density in mammary adenomas and adenocarcinomas in bitches. *Acta Vet Scand*. 2013;55:73.).

## ABSTRACT O02: Transcriptional profiling of the immune tumor microenvironment in canine cutaneous mast cell tumors

### **Kathleen L. Bardales** ^1^; Jennifer Lenz^2^; Charles Antoine Assenmacher^3^; Matthew Atherton^2^

#### ^1^School of Veterinary Medicine, University of Pennsylvania, Philadelphia, PA, USA; ^2^Department of Clinical Sciences and Advanced Medicine, School of Veterinary Medicine, University of Pennsylvania, Philadelphia, PA, USA; ^3^Department of Pathobiology, School of Veterinary Medicine, University of Pennsylvania, Philadelphia, PA, USA

**Background:** Canine cutaneous mast cell tumors (MCTs) are a common, yet clinically challenging tumor type given their variable biological behavior. While patients with histologically low‐grade MCTs can often be effectively managed with surgery alone, the majority of those with high‐grade MCTs succumb to their disease despite multimodal therapy. An improved understanding of the immune tumor microenvironment (TME) may help identify novel prognostic and therapeutic targets.

**Hypothesis/Objectives:** The objective of this study was to interrogate the transcriptional profiles for differences in the immune TME between low‐ and high‐grade MCTs.

**Animals:** Twelve client‐owned dogs with MCTs (6 Kiupel low‐grade with clinically benign behavior and 6 Kiupel high‐grade with clinically aggressive behavior) that underwent curative‐intent surgery (Figure 1).

**Methods:** Archived low‐ and high‐grade cutaneous MCTs were retrospectively identified. Tumor grade was confirmed by a single veterinary pathologist. RNA was extracted from all tumors followed by immune transcriptional profiling (Nanostring Canine IO panel) and analysis (ROSALIND platform).

**Results:** Immune transcriptional profiling identified 9 differentially expressed genes (p‐adj <0.05, Figure 2). Programmed cell death protein 1 (PDCD1) and inducible T‐cell costimulator ligand (ICOSLG) gene expression were significantly higher in a subset of high‐grade MCTs.

**Conclusions and Clinical Importance:** Our data revealed significant differences in the immune transcriptome of low‐ and high‐grade MCTs and provides early, pre‐clinical rationale for targeting a subset of high‐grade MCTs with immune checkpoint blockade. Efforts to confirm these findings on a protein level are ongoing.

**Figure:**
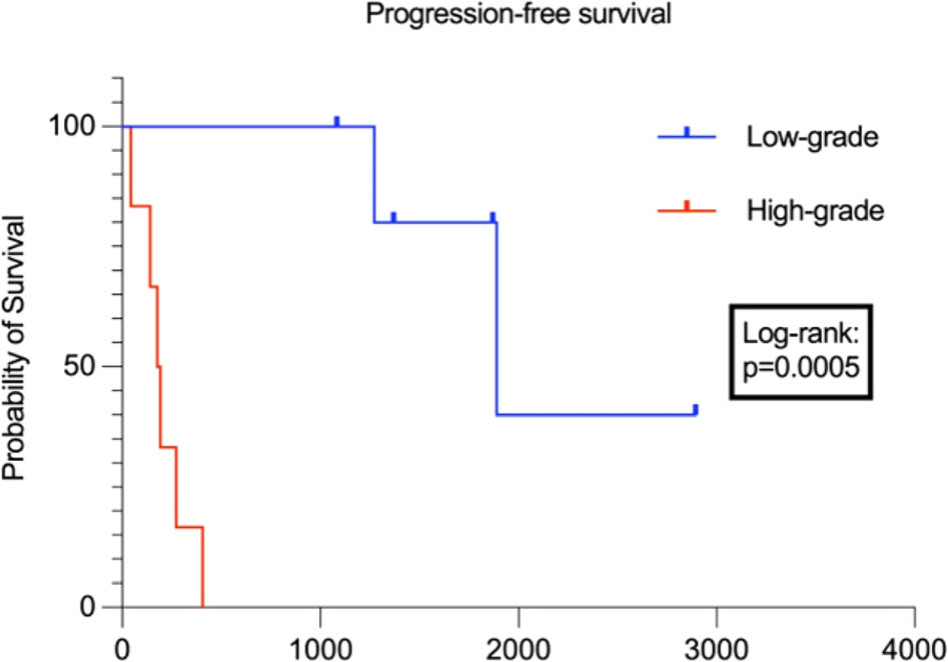
**Figure 1.** Patient progression‐free survival (PFS) times in 12 dogs with MCTs stratified by Kiupel grade (low vs. high). *P*‐value calculated using log‐rank test.

**Figure:**
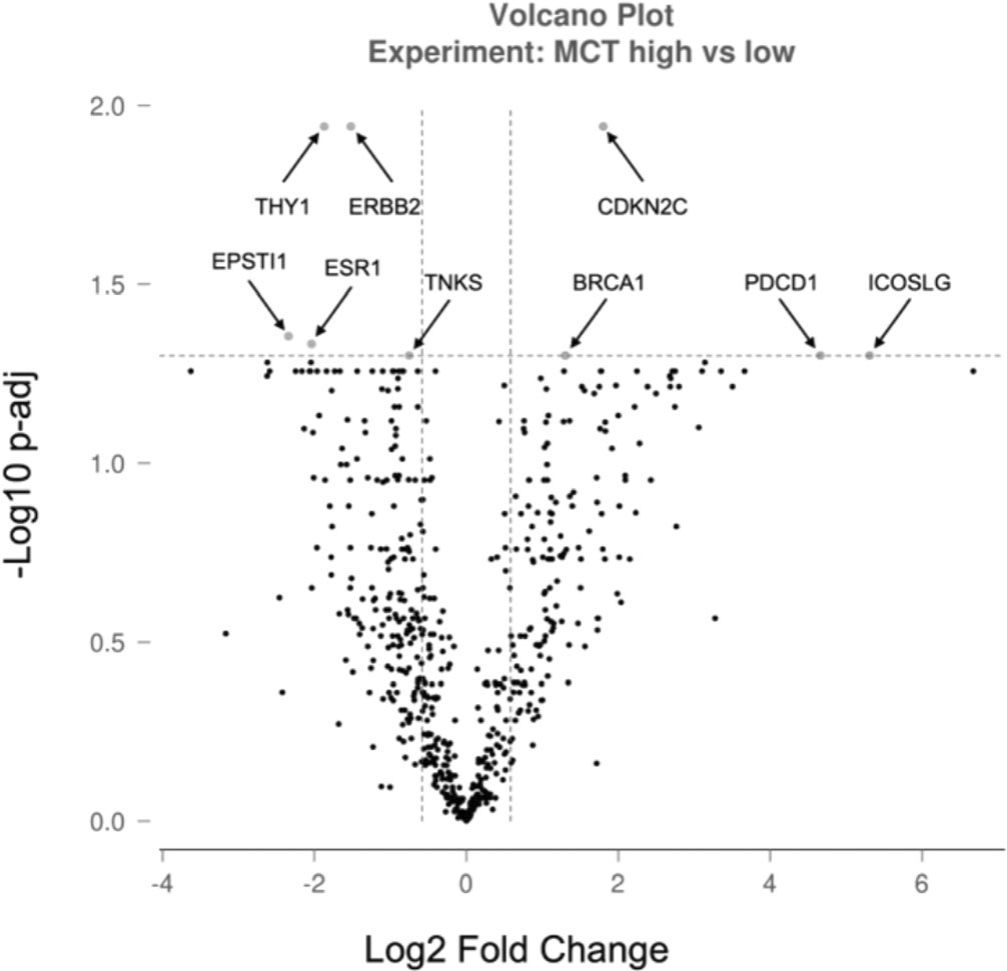
**Figure 2**. Volcano plot of differentially expressed genes between Kiupel low‐ and high‐grade MCTs. Each dot represents a single gene.

## ABSTRACT O03: Unveiling shared oncogenic mutations and signaling pathways in canine and human hepatocellular carcinoma

### **Guannan Wang** ^1^; William Hendricks^2^; Nikolaos Dervisis^3^, DVM, PhD

#### ^1^Vidium Animal Health, Phoenix, AZ, USA; ^2^Founder and Chief Scientific Officer, Vidium Animal Health, Phoenix, AZ, USA; ^3^Associate Professor, Department of Small Animal Clinical Sciences, Virginia‐Maryland College of Veterinary Medicine, Virginia Tech, Blacksburg, VA, USA

Hepatocellular carcinoma (HCC) is the most prevalent primary liver tumor in dogs, with no effective treatment for non‐resectable disease. To pave the way for the development of targeted therapeutics and to assess the translational value of the disease, it is imperative to study the genomic makeup of canine HCC. We aim to describe mutations in canine HCC, and draw comparisons with commonly affected genes and pathways in human HCC. Tumor tissues from 40 dogs diagnosed with HCC were collected via retrospective study of biobanked material, and through clinical cases submitted to Vidium. Forty HCC tumor samples were analyzed by SearchLight DNA, a 120‐gene sequencing panel validated to detect common cancer mutations. Mutations were annotated by Vidium Insight, a comprehensive canine precision oncology knowledgebase, for the prediction of their roles in HCC pathogenicity and clinical relevance for diagnosis, prognosis, and treatment. We identified 445 (155 unique) mutations in 67 genes. The mean and median mutation burden per case were 11 and 9, respectively (range: 1‐35). TP53, CDKN2B, CTNNB1, APC, and ATM were the most commonly mutated genes. Equivalent mutations in over 20 genes are known to be pathogenic in human HCC, and these genes are enriched in WNT/β‐catenin, cell cycle, and chromatin remodeling pathways. Our data revealed several molecular subtypes exhibiting unique mutation profiles in canine HCC. Prevalent mutations in the WNT/β‐catenin signaling resemble those found in human disease, highlighting the translational value of the canine disease as a model for human HCC.

## ABSTRACT O04: Comparative analysis of metabolic flexibility in canine and murine osteosarcoma cells

### **Andrew C. Poon** ^1^; Anita Luu^2^, MSc, PhD; Alicia Viloria‐Petit^3^, MSc, PhD; Anthony Mutsaers^3^, DVM, PhD, DACVIM (Oncology)

#### ^1^Ontario Veterinary College, University of Guelph, Markham, ON, Canada; ^2^Biomedical Sciences, Ontario Veterinary College, University of Guelph, Guelph, ON, Canada; ^3^Associate Professor, Biomedical Sciences, Ontario Veterinary College, University of Guelph, Guelph, ON, Canada

**Background:** Metabolic adaptations fuel the outgrowth of tumor cells during cancer initiation and progression. Appendicular osteosarcoma (OS) is an aggressive bone tumor of mesenchymal origin in domestic dogs, presenting a naturally occurring model for translational studies.

**Hypothesis/Objectives:** Using a cross‐species approach, central carbon metabolism was assessed in 10 primary and metastatic canine and murine OS cell lines in comparison with mesenchymal stromal cells.

**Methods:** In vitro study using patient‐derived and transgenic cell lines, grown under variable oxygen tension and biomimetic hydrogel environments. Metabolism measured using Seahorse respirometry, cell viability assays, and mass spectrometry of glycolysis, citric acid cycle, and pentose phosphate pathway metabolites.

**Results:** Metastatic canine and murine OS cells displayed a high degree of metabolic flexibility in the energetic state compared to primary cells. Hypoxia increased relative cell viability of canine and murine OS under selective availability of glucose, glutamine, or pyruvate. Adjusting hydrogel matrix stiffness alone was sufficient to reprogram the metabolic signature of primary and metastatic OS cells, with evidence of differentially upregulated metabolites in nine cell lines under soft, lung‐like hydrogel environments. L‐lactate consumption directly contributed to mitochondrial oxidation in real‐time, and inhibition of the malate‐aspartate shuttle impaired lactate metabolism. Inhibition of lactate shuttling by monocarboxylate transporter (MCT)1/4 dual inhibition was superior to MCT1 inhibition alone in combination with carboplatin chemotherapy for reduction of OS cell viability.

**Conclusions and Clinical Importance:** These findings represent an investigation into the metabolic flexibility of OS and may hold future clinical implications in precision oncology.

## ABSTRACT O05: Alkaline phosphatase and mast cell tryptase labeling in canine skin‐associated mast cell tumors

### **Leah Dunston** ^1^; Russell Moore^2^, DVM, MS, DACVP; Lisa Schlein^2^, DVM, PhD, DACVP

#### ^1^VCA Northwest Veterinary Specialists, Saint Paul, MN, USA; ^2^Department of Microbiology, Immunology, and Pathobiology, Colorado State University, Fort Collins, CO, USA

**Background:** A Colorado State University (CSU) dog was diagnosed with a poorly differentiated alkaline phosphatase (ALP) positive tumor that was most consistent with a mast cell tumor (MCT) with hematogenous involvement. Immature hematopoietic cells are ALP+ but reactivity in MCT is unknown. Tryptase immunohistochemistry labels MCT granules with limited data on immunocytochemical (ICC) labeling or its relation to grade.

**Objective:** Evaluate the association of ALP reactivity and tryptase ICC labeling with cytologic grade of canine MCT.

**Animals:** 38 dogs diagnosed with skin‐associated MCT at CSU between December 2021 and November 2023.

**Methods:** The CSU Clinical Pathology archives were searched for canine skin‐associated MCT cytologic specimens with sufficient archived Wright‐Giemsa‐stained slides. Staining for ALP reactivity was performed on destained slides, as previously published. Tryptase ICC was performed using the Leica Bond‐III system. Grades were assigned by two ACVP‐boarded pathologists separately using a published two‐tier grading system and then discordant cases were resolved. Cohen's κ and Fisher's exact statistical tests were used.

**Results:** Independent cytologic grades agreed for 36/38 cases, Cohen's κ (0.86, 0.68‐1.00) was excellent, but 9/28 patients with cytologic low grade MCTs had nodal metastasis. ALP reactivity was not different between grades (*P* = 1.00); 2/28 low grade and 0/10 high grade MCTs were ALP+. Tryptase labeling was not associated with grade (*P* = .29); 20/20 low grade and 7/8 high grade MCTs were tryptase+. The ALP+ and tryptase− cases had nodal metastasis.

**Conclusions and Clinical Importance:** Canine MCT is most often ALP−/Tryptase+. An association with cytologic grade was not found.

acvim24_596.

## ABSTRACT O06: Retrospective analysis of canine testicular tumors: 101 cases

### **Yiyu Li** ^1^; Gebin Li^2^; Yang Liu^1^; Yujia Peng^1^

#### ^1^Veterinary Teaching Hospital, China Agricultural University, Beijing, Beijing, China (People's Republic); ^2^China Agricultural University, Beijing, Beijing, China (People's Republic)

**Background:** Testicular tumors are the most common tumors of the canine male genitalia. However, studies in China with a large sample size were rare.

**Hypothesis/Objectives:** The aim of this study was to investigate the incidence and outcome of testicular tumors in dogs, and to explore the influence of tumor itself and bone marrow suppression on the prognosis.

**Animals:** 101 eligible canine testicular tumor cases were collected from the Veterinary Teaching Hospital of China Agricultural University from May 2020 to July 2022.

**Methods:** The clinical records were retrospectively reviewed. Dogs with non‐neoplastic testicular disease were excluded. Signalment, clinical presentation, clinicopathological abnormalities, treatment, and histopathology results were recorded.

**Results:** Leydig cell tumor (51.7%), seminomas (27.3%), and Sertoli cell tumor (17.5%) were the most common types of testicular tumors. Sertoli cell tumor (76.9%) was the most common tumor type of abdominal cryptorchid tumor. Poodles (22.8%), golden retrievers (19.8%), and mixed breed dogs (8.9%) were the most common breeds. The mean age at diagnosis was 10.9 years. There was no significant difference in the age at diagnosis between different types of tumors (*P* > .05). 4 (3.9%) cases had pancytopenia and all were diagnosed with abdominal cryptorchid Sertoli cell tumor, accounting for 17.4% (4/23) of all Sertoli cell tumor cases and 44.4% (4/9) of the cases that were diagnosed with abdominal cryptorchid Sertoli cell tumor. 4.2% (4/95) had lymph node involvement and a single patient had lung metastasis. The prognosis of dogs with pancytopenia was poor, and the survival period was within 30 days. The overall half‐year, one‐year, and two‐year survival rates of affected dogs were 88.4%, 73.8%, and 44.4%, respectively. The cause of most of the fatal cases (61.5%) was not related to testicular tumors.

**Conclusions and Clinical Importance:** The overall prognosis of dogs with testicular tumors was excellent, but for cases with pancytopenia or metastasis, the prognosis was grim.

## ABSTRACT O07: Unraveling correlations between exercise and neoplastic disease in golden retrievers using big data analysis

### **Dennis Ronzani** ^1^; Sarah Hooper^2^, DVM, MS, PhD

#### ^1^Veterinary Medicine, Ross University, Vernon, BC, Canada; ^2^Director Research Centre for Veterinary Education, Diversity, and Data Analytics, Biomedical Sciences, School of Veterinary Medicine, Ross University, Basseterre, Saint Kitts

**Background:** Neoplasia has been linked to exercise levels in humans for some time. Due to the similarities in the behavior of canine and human tumors this has often been extrapolated to dogs as well. The purpose of this study is to explore how exercise levels in canines correlate with the development of neoplasia.

**Hypothesis/Objectives:** We hypothesize machine learning models will be able to correctly classify if a golden retriever develops cancer, and important predictors will be related to the level of aerobic exercise over the patients' lifetime.

**Animals:** The first 7 years of available medical records and owner surveys from 3044 golden retrievers enrolled in the Morris Animal Foundation (MAF) Golden Retriever Lifetime Study (GRLS) were included.

**Methods:** All golden retriever records related to neoplastic diagnosis, activity, and exercise engagement underwent data pre‐processing and feature selection prior to the development of binary mixed model (BiMM) forest prediction models.

**Results:** Preliminary BiMM models incorporating 14 variables performed well when classifying if a golden retriever would develop cancer (average out‐of‐bag error of 4.77%). The top 5 most important predictors were the pace; average yearly activity level, the total time engaged in activity, the grade of the surface, and frequency of exercise.

**Conclusions and Clinical Importance:** Our results suggest that overall amount of time exercising and the pace of the activity are more influential than type of exercise. These results can help clinicians provide better recommendations related to exercise when discussing with owners how to reduce the risk of cancer.

## ABSTRACT O08: A preliminary prospective study: Sorafenib in aggressive canine carcinomas—Tolerability and clinical efficacy

### **Doyun Kim** ^1,2^; Mihyun Choi^3^, DVM, PhD; Yongsun Kim^3^, DVM, PhD; Jaechun Cho^1^, DVM, MS; Woo‐jin Song^4^, DVM,DKCVIM, PhD

#### ^1^Bon Referral Animal Medical Center, Seoul, Seoul‐t'ukpyolsi, Republic of Korea; ^2^Jeju National University, Jeju City, Republic of Korea; ^3^President, Bon Referral Animal Medical Center, Seoul, Seoul‐t'ukpyolsi, Republic of Korea; ^4^Professor, Veterinary Internal Medicine, Jeju National University, Jeju City, Republic of Korea

**Background:** Sorafenib, a multi‐kinase inhibitor targeting kinases such as RAF, VEGFR‐2, and PDGFR, has demonstrated efficacy in various canine carcinomas, including hepatocellular carcinoma (HCC) and transitional cell carcinoma (TCC).

**Objective:** Evaluate sorafenib's tolerability and efficacy in the treatment of various advanced canine carcinomas.

**Animals:** Study included 9 client‐owned dogs diagnosed with various unresectable or metastatic carcinomas: HCC (n = 4), cholangiocarcinoma (n = 1), TCC (n = 1), nasal carcinoma (n = 1), exocrine pancreatic carcinoma (n = 1), and undefined hepatic carcinoma (n = 1).

**Methods:** A prospective clinical trial was performed with 9 dogs. Clinical benefit (CB) was defined as the percentage of dogs achieving complete response, partial response (PR), and stable disease (SD). Progression‐free interval (PFI) was defined as the interval between the first day of sorafenib and the day of disease progression. Toxicity was graded according to VCOG‐CTCAE.

**Results:** Sorafenib median dosage: 6 mg/kg PO, once daily (range, 5‐7.1). Median treatment duration: 88 days (range, 14‐210). To date, two dogs achieved PR, 4 were stable, and 3 progressed, resulting in an overall CB of 75%. Overall PFI was 86 days (range, 0‐210). Seven dogs, not previously reported, exhibited improved appetite and increased body weight, indicative of clinical benefit. One unresectable cholangiocarcinoma case with hepatic failure improved by day 16, and one recurrent hepatocellular carcinoma decreased in volume over 70 days. No significant adverse effects were observed.

**Conclusions and Clinical Importance:** The results of this study suggest that sorafenib offers favorable clinical benefit with observed improvements and a positive safety profile.

## ABSTRACT O09: Assessment of the impact of TSG6 on chemoresistance using canine mammary gland tumor spheroids

### **Ga‐Hyun Lim** ^1,2^; Ju‐Hyun An^3^, DVM, PhD, Prof; Jeong‐Hwa Lee^4^, DVM, PhD; Kyoung‐Won Seo^4^, DVM, PhD, Prof; Hwa‐Young Youn^4^, DVM, PhD, Prof

#### ^1^Seoul National University, Seoul, Republic of Korea; ^2^VIP Animal Medical Center; ^3^Department of Veterinary Emergency and Critical Care Medicine and Institute of Veterinary Science, College of Veterinary Medicine, Kangwon National University, Chuncheon, South Korea; ^4^Laboratory of Veterinary Internal Medicine, Department of Veterinary Clinical Science, College of Veterinary Medicine, Seoul National University, Seoul, Republic of Korea

**Background:** Hypoxia affects tumor growth, angiogenesis, and anti‐cancer drug resistance in the tumor microenvironment, and TSG6 expression is known to influence HIF‐1α expression in tumor tissues. Objectives: In this study, we created TSG6 knockdown spheroids of canine mammary gland tumor (MGT) cells (CIPp and CIPm) to investigate the effects of TSG6 on angiogenesis and drug resistance in the tumor microenvironment.

**Methods:** Using a siRNA transfection system, we induced TSG6 knockdown in canine MGT cells and formed TSG6 knockdown spheroids using an ultra‐low adhesion plate. Cell viability and apoptosis were assessed by treating TSG6 knockdown spheroids with doxorubicin. Angiogenesis was evaluated by forming vascularized spheroids with canine MGT and endothelial cells (ECs).

**Results:** TSG6 knockdown led to reduced expression of tumor growth factors and multidrug resistance genes in canine MGT cells, as well as a significant reduction in hypoxic conditions within the spheroids. When treated with doxorubicin, TSG6 knockdown spheroids exhibited decreased viability and increased apoptosis. In a vascularized TSG6 knockdown spheroid model, TSG6 knockdown significantly reduced the expression of CD31 and tube formation in canine ECs.

**Conclusions and Clinical Importance:** In conclusion, we created a TSG6 knockdown spheroid model to investigate the role of TSG6 in the tumor microenvironment. TSG6 knockdown significantly reduced anti‐cancer drug resistance and angiogenesis in canine MGT cells. Therefore, TSG6 could be considered a potential therapeutic target for canine MGT.

## ABSTRACT O10: Evaluating the effectiveness of electrochemotherapy as an adjuvant therapy for oral malignant tumors following surgery

### **Gukil Joung**; Jeong‐Yeol Bae; Bo‐Kyung Kim; Joong‐Hyun Song

#### Veterinary Medical Teaching Hospital/Internal Medicine, Chungnam National University, Daejeon, South Korea

**Background:** Oral malignant tumors are locally aggressive and invasive, making it challenging to completely remove with surgery alone.

**Hypothesis/Objectives:** We hypothesize that the combination of cytoreductive surgery and electrochemotherapy (ECT) will result in better treatment outcomes for oral malignant tumors compared to surgery alone.

**Animals:** Three client‐owned dogs with malignant oral masses, diagnosed with squamous cell carcinoma (n = 2) and malignant melanoma (n = 1) were included, with no evidence of metastasis.

**Methods:** Tumoral volume was measured through visual assessment and computed tomography (CT) evaluation before and after surgery, and 4 months after ECT. Cytoreductive surgery was performed to the maximum extent of resection. Following the surgery, ECT was performed on the tumoral bed of the resection with intravenous bleomycin administration.

**Results:** The pre‐operative tumoral volumes were 4.8, 12.6, and 4.7 cm^3^, respectively. The postoperative residual tumor volumes were 1.0, 0.8, and 1.5 cm^3^. Four months after ECT, all three patients showed no evidence of tumors on visual assessment and CT evaluation. The overall response rate for ECT as an adjuvant therapy after surgery was 100% (3/3; 3 complete remission).

**Conclusions:** Adjuvant therapy with ECT is highly recommended when residual tumor remains after surgery for oral malignant tumors.

## ABSTRACT O11: Holmium laser‐assisted biopsy and palliative therapy for bladder masses in dogs and a cat

### **Hanah Go** ^1^; Jihyun Kim^1^, DVM, PhD; Seoungyob Ahn^1^, DVM, PhD; Seonyeong Jeong^1^, DVM; Yein Oh^2^, DVM, PhD, DAICVIM

#### ^1^VIP Animal Medical Center, Seoul, South Korea; ^2^Assistant Professor, College of Veterinary Medicine, Kyungpook National University, Daegu, South Korea

**Background:** Surgical options for bladder masses are limited due to their anatomic location. Additionally, frequent occurrences of urinary tract obstruction contribute to a diminished quality of life.

**Hypothesis/Objectives:** We conducted a retrospective study on the histological findings and treatment outcomes of benign/malignant bladder tumors using the holmium laser‐assisted method.

**Animals:** A total of 31 dogs and 1 cat with bladder masses were analyzed at the VIP Animal Medical Center between July 2020 and November 2023.

**Methods:** Transurethral bladder cystoscopy was performed, and the masses were removed using a holmium laser.

**Results:** Sixteen cases were diagnosed as benign and 16 as malignant. The most common histopathological finding in benign masses is polypoid cystitis (56.25%, n = 9). Fibrinous cystitis (12.5%, n = 2), and chronic cystitis with urothelial cell hyperplasia (12.5%, n = 2). All 16 malignant cases were diagnosed as urothelial carcinoma. Among these, 5 received concurrent NSAID treatment (Mean Survival Time [MST] 2.25 months), 7 received concurrent chemotherapy (MST 12.42 months), and 4 received laser treatment only (MST 12.37 months). In both benign and malignant cases, immediate and complete resolution of dysuria symptoms occurred in 12 cases.

**Conclusion and Clinical Importance:** Holmium laser‐assisted partial ablasion improves urinary obstruction, providing immediate symptom relief, and allows for concurrent histopathological examination. Additionally, significant side effects have not been observed. From this perspective, it appears to be effective alternative for the palliative treatment of bladder masses.

## ABSTRACT O12: Identifying functional roles and pathways of shared mutations in canine solid tumors

### **Hyeona Bae**; Yeseul Jeon; DoHyeon Yu

#### College of Veterinary Medicine, Gyeongsang National University, Jinju, South Korea

**ABSTRACT**

Genetic mutations contribute to solid tumors by altering the biological pathways related to tumor formation and development. Identifying these mutations is essential for the development of targeted therapies. This study aimed to identify mutated genes and altered pathways in canine solid tumors to provide a foundation for targeted cancer therapies. Four dogs with different types of neoplasias (urothelial carcinoma, adenocarcinoma, rhabdomyosarcoma, and chondrosarcoma) were randomly selected and classified into carcinoma and sarcoma groups based on histopathology. Tumor tissues were analyzed using whole‐genome sequencing, and significant variants shared within each tumor group were identified. Gene Ontology and Kyoto Encyclopedia of Genes and Genomes pathway enrichment analyses were conducted to compare the biological and functional pathways altered by the mutations in each group. Forty‐three and fifty‐eight genes with moderate‐to‐high‐impact variants were identified in the carcinoma and sarcoma groups, respectively. These genes are associated with altered pathways related to the immune system and tumor metastasis. Although mutations related to immune function were similarly observed in both groups, distinctions between the two groups were related to mutations of tumor metastasis. Mutations were identified in genes encoding cell adhesion molecules in the carcinoma group, whereas significant variations in extracellular matrix‐related molecules were evident in the sarcoma group. This study revealed mutations and modified pathways associated with immune and tumor metastatic functions in canine carcinoma and sarcoma, indicating their relevance to the development and progression of each tumor group. The distinctions indicated that different therapeutic approaches were required for each tumor group.

## ABSTRACT O13: Assessing prognostic significance of neutrophil to lymphocyte ratios in canine multicentric lymphoma in small‐breed dogs

### **SoYoung Park** ^1^; SeHoon Kim^1^; Chae‐Yoon Im^2^; Yeon‐Jung Hong^3^; Jung‐Hoon Park^4^; Mangil Han^5^; Younghee Lee^6^; Min‐Ok Ryu^7^; Hwa‐Young Youn^7^; KyoungWon Seo^7^

#### ^1^Laboratory of Veterinary Internal Medicine, College of Veterinary Medicine, Seoul National University, Seoul, South Korea; ^2^Seoul National University, Seoul, South Korea; ^3^Department of Veterinary Surgery, Western Animal Medical Center; ^4^Department of Veterinary Internal Medicine, Western Animal Medical Center; ^5^Royal Animal Medical Center, Seoul, Republic of Korea; ^6^College of Veterinary Medicine, Seoul National University, Seoul, South Korea; ^7^Laboratory of Veterinary Internal Medicine, College of Veterinary Medicine, Seoul National University, Seoul, South Korea

**Background:** Canine lymphoma, the most prevalent hematopoietic tumor in dogs, poses significant challenges in veterinary oncology. The neutrophil‐to‐lymphocyte ratio (NLR) has emerged as a prognostic indicator in various cancers, including its relevance in both human and veterinary oncology.

**Hypothesis/Objectives:** This study aims to investigate the prognostic significance of NLR in small‐ sized dogs (≤10 kg) diagnosed with multicentric lymphoma. The objective is to establish correlation between NLR and time‐to‐progression (TTP) and lymphoma‐specific survival (LSS) in these dogs.

**Animals:** A retrospective analysis involved 35 small‐sized diagnosed with multicentric lymphoma and weighing 10 kg or less.

**Methods:** Medical records and hematological data were analyzed to evaluate the correlation between NLR and clinical outcomes, specifically TTP and LSS. Prognostic significance was assessed using Kaplan‐Meier curves and Cox regression.

**Results:** Elevated NLR was correlated with a poorer prognosis concerning both TTP and LSS. Higher NLR values were associated with increased hazard ratios at various time points. Substage b dogs also exhibited earlier progression.

**Conclusions and Clinical Importance:** The study validates NLR as a valuable prognostic marker in small‐sized dogs with multicentric lymphoma, suggesting its potential utility in predicting disease outcomes in clinical settings. These findings emphasize the relevance of NLR in the clinical evaluation and management of canine multicentric lymphoma, particularly in smaller breeds.

## ABSTRACT O14: Increased disease‐free survival in dogs with oral malignant melanoma receiving CEA‐CAR‐NK‐92 MI cell therapies

### **Chiao‐Hsu Ke** ^1^; Chen‐Si Lin^1^; Ka‐Mei Sio^1^; Chin‐Hao Hu^1^; Chih‐Hung Huang^2^; Yu‐Shan Wang^1^

#### ^1^National Taiwan University, Taipei, Taiwan; ^2^National Taipei University of Technology, Taipei, Taiwan

**ABSTRACT**

Canine oral malignant melanoma (OMM) is an aggressive tumor with a high metastatic rate. Immunotherapy that potentiates dogs' immune responses is regarded as a prospectively anti‐tumor strategy. In human medicine, chimeric antigen receptor‐natural killer (CAR‐NK) cell therapy is a promising strategy for treating malignant tumors. Our previous study had established CAR‐NK‐92 MI cells targeting carcinoembryonic antigen‐expressing (CEA) tumors. Therefore, this study recruited six dogs with OMM, including two cases with grades II and four with grades III or IV (protocol code: IACUC No. NTU‐110‐EL‐00092). CEA‐CAR‐NK‐92 MI cells were intravenously administrated once a week for four or eight vaccinations. Physical examination and blood tests were regularly performed before every treatment. Side effects were assessed based on VCOG criteria. With CEA‐CAR‐NK‐92 MI cell therapies, digestive signs, especially diarrhea, were the most common side effects, but all toxicities were manageable. Notably, two dogs with local or distant metastasis had responses to treatment, which survived over 153 days. The median disease‐free interval (DFI) for the remaining four dogs was 179 days. Our data provide evidence that CEA‐CAR‐NK‐92 MI cell therapy increased the DFI in dogs with OMM with manageable side effects. However, additional clinical studies with more significant case numbers are warranted to verify our findings.

## ABSTRACT OH09: Investigating the pharmacokinetics and pharmacodynamics of glucoraphanin: A crossover study in healthy cats

### **David Griffin** ^1^; Rebekah Strunk^2^, MS; Grace Cornblatt^3^, PhD; Carolyn Warner^4^, RVT, LVT; Nicolette Wiezbiski^5^, DVM; Denise Passmore^6^, PhD

#### ^1^Nutramax Laboratories, Lancaster, SC, USA; ^2^Business & Scientific Assessment Manager, Innovation, Nutramax Laboratories Veterinary Sciences, Inc., Lancaster, SC, USA; ^3^Innovation, Nutramax Laboratories Veterinary Sciences, Inc., Lancaster, SC, USA; ^4^Clinical Research Manager, Innovation, Nutramax Laboratories Veterinary Sciences, Inc., Lancaster, SC, USA; ^5^Professional Services Veterinarian Manager, Innovation, Nutramax Laboratories Veterinary Sciences, Inc., Lancaster, SC, USA; ^6^Scientific Technical Writer, Innovation, Nutramax Laboratories Veterinary Sciences, Inc., Lancaster, SC, USA

**Background:** Sulforaphane (SFN), derived from cruciferous vegetables, shows health benefits in multiple pathways, such as Nrf2‐ARE transcription pathway, inducing phase 2 detoxification and antioxidative enzymes, and mediating the NF‐κB pathway, a pro‐inflammatory pathway. SFN bioavailability has been demonstrated in dogs.

**Objective:** This study examined oral administration regimens on the pharmacokinetic/pharmacodynamic profiles in cats for a proprietary blend of glucoraphanin (SFN precursor) and myrosinase enzyme (ERS92), and beta‐glucan blend (maitake mushroom and yeast extract).

**Animals:** Six healthy, neutered, male cats selected from a research facility.

**Methods:** In this crossover study, with a 14‐day washout between phases, 3 cats were administered a low‐dose, and 3 were administered a high‐dose in phase 1; doses were reversed for each group in phase 2. Cats were fasted 2 hours post‐administration.

**Results:** Plasma analysis revealed significant differences between doses for Cmax (*P* = .004) and AUC (*P* = 0002), with no significant differences in Tmax and T1/2. Urine analysis detected SFN metabolites in all cats. At 8 hours, cats receiving the low‐dose had an average 1.85‐fold increased NQO1 gene expression, HO‐1 gene expression was induced by 1.99‐fold ensuring the NRf2 pathway was impacted. Gene expression returned to baseline at 24 hours. Cats receiving the high‐dose had a 1.47‐fold increase in NQO1 gene expression at 8 hours and 3.22‐fold at 24 hours. HO‐1 was induced 1.92‐fold at 8 hours, and 2.65‐fold at 24 hours.

**Conclusion:** SFN has shown health benefits in humans and dogs. This is the first study on cats and SFN. Additional research is necessary to identify clinical applications.

## ABSTRACT ON01: MicroRNA differential expression analysis for identification of diagnostic biomarkers for canine visceral hemangiosarcoma

### **Laura Machado Ribas** ^1^; Kerstin Muner^2^, DVM, MSc; Nelly Elshafie^2^, DVM, PhD; Andrea Pires dos Santos^2^; Luis dos Santos^3^, DVM, MSc, PhD, DACVIM (Cardiology)

#### ^1^Purdue University, West Lafayette, IN, USA; ^2^Comparative Pathobiology, Purdue University, West Lafayette, IN, USA; ^3^Veterinary Clinical Sciences, Purdue University, West Lafayette, IN, USA

**Background:** Canine visceral hemangiosarcoma (HSA) has a poor prognosis, is often diagnosed at an advanced stage, and lacks non‐invasive definitive diagnostic methods. MicroRNAs (miRNAs) play a critical role in regulating gene expression and are known to be aberrantly expressed in several cancers; they are highly stable and conserved across species, making them appealing for biomarker studies. Our group previously identified 67 differentially expressed (DE) miRNAs in splenic HSA (≥2‐fold‐change; FDR <0.01).

**Hypothesis/Objectives:** We propose that DE miRNAs in HSA are organ‐specific. To address this hypothesis, we aim to evaluate the DE miRNAs in cardiac HSA and compare them to those previously described in splenic HSA.

**Animals:** The study comprised archived tissue samples from six dogs with cardiac HSA, six control cardiac samples, 18 splenic HSA, and six control spleens.

**Methods:** From formalin‐fixed, paraffin‐embedded tissues, total RNA was extracted, measured by fluorometry, and sequenced through small RNA sequencing (sRNA‐Seq).

**Results:** sRNA‐Seq revealed 71 miRNAs DE in cardiac tissue from HSA patients with ≥2‐fold‐change and FDR <0.01; 53 of these miRNAs were exclusively DE in the heart, supporting the claim that miRNA expression is organ‐specific in dogs with HSA.

**Conclusions and Clinical Importance:** These distinctive miRNA expression patterns may be useful as biomarkers for diagnosing and determining the site of visceral HSA in dogs. Validation in larger cohorts and blood and effusion samples will contribute to a more comprehensive understanding of the potential of miRNAs as a non‐invasive diagnostic tool for visceral HSA.

## ABSTRACT OT01: Hypoalbuminemia associated with decreased survival in cats presenting to a tertiary hospital: Retrospective case‐control study

### **Conner Hayes** ^1^; Ziaojuan Zhu^2^, PhD; Sarah Schmid^3^, DVM, DACVIM

#### ^1^University of Tennessee, Knoxville, TN, USA; ^2^Office of Innovative Technologies, University of Tennessee, Knoxville, TN, USA; ^3^Assistant Professor, Small Animal Clinical Sciences, University of Tennessee, Knoxville, TN, USA

**Background:** Hypoalbuminemia (HA) has been associated with morbidity and mortality in people. Little is known about the occurrence of hypoalbuminemia in cats and its impact on survival.

**Hypothesis/Objectives:** To describe the characteristics of cats presenting with moderate (albumin 2‐2.5 g/dL) or severe (albumin <2 g/dL) HA and compare their survival rates to cats without HA (albumin >2.9 g/dL).

**Animals:** 291 cats with HA and 291 age‐ and breed‐matched control cats.

**Methods:** Retrospective case‐control study (2018‐2023). Clinical data and outcome were reviewed. Duration of hospitalization was compared using a Mann‐Whitney *U* test. Short‐ and long‐term survival rates were analyzed with Kaplan‐Meier survival analysis accompanied by the log‐rank test and the restricted survival mean time (RMST).

**Results:** Of the 2927 cats that had a plasma albumin measured, 200 (6.8%) had moderate HA and 91 (3.1%) had severe HA. The most common disease processes associated with HA were infectious disease (34% HA, 14% controls) and neoplasia (22% HA, 10% controls). Duration of hospitalization was significantly longer in cats with HA compared to controls (median 2 [Interquartile range, IQR: 1‐4] days, median 2 [IQR: 1‐3] days, respectively; *P* < .0001). Cats with HA had lower short‐term and long‐term survival rates compared to controls (*P* < .0001 for both, Figures 1 and 2). The RMST for cats with severe HA (418 days, *P* = .0003) and moderate HA (638 days; *P* < .0001) were significantly less than that of controls (881 days).

**Conclusions and Clinical Importance:** Hypoalbuminemia is a clinically relevant negative prognostic indicator in cats.

**Figure:**
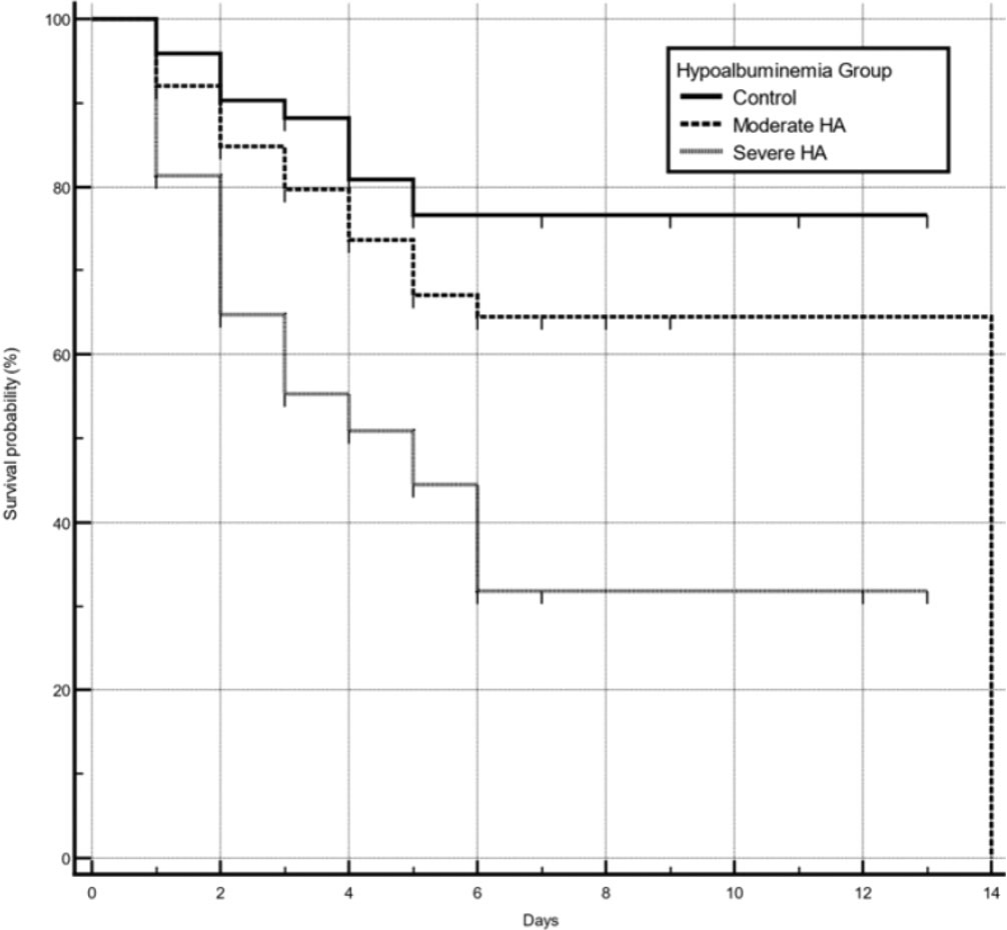
**Figure 1**. Short‐term survival of cats with moderate or severe hypoalbuminemia. Kaplan‐Meier survival curve for short‐term survival of cats presenting with moderate (albumin 2.0‐2.5 g/dL, n = 200) or severe (albumin <2.0 g/dL, n = 91) hypoalbuminemia (HA) compared to age‐ and breed‐matched control cats with normal albumin and presenting to the same service during the same time period (n = 291).

**Figure:**
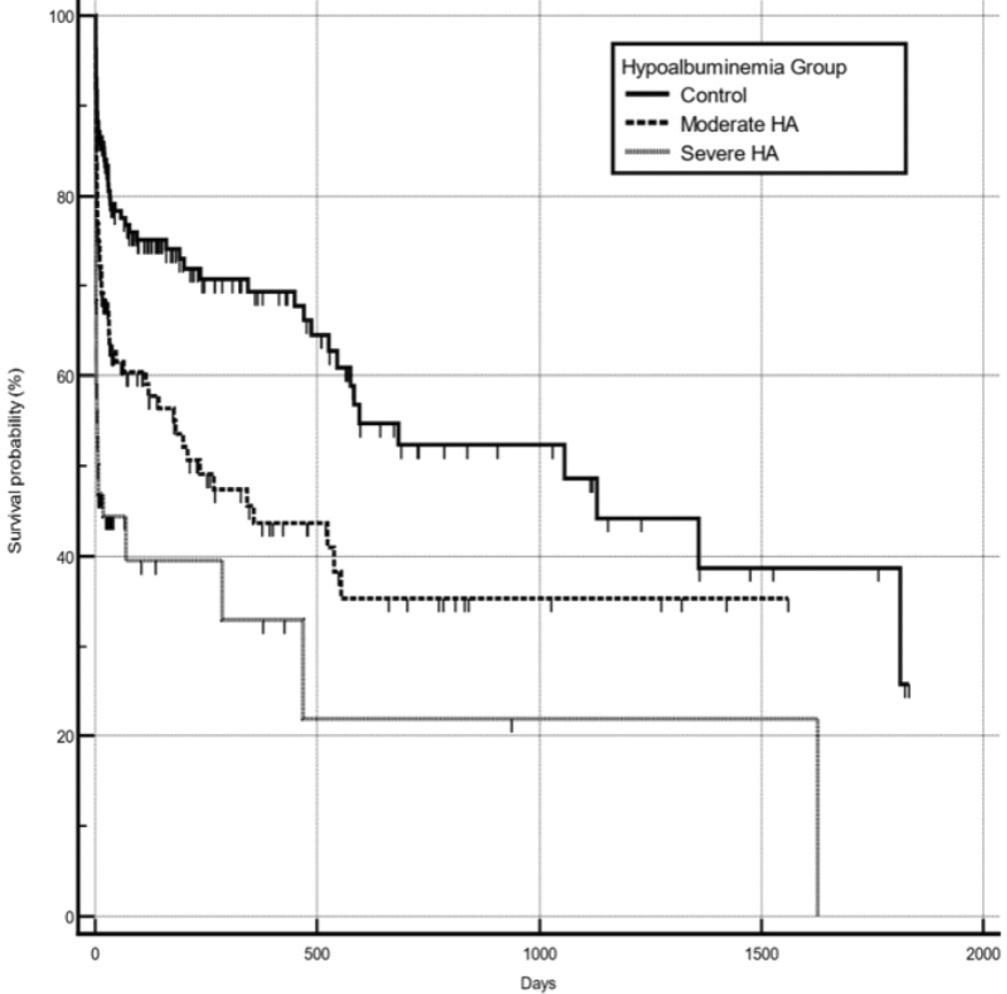
**Figure 2**. Long‐term survival of cats with moderate or severe hypoalbuminemia. Kaplan‐Meier survival curve for long‐term survival of cats presenting with moderate (albumin 2.0‐2.5 g/dL, n = 200) or severe (albumin <2.0 g/dL, n = 91) hypoalbuminemia (HA) compared to age‐ and breed‐matched control cats with normal albumin and presenting to the same service during the same time period (n = 291).

## ABSTRACT OT02: Oxidative stress induces phosphatidylserine externalization in canine erythrocytes in vitro

### **Eva Kao** ^1^; Andrea Santos^2^, DVM, MSc, PhD, DACVP (Clinical); Priscila Serpa^3^, DVM, MSc, DSc, DACVP; George Moore^4^, DVM, PhD; Andrew Woolcock^5^, DVM, DACVIM‐SAIM

#### ^1^College of Veterinary Medicine, Purdue University, West Lafayette, IN, USA; ^2^Associate Professor, Veterinary Clinical Pathology, Department of Comparative Pathobiology, College of Veterinary Medicine, Purdue University, West Lafayette, IN, USA; ^3^Research Assistant Professor, Clinical Pathology, Department of Biomedical Sciences and Pathobiology, Virginia‐Maryland College of Veterinary Medicine, Virginia Tech, Blacksburg, VA, USA; ^4^Professor of Epidemiology, Department of Veterinary Administration, College of Veterinary Medicine, Purdue University, West Lafayette, IN, USA; ^5^Associate Professor, Small Animal Internal Medicine, Department of Veterinary Clinical Sciences, College of Veterinary Medicine, Purdue University, West Lafayette, IN, USA

**Background:** Oxidative stress is well‐documented in people with hemolytic diseases and is reported in canine immune‐mediated hemolytic anemia (IMHA). Oxidative stress induces erythrocyte membrane phosphatidylserine externalization in people, causing eryptosis and thrombus formation. This has not been evaluated in dogs. Acute thromboembolism is the leading cause of natural death in canine IMHA despite thromboprophylaxis, highlighting the need for novel therapeutic targets.

**Hypothesis/Objectives:** To determine if oxidative stress induces PS externalization in canine erythrocytes (objective 1), and if exposure to antioxidants prevents such changes (objective 2).

**Animals:** Five healthy adult purpose‐bred research beagles.

**Methods:** In vitro, experimental study. Blood was collected from each dog and erythrocytes harvested. For objective 1, erythrocytes were exposed to pro‐oxidant agents tert‐butyl hydroperoxide (TBHP) at 2, 3, or 4 mM, or 2,2′‐azobis(2‐amidinopropane) dihydrochloride (AAPH) at 30, 40, or 50 mM. For objective 2, erythrocytes were exposed to 3 mM TBHP and N‐acetylcysteine‐amide (NACA) at various concentrations (0, 1, or 3 mM). PS externalization was assessed using flow cytometry (Annexin V assay) with median fluorescence intensity (MFI) recorded.

**Results:** Tert‐butyl hydroperoxide at 3 and 4 mM caused increased PS expression in erythrocytes (*P* < .05). AAPH at all concentrations caused increased PS expression (*P* < .01). NACA at all concentrations prevented significant PS expression from erythrocytes exposed to TBHP (*P* < .01).

**Conclusions and Clinical Importance:** Oxidative stress causes PS externalization in canine erythrocytes and NACA ameliorates this effect. Future studies are needed to determine if this occurs in IMHA dogs and its role in promoting thromboembolism.

**Figure:**
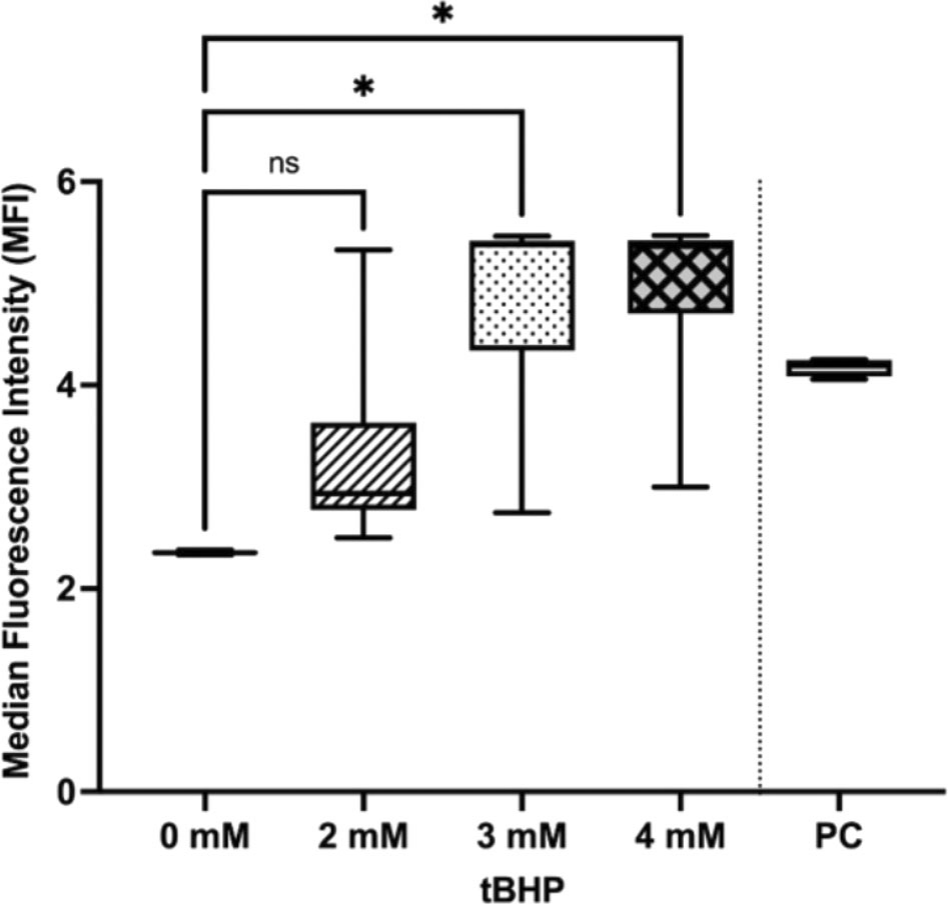
**Figure 1**. tBHP induces PS externalization on canine erythrocyte membrane. Canine erythrocytes PS externalization with different concentrations of tBHP (0, 2, 3, and 4 mM). tBHP exposure at 3 and 4 mM resulted in significantly higher median fluorescence intensity (239 559 ± 289 606, and 231 201 ± 293 923 respectively) as compared to the negative control erythrocytes (223 ± 24), indicating significant increases in phosphatidylserine expression on erythrocytes' plasma membranes. (PC = positive control; ns = not significant; * = *P* < .05; median fluorescence intensity values were log‐transformed.)

**Figure:**
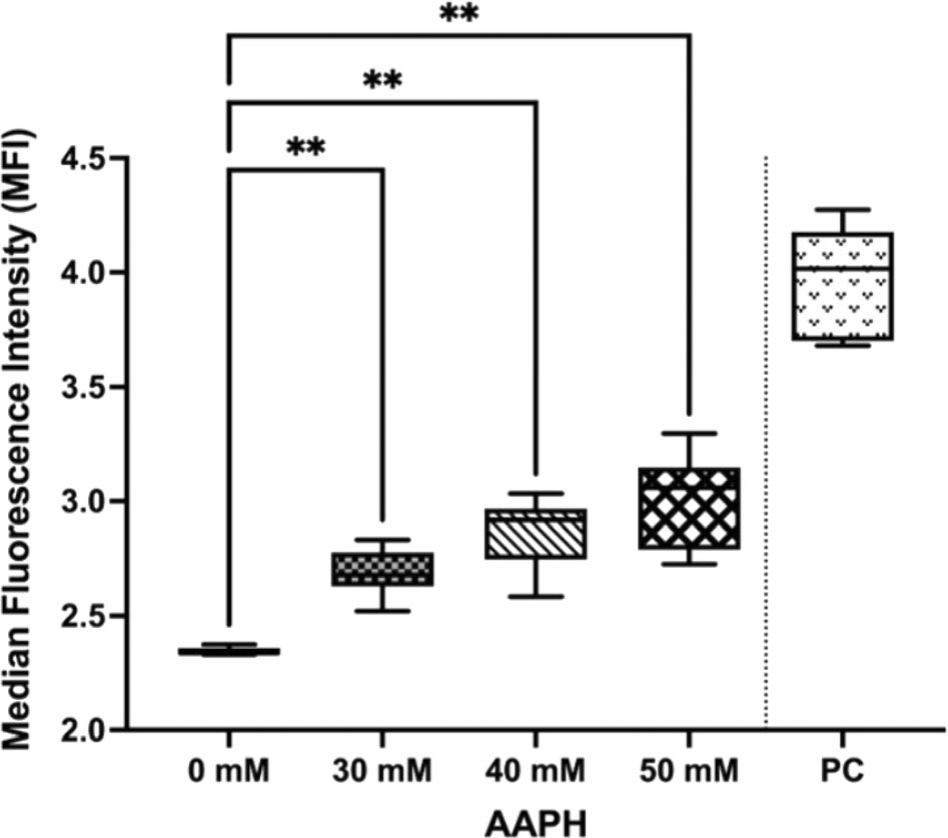
**Figure 2**. AAPH Induces PS externalization on canine erythrocyte membrane. Canine erythrocytes PS externalization with different concentrations of AAPH (30, 40, 50 mM). AAPH exposure at all concentrations resulted in significantly higher median fluorescence intensity (477 ± 348, 833 ± 697, and 1154 ± 1447 respectively) as compared to the negative control erythrocytes (217 ± 23), indicating significant increases in phosphatidylserine expression on erythrocytes plasma membranes. (PC = positive control; ns = not significant; ** = *P* < 0.01; median fluorescence intensity values were log‐transformed.)

## ABSTRACT OT03: Impact of generative AI on veterinary record‐keeping: A case study

### **Kimberly R. Kuhlman** ^1^; Gregory Kuhlman^2^, DVM, DACVIM (SAIM)

#### ^1^AI4Vets Consulting, Sandy, UT, USA; ^2^Red River Animal Emergency Hospital and Referral Center, Sandy, UT, USA

**Background:** The integration of Generative AI into veterinary practices offers potential improvements in record‐keeping efficiency and job satisfaction.

**Hypothesis/Objectives:** This study examines the impact of AI‐driven transcription and summarization tools on the record‐keeping efficiency of a board‐certified small animal internal medicine specialist, hypothesizing a reduction in time spent on records by 5‐10 hours per week.

**Animals:** Not applicable.

**Methods:** A case study approach was employed, focusing on a single veterinarian's experience before and after implementing AI tools for record‐keeping both for client and referring veterinarian consultations and ultrasound examinations. Time spent on documentation was tracked, and qualitative feedback on job satisfaction was gathered.

**Results:** The internist observed a notable decrease in time spent on record‐keeping, calculating a weekly time saving of 5‐10 hours. This reduction in administrative tasks corresponded with an enhanced sense of job satisfaction, primarily due to more time being available for direct patient care.

**Conclusions and Clinical Importance:** The case study underscores the efficacy of Generative AI in optimizing veterinary practice operations. The technology not only alleviates the administrative workload but also contributes to improved job satisfaction and quality of life for practitioners. However, it is crucial to emphasize the need for vigilant human supervision of AI systems to ensure accuracy and reliability. These findings reinforce the significance of integrating advanced technologies in veterinary medicine, not just for operational efficiency but also for enhancing the overall well‐being of veterinary professionals.

## ABSTRACT OT04: Evaluating the efficacy of NxVET, a multi‐functional wearable medical device for animal health monitoring

### **Gerard O'Leary**; Emily Newell

#### University of Toronto, Toronto, ON, Canada

**Background:** Few non‐invasive, wearable medical devices exist to allow for biomarker measurement in canines in out‐of‐clinic environments. Current clinical methods for obtaining biomarkers can be manual and invasive, leading to inaccurate readings.

**Objective:** To assess the efficacy of a non‐invasive device to monitor temperature, pulse, and respiration (TPR) in canines.

**Animals:** A total of 20 previously owned dogs were included within this study. Canines did not present acute or chronic conditions at the time of measurements.

**Methods:** NxVET, equipped with a phonocardiography, temperature sensing, and actigraphy sensing, was tested on a diverse range of canines at the Kingston Humane Society. TPR readings of these canines were obtained using NxVET and subsequently compared to measurements obtained by an anesthetic monitoring machine. All measurements were obtained by a board‐registered veterinarian.

**Results:** NxVET demonstrates a high degree of accuracy in TPR measurements, with a strong correlation to the anesthetic monitoring machine's data. The device's non‐invasive nature allowed for continuous monitoring without causing distress to the animals. Unique insights were gained through the device's ability to detect subtle physiological changes, contributing to proactive health management.

**Conclusion:** NxVET proves to be a valuable tool in veterinary medicine, offering accurate, non‐invasive, and continuous monitoring of vital signs. Its application can enhance animal health management, improve diagnostic accuracy, and potentially lead to better health outcomes in veterinary practice.

## ABSTRACT OT05: Age‐associated changes in the global, untargeted serum metabolome of healthy client‐owned domestic cats

### **Nora Jean Nealon** ^1^; Stacie Summers^2^, DVM, PhD, DACVIM (SAIM); Jessica Quimby^3^, DVM, PhD, DACVIM (SAIM); Jenessa Winston^3^, DVM, PhD, DACVIM (SAIM)

#### ^1^College of Veterinary Medicine, The Ohio State University, Columbus, OH, USA; ^2^College of Veterinary Medicine, Oregon State University, Corvallis, OR, USA; ^3^Veterinary Clinical Sciences, College of Veterinary Medicine, The Ohio State University, Columbus, OH, USA

**Background:** With veterinary medical advances, the cat population is aging. To distinguish health from disease, understanding age‐related metabolism is indicated. Metabolomics, which profiles biomolecules, is routinely used for biomarker discovery.

**Hypothesis/Objectives:** We compared the global, untargeted serum metabolome of clinically healthy young adult versus mature/senior cats using three multivariate approaches.

**Animals:** Healthy, sterilized (male and female) client‐owned cats (n = 7 young adult, aged 1‐6 years).

**Methods:** The serum metabolome was established by Metabolon© using ultra‐high performance liquid chromatography tandem mass‐spectrometry. Metabolite abundances were compared between young and mature/senior cats using a Wilcoxon test, partial least squares regression discriminant analysis variable importance projections (VIP), and random forest mean decrease accuracies (MDA). Metabolites were called meaningful contributors to age differences based on the following criteria: p 1, and/or MDA >0.001094.

**Results:** In total, 914 metabolites were detected, including 209 metabolites differentially abundant between young versus senior cats. These included 37 amino acids, 2 carbohydrates, 13 cofactors/vitamins, 75 lipids, 7 nucleotides, 8 peptides, 37 xenobiotics, and 30 unknown metabolites. A total of 183 metabolites were meaningful contributors to age differences in one model, 20 in two models, and 6 in all three models.

**Conclusions and Clinical Importance:** Aging influences the healthy feline serum metabolome across diverse chemical classes. These changes, along with their multivariate detection approach, should be considered when discriminating between life stages.

## ABSTRACT OT06: Primary hyperlipidemia in miniature schnauzers in Europe

### **Matina N. Pitropaki** ^1^; Isabelle Ruhnke^2^; Mariacristina Vecchio^2^; Panagiotis Xenoulis^1,3^

#### ^1^University of Thessaly, Karditsa, Greece; ^2^Royal Canin; ^3^Texas A&M University, College Station, TX, USA

**Background:** Hyperlipidemia in miniature schnauzers (MS) is the most common form of primary hyperlipidemia in dogs and has been reported to exist in several countries. In the USA the prevalence has been estimated to be around 30%. No studies have been reported regarding the existence of this condition and its prevalence in European countries.

**Hypothesis/Objectives:** We hypothesized that primary hyperlipidemia has the same prevalence in MS in European countries. Our goal was to assess the prevalence of primary hyperlipidemia in MS in selected European countries.

**Animals:** A total of 100 clinically healthy, adult MS from 6 European countries.

**Methods:** Blood samples were collected from all dogs after a 12‐hour fast and serum triglyceride and cholesterol concentrations were measured. All dogs were considered healthy based on history, physical examination, complete blood count (CBC), and biochemical profile.

**Results:** Serum triglyceride concentration was increased (>100 mg/dL) in 26/100 MS (23%), (median 140.5 mg/dL). Most dogs had mild increases in serum triglyceride concentrations (101‐263 mg/dL), while only 3/100 (3%) dogs had moderate to severe hypertriglyceridemia. Serum cholesterol concentration was increased (>320 mg/dL) in 3/100 (3%) dogs (median 357 mg/dL).

**Conclusions and Clinical Importance:** MS in these 6 European countries had a much lower prevalence of hypertriglyceridemia compared to MS in the United States. Further studies in larger populations are needed and under way to verify these results and identify possible genetic differences in these canine populations.

## ABSTRACT OT07: Gastrointestinal antimicrobial resistance in cats treated with antibiotics, a prospective cohort study

### **Amelia Frye** ^1^; Julie Siler^2^; Kelly Sams^3^; Y. Tina Yu^3^; Elizabeth Berliner^2^; Kevin Cummings^3^; Laura Goodman^3^; M. Erin Henry^2^; Lena DeTar^2^ ; Casey Cazer^4^

#### ^1^Comparative Coagulation Section, College of Veterinary Medicine, Cornell University, Ithaca, NY, USA; ^2^Population Medicine and Diagnostic Sciences, Cornell University, Ithaca, NY, USA; ^3^Public and Ecosystem Health, Cornell University, Ithaca, NY, USA; ^4^Clinical Sciences, Cornell University, Ithaca, NY, USA

**Background:** Antimicrobial resistance (AMR) is a growing One Health problem. Judicious antimicrobial use is needed to combat AMR, including identification and selection of antimicrobials less likely to select for AMR in treated patients.

**Objective:** Compare changes in antimicrobial resistance gene content of the gastrointestinal microbiome of cats treated with amoxicillin‐clavulanic acid or cefovecin.

**Animals:** 16 adult, client‐owned or shelter cats treated with amoxicillin‐clavulanic acid (duration <15 days) or cefovecin (one injection). All treatment decisions were at the discretion of the animal's primary veterinarian.

**Methods:** In this prospective cohort study, voided fecal samples from treated cats were collected at enrollment, and at weeks 1 through 4, 8, and 12. Commensal *E. coli* and enterococci isolated from all samples were tested for resistance to cefovecin and other antimicrobials. The baseline and week 2, 8, and 12 samples were tested for 813 AMR genes using deep amplicon next‐generation sequencing.

**Results:** Sequencing of samples from the first seven cats showed increased total AMR genes detected at week 12 compared to baseline. The most common AMR genes conferred resistance to tetracyclines, beta‐lactams, macrolides, aminoglycosides, and phenicols. Cats treated with amoxicillin‐clavulanic acid had more AMR genes than cefovecin‐treated cats at all timepoints. Kirby‐Bauer disk diffusion identified *E. coli* resistant to nalidixic acid, tetracycline, and TMS, and intermediate resistance to ampicillin and amoxicillin‐clavulanic acid.

**Conclusions and Clinical Importance:** Understanding the AMR effects of specific antimicrobials will help shape treatment recommendations and guide veterinarians to make the most responsible antimicrobial selections.

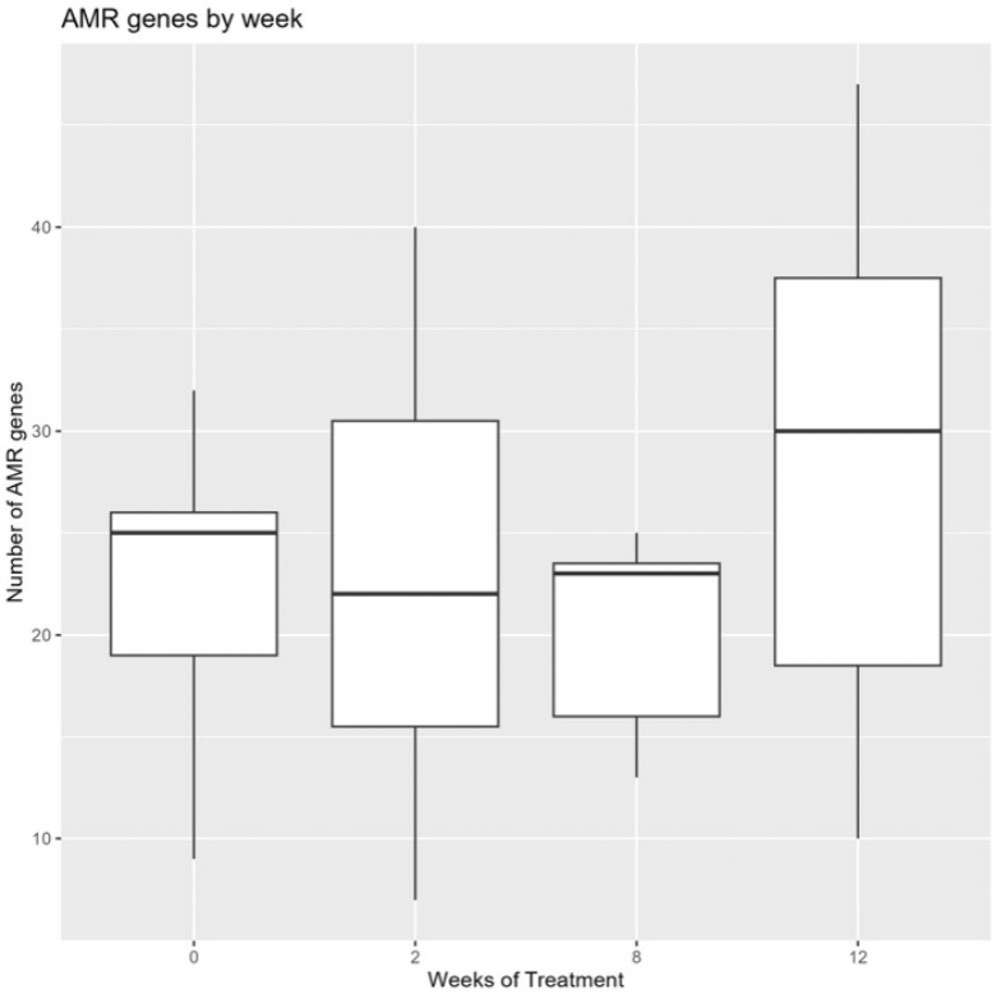

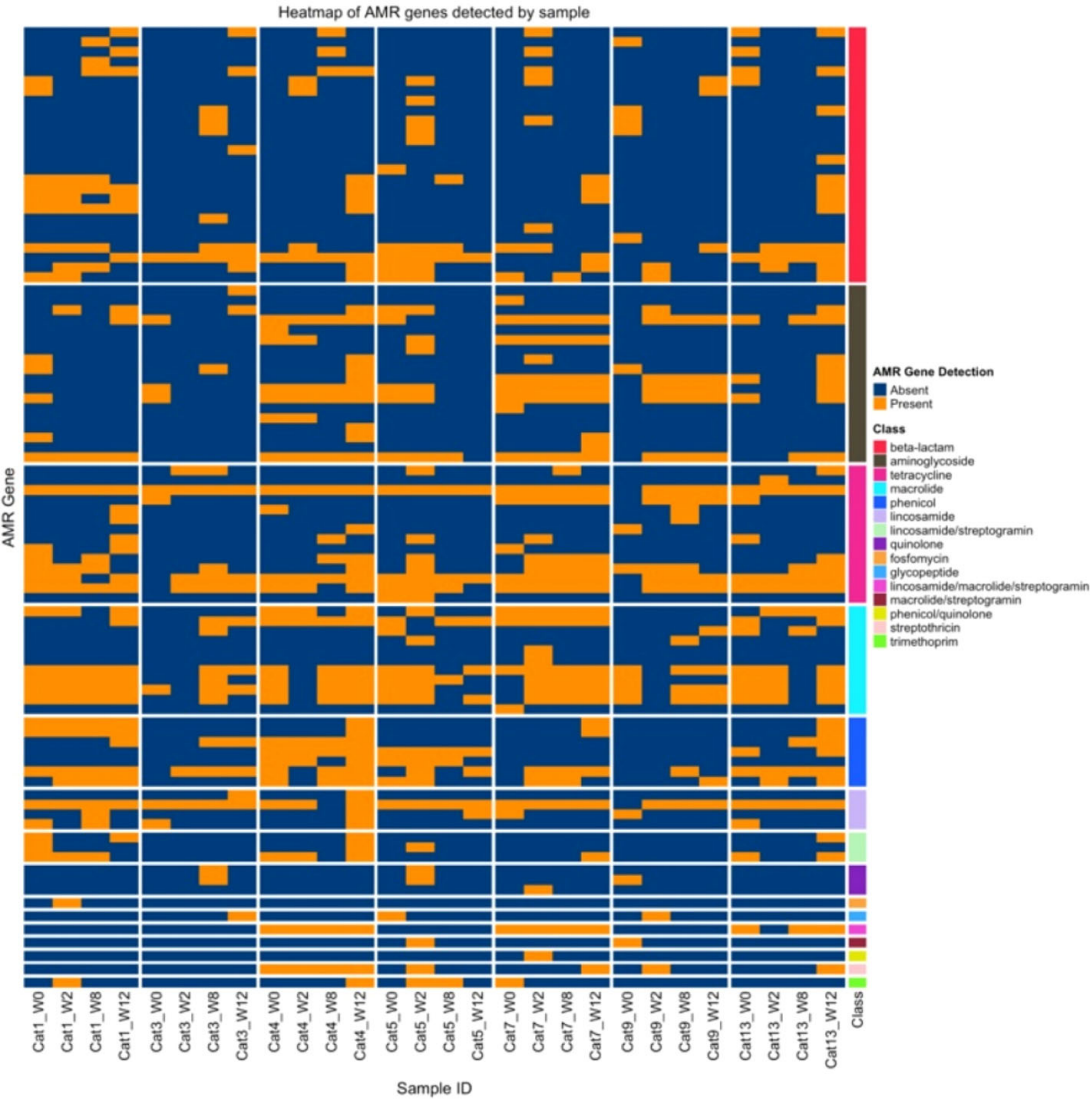

## ABSTRACT PH01: Single‐dose, intravenous and oral pharmacokinetics of isavuconazole in dogs

### **Yishan Kuo** ^1^; Zhong Li^2^, PhD; Lauren Forsythe^3^, PharmD, DICVP; Jeniffer Reinhart^3^, DVM, PhD, DACVIM (SAIM), DACVCP

#### ^1^University of Illinois Urbana‐Champaign, Urbana, IL, USA; ^2^Duke Proteomics and Metabolomics Core Facility, Duke University, Durham, NC, USA; ^3^Department of Veterinary Clinical Medicine, College of Veterinary Medicine, University of Illinois Urbana‐Champaign, Urbana, IL, USA

**Background:** Isavuconazole is a new triazole antifungal drug used in invasive fungal infections in people. It could be an effective therapy for canine mold infections, but limited information is available regarding its use in dogs.

**Objectives:** To evaluate the single‐dose pharmacokinetics of intravenously and orally administered isavuconazole and its safety in dogs.

**Animals:** Six healthy, adult dogs were recruited from a pet population.

**Methods:** After 12‐hour fasting, dogs received 186 mg isavuconazonium sulfate (100 mg isavuconazole equivalent) intravenously and orally with serial blood sampling over 28 days in a two‐way, randomized, crossover design with an 8‐week washout period. Plasma isavuconazole concentrations were measured by liquid chromatography‐mass spectrometry and pharmacokinetic parameters were determined by non‐compartmental analysis.

**Results:** Isavuconazole was well tolerated in each phase. After intravenous administration, clearance, volume of distribution at steady state, and terminal half‐life were 350 ± 112 mL/kg/h, 9.8 ± 4.5 L/kg, and 90 ± 44 h, respectively. After oral administration, the maximum concentration, time to maximum concentration, terminal half‐life, and observed area under the curve were 0.60 ± 0.27 μg/mL, 6.73 ± 2.45 h, 125 ± 80 h, and 7.32 ± 2.38 μg*h/mL, respectively. Oral bioavailability was 81.4 ± 12.8%.

**Conclusions and Clinical Importance:** These results suggest isavuconazole has a long half‐life in dogs and is relatively well‐absorbed orally when administered in the fasted state. Multidose and pharmacodynamic studies are warranted to establish a therapeutic regimen for isavuconazole in dogs.

## ABSTRACT PH02: Comparison of dissolution profiles of human oral generic cyclosporine to Atopica

### **Nicole E. Alleva** ^1^; Hsuan‐Ping Hong^2^, DVM, MVM, MS, DACVIM, CCAT; Joan Coates^3^, DVM, MS, DACVIM; Dawn Boothe^4,5^, DVM, MS, DACVIM (Internal Medicine), PhD

#### ^1^University of Missouri, Columbia, MO, USA; ^2^Assistant Teaching Professor Neurology & Neurosurgery, Veterinary Medicine/Surgery, College of Veterinary Medicine, University of Missouri, Columbia, MO, USA; ^3^Professor, Veterinary Neurology & Neurosurgery, Veterinary Medicine/Surgery, College of Veterinary Medicine, University of Missouri, Columbia, MO, USA; ^4^Alumni Professor, College of Veterinary Medicine, Auburn University, Auburn, AL, USA; ^5^Veterinary Information Network (VIN), Davis, CA, USA

**Background:** Atopica is the pioneer veterinary cyclosporine capsule product. While there are approved generic versions of Atopica, human generic products continue to be commonly prescribed by veterinarians. Presently, there is insufficient evidence to substantiate interchangeability of human oral generic cyclosporine and Atopica in dogs and cats. A potential first step in demonstrating bioequivalence among these products is a dissolution test as described by the U.S. Pharmacopeia (USP). This may provide guidance to appropriate products for in vivo bioequivalence studies in dogs.

**Hypothesis/Objectives:** To determine the dissolution profiles of human generic cyclosporine formulations in comparison to Atopica. The hypothesis is that human oral generic cyclosporine formulations and Atopica have different dissolution profiles.

**Animals:** None.

**Methods:** Dissolution testing was performed in adherence to USP guidelines with Distek 2100 A Tablet Dissolution System with Apparatus II paddles on three human oral generic cyclosporine formulations (Neoral, TEVA, and Apotex) and Atopica. Capsule rupture time was determined by visual inspection. Cyclosporine concentration in the dissolution medium was measured using high‐performance liquid chromatography at 7.5, 15, and 30 minutes.

**Results:** Capsule rupture times were statistically shorter for Neoral compared to Atopica and other human generic preparations. Although numerical concentrations of the Neoral capsule were higher at 7.5, 15, and 30 minutes, there was no statistical difference.

**Conclusions and Clinical Importance:** This study suggests a difference in dissolution profiles between Neoral and Atopica and other human generic preparations, which potentially indicates a variation in vivo. An in vivo pharmacokinetics study is warranted to further substantiate this finding.

**Figure:**
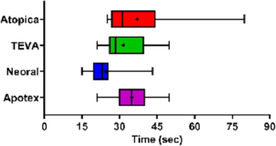
**Figure 1**. Capsule rupture times for Atopica, TEVA, Neoral, and Apotex. Fourteen separate capsule rupture times were determined for each cyclosporine product.

**Figure:**
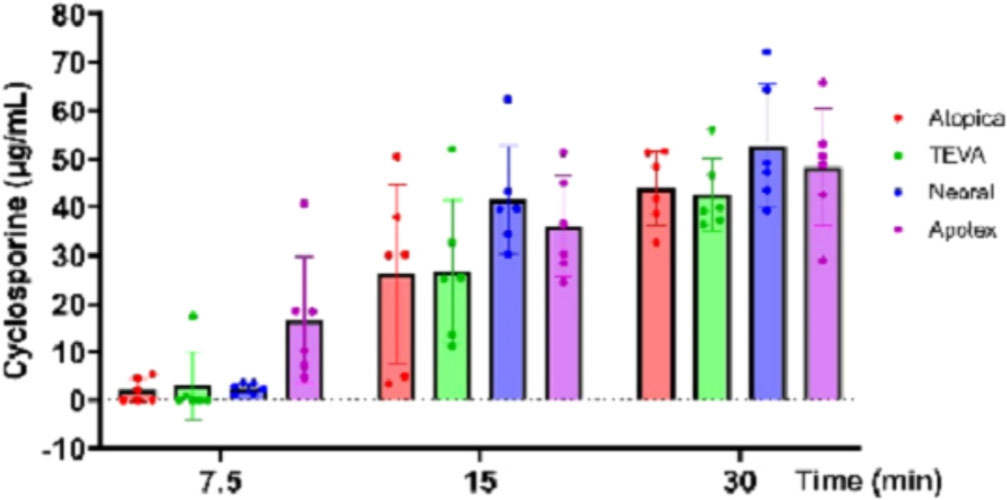
**Figure 2**. Cyclosporine concentrations of Atopica, TEVA, Neoral, and Apotex at 7.5, 15, and 30 minutes. Six samples were collected for each cyclosporine product at each time point.

## ABSTRACT PH03: Palatants increase the voluntary intake of placebo tablets by cats and dogs

### Jean‐Baptiste Jentzer

#### Symrise, Elven, Bretagne, France

**Background:** The generally bitter taste of the active ingredients and excipients used in medicines is often not appreciated by cats and dogs. Pet owners who feel that forced medication is painful for their pet may stop the medication, resulting in therapy failure.

**Objectives:** The study aims to assess the potential of several palatants to increase the voluntary intake of placebo tablets for cats and dogs.

**Animals and Methods:** 14 palatants were tested for their impact on tablet's voluntary intake with a sensory expert panel of 40 cats or dogs. Palatants were added in placebo tablets at 4 concentrations: 0%, 5%, 10%, and 15%. Tablets were presented individually to each animal and their consumption level was measured. All the concentrations of each ingredient were assessed in the same series, at random (14 series in cats, 14 series in dogs).

**Results:** All the palatants tested increase tablet consumption, some more than others. In dogs, on average, the placebo tablets without and with palatants were consumed at 70.9% (SD of 9.3%) and at 93.9% (SD of 3.9%) respectively. In cats, they were consumed at 1.5% (SD of 1.5%) and at 43.2% (SD of 18.0%) respectively. For both species, the dose effect was not significant.

**Conclusions and Clinical Importance:** The addition of palatants increases the consumption of placebo tablets by cats and dogs. This favors voluntary intake and could contribute to the treatment success. The next stage of the study will involve testing the palatants in tablets containing active ingredients.

## ABSTRACT PH04: Bioavailability of oral ondansetron in dogs, a cross‐over study

### **Amanda D. Garrick** ^1^; Kristin Zersen^1^, DVM, MS, DACVECC; Daniel Gustafson^1^, PhD; Jessica Quimby^2^, DVM, PhD, DACVIM (SAIM); Amanda Diaz^3^, DVM; Sarah Shropshire^1^, DVM, PhD, DACVIM (SAIM)

#### ^1^Colorado State University, Fort Collins, CO, USA; ^2^The Ohio State University, Columbus, OH, USA; ^3^University of Minnesota, Minneapolis, MN, USA

**Background:** Ondansetron is a 5‐HT3 serotonin receptor antagonist which is commonly prescribed as an anti‐nausea and anti‐emetic medication for veterinary patients. Ondansetron bioavailability is highly variable among species and there have been no cross‐over studies evaluating the bioavailability of ondansetron in client‐owned dogs.

**Objectives:** To evaluate the bioavailability of ondansetron, comparing oral (PO) to intravenous (IV) administration.

**Animals:** Eight healthy client‐owned dogs.

**Methods:** Dogs were randomized to one of two protocols in a crossover design, receiving PO or IV ondansetron at a dose of 1 mg/kg on Day 0 and the opposite formulation at an equal dose on Day 7. Plasma was collected at baseline and 1, 2, 4, and 8 hours post administration. Ondansetron concentrations were measured utilizing liquid chromatography.

**Results:** For IV administration, mean Cmax was 630±399 ng/ml and AUC0‐8 h was 1181±619 ng/ml*h, with all dogs having detectable plasma concentrations at all time points. For PO administration, mean Cmax was 22 11.3 ng/ml and AUC0‐8 h was 61.7±45.4 ng/ml*h, with all dogs having undetectable concentrations at various time points. Oral mean bioavailability was less than 10%.

**Conclusions and Clinical Importance:** The oral bioavailability of ondansetron is very low in healthy dogs, raising concern for the efficacy of this drug when given orally at 1 mg/kg. Future studies evaluating the pharmacodynamics of ondansetron in nauseous client‐owned dogs should be performed to investigate whether plasma drug concentrations are the optimal way to assess the efficacy of oral ondansetron.

## ABSTRACT PH05: Pirfenidone inhibits TGF‐Β1‐induced fibrosis via downregulation of smad AND ERK pathway in MDCK cells

### **Chaeyoon Im** ^1^; Se‐Hoon Kim^2^; So‐Young Park^2^; Ki‐Hoon Song^3^; Min‐Ok Ryu^2^; Kyoung‐Won Seo^2^; Hwa‐Young Youn^2^

#### ^1^Seoul National University, Seoul, South Korea; ^2^Laboratory of Veterinary Internal Medicine, College of Veterinary Medicine, Seoul National University, Seoul, South Korea; ^3^ViroCure Inc., Seoul, South Korea

**Background:** The prevalence of chronic kidney disease (CKD) in dogs increases with age, and renal fibrosis is an important pathophysiological mechanism in this process. However, only a few drugs that can effectively inhibit fibrosis in the kidneys of dogs are currently available. Pirfenidone is an FDA‐approved drug for human idiopathic pulmonary fibrosis (IPF) which has shown antifibrotic effects in various animal clinical studies.

**Hypothesis/Objectives:** Antifibrotic effects of pirfenidone on canine renal tubular epithelial cells (MDCK) were evaluated.

**Animals:** This is an in vitro experiment using a canine MDCK cell line.

**Methods:** MDCK cells were treated with various concentrations of pirfenidone, followed by transforming growth factor‐beta1 (TGF‐β1) to stimulate fibrotic conditions. A cell viability assay was performed to determine the effect of pirfenidone on cell survival. Fibrosis‐related markers and TGF‐β1 fibrotic pathway‐related markers were assessed using qPCR, western blot analysis and immunocytochemistry.

**Results:** Pirfenidone significantly reduced the expression of profibrotic markers such as α‐smooth muscle actin (α‐SMA), fibronectin, and collagen. Additionally, it upregulated the expression of E‐cadherin, an epithelial marker. Furthermore, pirfenidone effectively inhibited the phosphorylation of key factors involved in the TGF‐β1 pathway, including Smad2/3 and ERK1/2.

**Conclusions and Clinical Importance:** These results demonstrate that pirfenidone modulates TGF‐ β 1‐induced fibrosis in MDCK cells by attenuating key TGF‐ β 1 signaling pathways. These findings highlight the potential of pirfenidone as a therapeutic agent in the treatment of renal fibrosis in end‐stage CKD in dogs after further in vivo research.

## ABSTRACT PH06: The pharmacokinetics of ampicillin sulbactam in azotemic and non‐azotemic dogs

### **Zhe (Alice) Wang** ^1^; Kristin Zersen^1^, DVM, MS, DACVECC; Jessica Quimby^2^, DVM, PhD, DACVIM; Josh Daniels^1^; Daniel Gustafson^1^; Sarah Shropshire^1^, DVM, DACVIM (SAIM), PhD

#### ^1^Colorado State University, Fort Collins, CO, USA; ^2^Veterinary Medical Center, The Ohio State University, Columbus, OH, USA

**Background:** Previous research has shown that azotemic dogs have a lower clearance and higher drug plasma concentrations of ampicillin compared to healthy dogs.

**Hypothesis/Objectives:** Determine the pharmacokinetics of ampicillin after multiple intravenous doses in hospitalized azotemic and non‐azotemic dogs.

**Animals:** Thirty client‐owned dogs; 20 azotemic and 10 non‐azotemic.

**Methods:** Prospective study. Ampicillin‐sulbactam 22 mg/kg intravenously every 8 hours for up to 5 days. Blood samples were obtained at baseline, 1, 4, and 8 hours post‐ampicillin administration each day. Plasma ampicillin was measured using LCMS and non‐compartmental pharmacokinetic modeling and dose interval modeling were performed.

**Results:** Plasma ampicillin exposure (azotemic mean 206.1 ug/ml × hr ± 113.5, non‐azotemic mean 60.3 ± 35.7; *P* < .0009) and half‐life (azotemic mean 3.8 hours ± 2.3, non‐azotemic mean 1.5 hours ± 0.3; *P* < .00001) were statistically greater in azotemic dogs compared to non‐azotemic dogs. Single dose interval modeling showed that 75% of azotemic dogs had >50% time > MIC (MIC = 8) with q12 hour dosing (85% with q8) whereas no non‐azotemic dogs had >50% time > MIC (MIC = 8) with q12 hour dosing (10% with q8). On day 3, 67% of azotemic dogs had at least 50% time > MIC (MIC = 8) with q12 hour dosing (78% with q8).

**Conclusion and Clinical Importance:** Azotemic dogs have significantly higher ampicillin drug exposure and half‐life. Over multiple days of hospitalization, the majority of azotemic dogs continued to have greater than 50% time > MIC (MIC = 8) at q12 hour dosing.

## ABSTRACT RS01: Comparison of sedated respiratory‐gated computed tomography (CT) to anesthetized inspiratory:expiratory breath hold CT in dogs

### **Iliana M. Navarro** ^1^; Aida Vientós‐Plotts^2^, DVM, PhD, DACVIM (SAIM); Carol Reinero^3^, DVM, PhD, DACVIM (SAIM); Isabelle Masseau^4^, DVM, PhD, MSc, DACVR

#### ^1^University of Missouri, Columbia, MO, USA; ^2^Assistant Professor, Veterinary Internal Medicine, Department of Veterinary Medicine and Surgery, University of Missouri, Columbia, MO, USA; ^3^Professor and Director, Comparative Internal Medicine Laboratory, Department of Veterinary Medicine and Surgery, University of Missouri, Columbia, MO, USA; ^4^Faculté de Médecine Vétérinaire, Department of Sciences Cliniques, Université de Montréal, Saint‐Hyacinthe, QC, Canada

**Background:** Ventilator‐assisted inspiratory: expiratory breath‐hold computed tomography (I:E‐BH CT) is superior to sedated CT but poses anesthetic risk. Reduction of motion artifact and ability to interpret inspiratory and expiratory phases can be overcome with sedated respiratory gated CT (RG‐CT). Comparison of CT lung patterns (increased attenuation, decreased attenuation, nodular and linear patterns) and their sub‐patterns have not been compared between I:E‐BH CT and RG‐CT in dogs.

**Hypothesis:** We hypothesized that in dogs with respiratory signs, sedated RG‐CT would be a minimally‐invasive surrogate for anesthetized I:E‐BH CT with no significant difference in presence of the four major CT lung patterns and their sub‐patterns.

**Animals:** Forty‐one client‐owned dogs with respiratory clinical signs.

**Methods:** Sedated RG‐CT and anesthetized I:E‐BH CT images were prospectively acquired. A blinded board‐certified radiologist assessed all scans for the presence of CT lung patterns and sub‐patterns. For each dog, a Fisher's exact test was used to determine if there were nonrandom associations between the two scan types for each variable (significance, *P* < .05).

**Results:** Motion artifact was minimal with both types of scans. For presence of the four major lung patterns and 14 sub‐patterns, there was no significant difference between scan type (*P* < .05 for all).

**Conclusions and Clinical Importance:** Lung patterns and sub‐patterns from I:E‐BH and RG‐CT scans are comparable in dogs with respiratory disease. Superior technique and detail of anesthetized I:E‐BH CT allows for increased conspicuity of subtle sub‐patterns than RG‐CT in some dogs.

